# A Farewell to the Narcissism Epidemic? A Cross‐Temporal Meta‐Analysis of Global NPI Scores (1982–2023)

**DOI:** 10.1111/jopy.12982

**Published:** 2024-10-14

**Authors:** Sandra Oberleiter, Paul Stickel, Jakob Pietschnig

**Affiliations:** ^1^ Department of Developmental and Educational Psychology, Faculty of Psychology University of Vienna Wien Austria

**Keywords:** cross‐temporal meta‐analysis, narcissism, narcissistic personality inventory

## Abstract

**Objective:**

Several recent accounts have failed to replicate the so‐called Narcissism Epidemic, suggesting potential influences of the Global Financial Crisis (GFC) in 2008 as a reason for narcissism trend reversals. Here, we provide evidence for narcissism test score changes from 1982 to 2023.

**Methods:**

We investigated self‐report data on the Narcissistic Personality Inventory (NPI) from 1105 studies (*k* = 1621, *N* = 546,225) using precision‐weighted cross‐temporal meta‐analysis.

**Results:**

Data collection years were meaningfully negatively associated with narcissism scores in virtually all analyses (*b*s: −0.409 to −0.008; partial eta square's: < 0.001 to 0.118; *p*s: < 0.001 to 0.174), thus indicating cross‐temporally decreasing narcissism self‐report scores. Examination of regression segments pre‐ and post‐dating the GFC and segmented line regressions indicated mostly stable narcissism scores during the 1980s and 1990s that subsequently showed negative slopes with somewhat differing decreases onsets according to analytical subsets.

**Conclusions:**

Here, we provide evidence for negative cross‐temporal changes in narcissism from 1982 to 2023 globally, thus contrasting the idea of a Narcissism Epidemic having taken place at any point during the past four decades. Changes appear to generalize across different regions and participant sex, although mean scores were differentiated, yielding higher narcissism values for North American and younger samples.

## 1 Introduction

Over the past decades, several studies documented cross‐temporal changes in personality traits in the general population (e.g., Curran and Hill 2019; Hamamura, Johnson, and Stankovic 2020; Konrath, O'Brien, and Hsing 2011; Twenge and Campbell 2001). In particular, evidence for substantial cross‐temporal narcissism increases in U.S. college students has garnered considerable attention (Twenge et al. 2008). A cross‐temporal meta‐analysis showed an increase in mean self‐reported scores on the Narcissistic Personality Inventory (NPI) among U.S. college students between 1982 and 2006 based on 85 independent samples (*N* = 16,475). However, these findings have been controversially discussed in the scientific community (e.g., Arnett 2013; Trzesniewski and Donnellan 2010; Twenge 2013).

Further empirical studies seemed to corroborate cross‐temporal narcissism increases (e.g., Gentile, Twenge, and Campbell 2010; Smits et al. 2011; Twenge and Campbell 2001), thus sparking a debate about the emergence of a so‐called “Generation Me”. In this vein, it has been suggested that (sub‐clinical) self‐absorbed, egotistic, and narcissistic behaviors may increasingly characterize generations of young adults (Twenge 2013) and that these changes may be due to increasing individualism within Western societies (Bianchi 2016; Greenfield 2016; Grossmann and Varnum 2015). Possible candidate causes have been suggested to be related to cross‐temporal increases in self‐esteem (Gentile, Twenge, and Campbell 2010; Twenge and Campbell 2001), decreases in empathy (Konrath, O'Brien, and Hsing 2011), or decreases in trust in the community and religious institutions (Twenge, Campbell, and Carter 2014).

Another explanation for the observed positive time trends in the narcissistic traits of students is rooted in the idea that the narcissism self‐report values of women may have cross‐temporally approached those of men. Typically, women self‐report lower narcissism values than men (Grijalva et al. 2015). However, previous cross‐temporal meta‐analytical evidence indicated that NPI scores of women have increased over time, while those of men have not (Twenge et al. 2008). Decreasing narcissism sex differences over time have been suggested to be attributable to an increase of agentic traits and assertiveness in women due to societal change, which in turn affects self‐reported narcissism (Twenge 1997, 2001). Increases of narcissism in women, but not men, would thus be expected to lead to a general increase in narcissism self‐reports. The idea of changes in narcissism sex differences is contrasted by another meta‐analysis that showed no evidence for a convergence of scores of men and women (Grijalva et al. 2015).

In the light of recent failures to replicate positive U.S.‐based college student narcissism self‐report score trajectories, it has been suggested that narcissism changes may have followed a non‐linear trajectory, with values peaking around 2008. Some authors have attributed this to the effects of the Global Financial Crisis (GFC) on the development of narcissistic traits (e.g., Twenge et al. 2021). Specifically, it has been theorized that lost job prospects of youths due to a bleak economic outlook in the U.S. may have impacted the narcissism scores of post‐GFC youths; entering adulthood during a financial recession may reduce the likelihood of developing a narcissistic personality (Bianchi 2014). Therefore, such an effect can be expected to emerge most prominently in young adults from the United States. At least one study supports this idea by suggesting a non‐linear trajectory with a supposed shift of the observed narcissism pattern among U.S. college students in 2008 (Twenge et al. 2021).

Because of these conflicting findings, to date, it seems unclear whether the suspected Narcissism Epidemic has taken place (for an overview, see Grubbs and Riley 2018). It is unclear if the previously observed patterns of findings (i) represent trends that are still ongoing, (ii) generalize to non‐U.S. samples, (iii) represent genuine cross‐temporal changes in narcissism self‐reports of the general population (or college students), and (iv) may be due to non‐linearity of changes because of isolated events (e.g., the Global Financial Crisis) that conceivably may have had a large‐scale impact on personality traits in the general population (Twenge et al. 2021). To contribute to clarifying these questions, we present a cross‐temporal meta‐analysis to assess global NPI‐based narcissism changes from 1982 to 2023.

## 2 Methods

The present study was preregistered on the Open Science Framework (OSF) prior to accessing any data. The preregistration protocol and any deviations from the preregistration are available at https://osf.io/vzebk and https://osf.io/jzvfu. Supporting Information, including supplementary results, primary data, and the analysis code, can be retrieved from https://osf.io/fcwem/files/osfstorage#.

### 2.1 Literature Search

**FIGURE 1:**
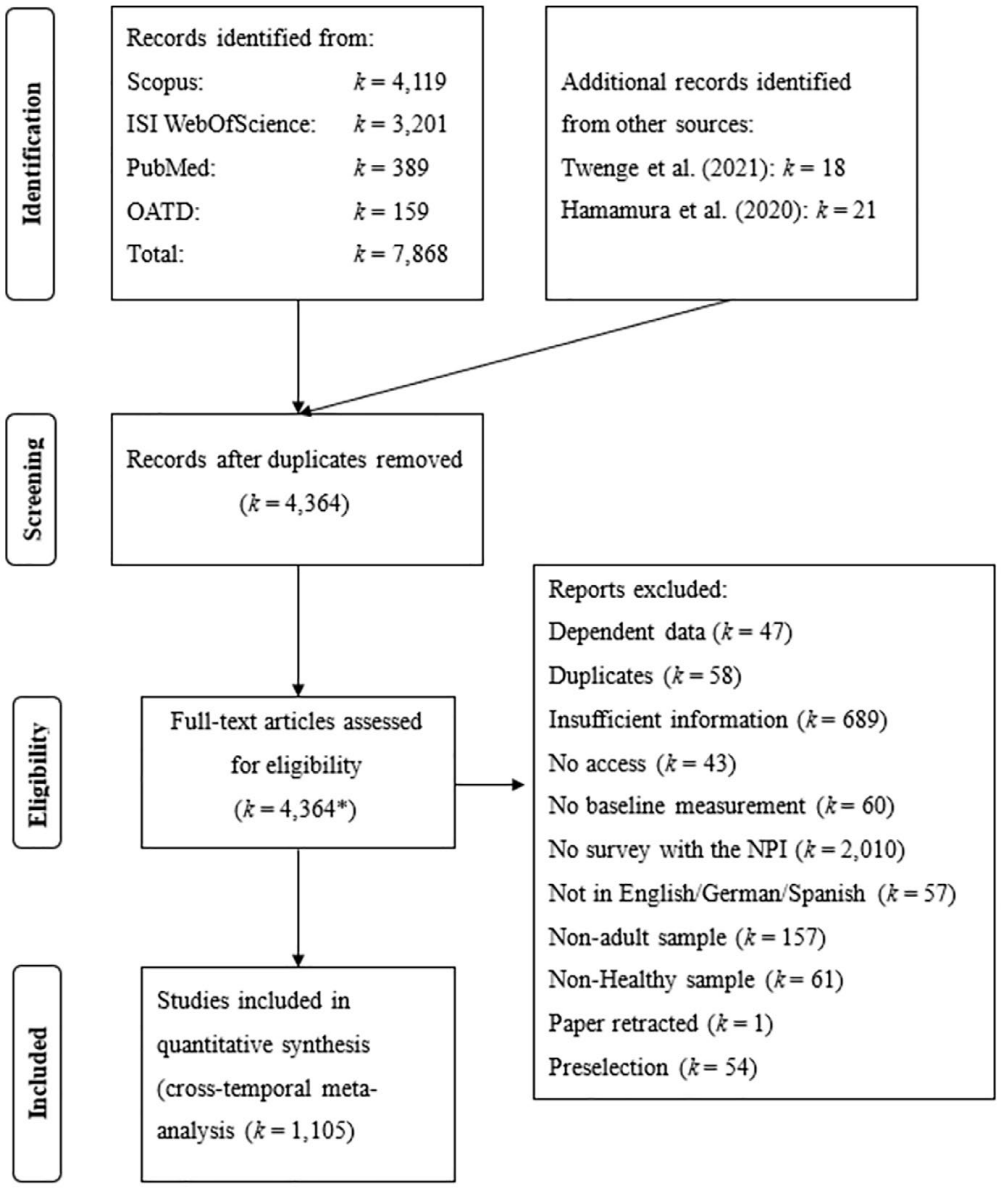
PRISMA flowchart of the study selection process. *Note:* *Data from 22 unpublished articles, as provided by Twenge et al. (2021) and Hamamura, Johnson, and Stankovic (2020), were included based on data reported in these secondary sources.

First, we conducted a cited reference search for Raskin and Hall (1981, 1979), Raskin and Terry (1988), Ames, Rose, and Anderson (2006), Emmons (1984), Gentile et al. (2013), and Schütz, Marcus, and Sellin (2004). This strategy was deemed reasonable because these papers provide NPI items in different languages and validation studies and are, therefore, likely to be cited by studies in which the NPI has been used. The literature search was conducted on May 21, 2021, and updated and concluded on January 15, 2024. This search yielded 4119 records on *Scopus*, 3201 on Web of Science, and 389 on *PubMed*. Unpublished articles were identified by searching the *Open Access Theses and Dissertation* (*OATD*; https://oatd.org) database for the search string “NPI OR Narcissistic AND Personality AND Inventory” (159 hits). In all, full texts of 4364 records were obtained after deduplication. A PRISMA flowchart of the study selection process is provided in Figure 1. The references of all included articles are provided in the Supporting Information (https://osf.io/vgspf).

### 2.2 Inclusion Criteria

Studies had to meet five criteria to be eligible for inclusion in the present meta‐analysis. First, they had to report fullscale scores of the NPI. In cases where only subscale scores were reported, we calculated fullscale scores. Second, samples had to comprise healthy adults (i.e., mean sample ages > 18 years) that had not been purposefully recruited to yield untypical narcissism scores (i.e., extreme groups). Third, sample mean NPI scores, participant numbers, number of NPI items, and response formats had to be reported. If data from multiple measurement times were reported (e.g., in experimental studies), only baseline scores were included. Fourth, only studies in English, German, or Spanish were included. Fifth, in cases of data dependencies, data from the largest and earliest published sample were used.

### 2.3 Coding

The coding of the studies was performed twice independently by the same experienced researchers [S.O., P.S.]. Coding inconsistencies were resolved through discussion with another independent coder [J.P.]. For each sample, author names and publication years were recorded. If the data collection year was not provided, it was coded as 2 years prior to publication for published studies or 1 year prior to publication for unpublished studies, following standard meta‐analytical protocol (e.g., Pietschnig, Voracek, and Formann 2010; Twenge et al. 2008). Furthermore, we recorded (i) data collection country and continent (Africa vs. Asia vs. Europe vs. Oceania vs. North America vs. South America), (ii) mean sample ages, (iii) publication status (published vs. unpublished), (iv) sample type (student vs. community sample), (v) NPI version, (vi) number of NPI‐response options (forced choice vs. polytomous scale), and (vii) statistical parameters. Moreover, scores of seven NPI‐subscales for the NPI versions comprising between 37 and 54 items were coded by an additional researcher [J.P.] to obtain (response scale‐adjusted) scores for authority, exhibitionism, superiority, entitlement, exploitativeness, self‐sufficiency, and vanity (Raskin and Terry 1988). The complete datasets are provided in our Supporting Information (https://osf.io/fcwem/files/osfstorage).

### 2.4 Data Analyses

We used precision‐weighted (i.e., larger samples being assigned larger weights) regressions to predict NPI scores by publication year. First, we ran our analyses to match the inclusion criteria of the original reports about the Narcissism Epidemic (Twenge et al. 2008, 2021; in our analyses, narcissism self‐reports from any U.S.‐based college were included in contrast to Twenge et al. 2008, 2021; meta‐analyses that included only data from specific U.S. colleges). This means we only included data from U.S. student samples that self‐reported responses on the NPI‐40 using a forced‐choice format. Subsequently, we used multiple linear precision‐weighted meta‐regressions to assess additional potential influences of age and sex as predictors of narcissism. Then, residualized cross‐temporal changes were calculated by first predicting NPI mean scores by sex and age and subsequently running a single regression of data collection year on residuals, to obtain age‐ and sex‐controlled change trajectories.

Second, we repeated these analyses by including data from all available samples to assess the generality of the narcissism time trends. Then, we reran our analyses for data subsets according to geographical regions (Asia, Europe, North America, Oceania, USA) and sample types (i.e., all samples vs. student samples only) that comprised a minimum of 20 samples from 1982 to 2023. For the above analyses, NPI scores from non‐forced‐choice NPI formats were transformed proportionally into the same ratio of forced‐choice‐based NPI‐40 scores, using the respective scale maxima and minima as anchor points. This procedure was adopted following the approach of Hamamura, Johnson, and Stankovic (2020) and calculated as described in Card (2015). For an overview of the analyses, see Table 1.

**TABLE 1:** Overview of the different data sets that were analyzed.

Region	Subgroup	Total time span		Before the GFC (until 2008)		After the GFC (since 2008)	
Global	All samples	1595 (814)	X	390 (278)	X	1231 (536)	X
	Students	868 (522)	X	307 (221)	X	561 (301)	X
Asia	All samples	176 (52)	X	22 (18)	—	154 (34)	—
	Students	69 (25)	X	11 (9)	—	58 (16)	—
Europe	All samples	430 (251)	X	43 (19)	—	387 (133)	—
	Students	142 (55)	X	18 (9)	—	124 (46)	—
North America	All samples	864 (543)	X	312 (232)	—	552 (311)	—
	Students	629 (430)	X	272 (198)	—	357 (232)	—
Oceania	All samples	54 (21)	X	8 (5)	—	46 (16)	—
	Students	21 (10)	—	5 (4)	—	16 (6)	—
USA	All samples	748 (469)	X	286 (212)	X	462 (257)	X
	Students	541 (371)	X	253 (185)	X	288 (186)	X

*Note:* Numbers specify the included samples; samples that completed the NPI‐40 with dichotomous response format are shown in parentheses; an X indicates that sufficient data were available to run analyses; analyses pre‐ and postdating the GFG were only conducted for global and U.S.‐based samples.

Abbreviation: GFC, Global Financial Crisis.

Finally, to examine cross‐temporal trends before and after the GFC, separate models were calculated for the period up to 2008 and after 2008 for global and U.S.‐based samples, for forced‐choice‐based NPI‐40 and any NPI formats, respectively. Potential differences in change trajectories according to sex were examined by an interaction term of data collection year and sex in global and U.S.‐based subset analyses. The alpha level was set at 0.05, and effect sizes were interpreted according to well‐established thresholds into small, moderate, or large effects (i.e., lower thresholds for partial eta squared effect strengths = 0.01, 0.06, and 0.14, respectively; Cohen 2013).

#### 2.4.1 Exploratory Analyses

We exploratorily examined potential systematic changes in the NPI trajectory using segmented line regression (Kim et al. 2000; Kim, Yu, and Feuer 2008) in U.S.‐based and global data (sub‐)sets. We used the grid search algorithm (Lerman 1980) to assess a possible non‐linearity of change trajectories when assuming a maximum number of two joinpoints. In this procedure, significant slope changes across time would be modeled by up to two joinpoints that connect regression segments with differing slopes.

In further exploratory analyses, we examined the influences of data collection region. In this context, we introduced the continent of data collection as an additional dummy‐coded factorial predictor in addition to the data collection year in the multiple regression models, using North America and, in further analyses, the USA as the reference category.

All statistical analyses were conducted using the open‐source software *R v.4.3.2* (R Core Team 2023) and the *Joinpoint Regression Program 4.9.0.0* (Kim et al. 2000).

### 2.5 Final Sample

**FIGURE 2:**
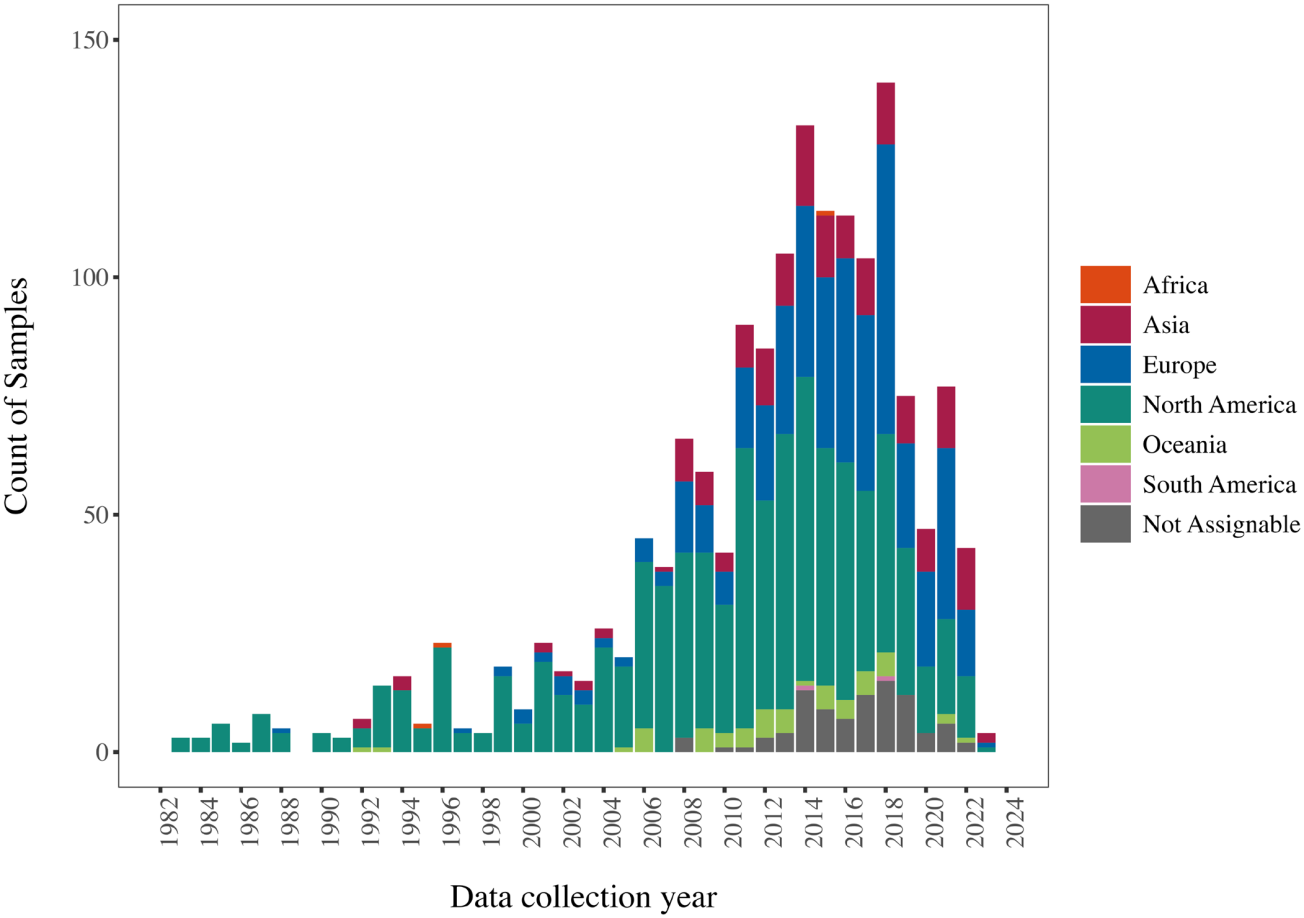
Number of samples according to data collection year and continent. [Color figure can be viewed at wileyonlinelibrary.com]

In all, a total of *k* = 1621 (*N* = 546,225) independent samples from 1105 published and unpublished studies from 55 different countries were included in this meta‐analysis (see Figure 2). Samples consisted either of students (*k* = 868) or general population participants (*k* = 753). Samples were predominantly administered the NPI‐40 (*k* = 890) and the NPI‐16 (*k* = 384), with the remaining samples having been administered another version. In terms of response formats, most scales used a forced‐choice format (*k* = 1366) or a 5‐point response scale (*k* = 142), with the remainder having used other scales. The mean sample age was 26.85 years (SD = 8.54) and samples comprised 55.36% women.

## 3 Results

### 3.1 Overall Time Trends (1982–2023)

**FIGURE 3:**
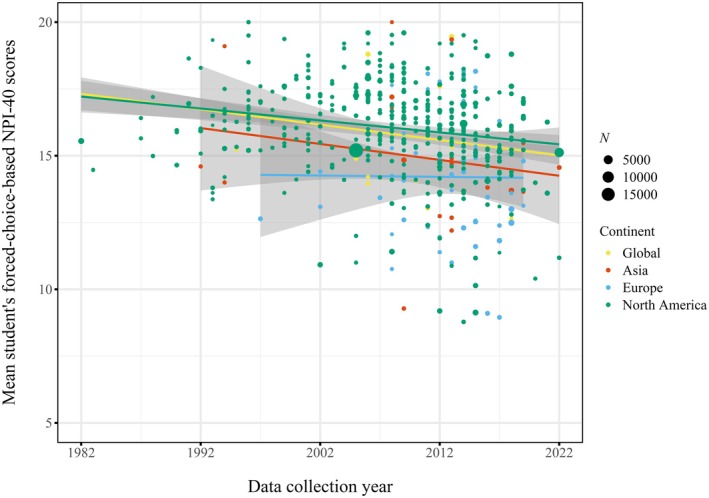
Global and continental single regressions for student samples on forced‐choice‐based NPI‐40 scores. [Color figure can be viewed at wileyonlinelibrary.com]

In the single linear weighted meta‐regressions, we observed negative trajectories of forced‐choice‐based NPI‐40 scores over time in U.S.‐based student samples (*k* = 371; *b* = −0.053; partial eta squared = 0.023; *p* < 0.001; see Table 2, Figure 3). This pattern of small‐to‐moderate effect strengths generalized across all NPI formats (*b* = −0.071, partial eta squared = 0.056; *p* < 0.001) and when examining all samples for both forced‐choice‐based NPI‐40 scores and other NPI formats (*b* = −0.108; partial eta squared = 0.068; *p* < 0.001 and *β* = −0.268; partial eta squared = 0.033; *p* < 0.001, respectively).

**TABLE 2:** Single and multiple linear weighted meta‐regression as well as residualized regression for U.S.‐based student samples on forced choice‐based NPI‐40 scores from 1982 to 2023.

Predictors	Model fit	*k*	*b*	SE	$\eta_{p}^{2}$	*p*
Single regression						
	*R* ^*2*^ = 0.020**; *F* (1, 369) = 8.60**	371				
Year of data collection (1982–2023)			−0.053	0.018	0.023	0.004
Multiple regression						
	*R* ^*2*^ = 0.093***; *F* (3, 246) = 9.52***	249				
Year of data collection (1982–2008)			−0.068	0.025	0.043	0.003
Sample mean age			0.050	0.156	0.001	0.751
Percentage of women in sample			−0.034	0.008	0.064	< 0.001
Regression on residualized values						
	*R* ^*2*^ = 0.002*; *F* (1, 248) = 6.61*	249				
Year of data collection^a^ (1982–2023)			−0.065	0.025	0.026	0.010

*Note:* Variables were weighted based on sample size; all *R* ^2^ are adjusted values; *k* = number of samples; *b* = unstandardized regression coefficient; SE = standard error of unstandardized coefficient; *β* = standardized regression coefficient; $\eta_{p}^{2}$ = partial eta squared; Variance Inflation Factors (VIFs) in multiple regression were all < 1.1.

^a^ Controlled for sex and age (i.e., percentage of women in sample and sample mean. age).

* *p* < 0.05.

** *p* < 0.01.

*** *p* < 0.001.

**FIGURE 4:**
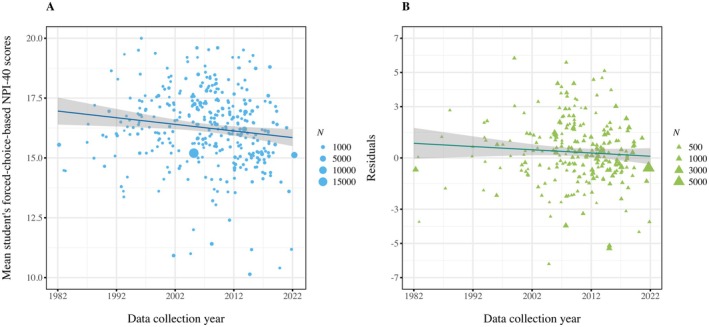
Changes in forced‐choice‐based NPI‐40 scores of US‐based student samples for single (panel A) and residualized regressions (panel B) from 1982 to 2023. [Color figure can be viewed at wileyonlinelibrary.com]

Consistent with the U.S.‐based trajectory, our global analyses revealed negative trends in narcissism over time (see Figure 4). These negative trajectories generalized over NPI format and sample type, yielding small‐to‐large effects (forced‐choice NPI‐40 student samples: *b* = −0.072; partial eta squared = 0.039; *p* < 0.001; any NPI format student samples: *b* = −0.096; partial eta squared = 0.041; *p* < 0.001; forced‐choice NPI‐40 all samples: *b* = −0.159; partial eta squared = 0.117; *p* < 0.001; any NPI format all samples: *b* = −0.145; partial eta squared = 0.050; *p* < 0.001).

Continent‐based analyses showed broadly consistent patterns, yielding negative changes in Europe and North America for both forced‐choice‐based NPI‐40 and all NPI formats in student and overall samples (*b* range: −0.379 to −0.057; partial eta squared range: 0.028 to 0.118; all *p*s < 0.001). Changes in Asian and Oceanian samples were predominantly negative (excepting a non‐significant positive slope in student samples for any NPI formats), although not all regression slopes reached nominal statistical significance (*b* range: −0.291 to 0.019; partial eta squared range: < 0.001 to 0.091; *p* range: < 0.001 to 0.174). Numerical results are detailed in the Tables S1–S21.

In supplementary analyses, we examined cross‐temporal changes of seven NPI subscales (Raskin and Terry 2008; *k* = 72–75; *N* = 20,864‐21,360): authority (*b* = −0.008, *p* = 0.25), exhibitionism (*b* = −0.008, *p* = 0.28), superiority (*b* = −0.018, *p* = 0.01), entitlement (*b* = 0.020, *p* = 0.01), exploitativeness (*b* = 0.017, *p* = 0.01), self‐sufficiency (*b* = 0.013, *p* = 0.06), and vanity (*b* = 0.004; *p* = 0.47).

### 3.2 Moderator Analyses

#### 3.2.1 Age

Age was observed to be negatively associated with NPI scores across virtually all samples, indicating lower self‐reported NPI scores in older individuals. This association emerged in both forced‐choice‐based NPI‐40 and other NPI formats in global, U.S.‐based, North American, as well as Oceanian data (*b* range: −0.227 to 0.050; partial eta squared range: 0.001 to 0.106; *p* range: < 0.001 to 0.751). However, age influences were considerably smaller and mostly non‐significant in Asian and European samples (*b* range: −0.219 to 0.757; partial eta squared range: < 0.001 to 0.155; *p* range: 0.008 to 0.871). Numerical details are provided in Table 2 and Tables S1–S21 in the Supporting Information (https://osf.io/cdsb3).

#### 3.2.2 Sex

The percentage of women within a sample was consistently negatively associated with NPI scores across all formats, yielding small‐to‐moderate effects in terms of strength (*b* range: −0.038 to −0.017; partial eta squared range: 0.017 to 0.102; *p* range: < 0.001 to 0.025). This suggests that women report lower NPI scores compared to men. However, results from Asian and Oceanian samples yielded less unequivocal sex effects, yielding predominantly trivial‐to‐small effects that mainly were statistically non‐significant (*b* range: −0.038 to < 0.001; partial eta squared range: 0.007 to 0.037; *p* range: 0.038 to 0.915). Numerical details are provided in Table 2 and Tables S1–S21.

#### 3.2.3 Interaction Effects of Sex Ratio With Data Collection Year

In our primary analysis of forced‐choice‐based NPI‐40 scores of U.S.‐based student samples, we observed a significant interaction between sex ratio and data collection year (*R* ^2^ = 0.092***; *F*(3, 336) = 12.58***; *b* = < 0.001; partial eta squared = 0.018, *p* = 0.012). This finding suggests smaller cross‐temporal narcissism changes in samples with larger percentages of women within U.S.‐based student samples. This was unexpected because it contrasts our expectation of narcissistic values of women cross‐temporally converging with those of men. However, no significant interaction was observed in any of the other (sub‐)analyses (all *b*s < 0.001; partial eta squared range: < 0.001 to 0.055; *p* range: 0.083 to 0.650).

#### 3.2.4 Influences of Region

Supplementary exploratory analyses revealed that European students consistently reported significantly lower narcissism scores compared to U.S.‐based and North American students across both forced‐choice and all NPI formats (*b* range: −2.065 to −1.042; all *p*s < 0.001). Furthermore, regardless of sample type or NPI format, we observed consistently lower self‐reported narcissism scores among Asians compared to U.S.‐based and North American participants (*b* range: −2.679 to −2.546; all *p*s < 0.001).

### 3.3 Change Trajectories Before and After the Global Financial Crisis

Analyses of U.S.‐based narcissism values before the GFC (from 1982 to 2008) revealed predominantly trivial effects that yielded inconsistent signs between subsamples of students vs. all participants and different NPI formats (*b* range: −0.012 to 0.042; partial eta squared range: < 0.001 to 0.014; *p* range: 0.047 to 0.965). No significant and meaningful effects were observed in global analyses (*b* range: 0.015 to 0.021; partial eta squared range: 0.001 to 0.002; *p* range: 0.360 to 0.552), except a small, positive, and statistically significant effect for student samples across all NPI formats (*b* = 0.043; partial eta squared = 0.015; *p* = 0.033).

**FIGURE 5:**
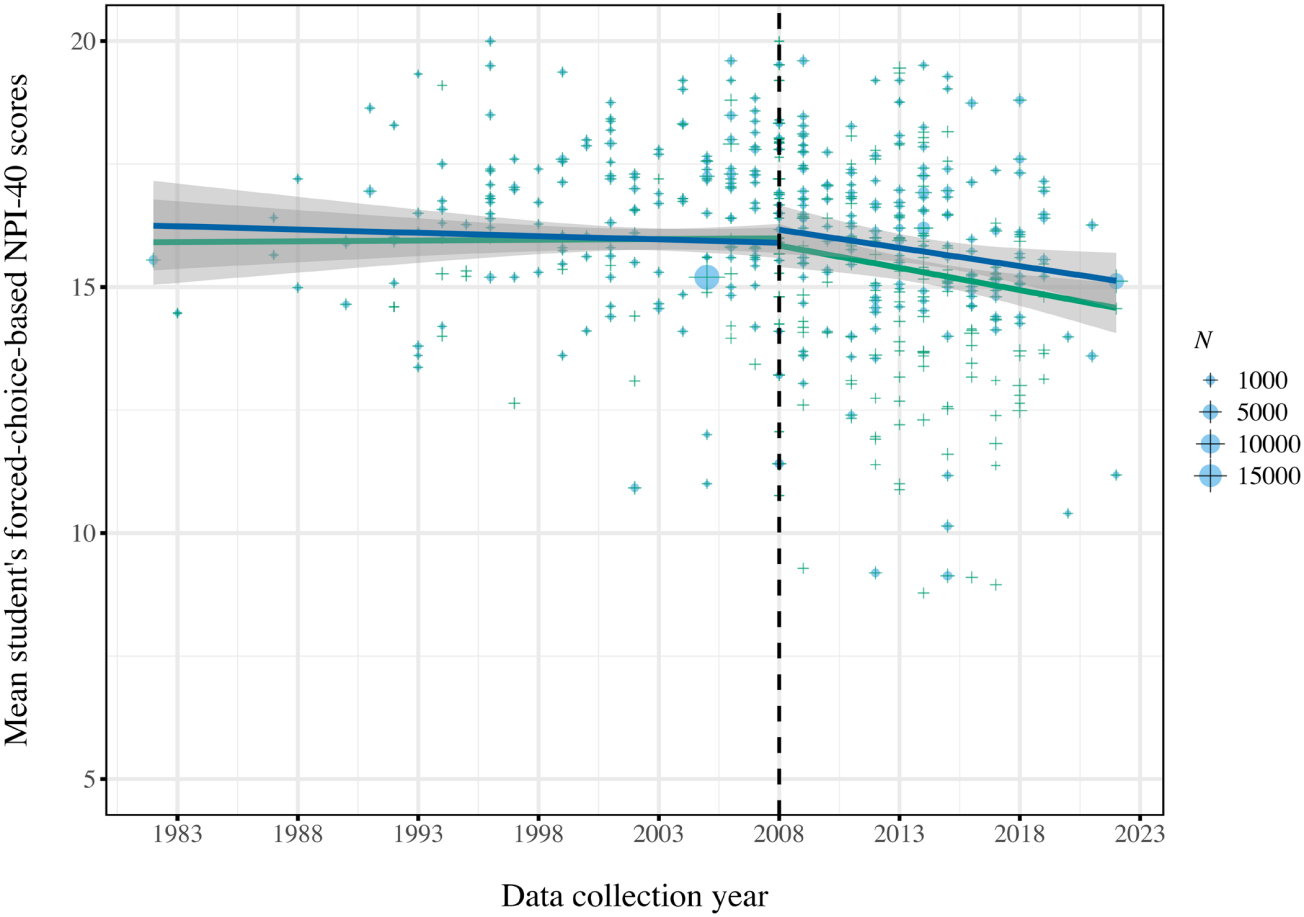
Changes in forced‐choice‐based NPI‐40 scores of U.S.‐based (blue) and global (green) student samples before (1982–2008) and after (2008–2023) the Global Financial Crisis. [Color figure can be viewed at wileyonlinelibrary.com]

However, following the onset of the GFC in 2008, negative cross‐temporal changes in narcissism scores emerged across both U.S.‐based and global student and overall samples, irrespective of the used NPI format (*b* range: −0.347 to −0.180; all *p*s < 0.001). Effects were small‐to‐large in terms of strength, with partial eta squares ranging from 0.038 to 0.130 (see Figure 5; numerical results are detailed in Tables S22–S43).

### 3.4 Segmented Line Regressions

We explored possible changes in the cross‐temporal trajectory by examining students and all participants from U.S.‐based and global samples in segmented line regressions fitting a maximum of two joinpoints. Analyses of forced‐choice‐based NPI‐40 scores in U.S.‐based student samples showed that a model with one joinpoint yielded the best fit compared to a linear and a two‐joinpoint solution. A first regression segment from 1982 to 2017 indicated virtually no effects (*b* = 0.02, *p* = 0.297), followed by decreasing scores from 2017 to 2022 (*b* = −0.91, *p* = 0.018), thus suggesting no meaningful role of the GFC in 2008 for narcissism trajectory changes.

When joinpoint analyses were repeated for all samples and any NPI formats in U.S.‐based or global samples, non‐linear trajectories yielding either one‐ or two‐joinpoint solutions invariably showed better fits than linear solutions. However, none of the solutions indicated evidence for meaningful positive changes up to 2008 and subsequent slope reversals, which would be expected according to the idea of the GFC being responsible for an end of a thus far ongoing Narcissism Epidemic. Numerical details are provided in the Supporting Information at https://osf.io/fcwem/files/osfstorage#.

## 4 Discussion

Here, we show that there remains only little support for a Narcissism Epidemic over the past decades on a global scale. These results broadly generalize across different regions, countries, and sample types. We observed meaningful negative time trends in virtually all trajectories of NPI‐based narcissism self‐reports from 1982 to 2023. This contrasts past observations of increasing NPI scores in U.S. students and ideas about an isolated event (i.e., the Global Financial Crisis) as a cause for a reversal of ongoing positive narcissism trajectories in 2008 (Twenge et al. 2008, 2021). These findings present several points of interest, as we discuss below.

First, our results indicate a meaningful, albeit small, negative time trend in narcissism among U.S. students from 1982 to 2023. These findings are in line with previously reported small declines in narcissism among U.S. students between 1990 and 2010 (*d* = −0.27; Wetzel et al. 2017), thus further calling the idea of a (former) Narcissism Epidemic in the USA into question (Trzesniewski and Donnellan 2010; Wetzel et al. 2017).

A similar pattern was observed for the entirety of all included samples, although negative trends appeared to be stronger in all compared to merely North American or U.S. samples. Of note, mean NPI scores from U.S.‐based and North American samples were significantly larger than those from European students and Asian participants in general. Such narcissism mean differences are consistent with prior evidence (Foster, Campbell, and Twenge 2003). It has been argued that these differences may be attributable to people from more individualistic regions having a higher self‐focus while those from more collectivistic ones have a more pronounced group‐focus (Triandis 2001). Region‐specific narcissism mean scores may be responsible for the more substantial negative cross‐temporal regression slope in all samples globally compared to U.S.‐based or North American samples because the number of includable studies from Asia and Europe has increased over time.

Alternatively, negative cross‐temporal narcissism trajectories have been suggested to represent a consequence of declining negative (e.g., decreasing juvenile crime rates) and increasing positive behaviors of youths (e.g., increases in volunteer work, Pryor et al. 2007; for a discussion, see Arnett 2013). In this vein, it has been argued that over time increasingly more emphasis has been placed in society on values like acceptance or tolerance (e.g., in terms of ethnicity or sexual orientation of others; Zogby 2008). Arguably, such behaviors and attitudes may be expected to contrast the development of narcissism. Consequently, according to this idea, younger generations could be expected to yield lower narcissism values compared to older generations.

Second, although regression slopes in some U.S.‐subsamples were positive before the Global Financial Crisis in 2008, effect strengths were trivial. This supports evidence from archival studies that did not yield any narcissism or self‐enhancement time trends from 1982 to 2007 in U.S. students (Trzesniewski, Donnellan, and Robins 2008) but contrasts prior reports of increases in self‐reported narcissism until the first decade of the 2000s (e.g., Twenge and Foster 2010; Twenge et al. 2008).

Specifically, it has been argued that narcissism has been increasing in the past, with the GFC in 2008 marking a turning point, after which narcissism may have started to decrease (Twenge et al. 2021). However, we found no evidence for such a change that could be convincingly attributed to a single event in 2008 (i.e., the GFC) among U.S. students or participants in general.

However, virtually all regression segments in our (sub‐)analyses that post‐date the 2008 GFC yielded substantial negative effects. In light of the virtual stagnation of narcissism values before the GFC, there may have indeed been a meaningful negative effect of the GFC on population narcissism. This observation may be seen to be in line with the idea of the GFC conceivably having influenced narcissism self‐reports, especially when it comes to U.S. students (see Twenge et al. 2021). In any case, even if the currently observable narcissism decreases were triggered by the GFC, there is no evidence for meaningful narcissism increases before the GFC.

It needs to be acknowledged that some results of our segmented line regressions indicated positive trajectories of the initial regression segments. However, these positive trajectories were small on the whole and joinpoints did not suggest slope changes in 2008, which were to be expected if changes in cross‐temporal trajectories were to be attributed to the GFC.

Third, in contrast to our expectations, our study indicated that in the single case where a meaningful interaction between sex and data collection year was observed, women showed negative narcissism changes while the scores of men were cross‐temporally stable. In prior work, it has been shown that women self‐reported increasingly masculine‐stereotyped personality characteristics such as agentic traits or assertiveness from the 1970s to the mid‐1990s (Twenge 1997, 2001), while self‐reports of men did not show equally substantial increases. It has been argued that similar patterns may be expected for other traits in which men are typically observed to score higher, such as narcissism (Twenge et al. 2008).

Our findings contrast this idea and mean that previously identified positive cross‐temporal narcissism changes (e.g., Twenge et al. 2008) are unlikely to be attributable to a sex‐specific narcissism increase in women. These findings are supported by other studies that did not find a convergence between the narcissism scores of women and men over time (Grijalva et al. 2015; Stronge, Milojev, and Sibley 2018).

Several explanations have been offered to explain cross‐temporal decreases in narcissism. One frequently discussed candidate theory pertains to the influence of social media on personality traits. It has been shown that social networking feeds that often portray embellished images and idealized portrayals of users can have a negative impact on the self‐esteem and well‐being of young adults (e.g., Schmuck et al. 2019; Wang et al. 2017). This negative influence is explained by social upward comparisons, which are promoted by the idealized feeds of social networking sites (Steers, Wickham, and Acitelli 2014). Because upward comparisons are associated with negative feelings toward self‐image (Buunk and Gibbons 2006), constant upward social comparisons on social networking sites have been argued to temper narcissistic tendencies (e.g., Twenge et al. 2021).

Another candidate theory suggests influences of cross‐temporally increasing psychopathology as a potential cause for decreasing narcissism. It has been frequently shown that narcissism is negatively related to depressive and anxiety symptoms (e.g., Raskin and Novacek 1989; Sedikides et al. 2004). Recent studies indicate an increase in depressive symptoms and anxiety disorders among children and adolescents in high‐income countries (e.g., Collishaw 2015; Keyes et al. 2019). Consequently, in these countries, decreases in self‐reported narcissism may conceivably be attributed to depression and anxiety increases.

Finally, cross‐temporal narcissism values appeared to be stable according to the subdomains authority, exhibitionism, self‐sufficiency, and vanity. However, superiority scores significantly decreased while entitlement and exploitativeness scores appeared to increase. However, due to the rather low numbers of studies that reported subscale scores and the uneven distribution of these studies according to data collection year, the meaningfulness of these findings remains uncertain.

### 4.1 Limitations

A meaningful interpretation of cross‐temporal changes depends on measurement invariance of the used test instrument. Presently, it was not possible to examine potential (non‐)invariance because item‐level data were unavailable. However, prior studies have shown that although NPI items appear not to be completely measurement invariant, NPI score interpretability remained largely unaffected (Hamamura, Johnson, and Stankovic 2020; Wetzel et al. 2017). However, we cannot entirely rule out that measurement non‐invariance may have affected change assessments.

## 5 Conclusion

In the present meta‐analysis, we show evidence for negative cross‐temporal changes in narcissism from 1982 to 2023 on a global scale, thus contrasting the idea of a Narcissism Epidemic during the past four decades. Changes generalized across different regions and participant sex, although mean scores were differentiated, yielding higher narcissism values for North American and younger samples. Previously hypothesized effects of the Global Financial Crisis as the cause for an end to a prior Narcissism Epidemic seems questionable in the light of our findings. In all, we show that self‐reported subclinical narcissism appears to have been decreasing over the past 40 years on a global scale.

## Author Contributions

**Sandra Oberleiter:** data curation, formal analysis, investigation, methodology, visualization, software, writing – original draft. **Paul Stickel:** conceptualization, data curation, formal analysis, investigation, methodology, software, writing – original draft. **Jakob Pietschnig:** conceptualization, investigation, validation, methodology, project administration, writing – original draft, supervision.

## Conflicts of Interest

The authors declare no conflicts of interest.

## Supporting information


Data S1.
