# Supplementary material for: A Farewell to the Narcissism Epidemic? A Cross‐Temporal Meta‐Analysis of Global NPI Scores (1982–2023)
Source: J Pers. 2024 Oct 14;93(4):884–94. doi: 10.1111/jopy.12982 (PMC12224556; doi:10.1111/jopy.12982)
Supplement: Supplementary file 1 — Data S1. [file JOPY-93-884-s001.zip › Tables S1 to S43.pdf]

Table 1

Single and multiple linear weighted meta-regression as well as residualized regressions for U.S.-based samples on any NPI scores from 1982 to 2023.

Predictors	Model fit	*k*	*b*	*SE*	η_p^2	*p*
	Single regression					
	$R^2 = .055^{***}$; $F(1, 746) = 44.20^{***}$	747				
Year of data collection (1982-2023)			-0.108	0.016	.056	<.001
	Multiple regression					
	$R^2 = .096^{***}$; $F(3, 547) = 20.69^{***}$	550				
Year of data collection (1982-2023)			-0.062	0.020	.031	.002
Sample mean age			0.114	0.183	.005	<.001
Percentage of women in sample			-0.026	0.007	.029	<.001
	Regression on residualized values					
	$R^2 = .014^{**}$; $F(1, 549) = 9.93^{**}$	550				
Year of data collection[a] (1982-2023)			-0.061	0.020	.016	.002

Note. Variables were weighted based on sample size; all R^2 are adjusted values; k = number of samples; b = unstandardized regression coefficient; SE = standard error of unstandardized coefficient; β = standardized regression coefficient; η_p^2 = partial eta squared; Variance Inflation Factors (VIFs) in multiple Regression were all <1.1.

[a] Adjusted for confounding variables (Percentage of women in sample & sample mean age).

$^{**}p<.01$; $^{***}p<.001$.

Table 2

Single and multiple linear weighted meta-regression as well as residualized regressions for U.S.-based samples on forced-choice-based NPI-40 scores from 1982 to 2023.

Predictors	Model fit	*k*	*b*	*SE*	η_p^2	*p*
	Single regression					
	$R^2 = .007$***; F (1, 467) = 34.20***	468				
Year of data collection (1982-2023)			-0.100	0.017	.068	<.001
	Multiple regression					
	$R^2 = .195$***; F (3, 330) = 27.91***	333				
Year of data collection (1982-2023)			-0.082	0.021	.069	<.001
Sample mean age			0.141	0.021	.106	<.001
Percentage of women in sample			-0.031	0.006	.057	<.001
	Regression on residualized values					
	$R^2 = .034$***; F (1, 332) = 14.47***	333				
Year of data collection[a] (1982-2023)			-0.081	0.021	.042	<.001

Note. Variables were weighted based on sample size; all R^2 are adjusted values; k = number of samples; b = unstandardized regression coefficient; SE = standard error of unstandardized coefficient; β = standardized regression coefficient; η_p^2 = partial eta squared; Variance Inflation Factors (VIFs) in multiple Regression were all <1.1.

[a] Adjusted for confounding variables (Percentage of women in sample & sample mean age).

***p <.001.

Table 3

Single and multiple linear weighted meta-regression as well as residualized regressions for U.S.-based student samples on any NPI scores from 1982 to 2023.

Predictors	Model fit	*k*	*b*	*SE*	η_p^2	*p*
	Single regression					
	R^2 = .031***; *F* (1, 539) = 18.53***	540				
Year of data collection (1982-2023)			-0.071	0.017	.033	<.001
	Multiple regression					
	R^2 = .060***; *F* (3, 371) = 9.03***	374				
Year of data collection (1982-2023)			-0.057	0.02	.027	.010
Sample mean age			0.270	0.111	.018	.016
Percentage of women in sample			-0.024	0.008	.026	.002
	Regression on residualized values					
	R^2 = .025*; *F* (1, 373) = 6.58*	374				
Year of data collection[a] (1982-2023)			-0.056	0.022	.017	.011

Note. Variables were weighted based on sample size; all R^2 are adjusted values; k = number of samples; b = unstandardized regression coefficient; SE = standard error of unstandardized coefficient; β = standardized regression coefficient; η_p^2 = partial eta squared; Variance Inflation Factors (VIFs) in multiple Regression were all <1.1.

[a] Adjusted for confounding variables (Percentage of women in sample & sample mean age).

$^*p<.05$; $^{**}p<.01$; $^{***}p<.001$.

Table 4

Single and multiple linear weighted meta-regression as well as residualized regressions for global samples on any NPI scores from 1982 to 2023.

Predictors	Model fit	k	b	SE	η_p^2	p
	Single regression					
	R^2 = .050***; F(1, 1619) = 86.73***	1620				
Year of data collection (1982-2023)			-0.145	0.016	.050	<.001
	Multiple regression					
	R^2 = .080***; F (3, 1326) = 39.80***	1329				
Year of data collection (1982-2023)			-0.153	0.019	.055	<.001
Sample mean age			-0.06	0.013	.008	<.001
Percentage of women in sample			-0.023	0.004	.022	<.001
	Regression on residualized values					
	R^2 = .042***; F (1, 1328) = 60.08***	1329				
Year of data collection[a] (1982-2023)			-0.148	0.019	.004	<.001

Note. Variables were weighted based on sample size; all R^2 are adjusted values; k = number of samples; b = unstandardized regression coefficient; SE = standard error of unstandardized coefficient; β = standardized regression coefficient; η_p^2 = partial eta squared; Variance Inflation Factors (VIFs) in multiple Regression were all <1.1.

[a] Adjusted for confounding variables (Percentage of women in sample & sample mean age, Scale, Items & Sample).

***p <.001.

Table 5

Single and multiple linear weighted meta-regression as well as residualized regressions for global samples on forced choice-based NPI-40 scores from 1982 to 2023.

Predictors	Model fit	*k*	*b*	*SE*	η_p^2	*p*
	Single regression					
	$R^2 = .116^{***}$; F (1, 812) = 107.8***	813				
Year of data collection (1982-2023)			-0.159	0.015	.117	<.001
	Multiple regression					
	$R^2 = .177^{***}$; F (3, 640) = 47.05***	643				
Year of data collection (1982-2023)			-0.133	0.018	.099	<.001
Sample mean age			-0.113	0.015	.061	<.001
Percentage of women in sample			-0.026	0.005	.044	<.001
	Regression on residualized values					
	$R^2 = .007^{***}$; F (1, 642) = 50.56***	643				
Year of data collection[a] (1982-2023)			-0.131	0.185	.007	<.001

Note. Variables were weighted based on sample size; all R^2 are adjusted values; k = number of samples; b = unstandardized regression coefficient; SE = standard error of unstandardized coefficient; β = standardized regression coefficient; η_p^2 = partial eta squared; Variance Inflation Factors (VIFs) in multiple Regression were all <1.1.

[a] Adjusted for confounding variables (Percentage of women in sample & sample mean age).

***p <.001.

Table 6

Single and multiple linear weighted meta-regression as well as residualized regressions for global student samples on any NPI scores from 1982 to 2023.

Predictors	Model fit	*k*	*b*	*SE*	η_p^2	*p*
	Single regression					
	R^2 = .040***; *F* (1, 866) = 37.47***	867				
Year of data collection (1982-2023)			-0.096	0.016	.041	<.001
	Multiple regression					
	R^2 = .060***; *F* (3, 674) = 15.47***	677				
Year of data collection (1982-2023)			-0.100	0.020	<.001	<.001
Sample mean age			-0.002	0.055	<.001	.969
Percentage of women in sample			-0.026	0.006	<.001	<.001
	Regression on residualized values					
	R^2 = .028***; *F* (1, 676) = 20.15***	677				
Year of data collection[a] (1982-2023)			-0.090	0.020	.029	<.001

Note. Variables were weighted based on sample size; all R^2 are adjusted values; k = number of samples; b = unstandardized regression coefficient; SE = standard error of unstandardized coefficient; β = standardized regression coefficient; η_p^2 = partial eta squared; Variance Inflation Factors (VIFs) in multiple Regression were all <1.1.

[a] Adjusted for confounding variables (Percentage of women in sample & sample mean age).

***p <.001.

Table 7

Single and multiple linear weighted meta-regression as well as residualized regressions for global student samples on forced choice-based NPI-40 scores from 1982 to 2023.

Predictors	Model fit	*k*	*b*	*SE*	η_p^2	*p*
	Single regression					
	R^2 = .038***; *F* (1, 520) = 21.35***	521				
Year of data collection (1982-2023)			-0.072	0.016	.039	<.001
	Multiple regression					
	R^2 = .104***; *F* (3, 387) = 16.14***	390				
Year of data collection (1982-2023)			-0.008	0.021	.050	<.001
Sample mean age			-0.122	0.071	.007	.005
Percentage of women in sample			-0.030	0.006	.061	<.001
	Regression on residualized values					
	R^2 = .032***; *F* (1, 389) = 14.06***	390				
Year of data collection[a] (1982-2023)			-0.078	0.021	.035	<.001

Note. Variables were weighted based on sample size; all R^2 are adjusted values; k = number of samples; b = unstandardized regression coefficient; SE = standard error of unstandardized coefficient; β = standardized regression coefficient; η_p^2 = partial eta squared; Variance Inflation Factors (VIFs) in multiple Regression were all <1.1.

[a] Adjusted for confounding variables (Percentage of women in sample & sample mean age).

***p <.001.

Table 8

Single and multiple linear weighted meta-regression as well as residualized regressions for Asian samples on any NPI scores from 1982 to 2023.

Predictors	Model fit	*k*	*b*	*SE*	η_p^2	*p*
	Single regression					
	R^2 = .086***; *F* (1, 174) = 17.46***	175				
Year of data collection (1982-2023)			-0.291	0.070	.091	<.001
	Multiple regression					
	R^2 = .120***; *F* (3, 136) = 7.263***	140				
Year of data collection (1982-2023)			-0.315	0.079	.114	<.001
Sample mean age			-0.011	0.065	<.001	.871
Percentage of women in sample			-0.019	0.009	.031	.038
	Regression on residualized values					
	R^2 = .093***; *F* (1, 138) = 15.19***	139				
Year of data collection[a] (1982-2023)			-0.294	0.075	.099	<.001

Note. Variables were weighted based on sample size; all R^2 are adjusted values; k = number of samples; b = unstandardized regression coefficient; SE = standard error of unstandardized coefficient; β = standardized regression coefficient; η_p^2 = partial eta squared; Variance Inflation Factors (VIFs) in multiple Regression were all <1.1.

[a] Adjusted for confounding variables (Percentage of women in sample & sample mean age).

***p <.001.

Table 9

Single and multiple linear weighted meta-regression as well as residualized regressions for Asian samples on forced choice-based NPI-40 scores from 1982 to 2023.

Predictors	Model fit	k	b	SE	η_p^2	p
	Single regression					
	R^2 = .017; F (1, 50) = 1.901	51				
Year of data collection (1982-2023)			-0.107	0.078	.036	.174
	Multiple regression					
	R^2 = .121*; F (3, 40) = 2.978*	43				
Year of data collection (1982-2023)			-0.124	0.086	.012	.162
Sample mean age			-0.219	0.078	.155	.008
Percentage of women in sample			-0.013	0.012	.027	.299
	Regression on residualized values					
	R^2 = .022; F (1, 42) = 43.4	43				
Year of data collection[a] (1982-2023)			-0.116	0.082	.045	.167

Note. Variables were weighted based on sample size; all R^2 are adjusted values; k = number of samples; b = unstandardized regression coefficient; SE = standard error of unstandardized coefficient; β = standardized regression coefficient; η_p^2 = partial eta squared; Variance Inflation Factors (VIFs) in multiple Regression were all <1.1.

[a] Adjusted for confounding variables (Percentage of women in sample & sample mean age).

*p <.05.

Table 10

Single and multiple linear weighted meta-regression as well as residualized regressions for Asian student samples on any NPI scores from 1982 to 2023.

Predictors	Model fit	*k*	*b*	*SE*	η_p^2	*p*
	Single regression					
	R^2 = <.001; *F* (1, 67) = 0.045	68				
Year of data collection (1982-2023)			0.019	.087	<.001	.083
	Multiple regression					
	R^2 = .130**; *F* (3, 57) = 3.978**	60				
Year of data collection (1982-2023)			-0.070	0.076	.004	.364
Sample mean age			0.535	0.164	.163	.020
Percentage of women in sample			-0.012	0.016	.012	.429
	Regression on residualized values					
	R^2 = .002; *F* (1, 59) = 0.851	60				
Year of data collection[a] (1982-2023)			-0.069	0.074	.014	.360

Note. Variables were weighted based on sample size; all R^2 are adjusted values; k = number of samples; b = unstandardized regression coefficient; SE = standard error of unstandardized coefficient; β = standardized regression coefficient; η_p^2 = partial eta squared; Variance Inflation Factors (VIFs) in multiple Regression were all <1.1.

[a] Adjusted for confounding variables (Percentage of women in sample & sample mean age).

$^{**}p < .01$.

Table 11

Single and multiple linear weighted meta-regression as well as residualized regressions for Asian student samples on forced-choice-based NPI-40 scores from 1982 to 2023.

Predictors	Model fit	*k*	*b*	*SE*	η_p^2	*p*
	Single regression					
	R^2 = <.001; *F* (1, 23) = 0.986	24				
Year of data collection (1982-2023)			-0.058	.058	.004	.331
	Multiple regression					
	R^2 = .120; *F* (3, 19) = 2.830	22				
Year of data collection (1982-2023)			-0.099	0.061	.030	.124
Sample mean age			0.757	0.299	.276	.020
Percentage of women in sample			-0.011	0.014	.034	.424
	Regression on residualized values					
	R^2 = .065; *F* (1, 21) = 2.53	22				
Year of data collection[a] (1982-2023)			-0.088	0.055	.107	.128

Note. Variables were weighted based on sample size; all R^2 are adjusted values; k = number of samples; b = unstandardized regression coefficient; SE = standard error of unstandardized coefficient; β = standardized regression coefficient; η_p^2 = partial eta squared; Variance Inflation Factors (VIFs) in multiple Regression were all <1.1.

[a] Adjusted for confounding variables (Percentage of women in sample & sample mean age).

Table 12

Single and multiple linear weighted meta-regression as well as residualized regressions for European samples on any NPI scores from 1982 to 2023.

Predictors	Model fit	*k*	*b*	*SE*	η_p^2	*p*
		Single regression				
	R^2 = .037***; *F* (1, 428) = 17.08***	429				
Year of data collection (1982-2023)			-0.213	0.052	.038	<.001
		Multiple regression				
	R^2 = .081***; *F* (3, 387) = 12.49***	390				
Year of data collection (1982-2023)			-0.296	0.570	.068	<.001
Sample mean age			-0.023	0.024	.006	.351
Percentage of women in sample			-0.022	0.009	.017	.009
		Regression on residualized values				
	R^2 = .006***; *F* (1, 389) = 26.78***	390				
Year of data collection[a] (1982-2023)			-0.291	0.056	.0644	<.001

Note. Variables were weighted based on sample size; all R^2 are adjusted values; k = number of samples; b = unstandardized regression coefficient; SE = standard error of unstandardized coefficient; β = standardized regression coefficient; η_p^2 = partial eta squared; Variance Inflation Factors (VIFs) in multiple Regression were all <1.1.

[a] Adjusted for confounding variables (Percentage of women in sample & sample mean age).

***p <.001.

Table 13

Single and multiple linear weighted meta-regression as well as residualized regressions for European samples on forced choice-based NPI-40 scores from 1982 to 2023.

Predictors	Model fit	*k*	*b*	*SE*	η_p^2	*p*
	Single regression					
	$R^2 = .183^{***}$; $F\ (1, 150) = 34.78^{***}$	151				
Year of data collection (1982-2023)			-0.379	0.064	.188	<.001
	Multiple regression					
	$R^2 = .271^{***}$; $F\ (3, 131) = 17.62^{***}$	134				
Year of data collection (1982-2023)			-0.410	0.066	.236	<.001
Sample mean age			-0.057	0.044	.027	.201
Percentage of women in sample			-0.034	0.115	.062	<.001
	Regression on residualized values					
	$R^2 = .022^{***}$; $F\ (1, 133) = 38.68^{***}$	134				
Year of data collection[a] (1982-2023)			-0.409	0.066	.022	<.001

Note. Variables were weighted based on sample size; all R^2 are adjusted values; k = number of samples; b = unstandardized regression coefficient; SE = standard error of unstandardized coefficient; β = standardized regression coefficient; η_p^2 = partial eta squared; Variance Inflation Factors (VIFs) in multiple Regression were all <1.1.

[a] Adjusted for confounding variables (Percentage of women in sample & sample mean age).

***p <.001.

Table 14

Single and multiple linear weighted meta-regression as well as residualized regressions for European student samples on any NPI scores from 1982 to 2023.

Predictors	Model fit	*k*	*b*	*SE*	η_p^2	*p*
	Single regression					
	$R^2 = .037^*$; F (1, 140) = 6.43*	141				
Year of data collection (1982-2023)			-.213	.083	.043	.001
	Multiple regression					
	$R^2 = .069^{**}$; F (3, 128) = 4.24*	131				
Year of data collection (1982-2023)			-0.220	0.088	.050	.013
Sample mean age			-0.085	0.094	.005	.368
Percentage of women in sample			-0.030	0.013	.039	.025
	Regression on residualized values					
	$R^2 = .039^{**}$; F (1, 130) = 6.378**	131				
Year of data collection[a] (1982-2023)			-0.220	0.087	.047	.013

Note. Variables were weighted based on sample size; all R^2 are adjusted values; k = number of samples; b = unstandardized regression coefficient; SE = standard error of unstandardized coefficient; β = standardized regression coefficient; η_p^2 = partial eta squared; Variance Inflation Factors (VIFs) in multiple Regression were all <1.1.

[a] Adjusted for confounding variables (Percentage of women in sample & sample mean age).

*p<.05; **p <.01.

Table 15

Single and multiple linear weighted meta-regression as well as residualized regressions for European student samples on forced-choice-based NPI-40 scores from 1982 to 2023.

Predictors	Model fit	*k*	*b*	*SE*	η_p^2	*p*
	Single regression					
	R^2 = .011; *F* (1, 53) = 1.60	54				
Year of data collection (1982-2023)			-.095	.075	.030	.211
	Multiple regression					
	R^2 = .007; *F* (3, 47) = 1.12	50				
Year of data collection (1982-2023)			-0.106	0.079	.030	.185
Sample mean age			0.011	0.090	.023	.903
Percentage of women in sample			-0.017	0.013	.102	.193
	Regression on residualized values					
	R^2 = .017; *F* (1, 49) = 1.87	50				
Year of data collection[a] (1982-2023)			-0.106	0.077	.037	.177

Note. Variables were weighted based on sample size; all R^2 are adjusted values; k = number of samples; b = unstandardized regression coefficient; SE = standard error of unstandardized coefficient; β = standardized regression coefficient; η_p^2 = partial eta squared; Variance Inflation Factors (VIFs) in multiple Regression were all <1.1.

[a] Adjusted for confounding variables (Percentage of women in sample & sample mean age).

Table 16

Single and multiple linear weighted meta-regression as well as residualized regressions for North American samples on any NPI scores from 1982 to 2023.

Predictors	Model fit	k	b	SE	η_p^2	p
	Single regression					
	R^2 = .060***; F (1, 862) = 55.77***	863				
Year of data collection (1982-2023)			-0.115	0.015	.060	<.001
	Multiple regression					
	R^2 = .099***; F (3, 655) = 25.12***	658				
Year of data collection (1982-2023)			-0.075	0.019	.038	<.001
Sample mean age			-0.106	0.016	.050	<.001
Percentage of women in sample			-0.022	0.006	.022	<.001
	Regression on residualized values					
	R^2 = .020***; F (1, 657) = 14.68***	658				
Year of data collection[a] (1982-2023)			-0.072	0.018	.029	<.001

Note. Variables were weighted based on sample size; all R^2 are adjusted values; k = number of samples; b = unstandardized regression coefficient; SE = standard error of unstandardized coefficient; β = standardized regression coefficient; η_p^2 = partial eta squared; Variance Inflation Factors (VIFs) in multiple Regression were all <1.1.

[a] Adjusted for confounding variables (Percentage of women in sample & sample mean age).

***p <.001.

Table 17

Single and multiple linear weighted meta-regression as well as residualized regressions for North American samples on forced choice-based NPI-40 scores from 1982 to 2023.

Predictors	Model fit	*k*	*b*	*SE*	η_p^2	*p*
		Single regression				
	$R^2 = .070$***; F (1, 541) = 39.72***	542				
Year of data collection (1982-2023)			-0.100	0.016	.069	<.001
		Multiple regression				
	$R^2 = .188$***; F (3, 398) = 30.09***	401				
Year of data collection (1982-2023)			-0.087	0.019	.070	<.001
Sample mean age			-0.119	0.018	.090	<.001
Percentage of women in sample			-0.028	0.006	.052	<.001
		Regression on residualized values				
	$R^2 = .046$***; F (1, 400) = 20.33***	401				
Year of data collection[a] (1982-2023)			-0.086	0.019	.049	<.001

Note. Variables were weighted based on sample size; all R^2 are adjusted values; k = number of samples; b = unstandardized regression coefficient; SE = standard error of unstandardized coefficient; β = standardized regression coefficient; η_p^2 = partial eta squared; Variance Inflation Factors (VIFs) in multiple Regression were all <1.1.

[a] Adjusted for confounding variables (Percentage of women in sample & sample mean age).

***p <.001.

Table 18

Single and multiple linear weighted meta-regression as well as residualized regressions for North American student samples on any NPI scores from 1982 to 2023.

Predictors	Model fit	*k*	*b*	*SE*	η_p^2	*p*
	Single regression					
	R^2 = .038***; *F* (1, 627) = 25.7***	628				
Year of data collection (1982-2023)			-.080	.016	.040	.001
	Multiple regression					
	R^2 = .070***; *F* (3, 455) = 12.52***	458				
Year of data collection (1982-2023)			-0.065	0.020	.034	<.001
Sample mean age			-0.256	0.098	.017	.009
Percentage of women in sample			-0.025	0.007	.029	<.001
	Regression on residualized values					
	R^2 = .020**; *F* (1, 457) = 10.30**	458				
Year of data collection[a] (1982-2023)			-0.064	0.020	.022	.001

Note. Variables were weighted based on sample size; all R^2 are adjusted values; k = number of samples; b = unstandardized regression coefficient; SE = standard error of unstandardized coefficient; β = standardized regression coefficient; η_p^2 = partial eta squared; Variance Inflation Factors (VIFs) in multiple Regression were all <1.1.

[a] Adjusted for confounding variables (Percentage of women in sample & sample mean age).

$^{**}p < .01$; $^{***}p < .001$.

Table 19

Single and multiple linear weighted meta-regression as well as residualized regressions for North American student samples on forced-choice-based NPI-40 scores from 1982 to 2023.

Predictors	Model fit	*k*	*b*	*SE*	η_p^2	*p*
			Single regression			
	R^2 = .0325***; *F* (1, 428) = 11.86***	429				
Year of data collection (1982-2023)			-.057	.017	.028	<.001
			Multiple regression			
	R^2 = .105***; *F* (3, 302) = 13.00***	305				
Year of data collection (1982-2023)			-0.068	0.023	.004	.003
Sample mean age			0.018	0.135	<.001	.892
Percentage of women in sample			-0.035	0.007	.075	<.001
			Regression on residualized values			
	R^2 = .026**; *F* (1, 304) = 9.03**	305				
Year of data collection[a] (1982-2023)			-0.067	0.022	.029	.002

Note. Variables were weighted based on sample size; all R^2 are adjusted values; k = number of samples; b = unstandardized regression coefficient; SE = standard error of unstandardized coefficient; β = standardized regression coefficient; η_p^2 = partial eta squared; Variance Inflation Factors (VIFs) in multiple Regression were all <1.1.

[a] Adjusted for confounding variables (Percentage of women in sample & sample mean age).

$^{**}p < .01$; $^{***}p < .001$.

Table 20

Single and multiple linear weighted meta-regression as well as residualized regressions for Oceanian samples on any NPI scores from 1982 to 2023.

Predictors	Model fit	*k*	*b*	*SE*	η_p^2	*p*
	Single regression					
	R^2 = .052; *F* (1, 52) = 3.872	53				
Year of data collection (1982-2023)			-0.220	0.112	.069	.054
	Multiple regression					
	R^2 = .126*; *F* (3, 47) = 3.41*	50				
Year of data collection (1982-2023)			-0.190	0.109	.076	.087
Sample mean age			-0.227	0.092	.088	.018
Percentage of women in sample			-0.038	0.028	.037	.184
	Regression on residualized values					
	R^2 = .042; *F* (1, 49) = 3.16	50				
Year of data collection[a] (1982-2023)			-0.060	0.106	.061	.082

Note. Variables were weighted based on sample size; all R^2 are adjusted values; k = number of samples; b = unstandardized regression coefficient; SE = standard error of unstandardized coefficient; β = standardized regression coefficient; η_p^2 = partial eta squared; Variance Inflation Factors (VIFs) in multiple Regression were all <1.1.

[a] Adjusted for confounding variables (Percentage of women in sample & sample mean age).

*$p < .05$.

Table 21

Single and multiple linear weighted meta-regression as well as residualized regressions for Oceanian samples on forced choice-based NPI-40 scores from 1982 to 2023.

Predictors	Model fit	*k*	*b*	*SE*	η_p^2	*p*
			Single regression			
	$R^2 = -.051$; $F\ (1, 19) = 0.024$	20				
Year of data collection (1982-2023)			-0.022	0.144	.001	.878
			Multiple regression			
	$R^2 = .375^*$; $F\ (3, 16) = 4.80^*$	19				
Year of data collection (1982-2023)			<0.001	<0.001	.001	.481
Sample mean age			<0.001	<0.001	.047	.003
Percentage of women in sample			<0.001	<0.001	.007	.915
			Regression on residualized values			
	$R^2 = -.024$; $F\ (1, 18) = 0.55$	19				
Year of data collection[a] (1982-2023)			-0.079	0.106	.030	.467

Note. Variables were weighted based on sample size; all R^2 are adjusted values; k = number of samples; b = unstandardized regression coefficient; SE = standard error of unstandardized coefficient; β = standardized regression coefficient; η_p^2 = partial eta squared; Variance Inflation Factors (VIFs) in multiple Regression were all <1.1.

[a] Adjusted for confounding variables (Percentage of women in sample & sample mean age).

$^*p < .05$.

Table 22

Single and multiple linear weighted meta-regression as well as residualized regressions for U.S.-based student samples on forced choice-based NPI-40 scores before the global financial crisis (until 2008).

Predictors	Model fit	*k*	*b*	*SE*	η_p^2	*p*
	Single regression					
	R^2 = <.001; F (1, 183) = 0.002	184				
Year of data collection (1982-2008)			-0.001	0.024	<.001	.965
	Multiple regression					
	R^2 = .130**; F (3, 97) = 5.99**	100				
Year of data collection (1982-2008)			0.047	0.030	.022	.113
Sample mean age			0.038	0.138	.001	.782
Percentage of women in sample			-0.024	0.006	.139	<.001
	Regression on residualized values					
	R^2 = .016; F (1, 99) = 2.61	100				
Year of data collection[a] (1982-2008)			0.047	0.029	.026	.109

Note. Variables were weighted based on sample size; all R^2 are adjusted values; k = number of samples; b = unstandardized regression coefficient; SE = standard error of unstandardized coefficient; β = standardized regression coefficient; η_p^2 = partial eta squared; Variance Inflation Factors (VIFs) in multiple Regression were all <1.1.

[a] Adjusted for confounding variables (Percentage of women in sample & sample mean age).

**p<.01.

Table 23

Single and multiple linear weighted meta-regression as well as residualized regressions for U.S.-based samples any NPI scores before the global financial crisis (until 2008).

Predictors	Model fit	*k*	*b*	*SE*	η_p^2	*p*
	Single regression					
	R^2 = .010*; *F* (1, 284) = 3.98*	285				
Year of data collection (1982-2008)			0.042	0.021	.014	.047
	Multiple regression					
	R^2 = .199***; *F* (3, 162) = 14.68***	165				
Year of data collection (1982-2008)			<0.001	<0.001	.073	<.001
Sample mean age			-<0.001	<0.001	.123	<.001
Percentage of women in sample			-<0.001	<0.001	.048	<.001
	Regression on residualized values					
	R^2 = .086***; *F* (1, 164) = 16.45***	165				
Year of data collection[a] (1982-2008)			0.102	0.025	.091	<.001

Note. Variables were weighted based on sample size; all R^2 are adjusted values; k = number of samples; b = unstandardized regression coefficient; SE = standard error of unstandardized coefficient; β = standardized regression coefficient; η_p^2 = partial eta squared; Variance Inflation Factors (VIFs) in multiple Regression were all <1.1.

[a] Adjusted for confounding variables (Percentage of women in sample & sample mean age).

*$p<.05$; ***$p<.001$.

Table 24

Single and multiple linear weighted meta-regression as well as residualized regressions for U.S.-based samples force-choice-based NPI-40 before the global financial crisis (until 2008).

Predictors	Model fit	*k*	*b*	*SE*	η_p^2	*p*
	Single regression					
	R^2 = -.003; *F* (1, 210) = 0.28	211				
Year of data collection (1982-2008)			-0.012	0.023	.001	.599
	Multiple regression					
	R^2 = .020***; *F* (3, 117) = 11.15***	120				
Year of data collection (1982-2008)			0.050	0.028	.021	.074
Sample mean age			0.097	0.031	.111	.002
Percentage of women in sample			-0.231	0.006	.012	<.001
	Regression on residualized values					
	R^2 = .019; *F* (1, 119) = 3.29	120				
Year of data collection[a] (1982-2008)			0.050	0.028	.027	.072

Note. Variables were weighted based on sample size; all R^2 are adjusted values; k = number of samples; b = unstandardized regression coefficient; SE = standard error of unstandardized coefficient; β = standardized regression coefficient; η_p^2 = partial eta squared; Variance Inflation Factors (VIFs) in multiple Regression were all <1.1.

[a] Adjusted for confounding variables (Percentage of women in sample & sample mean age).

***p <.001.

Table 25

Single and multiple linear weighted meta-regression as well as residualized regressions for U.S.-based student samples on any NPI scores before the global financial crisis (until 2008).

Predictors	Model fit	*k*	*b*	*SE*	η_p^2	*p*
		Single regression				
	$R^2 = .009$; $F\ (1, 251) = 3.83$	252				
Year of data collection (1982-2008)			0.038	0.020	.013	.067
		Multiple regression				
	$R^2 = .152$***; $F\ (3, 137) = 9.35$***	140				
Year of data collection (1982-2008)			<0.001	<0.001	.104	<.001
Sample mean age			-<0.001	<0.001	.011	.196
Percentage of women in sample			-<0.001	<0.001	.071	.001
		Regression on residualized values				
	$R^2 = .109$***; $F\ (1, 139) = 18.05$***	140				
Year of data collection[a] (1982-2008)			0.106	0.025	.115	<.001

Note. Variables were weighted based on sample size; all R^2 are adjusted values; k = number of samples; b = unstandardized regression coefficient; SE = standard error of unstandardized coefficient; β = standardized regression coefficient; η_p^2 = partial eta squared; Variance Inflation Factors (VIFs) in multiple Regression were all <1.1.

[a] Adjusted for confounding variables (Percentage of women in sample & sample mean age).

***p <.001.

Table 26

Single and multiple linear weighted meta-regression as well as residualized regressions for global samples on any NPI scores before the global financial crisis (until 2008).

Predictors	Model fit	*k*	*b*	*SE*	η_p^2	*p*
Single regression						
	R^2 = <.001; F(1, 388) = 0.84	389				
Year of data collection (1982-2008)			0.021	0.023	.002	.360
Multiple regression						
	R^2 = .173***; F (3, 252) = 18.85***	255				
Year of data collection (1982-2008)			<0.001	<0.001	.027	.001
Sample mean age			-<0.001	<0.001	.145	<.001
Percentage of women in sample			-<0.001	<0.001	.026	.009
Regression on residualized values						
	R^2 = .037**; F (1, 254) = 10.93**	255				
Year of data collection[a] (1982-2008)			0.083	0.025	.041	.001

Note. Variables were weighted based on sample size; all R^2 are adjusted values; k = number of samples; b = unstandardized regression coefficient; SE = standard error of unstandardized coefficient; β = standardized regression coefficient; η_p^2 = partial eta squared; Variance Inflation Factors (VIFs) in multiple Regression were all <1.1.

[a] Adjusted for confounding variables (Percentage of women in sample & sample mean age, Scale, Items & Sample).

**p<.01; ***p <.001.

Table 27

Single and multiple linear weighted meta-regression as well as residualized regressions for global samples on forced choice-based NPI-40 scores before the global financial crisis (until 2008).

Predictors	Model fit	*k*	*b*	*SE*	η_p^2	*p*
	Single regression					
	R^2 = -.002; *F* (1, 276) = 0.38	277				
Year of data collection (1982-2008)			0.015	0.024	.001	.540
	Multiple regression					
	R^2 = .192***; *F* (3, 174) = 15.10***	177				
Year of data collection (1982-2008)			0.055	0.028	.019	.053
Sample mean age			-0.132	0.025	.015	<.001
Percentage of women in sample			-0.019	0.006	.061	<.001
	Regression on residualized values					
	R^2 = .016; *F* (1, 176) = 3.82	177				
Year of data collection[a] (1982-2008)			0.055	0.028	.021	.052

Note. Variables were weighted based on sample size; all R^2 are adjusted values; k = number of samples; b = unstandardized regression coefficient; SE = standard error of unstandardized coefficient; β = standardized regression coefficient; η_p^2 = partial eta squared; Variance Inflation Factors (VIFs) in multiple Regression were all <1.1.

[a] Adjusted for confounding variables (Percentage of women in sample & sample mean age).

***p <.001.

Table 28

Single and multiple linear weighted meta-regression as well as residualized regressions for global student samples on any NPI scores before the global financial crisis (until 2008).

Predictors	Model fit	*k*	*b*	*SE*	η_p^2	*p*
			Single regression			
	R^2 = .012*; *F* (1, 305) = 4.61*	306				
Year of data collection (1982-2008)			.043	0.020	.015	.033
			Multiple regression			
	R^2 = .118***; *F* (3, 191) = 9.62***	194				
Year of data collection (1982-2008)			<0.001	<0.001	.075	<.001
Sample mean age			-<0.001	<0.001	.008	.217
Percentage of women in sample			-<0.001	<0.001	.058	<.001
			Regression on residualized values			
	R^2 = .078***; *F* (1, 193) = 17.50***	194				
Year of data collection[a] (1982-2008)			0.100	0.023	.083	<.001

Note. Variables were weighted based on sample size; all R^2 are adjusted values; k = number of samples; b = unstandardized regression coefficient; SE = standard error of unstandardized coefficient; β = standardized regression coefficient; η_p^2 = partial eta squared; Variance Inflation Factors (VIFs) in multiple Regression were all <1.1.

[a] Adjusted for confounding variables (Percentage of women in sample & sample mean age).

*p<.05; ***p <.001.

Table 29

Single and multiple linear weighted meta-regression as well as residualized regressions for global student samples on forced choice-based NPI-40 scores before the global financial crisis (until 2008).

Predictors	Model fit	*k*	*b*	*SE*	η_p^2	*p*
	Single regression					
	$R^2 = -.003$; $F\ (1, 219) = 0.36$	220				
Year of data collection (1982-2008)			0.013	0.022	.002	.552
	Multiple regression					
	$R^2 = .147$; $F\ (3, 133) = 8.80$***	136				
Year of data collection (1982-2008)			0.057	0.026	.033	.032
Sample mean age			-0.151	0.103	.020	.143
Percentage of women in sample			-0.024	0.006	.125	<.001
	Regression on residualized values					
	$R^2 = .027$*; $F\ (1, 135) = 4.76$*	136				
Year of data collection[a] (1982-2008)			0.057	0.026	.034	.030

Note. Variables were weighted based on sample size; all R^2 are adjusted values; k = number of samples; b = unstandardized regression coefficient; SE = standard error of unstandardized coefficient; β = standardized regression coefficient; η_p^2 = partial eta squared; Variance Inflation Factors (VIFs) in multiple Regression were all <1.1.

[a] Adjusted for confounding variables (Percentage of women in sample & sample mean age).

*p<.05; ***p <.001.

Table 30

Single and multiple linear weighted meta-regression as well as residualized regressions for U.S.-based student samples on forced choice-based NPI-40 scores after the global financial crisis (since 2008).

Predictors	Model fit	*k*	*b*	*SE*	η_p^2	*p*
	Single regression					
	R^2 = .048***; *F* (1, 206) = 11.33***	207				
Year of data collection (2008-2023)			-0.180	0.053	.052	<.001
	Multiple regression					
	R^2 = .116***; *F* (3, 164) = 8.29***	167				
Year of data collection (2008-2023)			-0.158	0.061	.061	.010
Sample mean age			0.179	0.224	.008	.423
Percentage of women in sample			-0.045	0.013	.073	<.001
	Regression on residualized values					
	R^2 = .030*; *F* (1, 166) = 6.13*	167				
Year of data collection[a] (2008-2023)			-0.143	0.058	.036	.014

Note. Variables were weighted based on sample size; all R^2 are adjusted values; k = number of samples; b = unstandardized regression coefficient; SE = standard error of unstandardized coefficient; β = standardized regression coefficient; η_p^2 = partial eta squared; Variance Inflation Factors (VIFs) in multiple Regression were all <1.1.

[a] Adjusted for confounding variables (Percentage of women in sample & sample mean age).

$^{*}p<.05$; $^{***}p<.001$.

Table 31

Single and multiple linear weighted meta-regression as well as residualized regressions for U.S.-based samples on any NPI scores after the global financial crisis (since 2008).

Predictors	Model fit	*k*	*b*	*SE*	η_p^2	*p*
	Single regression					
	$R^2 = .050$***; $F\,(1, 493) = 27.04$***	494				
Year of data collection (2008-2023)			-0.237	0.045	.052	<.001
	Multiple regression					
	$R^2 = .088$***; $F\,(3, 408) = 14.28$***	411				
Year of data collection (2008-2023)			-0.104	0.046	.026	.024
Sample mean age			-0.113	0.022	.041	<.001
Percentage of women in sample			-0.034	0.009	.035	<.001
	Regression on residualized values					
	$R^2 = .009$*; $F\,(1, 410) = 5.00$*	411				
Year of data collection[a] (2008-2023)			-0.101	0.045	.012	.026

Note. Variables were weighted based on sample size; all R^2 are adjusted values; k = number of samples; b = unstandardized regression coefficient; SE = standard error of unstandardized coefficient; β = standardized regression coefficient; η_p^2 = partial eta squared; Variance Inflation Factors (VIFs) in multiple Regression were all <1.1.

[a] Adjusted for confounding variables (Percentage of women in sample & sample mean age).

*p<.05; ***p <.001.

.

Table 32

Single and multiple linear weighted meta-regression as well as residualized regressions for U.S.-based samples on force-choice-based NPI-40 after the global financial crisis (since 2008).

Predictors	Model fit	*k*	*b*	*SE*	η_p^2	*p*
	Single regression					
	R^2 = .079***; *F* (1, 279) = 24.94***	280				
Year of data collection (2008-2023)			-0.251	0.050	.082	<.001
	Multiple regression					
	R^2 = .019***; *F* (3, 230) = 19.54***	233				
Year of data collection (2008-2023)			-0.136	0.050	.064	.007
Sample mean age			-0.152	0.026	.101	<.001
Percentage of women in sample			-0.042	0.010	.069	<.001
	Regression on residualized values					
	R^2 = .026**; *F* (1, 232) = 7.12**	233				
Year of data collection[a] (2008-2023)			-0.130	0.048	.030	.008

Note. Variables were weighted based on sample size; all R^2 are adjusted values; k = number of samples; b = unstandardized regression coefficient; SE = standard error of unstandardized coefficient; β = standardized regression coefficient; η_p^2 = partial eta squared; Variance Inflation Factors (VIFs) in multiple Regression were all <1.1.

[a] Adjusted for confounding variables (Percentage of women in sample & sample mean age).

**p<.01; ***p <.001.

Table 33

Single and multiple linear weighted meta-regression as well as residualized regressions for U.S.-based student samples on any NPI scores after the global financial crisis (since 2008).

Predictors	Model fit	*k*	*b*	*SE*	η_p^2	*p*
	Single regression					
	$R^2 = .055^{***}$; $F\,(1, 317) = 19.59^{***}$	318				
Year of data collection (2008-2023)			-0.222	0.050	.059	<.001
	Multiple regression					
	$R^2 = .095^{***}$; $F\,(3, 255) = 10.05^{***}$	258				
Year of data collection (2008-2023)			-0.162	0.052	.038	.002
Sample mean age			0.546	0.156	.052	<.001
Percentage of women in sample			-0.028	0.011	.023	.014
	Regression on residualized values					
	$R^2 = .030^{**}$; $F\,(1, 257) = 9.08^{**}$	258				
Year of data collection[a] (2008-2023)			-0.154	0.051	.034	.003

Note. Variables were weighted based on sample size; all R^2 are adjusted values; k = number of samples; b = unstandardized regression coefficient; SE = standard error of unstandardized coefficient; β = standardized regression coefficient; η_p^2 = partial eta squared; Variance Inflation Factors (VIFs) in multiple Regression were all <1.1.

[a] Adjusted for confounding variables (Percentage of women in sample & sample mean age).

** p <.01; ***p <.001.

Table 34

Single and multiple linear weighted meta-regression as well as residualized regressions for global samples on any NPI scores after the global financial crisis (since 2008).

Predictors	Model fit	*k*	*b*	*SE*	η_p^2	*p*
			Single regression			
	R^2 = .089***; *F*(1, 1295) = 127.90***	1296				
Year of data collection (2008-2023)			-0.347	0.031	.090	<.001
			Multiple regression			
	R^2 = .114***; *F* (3, 1130) = 49.75***	1133				
Year of data collection (2008-2023)			-0.322	0.032	.094	<.001
Sample mean age			-0.044	0.014	.004	.001
Percentage of women in sample			-0.024	0.005	.024	<.001
			Regression on residualized values			
	R^2 = .081***; *F* (1, 1132) = 101.20***	1133				
Year of data collection[a] (2008-2023)			-0.316	0.031	.082	<.001

Note. Variables were weighted based on sample size; all R^2 are adjusted values; k = number of samples; b = unstandardized regression coefficient; SE = standard error of unstandardized coefficient; β = standardized regression coefficient; η_p^2 = partial eta squared; Variance Inflation Factors (VIFs) in multiple Regression were all <1.1.

[a] Adjusted for confounding variables (Percentage of women in sample & sample mean age, Scale, Items & Sample).

$^{***}p < .001$.

Table 35

Single and multiple linear weighted meta-regression as well as residualized regressions for global samples on forced choice-based NPI-40 scores after the global financial crisis (since 2008).

Predictors	Model fit	*k*	*b*	*SE*	η_p^2	*p*
	Single regression					
	R^2 = .013***; *F* (1, 582) = 87.33***	583				
Year of data collection (2008-2023)			-0.343	0.037	.130	<.001
	Multiple regression					
	R^2 = .173***; *F* (3, 507) = 36.56***	510				
Year of data collection (2008-2023)			-0.243	0.037	.102	<.001
Sample mean age			-0.109	0.018	.051	<.001
Percentage of women in sample			-0.029	0.006	.046	<.001
	Regression on residualized values					
	R^2 = .075***; *F* (1, 509) = 42.66***	510				
Year of data collection[a] (2008-2023)			-0.238	0.036	.077	<.001

Note. Variables were weighted based on sample size; all R^2 are adjusted values; k = number of samples; b = unstandardized regression coefficient; SE = standard error of unstandardized coefficient; β = standardized regression coefficient; η_p^2 = partial eta squared; Variance Inflation Factors (VIFs) in multiple Regression were all <1.1.

[a] Adjusted for confounding variables (Percentage of women in sample & sample mean age).

***p <.001.

Table 36

Single and multiple linear weighted meta-regression as well as residualized regressions for global student samples on any NPI scores after the global financial crisis (since 2008).

Predictors	Model fit	*k*	*b*	*SE*	η_p^2	*p*
	Single regression					
	$R^2 = .037^{***}$; $F\,(1, 610) = 24.29^{***}$	611				
Year of data collection (2008-2023)			-.202	0.041	.038	<.001
	Multiple regression					
	$R^2 = .056^{***}$; $F\,(3, 524) = 11.48^{***}$	527				
Year of data collection (2008-2023)			-0.166	0.044	.033	<.001
Sample mean age			0.035	0.063	<.001	.577
Percentage of women in sample			-0.029	0.007	.030	<.001
	Regression on residualized values					
	$R^2 = .024^{***}$; $F\,(1, 526) = 13.96^{***}$	527				
Year of data collection[a] (2008-2023)			-0.161	0.043	.026	<.001

Note. Variables were weighted based on sample size; all R^2 are adjusted values; k = number of samples; b = unstandardized regression coefficient; SE = standard error of unstandardized coefficient; β = standardized regression coefficient; η_p^2 = partial eta squared; Variance Inflation Factors (VIFs) in multiple Regression were all <1.1.

[a] Adjusted for confounding variables (Percentage of women in sample & sample mean age).

***p <.001.

Table 37

Single and multiple linear weighted meta-regression as well as residualized regressions for global student samples on forced choice-based NPI-40 scores after the global financial crisis (since 2008).

Predictors	Model fit	*k*	*b*	*SE*	η_p^2	*p*
	Single regression					
	$R^2 = .052$***; F (1, 336) = 19.48***	337				
Year of data collection (2008-2023)			-0.188	0.043	.055	<.001
	Multiple regression					
	$R^2 = .110$***; F (3, 284) = 12.77***	287				
Year of data collection (2008-2023)			-0.164	0.046	.060	<.001
Sample mean age			-0.072	0.085	.002	.396
Percentage of women in sample			-0.035	0.008	.061	<.001
	Regression on residualized values					
	$R^2 = .370$***; F (1, 286) = 12.04***	287				
Year of data collection[a] (2008-2023)			-0.158	0.046	.040	<.001

Note. Variables were weighted based on sample size; all R^2 are adjusted values; k = number of samples; b = unstandardized regression coefficient; SE = standard error of unstandardized coefficient; β = standardized regression coefficient; η_p^2 = partial eta squared; Variance Inflation Factors (VIFs) in multiple Regression were all <1.1.

[a] Adjusted for confounding variables (Percentage of women in sample & sample mean age).

***p <.001.

Table 38

Multiple linear weighted meta-regression on the effects of study inclusion in Twenge et al. (2008) and data collection year for U.S.-based student samples on forced choice-based NPI-40 scores from 1982 to 2023.

Predictors	Model fit	*k*	*b*	*SE*	η_p^2	*p*
	R^2 = .031***; *F* (3, 367) = 4.94***	370				
Year of data collection (1982-2023)			-0.083	0.024	.023	< .001
Included in Twenge et al. (2008)			-296.612	123.206	< .001	.218
Year of data collection (1982-2023) * Included in Twenge et al. (2008)			0.148	0.061	.016	.017

Note. Variables were weighted based on sample size; all R^2 are adjusted values; *k* = number of samples; *b* = unstandardized regression coefficient; *SE* = standard error of unstandardized coefficient; β = standardized regression coefficient; η_p^2 = partial eta squared; Variance Inflation Factors (VIFs) in multiple Regression were all <1.1.

***p <.001.

Table 39

Multiple linear weighted meta-regression on the effects of study inclusion in Twenge et al. (2008) and data collection year for global samples on any NPI scores from 1982 to 2023.

Predictors	Model fit	*k*	*b*	*SE*	η_p^2	*p*
	R^2 = .054***; *F* (3, 11617) = 31.66***	11620				
Year of data collection (1982-2023)			-0.166	0.018	.051	< .001
Included in Twenge et al. (2008)			-416.909	181.663	.002	.199
Year of data collection (1982-2023) * Included in Twenge et al. (2008)			0.208	0.091	.003	.022

Note. Variables were weighted based on sample size; all R^2 are adjusted values; *k* = number of samples; *b* = unstandardized regression coefficient; *SE* = standard error of unstandardized coefficient; β = standardized regression coefficient; η_p^2 = partial eta squared; Variance Inflation Factors (VIFs) in multiple Regression were all <1.1.

****p* <.001.

Table 40

Multiple linear weighted meta-regression on the effects of age and data collection year for U.S.-based student samples on forced choice-based NPI-40 scores from 1982 to 2023.

Predictors	Model fit	*k*	*b*	*SE*	η_p^2	*p*
	R^2 = .044**; *F* (3, 249) = 3.81**	252				
Year of data collection (1982-2023)			-0.232	0.471	.042	.001
Sample mean age			-14.533	46.391	.001	.731
Year of data collection (1982-2023) * Sample mean age			0.007	0.023	.001	.753

Note. Variables were weighted based on sample size; all R^2 are adjusted values; *k* = number of samples; *b* = unstandardized regression coefficient; *SE* = standard error of unstandardized coefficient; β = standardized regression coefficient; η_p^2 = partial eta squared; Variance Inflation Factors (VIFs) in multiple Regression were all <1.1.

**p <.01.

Table 41

Multiple linear weighted meta-regression on the effects of age and data collection year for global samples on any NPI scores from 1982 to 2023.

Predictors	Model fit	*k*	*b*	*SE*	η_p^2	*p*
	R^2 = .059***; *F* (3, 1340) = 27.9***	1343				
Year of data collection (1982-2023)			-0.216	0.066	.052	<.001
Sample mean age			-5.048	5.112	.006	<.001
Year of data collection (1982-2023) * Sample mean age			0.002	0.003	.001	.327

Note. Variables were weighted based on sample size; all R^2 are adjusted values; *k* = number of samples; *b* = unstandardized regression coefficient; *SE* = standard error of unstandardized coefficient; β = standardized regression coefficient; η_p^2 = partial eta squared; Variance Inflation Factors (VIFs) in multiple Regression were all <1.1.

****p* <.001.

Table 42

Multiple linear weighted meta-regression on the effects of NPI-type (forced-choice NPI-40 vs. else) and data collection year on global NPI scores from 1982 to 2023.

Predictors	Model fit	*k*	*b*	*SE*	η_p^2	*p*
	$R^2 = 0.066$***; F (3, 1617) = 38.94***	1620				
Year of data collection (1982-2023)			-0.173	0.024	.052	<.001
NPI type			-28.739	64.833	.017	<.001
Year of data collection (1982-2023) * NPI type			0.014	0.032	.001	.670

Note. Variables were weighted based on sample size; all R^2 are adjusted values; *k* = number of samples; *b* = unstandardized regression coefficient; *SE* = standard error of unstandardized coefficient; β = standardized regression coefficient; η_p^2 = partial eta squared; Variance Inflation Factors (VIFs) in multiple Regression were all <1.1.

***p <.001.

Table 43

Multiple linear weighted meta-regression on the effects of sample type (student samples vs. else) and data collection year for global samples on any NPI scores from 1982 to 2023.

Predictors	Model fit	*k*	*b*	*SE*	η_p^2	*p*
	$R^2 = 0.058$***; F (3, 1617) = 34.13***	1620				
Year of data collection (1982-2023)			-0.223	0.027	.052	<.001
Sample type			-256.267	68.278	.017	.994
Year of data collection (1982-2023) * Sample type			0.127	0.035	.001	<.001

Note. Variables were weighted based on sample size; all R^2 are adjusted values; *k* = number of samples; *b* = unstandardized regression coefficient; *SE* = standard error of unstandardized coefficient; β = standardized regression coefficient; η_p^2 = partial eta squared; Variance Inflation Factors (VIFs) in multiple Regression were all <1.1.

***p <.001.
