# Supplementary material for: A Farewell to the Narcissism Epidemic? A Cross‐Temporal Meta‐Analysis of Global NPI Scores (1982–2023)
Source: J Pers. 2024 Oct 14;93(4):884–94. doi: 10.1111/jopy.12982 (PMC12224556; doi:10.1111/jopy.12982)
Supplement: Supplementary file 1 — Data S1. [file JOPY-93-884-s001.zip › Deviations from preregistration.docx]

**Deviations from pre-registration**

A farewell to the narcissism epidemic? A cross-temporal meta-analysis of global NPI scores (1982 – 2023)
Sandra Oberleiter*, Paul Stickel*, & Jakob Pietschnig

- Hypotheses 2 and 5 were split into hypotheses predicting an increase in narcissism up to 2008 and hypotheses predicting a decrease in narcissism since 2009. This change was made because previously 2 hypotheses were included in one hypothesis.
- The pre-registered hypothesis, "Differences in NPI mean values between the sexes are smaller in 2021 than in 1992", was changed to an interaction hypothesis: "There will be an interaction between sex and year of data collection with women showing significantly larger increases than men". This change in wording was made because, statistically, an interaction is investigated, not absolute differences.
- The inclusion of studies with an average age of at least 18 years represents a change from pre-registration. This was changed because, in some student samples, 17-year-olds are occasionally included. Primarily in order not to lose this data, the inclusion criterion was changed so that not every individual had to be at least 18 years old, but the mean age of the sample had to be at least 18 years. This was also the approach adopted in the studies by Twenge et al. (2008) and Hamamura et al. (2020).
- The simultaneous inclusion of all other predictors represents a change from preregistration. A block-wise inclusion of the predictors was registered. It was decided not to do so for two reasons. First, the explained variance of individual predictors is not the focus of these analyses, and second, the most relevant analyses (student samples that were collected databased on the NPI-40 with dichotomous response format) contain only the predictor's age and sex ratio, which would have been included in the first block after preregistration. In addition, the published variable was not included as a further predictor since only a small fraction (60 of 1,105 samples in total) of all included samples were not published.
- To assess a possible non-linearity of change trajectories, we assumed a maximum number of two joinpoints instead of one.
