# Supplementary material for: A Farewell to the Narcissism Epidemic? A Cross‐Temporal Meta‐Analysis of Global NPI Scores (1982–2023)
Source: J Pers. 2024 Oct 14;93(4):884–94. doi: 10.1111/jopy.12982 (PMC12224556; doi:10.1111/jopy.12982)
Supplement: Supplementary file 1 — Data S1. [file JOPY-93-884-s001.zip › Included studies.docx]

**Included Articles in the Study:**

Abe, S., & Ota, J. (2023). The Effects of Narcissism and Humor Coping on Anger Expression in Married Couples. *Japanese Psychological Research*, *65*(1), 1-8. <https://doi.org/10.1111/jpr.12344>

Abeyta, A. A., Routledge, C., & Sedikides, C. (2017). Material Meaning: Narcissists Gain Existential Benefits From Extrinsic Goals. *Social Psychological and Personality Science, 8*(2), 219-228. doi:10.1177/1948550616667618

Ackerman, R. A., Donnellan, M. B., & Robins, R. W. (2012). An item response theory analysis of the narcissistic personality inventory. *Journal of Personality Assessment, 94*(2), 141-155. doi:10.1080/00223891.2011.645934

Ackerman, R. A., Witt, E. A., Donnellan, M. B., Trzesniewski, K. H., Robins, R. W., & Kashy, D. A. (2011). What does the Narcissistic personality inventory really measure? *Assessment, 18*(1), 67-87. doi:10.1177/1073191110382845

Adams, J. M., Florell, D., Alex Burton, K., & Hart, W. (2014). Why do narcissists disregard social-etiquette norms? A test of two explanations for why narcissism relates to offensive-language use. *Personality and Individual Differences, 58*, 26-30. doi:10.1016/j.paid.2013.09.027

Adams, J. M., Hart, W., & Alex Burton, K. (2015). I only like the idea of you: Narcissists tolerate others' narcissistic traits but not their corresponding behaviors. *Personality and Individual Differences, 82*, 232-236. doi:10.1016/j.paid.2015.02.019

Aghaz, A., Atashgah, M. S. S., & Zoghipour, M. (2014). Narcissism and counterproductive workplace behaviors among Iranian managers and nonmanagerial employees. *Asian Journal of Business Ethics, 3*(2), 155-169. doi:10.1007/s13520-014-0039-2

Ahadzadeh, A. S., Ong, F. S., & Wu, S. L. (2021). Social media skepticism and belief in conspiracy theories about COVID-19: the moderating role of the dark triad. *Current Psychology*, *42*(11), 8874-8886. <https://doi.org/10.1007/s12144-021-02198-1>

Ahmad, R., Ishaq, M. I., & Raza, A. (2023). Impression management as a friend or foe? Testing mediating role of narcissism and moderating role of ingratiation. *Current Psychology*, 12. <https://doi.org/10.1007/s12144-023-04628-8>

Ahmadi, V., Ahmadi, S., Honarmand, M. M., Zargar, Y., Arshadi, N., & Mirshekar, S. (2013). The Relationships Between Attachment Styles and Narcissism Among Students of Shahid Chamran University in Iran. In H. Uzunboylu & M. Demirok (Eds.), *3RD WORLD CONFERENCE ON PSYCHOLOGY, COUNSELING AND GUIDANCE, WCPCG-2012* (Vol. 84, pp. 215-218).

Akehurst, S., & Thatcher, J. (2010). Narcissism, social anxiety and self-presentation in exercise. *Personality and Individual Differences, 49*(2), 130-135. doi:10.1016/j.paid.2010.03.021

Akhtar, N., Francis, L. J., McKenna, U., & Hasan, S. S. (2023). Introducing the Shorter Dark Tetrad for Muslim Societies (SD4-MS): a study among young adults in Pakistan. *Mental Health Religion & Culture*, *26*(6), 539-549. <https://doi.org/10.1080/13674676.2022.2029380>

Alba, B., & Haslam, N. (2015). Dog people and cat people differ on dominance-related traits. *Anthrozoos, 28*(1), 37-44. doi:10.2752/089279315X14129350721858

Alexander, M. B., Gore, J., & Estep, C. (2021). How Need for Power Explains Why Narcissists Are Antisocial. *Psychological Reports, 124*(3), 1335-1352. doi:10.1177/0033294120926668

Alsawalqa, R. O. (2020). Emotional labour, social intelligence, and narcissism among physicians in Jordan. *Humanities and Social Sciences Communications, 7*(1). doi:10.1057/s41599-020-00666-w

Altgelt, E. E., Reyes, M. A., French, J. E., Meltzer, A. L., & McNulty, J. K. (2018). Who is sexually faithful? Own and partner personality traits as predictors of infidelity. *Journal of Social and Personal Relationships, 35*(4), 600-614. doi:10.1177/0265407517743085

Alves, C. F., & Guedes, M. J. (2022). Narcissistic leaders do not share! The relationship between top managers' narcissism and the distribution of value added. *Finance Research Letters*, *49*, Article 103054. <https://doi.org/10.1016/j.frl.2022.103054>

Amad, S., Gray, N. S., & Snowden, R. J. (2020). Self-Esteem, Narcissism, and Aggression: Different Types of Self-Esteem Predict Different Types of Aggression. *Journal of Interpersonal Violence*. doi:10.1177/0886260520905540

Ames, D. R., Rose, P., & Anderson, C. P. (2006). The NPI-16 as a short measure of narcissism. *Journal of Research in Personality, 40*(4), 440-450. doi:10.1016/j.jrp.2005.03.002

Anderson, C., Ames, D. R., & Gosling, S. D. (2008). Punishing hubris: The perils of overestimating one's status in a group. *Personality and Social Psychology Bulletin, 34*(1), 90-101. doi:10.1177/0146167207307489

Anderson, C., Hildreth, J. A. D., & Sharps, D. L. (2020). The Possession of High Status Strengthens the Status Motive. *Personality and Social Psychology Bulletin, 46*(12), 1712-1723. doi:10.1177/0146167220937544

Andreassen, C. S., Pallesen, S., & Griffiths, M. D. (2017). The relationship between addictive use of social media, narcissism, and self-esteem: Findings from a large national survey. *Addictive Behaviors, 64*, 287-293. doi:10.1016/j.addbeh.2016.03.006

Andreassen, C. S., Ursin, H., Eriksen, H. R., & Pallesen, S. (2012). The relationship of narcissism with workaholism, work engagement, and professional position. *Social Behavior and Personality, 40*(6), 881-890. doi:10.2224/sbp.2012.40.6.881

Anello, K., Lannin, D. G., & Hermann, A. D. (2019). The values of narcissistic grandiosity and vulnerability. *Personality and Individual Differences, 150*. doi:10.1016/j.paid.2019.06.021

Anninos, L. N., Chytiri, A. P., & Chytiris, L. (2019). The magnificent “I” in business education: evidence from Greece. *Journal of Applied Research in Higher Education, 12*(2), 250-270. doi:10.1108/JARHE-02-2019-0031

Antes, A. L., Brown, R. P., Murphy, S. T., Waples, E. P., Mumford, M. D., Connelly, S., & Devenport, L. D. (2007). Personality and ethical decision-making in research: The role of perceptions of self and others. *Journal of Empirical Research on Human Research Ethics, 2*(4), 15-34. doi:10.1525/jer.2007.2.4.15

Appel, M., Slater, M. D., & Oliver, M. B. (2019). Repelled by virtue? The dark triad and eudaimonic narratives. *Media Psychology, 22*(5), 769-794. doi:10.1080/15213269.2018.1523014

Arble, E., & Barnett, D. (2017). An Analysis of Self: The Development and Assessment of a Measure of Selfobject Needs. *Journal of Personality Assessment, 99*(6), 608-618. doi:10.1080/00223891.2016.1278379

Arnocky, S., Piché, T., Albert, G., Ouellette, D., & Barclay, P. (2017). Altruism predicts mating success in humans. *British Journal of Psychology, 108*(2), 416-435. doi:10.1111/bjop.12208

Ashe, D. D., Maltby, J., & McCutcheon, L. E. (2005). Are celebrity-worshippers more prone to narcissism? A brief report. *North American Journal of Psychology, 7*(2), 239-246. Retrieved from https://www.scopus.com/inward/record.uri?eid=2-s2.0-33644614192&partnerID=40&md5=5b5e7c6543f4d86b64151cacc051427a

Aşkun, D., & Çetin, F. (2017a). How Do We Demonstrate Oneness as a Behavior? Operationalizing Oneness Through Scale Measurement. *Journal of Spirituality in Mental Health, 19*(1), 34-60. doi:10.1080/19349637.2016.1184998

Aşkun, D., & Çetin, F. (2017b). Turkish Version of Self-Reflection and Insight Scale: A Preliminary Study for Validity and Reliability of the Constructs. *Psychological Studies, 62*(1), 21-34. doi:10.1007/s12646-017-0390-1

Asrar-ul-Haq, M., & Anjum, T. (2020). Impact of narcissistic leadership on employee work outcomes in banking sector of Pakistan. *Future Business Journal, 6*(1). doi:10.1186/s43093-020-00040-x

Auerbach, J. S. (1984). Validation of Two Scales for Narcissistic Personality Disorder. *Journal of Personality Assessment, 48*(6), 649-653. doi:10.1207/s15327752jpa4806_13

Austin, E. J., Saklofske, D. H., Smith, M., & Tohver, G. (2014). Associations of the managing the emotions of others (MEOS) scale with personality, the Dark Triad and trait EI. *Personality and Individual Differences, 65*, 8-13. doi:10.1016/j.paid.2014.01.060

Aytaç, M. B., & Akın, E. (2020). Hatzfeld syndrome: Narcissistic postpurchase state of mind. *Canadian Journal of Administrative Sciences*. doi:10.1002/cjas.1597

Bachrach, D. G., Kim, K. Y., Patel, P. C., & Harms, P. D. (2023). Birds of a feather?: Firm sales growth and narcissism in the upper echelons at the CEO-TMT interface. *Leadership Quarterly*, *34*(2), 16. <https://doi.org/10.1016/j.leaqua.2022.101621>

Back, M. D., Schmukle, S. C., & Egloff, B. (2010). Why Are Narcissists so Charming at First Sight? Decoding the Narcissism-Popularity Link at Zero Acquaintance. *Journal of Personality and Social Psychology, 98*(1), 132-145. doi:10.1037/a0016338

Baggette, W., & Tobacyk, J. (1988). MENSA MEMBERSHIP AND NARCISSISM. *Psychological Reports, 62*(2), 434-434. doi:10.2466/pr0.1988.62.2.434

Baggio, S., Iglesias, K., Duarte, M., Nicastro, R., Hasler, R., Euler, S., Debbané, M., Starcevic, V., & Perroud, N. (2022). Validation of self-report measures of narcissism against a diagnostic interview. *Plos One*, *17*(4), e0266540. <https://doi.org/10.1371/journal.pone.0266540>

Bailey, C. D. (2019). The joint effects of narcissism and psychopathy on accounting students’ attitudes towards unethical professional practices. *Journal of Accounting Education, 49*. doi:10.1016/j.jaccedu.2019.08.001

Bak, W., & Kutnik, J. (2021). Domains of intellectual humility: Self-esteem and narcissism as independent predictors. *Personality and Individual Differences, 177*. doi:10.1016/j.paid.2021.110815

Balaji, V., & Balasundaram, I. (2015). A study on sub-clinical narcissistic personality score and its relationship with academic performance-an Indian experience. *Asian Social Science, 11*(2), 96-102. doi:10.5539/ass.v11n2p96

Balakrishnan, A., Plouffe, R. A., & Saklofske, D. H. (2017). What do sadists value? Is honesty-humility an intermediary? Replicating and extending findings on the link between values and “dark” personalities. *Personality and Individual Differences, 109*, 142-147. doi:10.1016/j.paid.2016.12.055

Balcerowska, J. M., Biernatowska, A., Golińska, P., & Barańska, J. (2019). Relationship between dimensions of grandiose narcissism and Facebook addiction among university students. *Current Issues in Personality Psychology, 7*(4), 313-323. doi:10.5114/cipp.2019.92957

Bang, H., Choi, D., Wojdynski, B. W., & Lee, Y. I. (2019). How the level of personalization affects the effectiveness of personalized ad messages: the moderating role of narcissism. *International Journal of Advertising, 38*(8), 1116-1138. doi:10.1080/02650487.2019.1590069

Barelds, D., & Dijkstra, P. (2010). Narcissistic personality inventory: Structure of the adapted dutch version. *Scandinavian Journal of Psychology, 51*(2), 132-138. doi:10.1111/j.1467-9450.2009.00737.x

Barry, C. T., Chaplin, W. F., & Grafeman, S. J. (2006). Aggression following performance feedback: The influences of narcissism, feedback valence, and comparative standard. *Personality and Individual Differences, 41*(1), 177-187. doi:10.1016/j.paid.2006.01.008

Barry, C. T., Doucette, H., Loflin, D. C., Rivera-Hudson, N., & Herrington, L. L. (2017). "Let me take a selfie": Associations between self-photography, narcissism, and self-esteem. *Psychology of Popular Media Culture, 6*(1), 48-60. doi:10.1037/ppm0000089

Barry, C. T., McDougall, K. H., Anderson, A. C., Perkins, M. D., Lee-Rowland, L. M., Bender, I., & Charles, N. E. (2019). ‘Check Your Selfie before You Wreck Your Selfie’: Personality ratings of Instagram users as a function of self-image posts. *Journal of Research in Personality, 82*. doi:10.1016/j.jrp.2019.07.001

Barry, C. T., Reiter, S. R., Anderson, A. C., Schoessler, M. L., & Sidoti, C. L. (2019). "Let me take another selfie": Further examination of the relation between narcissism, self-perception, and instagram posts. *Psychology of Popular Media Culture, 8*(1), 22-33. doi:10.1037/ppm0000155

Basran, J., Pires, C., Matos, M., McEwan, K., & Gilbert, P. (2019). Styles of leadership, fears of compassion, and competing to avoid inferiority. *Frontiers in Psychology, 9*(JAN). doi:10.3389/fpsyg.2018.02460

Baughman, H. M. (2015). Relationships between the Dark Triad and delayed gratification: An evolutionary perspective.

Baum, N., & Shnit, D. (2003). Divorced parents’ conflict management Styles: Self-differentiation and narcissism. *Journal of Divorce and Remarriage, 39*(3-4), 37-58. doi:10.1300/J087v39n03_02

Baum, N., & Shnit, D. (2005). Self-differentiation and narcissism in divorced parents' co-parental relationships and functioning. *Journal of Divorce and Remarriage, 42*(3-4), 33-60. doi:10.1300/J087v42n03_03

Beattie, P., Bettache, K., & Chong, K. C. Y. (2019). Who is the Neoliberal? Exploring Neoliberal Beliefs across East and West. *Journal of Social Issues, 75*(1), 20-48. doi:10.1111/josi.12309

Beattie, S., Dempsey, C., Roberts, R., Woodman, T., & Cooke, A. (2017). The moderating role of narcissism on the reciprocal relationship between self-efficacy and performance. *Sport, Exercise, and Performance Psychology, 6*(2), 199-214. doi:10.1037/spy0000092

Belmi, P., & Schroeder, J. (2021). Human “resources”? Objectification at work. *Journal of Personality and Social Psychology, 120*(2), 384-417. doi:10.1037/pspi0000254

Ben Shlomo, S., & Taubman-Ben-Ari, O. (2017). What factors may assist social workers to promote life satisfaction and personal growth among first-time grandfathers? *Child and Family Social Work, 22*(1), 482-491. doi:10.1111/cfs.12267

Benjaminsen, S., Krarup, G., & Lauritsen, R. (1990). Personality, parental rearing behaviour and parental loss in attempted suicide: a comparative study. *Acta Psychiatrica Scandinavica, 82*(5), 389-397. doi:10.1111/j.1600-0447.1990.tb01408.x

Bell, E., Kowalski, C. M., Vernon, P. A., & Schermer, J. A. (2021). Political Hearts of Darkness: The Dark Triad as Predictors of Political Orientations and Interest in Politics. *Behavioral Sciences*, *11*(12). <https://doi.org/10.3390/bs11120169>

Benson, A. J., Jordan, C. H., & Christie, A. M. (2016). Narcissistic Reactions to Subordinate Role Assignment: The Case of the Narcissistic Follower. *Personality and Social Psychology Bulletin, 42*(7), 985-999. doi:10.1177/0146167216649608

Berg, J. M., Lilienfeld, S. O., & Waldman, I. D. (2013). Bargaining with the devil: Using economic decision-making tasks to examine the heterogeneity of psychopathic traits. *Journal of Research in Personality, 47*(5), 472-482. doi:10.1016/j.jrp.2013.04.003

Bergman, J. Z., Westerman, J. W., Bergman, S. M., Westerman, J., & Daly, J. P. (2014). Narcissism, Materialism, and Environmental Ethics in Business Students. *Journal of Management Education, 38*(4), 489-510. doi:10.1177/1052562913488108

Bergman, S. M., Fearrington, M. E., Davenport, S. W., & Bergman, J. Z. (2011). Millennials, narcissism, and social networking: What narcissists do on social networking sites and why. *Personality and Individual Differences, 50*(5), 706-711. doi:10.1016/j.paid.2010.12.022

Bertl, B., Pietschnig, J., Tran, U. S., Stieger, S., & Voracek, M. (2017). More or less than the sum of its parts? Mapping the Dark Triad of personality onto a single Dark Core. *Personality and Individual Differences, 114*, 140-144. doi:10.1016/j.paid.2017.04.002

Biancalani, G., Ronconi, L., & Testoni, I. (2023). Differences in Social Networking Behaviors Between Italian Gay and Heterosexual Men. *Sexuality and Culture*, *27*(1), 326-342. <https://doi.org/10.1007/s12119-022-10015-5>

Bianchi, E. C. (2014). Entering Adulthood in a Recession Tempers Later Narcissism. *Psychological Science, 25*(7), 1429-1437. doi:10.1177/0956797614532818

Biesen, J. N., & Smith, D. A. (2023). Narcissism in Romantic Relationships: Using Communal Activation to Promote Relationship Enhancing Attitudes. *Journal of Psychology*, *157*(8), 516-547. <https://doi.org/10.1080/00223980.2023.2255925>

Billet, M. I., & Fekken, G. C. (2020). The influence of instrumentality in trusting Dark Triad members. *Personality and Individual Differences, 154*. doi:10.1016/j.paid.2019.109690

Biolcati, R., & Passini, S. (2018). Narcissism and self-esteem: Different motivations for selfie posting behaviors. *Cogent Psychology, 5*(1). doi:10.1080/23311908.2018.1437012

Biolcati, R., Passini, S., & Griffiths, M. D. (2015). All-in and bad beat: Professional poker players and pathological gambling. *International Journal of Mental Health and Addiction, 13*(1), 19-32. doi:10.1007/s11469-014-9506-1

Bird, B. M., Carre, J. M., Knack, J. M., & Arnocky, S. (2016). Threatening men's mate value influences aggression toward an intrasexual rival: The moderating role of narcissism. *American Journal of Psychology, 129*(2), 169-183. doi:10.5406/amerjpsyc.129.2.0169

Biscardi, D., & Schill, T. (1985). CORRELATIONS OF NARCISSISTIC TRAITS WITH DEFENSIVE STYLE, MACHIAVELLIANISM, AND EMPATHY. *Psychological Reports, 57*(2), 354-354. doi:10.2466/pr0.1985.57.2.354

Bizumic, B., & Duckitt, J. (2008). "My group is not worthy of me": Narcissism and ethnocentrism. *Political Psychology, 29*(3), 437-453. doi:10.1111/j.1467-9221.2008.00638.x

Blair, C. A., Palmieri, R. E., & Paz-Aparicio, C. (2018). Do Big 5 personality characteristics and narcissism predict engagement in leader development? *Frontiers in Psychology, 9*(SEP). doi:10.3389/fpsyg.2018.01817

Blanchard, A. E., Keenan, G., Heym, N., & Sumich, A. (2023). COVID-19 prevention behaviour is differentially motivated by primary psychopathy, grandiose narcissism and vulnerable Dark Triad traits. *Personality and Individual Differences*, *204*. <https://doi.org/10.1016/j.paid.2022.112060>

Bleske-Rechek, A., Remiker, M. W., & Baker, J. P. (2008). Narcissistic men and women think they are so hot - But they are not. *Personality and Individual Differences, 45*(5), 420-424. doi:10.1016/j.paid.2008.05.018

Bleske-Rechek, A., Remiker, M. W., & Baker, J. P. (2009). Similar from the start: Assortment in young adult dating couples and its link to relationship stability over time. *Individual Differences Research, 7*(3), 142-158. Retrieved from https://www.scopus.com/inward/record.uri?eid=2-s2.0-70350608407&partnerID=40&md5=70b61f29841bfc43b4aa913940c1087d

Blinkhorn, V., Lyons, M., & Almond, L. (2015). The ultimate femme fatale? Narcissism predicts serious and aggressive sexually coercive behaviour in females. *Personality and Individual Differences, 87*, 219-223. doi:10.1016/j.paid.2015.08.001

Blinkhorn, V., Lyons, M., & Almond, L. (2019). Criminal Minds: Narcissism Predicts Offending Behavior in a Non-Forensic Sample. *Deviant Behavior, 40*(3), 353-360. doi:10.1080/01639625.2017.1422458

Blinkhorn, V., Lyons, M., Collier, E. S., & Almond, L. (2021). The relationship between narcissism and acceptance of violence revealed through a game designed to induce social ostracism. *Journal of Social Psychology, 161*(3), 261-271. doi:10.1080/00224545.2020.1816884

Bloxsom, C. A. J., Firth, J., Kibowski, F., Egan, V., Sumich, A. L., & Heym, N. (2021). Dark shadow of the self: How the dark triad and empathy impact parental and intimate adult attachment relationships in women. *Forensic Science International: Mind and Law*, *2*. <https://doi.org/10.1016/j.fsiml.2021.100045>

Bodroža, B. (2014). Validation of two conceptualizations of fragile self-esteem: Contingent high self-esteem and incongruent high self-esteem. *Psihologija, 47*(4), 373-391. doi:10.2298/PSI1404373B

Bogart, L. M., Benotsch, E. G., & Pavlovic, J. D. (2004). Feeling Superior but Threatened: The Relation of Narcissism to Social Comparison. *Basic and Applied Social Psychology, 26*(1), 35-44. doi:10.1207/s15324834basp2601_4

Boldero, J. M., Bell, R. C., & Davies, R. C. (2015). The Structure of the Narcissistic Personality Inventory with Binary and Rating Scale Items. *Journal of Personality Assessment, 97*(6), 626-637. doi:10.1080/00223891.2015.1039015

Boldero, J. M., Higgins, E. T., & Hulbert, C. A. (2015). Self-regulatory and narcissistic grandiosity and vulnerability: Common and discriminant relations. *Personality and Individual Differences, 76*, 171-176. doi:10.1016/j.paid.2014.12.019

Borkenau, P., & Zaltauskas, K. (2009). Effects of self-enhancement on agreement on personality profiles. *European Journal of Personality, 23*(2), 107-123. doi:10.1002/per.707

Boulter, M. W., Roberts, R., & Hardy, J. (2022). Upsetting the apple cart: Within-team profiles of intragroup conflict and their associations with narcissism. *Psychology of Sport and Exercise*, *63*. <https://doi.org/10.1016/j.psychsport.2022.102291>

Boulter, M. W., & Sandgren, S. S. (2022). Me, myself, and my muscles: associations between narcissism and muscle dysmorphia. *Eating Disorders*, *30*(1), 110-116. <https://doi.org/10.1080/10640266.2021.1930348>

Bowes, S. M., Costello, T. H., Ma, W., & Lilienfeld, S. O. (2020). Looking under the tinfoil hat: Clarifying the personological and psychopathological correlates of conspiracy beliefs. *Journal of Personality*. doi:10.1111/jopy.12588

Braddock, K., Schumann, S., Corner, E., & Gill, P. (2022). The Moderating Effects of “Dark” Personality Traits and Message Vividness on the Persuasiveness of Terrorist Narrative Propaganda. *Frontiers in Psychology*, *13*. <https://doi.org/10.3389/fpsyg.2022.779836>

Bradlee, P. M., & Emmons, R. A. (1992). Locating narcissism within the interpersonal circumplex and the five-factor model. *Personality and Individual Differences, 13*(7), 821-830. doi:10.1016/0191-8869(92)90056-U

Brailovskaia, J., & Bierhoff, H. W. (2016). Cross-cultural narcissism on Facebook: Relationship between self-presentation, social interaction and the open and covert narcissism on a social networking site in Germany and Russia. *Computers in Human Behavior, 55*, 251-257. doi:10.1016/j.chb.2015.09.018

Brailovskaia, J., Teismann, T., Zhang, X. C., & Margraf, J. (2019). Grandiose narcissism, depression and suicide ideation in Chinese and German students. *Current Psychology*, *40*(8), 3922-3930. <https://doi.org/10.1007/s12144-019-00355-1>

Brailovskaia, J., & Bierhoff, H. W. (2020). The Narcissistic Millennial Generation: A Study of Personality Traits and Online Behavior on Facebook. *Journal of Adult Development, 27*(1), 23-35. doi:10.1007/s10804-018-9321-1

Brailovskaia, J., Bierhoff, H. W., & Rohmann, E. (2021). Loneliness and depression symptoms: The moderating role of narcissism. *Journal of Affective Disorders Reports*, *6*. <https://doi.org/10.1016/j.jadr.2021.100264>

Brailovskaia, J., Bierhoff, H. W., & Margraf, J. (2019). How to Identify Narcissism With 13 Items? Validation of the German Narcissistic Personality Inventory–13 (G-NPI-13). *Assessment, 26*(4), 630-644. doi:10.1177/1073191117740625

Brailovskaia, J., Bierhoff, H. W., Rohmann, E., Raeder, F., & Margraf, J. (2020). The relationship between narcissism, intensity of Facebook use, Facebook flow and Facebook addiction. *Addictive Behaviors Reports, 11*. doi:10.1016/j.abrep.2020.100265

Brailovskaia, J., & Margraf, J. (2016). Comparing Facebook users and Facebook non-users: Relationship between personality traits and mental health variables - An exploratory study. *PLoS ONE, 11*(12). doi:10.1371/journal.pone.0166999

Brailovskaia, J., & Margraf, J. (2017). Facebook Addiction Disorder (FAD) among German students—A longitudinal approach. *PLoS ONE, 12*(12). doi:10.1371/journal.pone.0189719

Brailovskaia, J., & Margraf, J. (2018). What does media use reveal about personality and mental health? An exploratory investigation among German students. *PLoS ONE, 13*(1). doi:10.1371/journal.pone.0191810

Brailovskaia, J., & Margraf, J. (2019). I present myself and have a lot of Facebook-friends – Am I a happy narcissist!? *Personality and Individual Differences, 148*, 11-16. doi:10.1016/j.paid.2019.05.022

Brailovskaia, J., & Margraf, J. (2020). How to measure self-esteem with one item? validation of the German single-item self-esteem scale (G-SISE). *Current Psychology, 39*(6), 2192-2202. doi:10.1007/s12144-018-9911-x

Brailovskaia, J., Rohmann, E., Bierhoff, H. W., & Margraf, J. (2020). The anxious addictive narcissist: The relationship between grandiose and vulnerable narcissism, anxiety symptoms and Facebook Addiction. *PLoS ONE, 15*(11 November). doi:10.1371/journal.pone.0241632

Brailovskaia, J., Teismann, T., Zhang, X. C., & Margraf, J. (2019). Grandiose narcissism, depression and suicide ideation in Chinese and German students. *Current Psychology*. doi:10.1007/s12144-019-00355-1

Braun, S., Aydin, N., Frey, D., & Peus, C. (2018). Leader Narcissism Predicts Malicious Envy and Supervisor-Targeted Counterproductive Work Behavior: Evidence from Field and Experimental Research. *Journal of Business Ethics, 151*(3), 725-741. doi:10.1007/s10551-016-3224-5

Braun, S., Kempenaers, C., Linkowski, P., & Loas, G. (2016). French adaptation of the Narcissistic personality inventory in a Belgian French-speaking sample. *Frontiers in Psychology, 7*(DEC). doi:10.3389/fpsyg.2016.01980

Brender-Ilan, Y., & Sheaffer, Z. (2019). How do self-efficacy, narcissism and autonomy mediate the link between destructive leadership and counterproductive work behaviour. *Asia Pacific Management Review, 24*(3), 212-222. doi:10.1016/j.apmrv.2018.05.003

Brewer, G., Hunt, D., James, G., & Abell, L. (2015). Dark Triad traits, infidelity and romantic revenge. *Personality and Individual Differences, 83*, 122-127. doi:10.1016/j.paid.2015.04.007

Brewer, G., Lyons, M., Perry, A., & O’Brien, F. (2019). Dark Triad Traits and Perceptions of Sexual Harassment. *Journal of Interpersonal Violence*. doi:10.1177/0886260519827666

Brewer, G., Lyons, M., Perry, A., & O'Brien, F. (2021). Dark Triad Traits and Perceptions of Sexual Harassment. *Journal of Interpersonal Violence*, *36*(13-14). <https://doi.org/10.1177/0886260519827666>

Briganti, G., & Linkowski, P. (2020). Exploring network structure and central items of the Narcissistic Personality Inventory. *International Journal of Methods in Psychiatric Research, 29*(1). doi:10.1002/mpr.1810

Brotto, G. L. (2017). Toward a Psychological Typology for Victims of Interpersonal Violent Crimes: An Empirical Analysis of Behavioural.

Brown, A. A., & Brunell, A. B. (2017). The “modest mask”? An investigation of vulnerable narcissists' implicit self-esteem. *Personality and Individual Differences, 119*, 160-167. doi:10.1016/j.paid.2017.07.020

Brown, A. A., Freis, S. D., Carroll, P. J., & Arkin, R. M. (2016). Perceived agency mediates the link between the narcissistic subtypes and self-esteem. *Personality and Individual Differences, 90*, 124-129. doi:10.1016/j.paid.2015.10.055

Brown, J., & Graham, D. (2008). Body satisfaction in gym-active males: An exploration of sexuality, gender, and narcissism. *Sex Roles, 59*(1-2), 94-106. doi:10.1007/s11199-008-9416-4

Brown, R. P., Budzek, K., & Tamborski, M. (2009). On the meaning and measure of narcissism. *Personality and Social Psychology Bulletin, 35*(7), 951-964. doi:10.1177/0146167209335461

Brown, R. P., & Zeigler-Hill, V. (2004). Narcissism and the non-equivalence of self-esteem measures: A matter of dominance? *Journal of Research in Personality, 38*(6), 585-592. doi:10.1016/j.jrp.2003.11.002

Brummelman, E., Thomaes, S., Nelemans, S. A., de Castro, B. O., & Bushman, B. J. (2015). My child is god's gift to humanity: Development and validation of the parental overvaluation scale (POS). *Journal of Personality and Social Psychology, 108*(4), 665-679. doi:10.1037/pspp0000012

Brunell, A. B., & Buelow, M. T. (2015). Narcissism and Performance on Behavioral Decision-making Tasks. *Journal of Behavioral Decision Making, 30*(1), 3-14. doi:10.1002/bdm.1900

Brunell, A. B., & Buelow, M. T. (2018a). Homogenous scales of narcissism: Using the psychological entitlement scale, interpersonal exploitativeness scale, and narcissistic grandiosity scale to study narcissism. *Personality and Individual Differences, 123*, 182-190. doi:10.1016/j.paid.2017.11.029

Brunell, A. B., Wicker, J. L., Deems, N. P., & Daddis, C. (2018b). Can coders detect grandiose narcissism in others? *Current Psychology*, *40*(4), 1601-1607. <https://doi.org/10.1007/s12144-018-0085-3>

Brunell, A. B., & Buelow, M. T. (2019). Using the bogus pipeline to investigate trait narcissism and well-being. *Personality and Individual Differences, 151*. doi:10.1016/j.paid.2019.109509

Brunell, A. B., & Davis, M. S. (2016). Grandiose Narcissism and Fairness in Social Exchanges. *Current Psychology, 35*(2), 220-233. doi:10.1007/s12144-016-9415-5

Brunell, A. B., Davis, M. S., Schley, D. R., Eng, A. L., van Dulmen, M. H. M., Wester, K. L., & Flannery, D. J. (2013). A new measure of interpersonal exploitativeness. *Frontiers in Psychology, 4*(MAY). doi:10.3389/fpsyg.2013.00299

Brunell, A. B., & Fisher, T. D. (2014). Using the bogus pipeline to investigate grandiose narcissism. *Journal of Experimental Social Psychology, 55*, 37-42. doi:10.1016/j.jesp.2014.05.015

Brunell, A. B., Robison, J., Deems, N. P., & Okdie, B. M. (2018). Are narcissists more attracted to people in relationships than to people not in relationships? *PLoS ONE, 13*(3). doi:10.1371/journal.pone.0194106

Brunell, A. B., Staats, S., Barden, J., & Hupp, J. M. (2011). Narcissism and academic dishonesty: The exhibitionism dimension and the lack of guilt. *Personality and Individual Differences, 50*(3), 323-328. doi:10.1016/j.paid.2010.10.006

Brunell, A. B., Tumblin, L., & Buelow, M. T. (2014). Narcissism and the Motivation to Engage in Volunteerism. *Current Psychology, 33*(3), 365-376. doi:10.1007/s12144-014-9216-7

Bruno, A., Quattrone, D., Scimeca, G., Cicciarelli, C., Romeo, V. M., Pandolfo, G., . . . Muscatello, M. R. (2014). Unraveling exercise addiction: the role of narcissism and self-esteem. *J Addict, 2014*, 987841. doi:10.1155/2014/987841

Bucknall, V., Burwaiss, S., MacDonald, D., Charles, K., & Clement, R. (2015). Mirror mirror on the ward, who's the most narcissistic of them all? Pathologic personality traits in health care. *CMAJ, 187*(18), 1359-1363. doi:10.1503/cmaj.151135

Buelow, M. T., & Brunell, A. B. (2014). Facets of grandiose narcissism predict involvement in health-risk behaviors. *Personality and Individual Differences, 69*, 193-198. doi:10.1016/j.paid.2014.05.031

Buffardi, L. E., & Campbell, W. K. (2008). Narcissism and social networking web sites. *Personality and Social Psychology Bulletin, 34*(10), 1303-1314. doi:10.1177/0146167208320061

Buratti, S., Allwood, C. M., & Kleitman, S. (2013). First- and second-order metacognitive judgments of semantic memory reports: The influence of personality traits and cognitive styles. *Metacognition and Learning, 8*(1), 79-102. doi:10.1007/s11409-013-9096-5

Burris, C. T., Rempel, J. K., Munteanu, A. R., & Therrien, P. A. (2013). More, More, More: The Dark Side of Self-Expansion Motivation. *Personality and Social Psychology Bulletin, 39*(5), 578-595. doi:10.1177/0146167213479134

Burton, K. A., Adams, J. M., Hart, W., Grant, B., Richardson, K., & Tortoriello, G. (2017). You remind me of someone awesome: Narcissistic tolerance is driven by perceived similarity. *Personality and Individual Differences, 104*, 499-503. doi:10.1016/j.paid.2016.09.019

Bushman, B. J., & Baumeister, R. F. (1998). Threatened Egotism, Narcissism, Self-Esteem, and Direct and Displaced Aggression: Does Self-Love or Self-Hate Lead to Violence? *Journal of Personality and Social Psychology, 75*(1), 219-229. doi:10.1037/0022-3514.75.1.219

Bushman, B. J., Baumeister, R. F., Thomaes, S., Ryu, E., Begeer, S., & West, S. G. (2009). Looking again, and harder, for a link between low self-esteem and aggression. *Journal of Personality, 77*(2), 427-446. doi:10.1111/j.1467-6494.2008.00553.x

Bushman, B. J., Bonacci, A. M., Van Dijk, M., & Baumeister, R. F. (2003). Narcissism, Sexual Refusal, and Aggression: Testing a Narcissistic Reactance Model of Sexual Coercion. *Journal of Personality and Social Psychology, 84*(5), 1027-1040. doi:10.1037/0022-3514.84.5.1027

Bushman, B. J., Moeller, S. J., & Crocker, J. (2011). Sweets, Sex, or Self-Esteem? Comparing the Value of Self-Esteem Boosts With Other Pleasant Rewards. *Journal of Personality, 79*(5), 993-1012. doi:10.1111/j.1467-6494.2011.00712.x

Bushman, B. J., Steffgen, G., Kerwin, T., Whitlock, T., & Weisenberger, J. M. (2018). “Don't you know I own the road?” The link between narcissism and aggressive driving. *Transportation Research Part F: Traffic Psychology and Behaviour, 52*, 14-20. doi:10.1016/j.trf.2017.10.008

Byrne, K. A., & Worthy, D. A. (2013). Do narcissists make better decisions? An investigation of narcissism and dynamic decision-making performance. *Personality and Individual Differences, 55*(2), 112-117. doi:10.1016/j.paid.2013.02.020

Cai, H., Kwan, V. S. Y., & Sedikides, C. (2012). A Sociocultural Approach to Narcissism: The Case of Modern China. *European Journal of Personality, 26*(5), 529-535. doi:10.1002/per.852

Cai, H. J., & Gries, P. (2013). National narcissism: Internal dimensions and international correlates. *Psych Journal, 2*(2), 122-132. doi:10.1002/pchj.26

Cai, H. J., Shi, Y. Y., Fang, X., & Luo, Y. L. L. (2015). Narcissism predicts impulsive buying: phenotypic and genetic evidence. *Frontiers in Psychology, 6*. doi:10.3389/fpsyg.2015.00881

Caiozzo, C. N., Houston, J., & Grych, J. (2016). Predicting aggression in late adolescent romantic relationships: A short-term longitudinal study. *Journal of Adolescence, 53*, 237-248. doi:10.1016/j.adolescence.2016.10.012

Cao, C., Lou, J., & Liu, W. (2022). Local inefficiency of the default mode network in young men with narcissistic personality disorder. *Neuroscience Letters*, *784*. <https://doi.org/10.1016/j.neulet.2022.136720>

Cairncross, M., Veselka, L., Schermer, J. A., & Vernon, P. A. (2013). A behavioral genetic analysis of alexithymia and the dark triad traits of personality. *Twin Research and Human Genetics, 16*(3), 690-697. doi:10.1017/thg.2013.19

Campbell, J., Schermer, J. A., Villani, V. C., Nguyen, B., Vickers, L., & Vernon, P. A. (2009). A behavioral genetic study of the dark triad of personality and moral development. *Twin Research and Human Genetics, 12*(2), 132-136. doi:10.1375/twin.12.2.132

Campbell, R. L., Eisner, S., & Riggs, N. (2010). Sources of self-esteem: From theory to measurement and back again. *New Ideas in Psychology, 28*(3), 338-349. doi:10.1016/j.newideapsych.2009.09.008

Campbell, W. K. (1999). Narcissism and romantic attraction. *Journal of Personality and Social Psychology, 77*(6), 1254-1270. doi:10.1037/0022-3514.77.6.1254

Campbell, W. K., Bosson, J. K., Goheen, T. W., Lakey, C. E., & Kernis, M. H. (2007). Do narcissists dislike themselves "deep down inside"? *Psychological Science, 18*(3), 227-229. doi:10.1111/j.1467-9280.2007.01880.x

Campbell, W. K., Bush, C. P., Brunell, A. B., & Shelton, J. (2005). Understanding the social costs of narcissism: The case of the tragedy of the commons. *Personality and Social Psychology Bulletin, 31*(10), 1358-1368. doi:10.1177/0146167205274855

Campbell, W. K., & Foster, C. A. (2002). Narcissism and commitment in romantic relationships: An investment model analysis. *Personality and Social Psychology Bulletin, 28*(4), 484-495. doi:10.1177/0146167202287006

Campbell, W. K., Foster, C. A., & Finkel, E. J. (2002). Does self-love lead to love for others? A story of narcissistic game playing. *Journal of Personality and Social Psychology, 83*(2), 340-354. doi:10.1037/0022-3514.83.2.340

Campbell, W. K., Goodie, A. S., & Foster, J. D. (2004). Narcissism, confidence, and risk attitude. *Journal of Behavioral Decision Making, 17*(4), 297-311. doi:10.1002/bdm.475

Campbell, W. K., Miller, J. D., & Buffardi, L. E. (2010). The United States and the "Culture of Narcissism": An Examination of Perceptions of National Character. *Social Psychological and Personality Science, 1*(3), 222-229. doi:10.1177/1948550610366878

Campbell, W. K., Reeder, G. D., Sedikides, C., & Elliot, A. J. (2000). Narcissism and Comparative Self-Enhancement Strategies. *Journal of Research in Personality, 34*(3), 329-347. doi:10.1006/jrpe.2000.2282

Campbell, W. K., Rudich, E. A., & Sedikides, C. (2002). Narcissism, self-esteem, and the positivity of self-views: Two portraits of self-love. *Personality and Social Psychology Bulletin, 28*(3), 358-368. doi:10.1177/0146167202286007

Carey, A. L., Brucks, M. S., Küfner, A. C. P., Holtzman, N. S., Deters, F. G., Back, M. D., . . . Mehl, M. R. (2015). Narcissism and the use of personal pronouns revisited. *Journal of Personality and Social Psychology, 109*(3), e1-e15. doi:10.1037/pspp0000029

Carlson, E. N. (2013). Honestly Arrogant or Simply Misunderstood? Narcissists' Awareness of their Narcissism. *Self and Identity, 12*(3), 259-277. doi:10.1080/15298868.2012.659427

Carlson, E. N., & DesJardins, N. M. L. (2015). Do mean guys always finish first or just say that they do? Narcissists’ awareness of their social status and popularity over time. *Personality and Social Psychology Bulletin, 41*(7), 901-917. doi:10.1177/0146167215581712

Carlson, E. N., Naumann, L. P., & Vazire, S. (2012). Getting to Know a Narcissist Inside and Out. In *The Handbook of Narcissism and Narcissistic Personality Disorder: Theoretical Approaches, Empirical Findings, and Treatments* (pp. 283-299).

Carlson, E. N., Vazire, S., & Oltmanns, T. F. (2011). You Probably Think This Paper's About You: Narcissists' Perceptions of Their Personality and Reputation. *Journal of Personality and Social Psychology, 101*(1), 185-201. doi:10.1037/a0023781

Carmody, P., & Gordon, K. (2011). Offender variables: Unique predictors of Benevolence, Avoidance, and Revenge? *Personality and Individual Differences, 50*(7), 1012-1017. doi:10.1016/j.paid.2010.12.037

Carnahan, T., & McFarland, S. (2007). Revisiting the stanford prison experiment: Could participant self-selection have led to the cruelty? *Personality and Social Psychology Bulletin, 33*(5), 603-614. doi:10.1177/0146167206292689

Carnevale, J. B., Huang, L., & Harms, P. D. (2018). Leader consultation mitigates the harmful effects of leader narcissism: A belongingness perspective. *Organizational Behavior and Human Decision Processes, 146*, 76-84. doi:10.1016/j.obhdp.2018.04.003

Carr, D. L. (2008). *Relationships among overt and covert narcissism and vocational interests with respect to gender*: The Florida State University.

Carroll, L. (1987). A STUDY OF NARCISSISM, AFFILIATION, INTIMACY, AND POWER MOTIVES AMONG STUDENTS IN BUSINESS ADMINISTRATION. *Psychological Reports, 61*(2), 355-358. doi:10.2466/pr0.1987.61.2.355

Carroll, L. (1989). A COMPARATIVE-STUDY OF NARCISSISM, GENDER, AND SEX-ROLE ORIENTATION AMONG BODYBUILDERS, ATHLETES, AND PSYCHOLOGY STUDENTS. *Psychological Reports, 64*(3), 999-1006. doi:10.2466/pr0.1989.64.3.999

Carter, G. L., & Douglass, M. D. (2018). The aging narcissus: Just a myth? Narcissism moderates the age-loneliness relationship in older age. *Frontiers in Psychology, 9*(JUL). doi:10.3389/fpsyg.2018.01254

Casale, S., & Fioravanti, G. (2018). Why narcissists are at risk for developing Facebook addiction: The need to be admired and the need to belong. *Addictive Behaviors, 76*, 312-318. doi:10.1016/j.addbeh.2017.08.038

Casale, S., Fioravanti, G., Baldi, V., Flett, G. L., & Hewitt, P. L. (2020). Narcissism, perfectionistic self-presentation, and relationship satisfaction from a dyadic perspective: Narcissism and Relationship Satisfaction. *Self and Identity, 19*(8), 948-966. doi:10.1080/15298868.2019.1707272

Casale, S., Fioravanti, G., & Rugai, L. (2016). Grandiose and Vulnerable Narcissists: Who is at Higher Risk for Social Networking Addiction? *Cyberpsychology, Behavior, and Social Networking, 19*(8), 510-515. doi:10.1089/cyber.2016.0189

Casale, S., Fioravanti, G., Rugai, L., Flett, G. L., & Hewitt, P. L. (2016). The interpersonal expression of perfectionism among grandiose and vulnerable narcissists: Perfectionistic self-presentation, effortless perfection, and the ability to seem perfect. *Personality and Individual Differences, 99*, 320-324. doi:10.1016/j.paid.2016.05.026

Casale, S., Rugai, L., Fioravanti, G., & Puccetti, C. (2018). Narcissism and authentic self: An unfeasible marriage? *Personality and Individual Differences, 135*, 131-136. doi:10.1016/j.paid.2018.07.008

Cashwell, C. S., Glosoff, H. L., & Hammond, C. (2010). Spiritual bypass: A preliminary investigation. *Counseling and Values, 54*(2), 162-174. doi:10.1002/j.2161-007X.2010.tb00014.x

Chabrol, H., Bronchain, J., Morgades Bamba, C. I., & Raynal, P. (2020). The Dark Tetrad and radicalization: personality profiles in young women. *Behavioral Sciences of Terrorism and Political Aggression, 12*(2), 157-168. doi:10.1080/19434472.2019.1646301

Chamberlain, J. M., & Haaga, D. A. F. (2001). Unconditional self-acceptance and psychological health. *Journal of Rational - Emotive and Cognitive - Behavior Therapy, 19*(3), 163-176. doi:10.1023/A:1011189416600

Chan, C. Y., & Cheung, K. L. (2020). Exploring the gender difference in relationships between narcissism, competitiveness, and mental health problems among college students. *Journal of American College Health*, 1-10. doi:10.1080/07448481.2020.1788565

Charoensukmongkol, P. (2016). Exploring personal characteristics associated with selfie-liking. *Cyberpsychology, 10*(2). doi:10.5817/CP2016-2-7

Chen, G. M. (2014). Revisiting the social enhancement hypothesis: Extroversion indirectly predicts number of Facebook friends operating through Facebook usage. *Computers in Human Behavior, 39*, 263-269. doi:10.1016/j.chb.2014.07.015

Chen, J., Nevicka, B., Homan, A. C., & van Kleef, G. A. (2021). How Narcissism Shapes Responses to Antisocial and Prosocial Behavior: Hypo-Responsiveness or Hyper-Responsiveness? *Personality and Social Psychology Bulletin*. doi:10.1177/01461672211007293

Chen, L. (2016). Linking leader personality traits to motivation to lead: A self-concept approach. *Social Behavior and Personality, 44*(11), 1913-1925. doi:10.2224/sbp.2016.44.11.1913

Chen, S., Friesdorf, R., & Jordan, C. H. (2021). State and trait narcissism predict everyday helping. *Self and Identity, 20*(2), 182-198. doi:10.1080/15298868.2019.1598892

Chen, Y., Ferris, D. L., Kwan, H. K., Yan, M., Zhou, M., & Hong, Y. (2013). Self-love's lost labor: A self-enhancement model of workplace incivility. *Academy of Management Journal, 56*(4), 1199-1219. doi:10.5465/amj.2010.0906

Cheng, J. T., Tracy, J. L., Foulsham, T., Kingstone, A., & Henrich, J. (2013). Two ways to the top: evidence that dominance and prestige are distinct yet viable avenues to social rank and influence. *Journal of Personality and Social Psychology, 104*(1), 103.

Cheng, J. T., Tracy, J. L., & Henrich, J. (2010). Pride, personality, and the evolutionary foundations of human social status. *Evolution and Human Behavior, 31*(5), 334-347. doi:10.1016/j.evolhumbehav.2010.02.004

Cheng, J. T., Tracy, J. L., & Miller, G. E. (2013). Are narcissists hardy or vulnerable? The role of narcissism in the production of stress-related biomarkers in response to emotional distress. *Emotion, 13*(6), 1004-1011. doi:10.1037/a0034410

Chester, D. S., & Dewall, C. N. (2016). Sound the Alarm: The Effect of Narcissism on Retaliatory Aggression Is Moderated by dACC Reactivity to Rejection. *Journal of Personality, 84*(3), 361-368. doi:10.1111/jopy.12164

Cheung, H. Y., Wu, J., & Tao, J. (2016). Predicting domain-specific risk-taking attitudes of mainland China university students: A hyper core self-evaluation approach. *Journal of Risk Research, 19*(1), 79-100. doi:10.1080/13669877.2014.948903

Chiorri, C., Garofalo, C., & Velotti, P. (2019). Does the Dark Triad Manifest Similarly in men and Women? Measurement Invariance of the Dirty Dozen across sex. *Current Psychology, 38*(3), 659-675. doi:10.1007/s12144-017-9641-5

Choi, M., Panek, E. T., Nardis, Y., & Toma, C. L. (2015). When social media isn't social: Friends' responsiveness to narcissists on Facebook. *Personality and Individual Differences, 77*, 209-214. doi:10.1016/j.paid.2014.12.056

Chou, C. H., Lin, Y. W., Lin, C. H., & Farn, C. K. (2017). *Cannot live without Facebook? An expectation confirmation model with the moderating effects of narcissism*.

Chou, S. Y., & Ramser, C. (2023). Narcissists going above and beyond? The role of perceived negative inequity and self-enhancement motivation. *Personnel Review*, *52*(9), 2245-2264. <https://doi.org/10.1108/PR-11-2021-0799>

Chowdhury, R. (2023). Perceived Income Inequality, Trust, and Consumers' Ethical Judgments [Article]. *Journal of Macromarketing*, *43*(4), 476-493. <https://doi.org/10.1177/02761467231187306>

Church, B. K., Dai, N. T., Kuang, X., & Liu, X. (2020). The Role of Auditor Narcissism in Auditor-Client Negotiations: Evidence from China. *Contemporary Accounting Research, 37*(3), 1756-1787. doi:10.1111/1911-3846.12565

Cichocka, A., Dhont, K., & Makwana, A. P. (2017). On Self-Love and Outgroup Hate: Opposite Effects of Narcissism on Prejudice via Social Dominance Orientation and Right-Wing Authoritarianism. *European Journal of Personality, 31*(4), 366-384. doi:10.1002/per.2114

Cichocka, A., Marchlewska, M., & de Zavala, A. G. (2016). Does Self-Love or Self-Hate Predict Conspiracy Beliefs? Narcissism, Self-Esteem, and the Endorsement of Conspiracy Theories. *Social Psychological and Personality Science, 7*(2), 157-166. doi:10.1177/1948550615616170

Clark, M. A., Lelchook, A. M., & Taylor, M. L. (2010). Beyond the Big Five: How narcissism, perfectionism, and dispositional affect relate to workaholism. *Personality and Individual Differences, 48*(7), 786-791. doi:10.1016/j.paid.2010.01.013

Clemente, M., Padilla-Racero, D., & Espinosa, P. (2020). The Dark Triad and the Detection of Parental Judicial Manipulators. Development of a Judicial Manipulation Scale. *International Journal of Environmental Research and Public Health, 17*(8). doi:10.3390/ijerph17082843

Clements, Z. A., & Munro, G. D. (2021). Biases and their impact on opinions of transgender bathroom usage. *Journal of Applied Social Psychology, 51*(4), 370-383. doi:10.1111/jasp.12741

Coleman, S. R. M., Bernstein, M. J., Benfield, J. A., & Smyth, J. M. (2020). Narcissistic grandiosity and risky health behaviors in college students. *Journal of American College Health*. doi:10.1080/07448481.2020.1762606

Coleman, G., Furnham, A., & Treglown, L. (2022). Exploring the Dark side of conscientiousness. The relationship between conscientiousness and its potential derailers: perfectionism and narcissism. *Current Psychology*, *42*(31), 27744-27757. <https://doi.org/10.1007/s12144-022-03828-y>

Colle, L., Dimaggio, G., Carcione, A., Nicolò, G., Semerari, A., & Chiavarino, C. (2020). Do Competitive Contexts Affect Mindreading Performance? *Frontiers in Psychology, 11*. doi:10.3389/fpsyg.2020.01284

Collis, N., Lewis, V., & Crisp, D. (2016). When Is Buff Enough? the Effect of Body Attitudes and Narcissistic Traits on Muscle Dysmorphia. *Journal of Men's Studies, 24*(2), 213-225. doi:10.1177/1060826516641097

Collisson, B., Howell, J. L., & Harig, T. (2020). Foodie Calls: When Women Date Men for a Free Meal (Rather Than a Relationship). *Social Psychological and Personality Science, 11*(3), 425-432. doi:10.1177/1948550619856308

Cook, R. H., Griffiths, M. D., & Pontes, H. M. (2020). Personality Factors in Exercise Addiction: A Pilot Study Exploring the Role of Narcissism, Extraversion, and Agreeableness. *International Journal of Mental Health and Addiction, 18*(1), 89-102. doi:10.1007/s11469-018-9939-z

Cooper, A. B., Blake, A. B., Pauletti, R. E., Cooper, P. J., Sherman, R. A., & Lee, D. I. (2020). Personality Assessment through the Situational and Behavioral Features of Instagram Photos. *European Journal of Psychological Assessment, 36*(6), 959-972. doi:10.1027/1015-5759/a000596

Cooper, M. J., Pullig, C., & Dickens, C. (2016). Effects of Narcissism and Religiosity on Church Ministers With Respect to Ethical Judgment, Confidence, and Forgiveness. *Journal of Psychology and Theology, 44*(1), 42-54.

Cornish, M. A., Woodyatt, L., Morris, G., Conroy, A., & Townsdin, J. (2018). Self-forgiveness, self-exoneration, and self-condemnation: Individual differences associated with three patterns of responding to interpersonal offenses. *Personality and Individual Differences, 129*, 43-53. doi:10.1016/j.paid.2018.03.003

Corry, N., Merritt, R. D., Mrug, S., & Pamp, B. (2008). The factor structure of the narcissistic personality inventory. *Journal of Personality Assessment, 90*(6), 593-600. doi:10.1080/00223890802388590

Cramer, P. (1995). Identity, narcissism, and defence mechanisms in late adolescence. *Journal of Research in Personality, 29*(3), 341-361. doi:10.1006/jrpe.1995.1020

Creech, R. S. (2015). *Social Media, Social Exclusion, and Narcissism.* Xavier University,

Crossley, L., Woodworth, M., Black, P. J., & Hare, R. (2016). The dark side of negotiation: Examining the outcomes of face-to-face and computer-mediated negotiations among dark personalities. *Personality and Individual Differences, 91*, 47-51. doi:10.1016/j.paid.2015.11.052

Crouch, J. L., Hiraoka, R., Rutledge, E., Zengel, B., Skowronski, J. J., & Milner, J. S. (2015). Is Narcissism Associated with Child Physical Abuse Risk? *Journal of Family Violence, 30*(3), 373-380. doi:10.1007/s10896-015-9672-3

Cui, Z. L. (2023). Good soldiers or bad apples? Exploring the impact of employee narcissism on constructive and destructive voice. *Humanities & Social Sciences Communications*, *10*(1), 11. <https://doi.org/10.1057/s41599-023-02230-8>

Cutler, A. D., Carden, S. W., Dorough, H. L., & Holtzman, N. S. (2021). Inferring Grandiose Narcissism From Text: LIWC Versus Machine Learning. *Journal of Language and Social Psychology, 40*(2), 260-276. doi:10.1177/0261927X20936309

Czarna, A. Z., Czerniak, A., & Szmajke, A. (2014). Does communal context bring the worst in narcissists? *Polish Psychological Bulletin, 45*(4), 464-468. doi:10.2478/ppb-2014-0056

Czarna, A. Z., Dufner, M., & Clifton, A. D. (2014). The effects of vulnerable and grandiose narcissism on liking-based and disliking-based centrality in social networks. *Journal of Research in Personality, 50*(1), 42-45. doi:10.1016/j.jrp.2014.02.004

Czarna, A. Z., Jonason, P. K., Dufner, M., & Kossowska, M. (2016). The Dirty Dozen scale: Validation of a polish version and extension of the nomological net. *Frontiers in Psychology, 7*(MAR). doi:10.3389/fpsyg.2016.00445

Czarna, A. Z., Leifeld, P., Śmieja, M., Dufner, M., & Salovey, P. (2016). Do Narcissism and Emotional Intelligence Win Us Friends? Modeling Dynamics of Peer Popularity Using Inferential Network Analysis. *Personality and Social Psychology Bulletin, 42*(11), 1588-1599. doi:10.1177/0146167216666265

Czarna, A. Z., Śmieja, M., Wider, M., Dufner, M., & Sedikides, C. (2022). Narcissism and partner-enhancement at different relationship stages. *Journal of Research in Personality*, *98*. <https://doi.org/10.1016/j.jrp.2022.104212>

Czarna, A. Z., Wróbel, M., Dufner, M., & Zeigler-Hill, V. (2015). Narcissism and Emotional Contagion: Do Narcissists “Catch” the Emotions of Others? *Social Psychological and Personality Science, 6*(3), 318-324. doi:10.1177/1948550614559652

Czarna, A. Z., Zajenkowski, M., Maciantowicz, O., & Szymaniak, K. (2019). The relationship of narcissism with tendency to react with anger and hostility: The roles of neuroticism and emotion regulation ability. *Current Psychology*. doi:10.1007/s12144-019-00504-6

Czarna, A. Z., Zajenkowski, M., Maciantowicz, O., & Szymaniak, K. (2021). The relationship of narcissism with tendency to react with anger and hostility: The roles of neuroticism and emotion regulation ability. *Current Psychology*, *40*(11), 5499-5514. <https://doi.org/10.1007/s12144-019-00504-6>

Czibor, A., Szabo, Z. P., Jones, D. N., Zsido, A. N., Paal, T., Szijjarto, L., . . . Bereczkei, T. (2017). Male and female face of Machiavellianism: Opportunism or anxiety? *Personality and Individual Differences, 117*, 221-229. doi:10.1016/j.paid.2017.06.002

D'Souza, M. F., & de Lima, G. (2021). Narcissism, risk and uncertainties: analysis in the light of prospect and fuzzy-trace theories. *Rausp Management Journal, 56*(1), 129-147. doi:10.1108/RAUSP-10-2019-0226

Daddis, C., & Brunell, A. B. (2015). Entitlement, exploitativeness, and reasoning about everyday transgressions: A social domain analysis. *Journal of Research in Personality, 58*, 115-126. doi:10.1016/j.jrp.2015.07.007

Dahmen-Wassenberg, P., Kämmerle, M., Unterrainer, H. F., & Fink, A. (2016). The Relation Between Different Facets of Creativity and the Dark Side of Personality. *Creativity Research Journal, 28*(1), 60-66. doi:10.1080/10400419.2016.1125267

Dakanalis, A., Clerici, M., & Carrà, G. (2016). Narcissistic Vulnerability and Grandiosity as Mediators Between Insecure Attachment and Future Eating Disordered Behaviors: A Prospective Analysis of Over 2,000 Freshmen. *Journal of Clinical Psychology, 72*(3), 279-292. doi:10.1002/jclp.22237

Davenport, S. W., Bergman, S. M., Bergman, J. Z., & Fearrington, M. E. (2014). Twitter versus Facebook: Exploring the role of narcissism in the motives and usage of different social media platforms. *Computers in Human Behavior, 32*, 212-220. doi:10.1016/j.chb.2013.12.011

Davis, C., Claridge, G., & Brewer, H. (1996). The two faces of narcissism: Personality dynamics of body esteem. *Journal of Social and Clinical Psychology, 15*(2), 153-166. doi:10.1521/jscp.1996.15.2.153

Davis, C., Claridge, G., & Cerullo, D. (1997). Personality factors and weight preoccupation: A continuum approach to the association between eating disorders and personality disorders. *Journal of Psychiatric Research, 31*(4), 467-480. doi:10.1016/S0022-3956(97)00006-X

Davis, C., Dionne, M., & Shuster, B. (2001). Physical and psychological correlates of appearance orientation. *Personality and Individual Differences, 30*(1), 21-30. doi:10.1016/S0191-8869(00)00006-4

Davis, C., Karvinen, K., & McCreary, D. R. (2005). Personality correlates of a drive for muscularity in young men. *Personality and Individual Differences, 39*(2), 349-359. doi:10.1016/j.paid.2005.01.013

Davis, D. E., Worthington Jr, E. L., Hook, J. N., Emmons, R. A., Hill, P. C., Bollinger, R. A., & van Tongeren, D. R. (2013). Humility and the Development and Repair of Social Bonds: Two Longitudinal Studies. *Self and Identity, 12*(1), 58-77. doi:10.1080/15298868.2011.636509

Davis, M. S., & Brunell, A. B. (2012). Measuring Narcissism Within Add Health: The Development and Validation of a New Scale. *Journal of Research on Adolescence, 22*(4), 632-645. doi:10.1111/j.1532-7795.2012.00833.x

Day, L. C., Muise, A., & Impett, E. A. (2017). Is Comparison the Thief of Joy? Sexual Narcissism and Social Comparisons in the Domain of Sexuality. *Personality and Social Psychology Bulletin, 43*(2), 233-244. doi:10.1177/0146167216678862

de Bellis, E., Sprott, D. E., Herrmann, A., Bierhoff, H. W., & Rohmann, E. (2016). The Influence of Trait and State Narcissism on the Uniqueness of Mass-Customized Products. *Journal of Retailing, 92*(2), 162-172. doi:10.1016/j.jretai.2015.11.003

De Hoogh, A. H. B., Den Hartog, D. N., & Nevicka, B. (2015). Gender Differences in the Perceived Effectiveness of Narcissistic Leaders. *Applied Psychology, 64*(3), 473-498. doi:10.1111/apps.12015

de Roos, M. S., & Jones, D. N. (2021). Assessing Deception Differences with Mimicry Deception Theory. *Journal of Personality Assessment*. doi:10.1080/00223891.2021.1898969

de Zavala, A. G., & Bierwiaczonek, K. (2020). Male, National, and Religious Collective Narcissism Predict Sexism. *Sex Roles*. doi:10.1007/s11199-020-01193-3

de Zavala, A. G., Cichocka, A., Eidelson, R., & Jayawickreme, N. (2009). Collective Narcissism and Its Social Consequences. *Journal of Personality and Social Psychology, 97*(6), 1074-1096. doi:10.1037/a0016904

de Zavala, A. G., Cichocka, A., & Irena, I. G. (2013). Collective narcissism moderates the effect of in-group image threat on intergroup hostility. *Journal of Personality and Social Psychology, 104*(6), 1019-1039. doi:10.1037/a0032215

Dębska, M., Dębski, P., Polechoński, J., Rozpara, M., & Tomik, R. (2021). The dark triad of personality in the context of health behaviors: Ally or enemy? *International Journal of Environmental Research and Public Health, 18*(8). doi:10.3390/ijerph18084113

Dechesne, M. (2009). Explorations in the experimental social psychology of terrorism: The struggle-violence link and its predictors. *Revue Internationale de Psychologie Sociale, 22*(3-4), 87-102. Retrieved from https://www.scopus.com/inward/record.uri?eid=2-s2.0-77955332133&partnerID=40&md5=384fcc69370dbb401dfb78d6c8de081f

del Rosario, P. M., & White, R. M. (2005). The Narcissistic Personality Inventory: Test-retest stability and internal consistency. *Personality and Individual Differences, 39*(6), 1075-1081. doi:10.1016/j.paid.2005.08.001

Delič, L., Novak, P., Kovačič, J., & Avsec, A. (2011). Self-reported emotional and social intelligence and empathy as distinctive predictors of narcissism. *Psihologijske Teme, 20*(3), 477-488. Retrieved from https://www.scopus.com/inward/record.uri?eid=2-s2.0-84861132688&partnerID=40&md5=7a11b6241d93671264b837efcdacb076

DeMarree, K. G., Petty, R. E., Briñol, P., & Xia, J. (2020). Documenting individual differences in the propensity to hold attitudes with certainty. *Journal of Personality and Social Psychology, 119*(6), 1239-1265. doi:10.1037/pspa0000241

Demetrioff, S. (2013). Psychopathic Traits and Interpersonal Judgment: Examining Accuracy, Tendency, and Influence of Sex of Judge and Target.

Den Hartog, D. N., De Hoogh, A. H. B., & Belschak, F. D. (2020). Toot Your Own Horn? Leader Narcissism and the Effectiveness of Employee Self-Promotion. *Journal of Management, 46*(2), 261-286. doi:10.1177/0149206318785240

Deol, G., & Schermer, J. A. (2021). The dark side of MEE: The dark triad of personality and employee entitlement. *Personality and Individual Differences, 170*. doi:10.1016/j.paid.2020.110415

Derry, K. L., Ohan, J. L., & Bayliss, D. M. (2019). Toward understanding and measuring grandiose and vulnerable narcissism within trait personality models. *European Journal of Psychological Assessment, 35*(4), 498-511. doi:10.1027/1015-5759/a000432

Di Blasi, M., Giardina, A., Lo Coco, G., Giordano, C., Billieux, J., & Schimmenti, A. (2020). A compensatory model to understand dysfunctional personality traits in problematic gaming: The role of vulnerable narcissism. *Personality and Individual Differences, 160*. doi:10.1016/j.paid.2020.109921

Di Pierro, R., & Fanti, E. (2021). Self-Concept in Narcissism: Profile Comparisons of Narcissistic Manifestations on Facets of the Self. *Clin Neuropsychiatry*, *18*(4), 211-222. <https://doi.org/10.36131/cnfioritieditore20210404>

Dickinson, K. A., & Pincus, A. L. (2003). Interpersonal analysis of grandiose and vulnerable narcissism. *Journal of Personality Disorders, 17*(3), 188-207. doi:10.1521/pedi.17.3.188.22146

Dinić, B. M., & Vujić, A. (2019). Five-Factor Model Best Describes Narcissistic Personality Inventory Across Different Item Response Formats. *Psychological Reports, 122*(5), 1946-1966. doi:10.1177/0033294118794404

Dinić, B. M., & Vujić, A. (2020). The Pathological Narcissism Inventory: Measurement Invariance across Serbian and USA Samples and Further Validation. *European Journal of Psychological Assessment, 36*(4), 670-680. doi:10.1027/1015-5759/a000537

Dinić, B. M., Wertag, A., Tomašević, A., & Sokolovska, V. (2020). Centrality and redundancy of the Dark Tetrad traits. *Personality and Individual Differences, 155*. doi:10.1016/j.paid.2019.109621

Dirtu, M. C., & Prundeanu, O. (2023). Narcissism and Pro-Environmental Behaviors: The Mediating Role of Self-Monitoring, Environmental Control and Attitudes. *Sustainability*, *15*(2), 1571. <https://www.mdpi.com/2071-1050/15/2/1571>

Doerfler, S. M., Tajmirriyahi, M., Ickes, W., & Jonason, P. K. (2021). The self-concepts of people with Dark Triad traits tend to be weaker, less clearly defined, and more state-related. *Personality and Individual Differences, 180*. doi:10.1016/j.paid.2021.110977

Drat-Ruszczak, K., Bazińska, R., & Niemyjska, A. (2014). The mystery of communion in narcissism: The success-as-a-flaw effect. *Polish Psychological Bulletin, 45*(4), 453-463. doi:10.2478/ppb-2014-0055

DuBois, J. M., Chibnall, J. T., Tait, R. C., Vander Wal, J. S., Baldwin, K. A., Antes, A. L., & Mumford, M. D. (2016). Professional Decision-Making in Research (PDR): The Validity of a New Measure. *Science and Engineering Ethics, 22*(2), 391-416. doi:10.1007/s11948-015-9667-8

Dufner, M., Rauthmann, J. F., Czarna, A. Z., & Denissen, J. J. A. (2013). Are Narcissists Sexy? Zeroing in on the Effect of Narcissism on Short-Term Mate Appeal. *Personality and Social Psychology Bulletin, 39*(7), 870-882. doi:10.1177/0146167213483580

Dumas, T. M., Maxwell-Smith, M., Davis, J. P., & Giulietti, P. A. (2017). Lying or longing for likes? Narcissism, peer belonging, loneliness and normative versus deceptive like-seeking on Instagram in emerging adulthood. *Computers in Human Behavior, 71*, 1-10. doi:10.1016/j.chb.2017.01.037

Dumitrescu, A. L., Zetu, L., Zetu, M., & Pacurar, M. (2013). The Relationship between Narcissism, Oral Health Status and Oral Health-Related Behaviors. In C. Vasile, M. Anitei, & M. Chraif (Eds.), *PSIWORLD 2012* (Vol. 78, pp. 496-500).

Dunofsky, M. (1997). Psychological characteristics of women who undergo single and multiple cosmetic surgeries. *Annals of Plastic Surgery, 39*(3), 223-228. doi:10.1097/00000637-199709000-00001

Dworkis, K. K., & Patelli, L. (2022). Asymmetric responses to multidimensional performance evaluation systems: The role of non-pathological narcissism. *Management Accounting Research*, *57*. <https://doi.org/10.1016/j.mar.2022.100806>

Eaton, J., Ward Struthers, C., & Santelli, A. G. (2006). Dispositional and state forgiveness: The role of self-esteem, need for structure, and narcissism. *Personality and Individual Differences, 41*(2), 371-380. doi:E

Edelstein, R. S., Yim, I. S., & Quas, J. A. (2010). Narcissism predicts heightened cortisol reactivity to a psychosocial stressor in men. *Journal of Research in Personality, 44*(5), 565-572. doi:10.1016/j.jrp.2010.06.008

Edershile, E. A., Woods, W. C., Sharpe, B. M., Crowe, M. L., Miller, J. D., & Wright, A. G. C. (2019). A day in the life of narcissus: Measuring narcissistic grandiosity and vulnerability in daily life. *Psychological Assessment, 31*(7), 913-924. doi:10.1037/pas0000717

Efrati, Y., Gerber, Z., & Tolmacz, R. (2019). The Relation of Intra-Psychic and Relational Aspects of the Self to Compulsive Sexual Behavior. *Journal of Sex and Marital Therapy, 45*(7), 618-631. doi:10.1080/0092623X.2019.1599092

Ehrenberg, M. F., Hunter, M. A., & Elterman, M. F. (1996). Shared parenting agreements after marital separation: The roles of empathy and narcissism. *Journal of Consulting and Clinical Psychology, 64*(4), 808-818. doi:10.1037/0022-006X.64.4.808

Eksi, F. (2016). The short form of the five-factor narcissism inventory: Psychometric equivalence of the Turkish version. *Kuram ve Uygulamada Egitim Bilimleri, 16*(4), 1081-1096. doi:10.12738/estp.2016.4.0001

Elaad, E., Hanania, S. B., Mazor, S., & Zvi, L. (2020). The relations between deception, narcissism and self-assessed lie- and truth-related abilities. *Psychiatry, Psychology and Law, 27*(5), 880-893. doi:10.1080/13218719.2020.1751328

Elaad, E. (2022). Deceptive Behavior: Effects of Rational Thinking, Narcissism, and Self-Assessed Lie- and Truth Related Abilities. *Sage Open*, *12*(2). <https://doi.org/10.1177/21582440221085012>

Engyel, M., de Ruiter, N. M. P., & Urbán, R. (2022). Momentarily narcissistic? Development of a short, state version of the Pathological Narcissism Inventory applicable in momentary assessment. *Frontiers in Psychology*, *13*, 992271. <https://doi.org/10.3389/fpsyg.2022.992271>

Erkutlu, H. (2014). Exploring the moderating effect of psychological capital on the relationship between narcissism and psychological well-being. In M. Ozsahin (Ed.), *10TH INTERNATIONAL STRATEGIC MANAGEMENT CONFERENCE 2014* (Vol. 150, pp. 1148-1156).

Erkutlu, H., & Chafra, J. (2016). Impact of behavioral integrity on workplace ostracism: The moderating roles of narcissistic personality and psychological distance. *Journal of Applied Research in Higher Education, 8*(2), 222-237. doi:10.1108/JARHE-01-2015-0007

Eşkisu, M., Hoşoğlu, R., & Rasmussen, K. (2017). An investigation of the relationship between Facebook usage, Big Five, self-esteem and narcissism. *Computers in Human Behavior, 69*, 294-301. doi:10.1016/j.chb.2016.12.036

Estrin, T. D. (2003). *A sentence completion measure of Kohut and Wolf's narcissistic personality types.* Arts & Social Sciences: Department of Psychology,

Eterović, M., Medved, V., & Bilić, V. (2020). Unresponsiveness to intensified shame-inducing scenarios helps distinguish between genuine and defensive low shame in narcissism. *Psychoanalytic Psychology*. doi:10.1037/pap0000329

Etgar, S., & Amichai-Hamburger, Y. (2017). Not all selfies took alike: Distinct selfie motivations are related to different personality characteristics. *Frontiers in Psychology, 8*(MAY). doi:10.3389/fpsyg.2017.00842

Exline, J. J., Baumeister, R. F., Zell, A. L., Kraft, A. J., & Witvliet, C. V. O. (2008). Not so innocent: does seeing one's own capability for wrongdoing predict forgiveness? *Journal of Personality and Social Psychology, 94*(3), 495-515. doi:10.1037/0022-3514.94.3.495

Exline, J. J., Bushman, B. J., Baumeister, R. F., Keith Campbell, W., & Finkel, E. J. (2004). Too proud to let go: Narcissistic entitlement as a barrier to forgiveness. *Journal of Personality and Social Psychology, 87*(6), 894-912. doi:10.1037/0022-3514.87.6.894

Exline, J. J., & Geyer, A. L. (2004). Perceptions of Humility: A Preliminary Study. *Self and Identity, 3*(2), 95-114. doi:10.1080/13576500342000077

Exline, J. J., Single, P. B., Lobel, M., & Geyer, A. L. (2004). Glowing praise and the envious gaze: Social dilemmas surrounding the public recognition of achievement. *Basic and Applied Social Psychology, 26*(2-3), 119-130. doi:10.1207/s15324834basp2602&3_2

Exline, J. J., & Zell, A. L. (2012). Who Doesn't Want to be Envied? Personality Correlates of Emotional Responses to Outperformance Scenarios. *Basic and Applied Social Psychology, 34*(3), 236-253. doi:10.1080/01973533.2012.674412

Eyring Iii, W. E., & Sobelman, S. (1996). Narcissism and birth order. *Psychological Reports, 78*(2), 403-406. doi:10.2466/pr0.1996.78.2.403

Falco, A., Girardi, D., Di Sipio, A., Calvo, V., Marogna, C., & Snir, R. (2020). Is narcissism associated with heavy work investment? The moderating role of workload in the relationship between narcissism, workaholism, and work engagement. *International Journal of Environmental Research and Public Health, 17*(13), 1-23. doi:10.3390/ijerph17134750

Falkenbach, D. M., Howe, J. R., & Falki, M. (2013). Using self-esteem to disaggregate psychopathy, narcissism, and aggression. *Personality and Individual Differences, 54*(7), 815-820. doi:10.1016/j.paid.2012.12.017

Falkenbach, D. M., McKinley, S. J., & Larson, F. R. R. (2017). Two Sides of the Same Coin: Psychopathy Case Studies From an Urban Police Department. *Journal of Forensic Psychology Research and Practice, 17*(5), 338-356. doi:10.1080/24732850.2017.1378860

Farwell, L., & Wohlwend-Lloyd, R. (1998). Narcissistic Processes: Optimistic Expectations, Favorable Self-Evaluations, and Self-Enhancing Attributions. *Journal of Personality, 66*(1), 65-83. doi:10.1111/1467-6494.00003

Farzand, M., Cerkez, Y., & Baysen, E. (2021). Effects of Self-Concept on Narcissism: Mediational Role of Perceived Parenting. *Frontiers in Psychology*, *12*, 674679. <https://doi.org/10.3389/fpsyg.2021.674679>

Fatfouta, R., & Schröder-Abé, M. (2018). Agentic to the core? Facets of narcissism and positive implicit self-views in the agentic domain. *Journal of Research in Personality, 74*, 78-82. doi:10.1016/j.jrp.2018.02.006

Fatfouta, R., & Heinze, P. E. (2023). Do bigger egos mean bigger presence? Facets of grandiose narcissism and mindfulness. *Current Psychology*, *42*(23), 19795-19807. <https://doi.org/10.1007/s12144-022-03115-w>

Fazekas, Z., & Hatemi, P. K. (2021). Narcissism in Political Participation. *Personality and Social Psychology Bulletin, 47*(3), 347-361. doi:10.1177/0146167220919212

Ferenczi, N., Marshall, T. C., & Bejanyan, K. (2017). Are sex differences in antisocial and prosocial Facebook use explained by narcissism and relational self-construal? *Computers in Human Behavior, 77*, 25-31. doi:10.1016/j.chb.2017.08.033

Filipe, A. T. d. N. (2016). *Narcissistic leaders and their effect on company performance: a study of the portuguese case.* Instituto Superior de Economia e Gestão,

Findley, D., & Ojanen, T. (2013). Agentic and Communal Goals in Early Adulthood: Associations with Narcissism, Empathy, and Perceptions of Self and Others. *Self and Identity, 12*(5), 504-526. doi:10.1080/15298868.2012.694660

Finkel, E. J., Campbell, W. K., Buffardi, L. E., Kumashiro, M., & Rusbult, C. E. (2009). The metamorphosis of narcissus: Communal activation promotes relationship commitment among narcissists. *Personality and Social Psychology Bulletin, 35*(10), 1271-1284. doi:10.1177/0146167209340904

Fisher, M. L., & Exline, J. J. (2006). Self-forgiveness versus excusing: The roles of remorse, effort, and acceptance of responsibility. *Self and Identity, 5*(2), 127-146. doi:10.1080/15298860600586123

Foster, J. D., & Campbell, W. K. (2005). Narcissism and resistance to doubts about romantic partners. *Journal of Research in Personality, 39*(5), 550-557. doi:10.1016/j.jrp.2004.11.001

Foster, J. D., & Campbell, W. K. (2007). Are there such things as "Narcissists" in social psychology? A taxometric analysis of the Narcissistic Personality Inventory. *Personality and Individual Differences, 43*(6), 1321-1332. doi:10.1016/j.paid.2007.04.003

Foster, J. D., Campbell, W. K., & Twenge, J. M. (2003). Individual differences in narcissism: Inflated self-views across the lifespan and around the world. *Journal of Research in Personality, 37*(6), 469-486. doi:10.1016/S0092-6566(03)00026-6

Foster, J. D., Jonason, P. K., Shrira, I., Keith Campbell, W., Shiverdecker, L. K., & Varner, S. C. (2014). What do you get when you make somebody else's partner your own? An analysis of relationships formed via mate poaching. *Journal of Research in Personality, 52*, 78-90. doi:10.1016/j.jrp.2014.07.008

Foster, J. D., McCain, J. L., Hibberts, M. F., Brunell, A. B., & Burke Johnson, R. (2015). The Grandiose Narcissism Scale: A Global and Facet-Level Measure of Grandiose Narcissism. *Personality and Individual Differences, 73*, 12-16. doi:10.1016/j.paid.2014.08.042

Foster, J. D., Misra, T. A., & Reidy, D. E. (2009). Narcissists are approach-oriented toward their money and their friends. *Journal of Research in Personality, 43*(5), 764-769. doi:10.1016/j.jrp.2009.05.005

Foster, J. D., Raley, J. R., & Isen, J. D. (2020). Further evidence that only children are not more narcissistic than individuals with siblings. *Personality and Individual Differences, 161*. doi:10.1016/j.paid.2020.109977

Foster, J. D., Reidy, D. E., Misra, T. A., & Goff, J. S. (2011). Narcissism and stock market investing: Correlates and consequences of cocksure investing. *Personality and Individual Differences, 50*(6), 816-821. doi:10.1016/j.paid.2011.01.002

Foster, J. D., Shenesey, J. W., & Goff, J. S. (2009). Why do narcissists take more risks? Testing the roles of perceived risks and benefits of risky behaviors. *Personality and Individual Differences, 47*(8), 885-889. doi:10.1016/j.paid.2009.07.008

Foster, J. D., Shiverdecker, L. K., & Turner, I. N. (2016). What Does the Narcissistic Personality Inventory Measure Across the Total Score Continuum? *Current Psychology, 35*(2), 207-219. doi:10.1007/s12144-016-9407-5

Foster, J. D., Shrira, I., & Campbell, W. K. (2006). Theoretical models of narcissism, sexuality, and relationship commitment. *Journal of Social and Personal Relationships, 23*(3), 367-386. doi:10.1177/0265407506064204

Foster, J. D., & Trimm Iv, R. F. (2008). On being eager and uninhibited: Narcissism and approach-avoidance motivation. *Personality and Social Psychology Bulletin, 34*(7), 1004-1017. doi:10.1177/0146167208316688

Foti, G. (2012). *An Investigation of Narcissism and Self-Regulation as Predictors of Aggression.* University of Guelph,

Foti, R. J., Bray, B. C., Thompson, N. J., & Allgood, S. F. (2012). Know thy self, know thy leader: Contributions of a pattern-oriented approach to examining leader perceptions. *Leadership Quarterly, 23*(4), 702-717. doi:10.1016/j.leaqua.2012.03.007

Fox, F. R., Smith, M. B., & Webster, B. D. (2023). Take your ethics and shove it! Narcissists' angry responses to ethical leadership. *Personality and Individual Differences*, *204*. <https://doi.org/10.1016/j.paid.2022.112032>

Freis, S. D., & Allen, W. M. (2019). Decision-making among the narcissistic subtypes: If I can’t benefit, then who should? *Current Psychology*. doi:10.1007/s12144-019-00558-6

Freis, S. D., & Brunell, A. B. (2021). Effects of narcissism in essential workers during COVID-19. *Personality and Individual Differences, 171*. doi:10.1016/j.paid.2020.110533

Freis, S. D., & Hansen-Brown, A. A. (2021). Justifications of entitlement in grandiose and vulnerable narcissism: The roles of injustice and superiority. *Personality and Individual Differences, 168*. doi:10.1016/j.paid.2020.110345

Freis, S. D., & Brunell, A. B. (2022). Narcissistic motivations to help during the COVID-19 quarantine. *Personality and Individual Differences*, *194*, 111623. <https://doi.org/10.1016/j.paid.2022.111623>

Freund, V. L., Peeters, F., Meesters, C., Geschwind, N., Lemmens, L., Bernstein, D. P., & Lobbestael, J. (2022). Narcissistic traits and compassion: Embracing oneself while devoiding others. *Frontiers in Psychology*, *13*, 914270. <https://doi.org/10.3389/fpsyg.2022.914270>

Fukunishi, I. (1994). SOCIAL DESIRABILITY AND ALEXITHYMIA. *Psychological Reports, 75*(2), 835-838. doi:10.2466/pr0.1994.75.2.835

Fukunishi, I., & Aoki, T. (1996). Relationship between narcissistic tendencies and psychiatric conditions in patients undergoing physical rehabilitation. *Perceptual and Motor Skills, 85*(2), 403-408. doi:10.2466/pms.1996.83.2.403

Fukunishi, I., Nakagawa, T., Nakamura, H., Li, K., Hua, Z. Q., & Kratz, T. S. (1996). Relationships between type a behavior, narcissism, and maternal closeness for college students in Japan, the United States of America, and the People's Republic of China. *Psychological Reports, 78*(3 PART 1), 939-944. doi:10.2466/pr0.1996.78.3.939

Fukushima, O., & Hosoe, T. (2011). Narcissism, variability in self-concept, and well-being. *Journal of Research in Personality, 45*(6), 568-575. doi:10.1016/j.jrp.2011.07.002

Fukushima, O., Iwasaki, K., Aoki, S. i., & Kikuchi, J. (2007). Parents' narcissism and aggression against children: When parents attribute misfortune to their child. *Research in Social Psychology, 22*(1), 1-11. Retrieved from https://www.scopus.com/inward/record.uri?eid=2-s2.0-70350179558&partnerID=40&md5=c760922a1fb19ddfc9224f4bdb28113e

Fulford, D., Johnson, S. L., & Carver, C. S. (2008). Commonalities and differences in characteristics of persons at risk for narcissism and mania. *Journal of Research in Personality, 42*(6), 1427-1438. doi:10.1016/j.jrp.2008.06.002

Gabriel, M. T., Critelli, J. W., & Ee, J. S. (1994). Narcissistic Illusions in Self‐Evaluations of Intelligence and Attractiveness. *Journal of Personality, 62*(1), 143-155. doi:10.1111/j.1467-6494.1994.tb00798.x

Geary, C., March, E., & Grieve, R. (2021). Insta-identity: Dark personality traits as predictors of authentic self-presentation on Instagram. *Telematics and Informatics*, *63*, Article 101669. <https://doi.org/10.1016/j.tele.2021.101669>

Garcia, D., & Sikström, S. (2014). The dark side of Facebook: Semantic representations of status updates predict the Dark Triad of personality. *Personality and Individual Differences, 67*, 69-74. doi:10.1016/j.paid.2013.10.001

Garduño, J. M. G., & Sotres, J. F. C. (1998). The measurement of narcissistic personality. *Psicothema, 10*(3), 725-735. Retrieved from https://www.scopus.com/inward/record.uri?eid=2-s2.0-3142571297&partnerID=40&md5=324f473ced81263233cfbb012bfc0330

Gawad, N., Ibrahim, A. M., Duffy, M., Raiche, I., & Nessim, C. (2019). Going Beyond the Numerical Scoresheet: Identifying Maladaptive Narcissistic Traits in Residency Applicants. *Journal of Surgical Education, 76*(1), 65-76. doi:10.1016/j.jsurg.2018.06.024

Gazit, T., & Aharony, N. (2018). Factors explaining participation in WhatsApp groups: an exploratory study. *Aslib Journal of Information Management, 70*(4), 390-413. doi:10.1108/AJIM-03-2018-0053

Gerbasi, M. E., & Prentice, D. A. (2013). The self-and other-interest inventory. *Journal of Personality and Social Psychology, 105*(3), 495-514. doi:10.1037/a0033483

Gerstenberg, F. X. R., Imhoff, R., Banse, R., & Schmitt, M. (2014). Discrepancies between implicit and explicit self-concepts of intelligence: Relations to modesty, narcissism, and achievement motivation. *Frontiers in Psychology, 5*(FEB). doi:10.3389/fpsyg.2014.00085

Geukes, K., Breil, S. M., Hutteman, R., Nestler, S., Küfner, A. C. P., & Back, M. D. (2019). Explaining the longitudinal interplay of personality and social relationships in the laboratory and in the field: The PILS and the CONNECT study. *PLoS ONE, 14*(1). doi:10.1371/journal.pone.0210424

Ghinassi, S., Fioravanti, G., & Casale, S. (2023). Is shame responsible for maladaptive daydreaming among grandiose and vulnerable narcissists? A general population study. *Personality and Individual Differences*, *206*. <https://doi.org/10.1016/j.paid.2023.112122>

Ghorbani, N., Watson, P. J., Hamzavy, F., & Weathington, B. L. (2010). Self-knowledge and narcissism in Iranians: Relationships with empathy and self-esteem. *Current Psychology, 29*(2), 135-143. doi:10.1007/s12144-010-9079-5

Giacomin, M., & Jordan, C. H. (2014). Down-Regulating Narcissistic Tendencies: Communal Focus Reduces State Narcissism. *Personality and Social Psychology Bulletin, 40*(4), 488-500. doi:10.1177/0146167213516635

Giacomin, M., & Jordan, C. H. (2015). Validating Power Makes Communal Narcissists Less Communal. *Self and Identity, 14*(5), 583-601. doi:10.1080/15298868.2015.1031820

Giacomin, M., & Jordan, C. H. (2016a). Self-focused and feeling fine: Assessing state narcissism and its relation to well-being. *Journal of Research in Personality, 63*, 12-21. doi:10.1016/j.jrp.2016.04.009

Giacomin, M., & Jordan, C. H. (2016b). The Wax and Wane of Narcissism: Grandiose Narcissism as a Process or State. *Journal of Personality, 84*(2), 154-164. doi:10.1111/jopy.12148

Giacomin, M., & Rule, N. O. (2019). Eyebrows cue grandiose narcissism. *Journal of Personality, 87*(2), 373-385. doi:10.1111/jopy.12396

Giacomin, M., Brinton, C., & Rule, N. O. (2022). Narcissistic individuals exhibit poor recognition memory. *Journal of Personality*, *90*(5), 675-689. <https://doi.org/10.1111/jopy.12690>

Giacomin, M., Johnston, E. E., & Legge, E. L. G. (2023). Exploring narcissism and human- and animal-centered empathy in pet owners. *Frontiers in Psychology*, *14*. <https://doi.org/10.3389/fpsyg.2023.1087049>

Giambatista, R. C., & Hoover, J. D. (2018). Narcissism and teamwork skill acquisition in management education. *Psychologist-Manager Journal, 21*(1), 55-74. doi:10.1037/mgr0000064

Gino, F., & Ariely, D. (2012). The dark side of creativity: Original thinkers can be more dishonest. *Journal of Personality and Social Psychology, 102*(3), 445-459. doi:10.1037/a0026406

Giordano, C., Salerno, L., Pavia, L., Cavani, P., Lo Coco, G., Tosto, C., & Di Blasi, M. (2019). Magic mirror on the wall: Selfie-related behavior as mediator of the relationship between narcissism and problematic smartphone use. *Clinical Neuropsychiatry, 16*(5-6), 197-205. doi:10.36131/clinicalnpsych2019050602

Given-Wilson, Z., McIlwain, D., & Warburton, W. (2011). Meta-cognitive and interpersonal difficulties in overt and covert narcissism. *Personality and Individual Differences, 50*(7), 1000-1005. doi:10.1016/j.paid.2011.01.014

Glover, N., Miller, J. D., Lynam, D. R., Crego, C., & Widiger, T. A. (2012). The five-factor narcissism inventory: A five-factor measure of narcissistic personality traits. *Journal of Personality Assessment, 94*(5), 500-512. doi:10.1080/00223891.2012.670680

Gökçearslan, Ş., Yildiz Durak, H., Berikan, B., & Saritepeci, M. (2021). Smartphone Addiction, Loneliness, Narcissistic Personality, and Family Belonging Among University Students: A Path Analysis. *Social Science Quarterly*. doi:10.1111/ssqu.12949

Golec de Zavala, A., Peker, M., Guerra, R., & Baran, T. (2016). Collective Narcissism Predicts Hypersensitivity to In-group Insult and Direct and Indirect Retaliatory Intergroup Hostility. *European Journal of Personality, 30*(6), 532-551. doi:10.1002/per.2067

Golec de Zavala, A., Förster, C., Ziegler, M., Nalberczak-Skóra, M., Ciesielski, P., & Mazurkiewicz, M. (2024). The shape of the change: Cumulative and incremental changes in daily mood during mobile-app-supported mindfulness training. *Applied Psychology: Health and Well-Being*. <https://doi.org/10.1111/aphw.12518>

Gómez-Leal, R., Megías-Robles, A., Gutiérrez-Cobo, M. J., Cabello, R., Fernández-Abascal, E. G., & Fernández-Berrocal, P. (2019). Relationship between the Dark Triad and depressive symptoms. *PeerJ, 2019*(11). doi:10.7717/peerj.8120

Goncalo, J. A., Flynn, F. J., & Kim, S. H. (2010). Are two narcissists better than one? the link between narcissism, perceived creativity, and creative performance. *Personality and Social Psychology Bulletin, 36*(11), 1484-1495. doi:10.1177/0146167210385109

Goodwin, R., Graham, J., & Diekmann, K. A. (2020). Good intentions aren't good enough: Moral courage in opposing sexual harassment. *Journal of Experimental Social Psychology, 86*. doi:10.1016/j.jesp.2019.103894

Gordon, K. H., & Dombeck, J. J. (2010). The associations between two facets of narcissism and eating disorder symptoms. *Eating Behaviors, 11*(4), 288-292. doi:10.1016/j.eatbeh.2010.08.004

Górnik-Durose, M. E. (2020). Materialism and Well-Being Revisited: The Impact of Personality. *Journal of Happiness Studies, 21*(1), 305-326. doi:10.1007/s10902-019-00089-8

Górnik-Durose, M. E. (2021). Regulatory Focus in Materialists and Its Consequences for Their Well-Being. *Journal of Happiness Studies*. doi:10.1007/s10902-020-00349-y

Górnik-Durose, M. E., & Pyszkowska, A. (2020). Personality matters – Explaining the link between materialism and well-being in young adults. *Personality and Individual Differences, 163*. doi:10.1016/j.paid.2020.110075

Gottheim, C. P. (2010). *Self-esteem, self-compassion, defensive self-esteem, and related features of narcissism as predictors of aggression.* State University of New York at Albany, Retrieved from <http://oatd.org/oatd/record?record=oai\:pqdtoai.proquest.com\:3387118&q=%20%28NPI%29%20OR%20%28Narcissistic%20AND%20Personality%20AND%20Inventory%29%20>

Grapsas, S., Brummelman, E., Dufner, M., & Denissen, J. J. A. (2022). Affective Contingencies of Narcissism. *Journal of Personality and Social Psychology*, *123*(2), 444-462. <https://doi.org/10.1037/pspp0000406>

Gray, C. J., Carter, N. T., & Sears, K. L. (2017). The UWBQ-I: An Adaption and Validation of a Measure of Instigated Incivility. *Journal of Business and Psychology, 32*(1), 21-39. doi:10.1007/s10869-015-9433-6

Greaves, C. E., Zacher, H., McKenna, B., & Rooney, D. (2014). Wisdom and narcissism as predictors of transformational leadership. *Leadership and Organization Development Journal, 35*(4), 335-358. doi:10.1108/LODJ-07-2012-0092

Greenberger, E., Lessard, J., Chen, C., & Farruggia, S. P. (2008). Self-entitled college students: Contributions of personality, parenting, and motivational factors. *Journal of Youth and Adolescence, 37*(10), 1193-1204. doi:10.1007/s10964-008-9284-9

Greenier, K. D. (2018). The Relationship Between Personality and Schadenfreude in Hypothetical Versus Live Situations. *Psychological Reports, 121*(3), 445-458. doi:10.1177/0033294117745562

Greenwood, D., McCutcheon, L. E., Collisson, B., & Wong, M. (2018). What's fame got to do with it? Clarifying links among celebrity attitudes, fame appeal, and narcissistic subtypes. *Personality and Individual Differences, 131*, 238-243. doi:10.1016/j.paid.2018.04.032

Grieve, R., March, E., & Watkinson, J. (2020). Inauthentic self-presentation on facebook as a function of vulnerable narcissism and lower self-esteem. *Computers in Human Behavior, 102*, 144-150. doi:10.1016/j.chb.2019.08.020

Grijalva, E., & Newman, D. A. (2015). Narcissism and counterproductive work behavior (CWB): Meta-analysis and consideration of collectivist culture, big five personality, and narcissism’s facet structure. *Applied Psychology, 64*(1), 93-126. doi:10.1111/apps.12025

Große Deters, F., Mehl, M. R., & Eid, M. (2014). Narcissistic power poster? On the relationship between narcissism and status updating activity on Facebook. *Journal of Research in Personality, 53*, 165-174. doi:10.1016/j.jrp.2014.10.004

Grover, S., & Furnham, A. (2021a). The Dark Triad, emotional intelligence, self-monitoring and executive coach effectiveness and satisfaction. *Coaching*. doi:10.1080/17521882.2021.1881575

Grover, S., & Furnham, A. (2021b). Does emotional intelligence and resilience moderate the relationship between the Dark Triad and personal and work burnout? *Personality and Individual Differences, 169*. doi:10.1016/j.paid.2020.109979

Grover, S., & Furnham, A. (2021c). The moderating effects of emotional stability on the relationship between the Dark Triad and different measures of risk-taking. *Personality and Individual Differences, 171*. doi:10.1016/j.paid.2020.110450

Grover, S., & Furnham, A. (2021d). Personality at home vs. work: Does framing for work increase predictive validity of the Dark Triad on work outcomes? *Personality and Individual Differences, 169*. doi:10.1016/j.paid.2020.109848

Grubbs, J. B., Exline, J. J., Keith Campbell, W., Twenge, J. M., & Pargament, K. I. (2018). God owes me: The role of divine entitlement in predicting struggles with a deity. *Psychology of Religion and Spirituality, 10*(4), 356-367. doi:10.1037/rel0000147

Grubbs, J. B., Exline, J. J., McCain, J., Keith Campbell, W., & Twenge, J. M. (2019). Emerging adult reactions to labeling regarding age-group differences in narcissism and entitlement. *PLoS ONE, 14*(5). doi:10.1371/journal.pone.0215637

Gu, Y., He, N., & Zhao, G. (2013). Attentional bias for performance-related words in individuals with narcissism. *Personality and Individual Differences, 55*(6), 671-675. doi:10.1016/j.paid.2013.05.009

Gu, W., & Watts, L. L. (2021). Are narcissistic hiring managers more susceptible to candidate flattery? A within-subjects experimental simulation. *Personality and Individual Differences*, *177*. <https://doi.org/10.1016/j.paid.2021.110803>

Gu, Z. B., He, Y. Y., Liu, L., Liang, Y., Huang, L. L., Dang, J. N., Wei, C., Liu, Z., & Su, Q. (2021). How does narcissism influence corruption? The moderating role of boredom. *Personality and Individual Differences*, *183*, 10. <https://doi.org/10.1016/j.paid.2021.111149>

Guedes, M. J., & da Conceição Gonçalves, V. (2019). Top managers' characteristics as causal explanations for self-reported performance. *Journal of Business Research, 101*, 869-874. doi:10.1016/j.jbusres.2018.11.014

Guedes, M. J. C. (2017). Mirror, mirror on the wall, am I the greatest performer of all? Narcissism and self-reported and objective performance. *Personality and Individual Differences, 108*, 182-185. doi:10.1016/j.paid.2016.12.030

Gurtman, M. B. (1992). CONSTRUCT-VALIDITY OF INTERPERSONAL PERSONALITY MEASURES - THE INTERPERSONAL CIRCUMPLEX AS A NOMOLOGICAL NET. *Journal of Personality and Social Psychology, 63*(1), 105-118. doi:10.1037/0022-3514.63.1.105

Gustafson, S. B., & Ritzer, D. R. (1995). The dark side of normal: A psychopathy‐linked pattern called aberrant self‐promotion. *European Journal of Personality, 9*(3), 147-183. doi:10.1002/per.2410090302

Ha, S. B., Lee, S., Byun, G., & Dai, Y. (2020). Leader narcissism and subordinate change-oriented organizational citizenship behavior: Overall justice as a moderator. *Social Behavior and Personality, 48*(7). doi:10.2224/sbp.9330

Halper, L. R., & Rios, K. (2019). Feeling Powerful but Incompetent: Fear of Negative Evaluation Predicts Men’s Sexual Harassment of Subordinates. *Sex Roles, 80*(5-6), 247-261. doi:10.1007/s11199-018-0938-0

Ham, C., Seybert, N., & Wang, S. (2018). Narcissism is a bad sign: CEO signature size, investment, and performance. *Review of Accounting Studies, 23*(1), 234-264. doi:10.1007/s11142-017-9427-x

Hample, D., & Irions, A. L. (2015). Arguing to Display Identity. *Argumentation, 29*(4), 389-416. doi:10.1007/s10503-015-9351-9

Hamstra, M. R. W., Schreurs, B., Jawahar, I. M., Laurijssen, L. M., & Hünermund, P. (2021). Manager narcissism and employee silence: A socio-analytic theory perspective. *Journal of Occupational and Organizational Psychology, 94*(1), 29-54. doi:10.1111/joop.12337

Hanke, S., Rohmann, E., & Förster, J. (2018). Relationships between narcissistic grandiosity, narcissistic vulnerability, regulatory focus, regulatory mode, and life-satisfaction: Data from two surveys. *Data in Brief, 21*, 861-865. doi:10.1016/j.dib.2018.10.042

Hansen-Brown, A. A., & Freis, S. D. (2021). Assuming the worst: Hostile attribution bias in vulnerable narcissists. *Self and Identity, 20*(2), 152-164. doi:10.1080/15298868.2019.1609574

Hanson, S. K., Valentine, S. R., & Shultz, P. L. (2023). Bright or dark, vain or villainous? How entrepreneurial fitness, Machiavellianism and narcissism relate to entrepreneurial intention. *Journal of Management & Organization*, 24. <https://doi.org/10.1017/jmo.2023.15>

Hardaker, M., & Tsakanikos, E. (2021). Early information processing in narcissism: Heightened sensitivity to negative but not positive evaluative attributes. *Personality and Individual Differences*, *168*. <https://doi.org/10.1016/j.paid.2020.110386>

Hardaker, M., & Tsakanikos, E. (2022). Can self-affirmation reduce narcissistic hypervigilance to self-threat? *Personality and Individual Differences*, *199*. <https://doi.org/10.1016/j.paid.2022.111835>

Harhoff, N., Reinhardt, N., Reinhard, M. A., & Mayer, M. (2023). Agentic and communal narcissism in predicting different types of lies in romantic relationships. *Frontiers in Psychology*, *14*, 10, Article 1146732. <https://doi.org/10.3389/fpsyg.2023.1146732>

Harmanci, B. S., & Okray, Z. (2021). Body Image, Muscle Dysmorphia and Narcissistic Characteristics of Bodybuilder Males in TRNC. *Cyprus Turkish Journal of Psychiatry and Psychology*, *3*(2), 82-90. <https://doi.org/10.35365/ctjpp.21.2.09>

Harris, M. W., Byrne, K. A., Liu, Y., & Anaraky, R. G. (2023). The cost of giving: Examining the relationship between narcissistic, self-sacrificing, and empathetic traits on effortful versus effortless prosocial behavior. *Journal of Research in Personality*, *102*, Article 104320. <https://doi.org/10.1016/j.jrp.2022.104320>

Hart, C. M., Bush-Evans, R. D., Hepper, E. G., & Hickman, H. M. (2017). The children of narcissus: Insights into narcissists' parenting styles. *Personality and Individual Differences, 117*, 249-254. doi:10.1016/j.paid.2017.06.019

Hart, C. M., Sedikides, C., Wildschut, T., Arndt, J., Routledge, C., & Vingerhoets, A. J. J. M. (2011). Nostalgic recollections of high and low narcissists. *Journal of Research in Personality, 45*(2), 238-242. doi:10.1016/j.jrp.2011.01.002

Hart, W., Adams, J., Burton, K. A., & Tortoriello, G. K. (2017). Narcissism and self-presentation: Profiling grandiose and vulnerable Narcissists' self-presentation tactic use. *Personality and Individual Differences, 104*, 48-57. doi:10.1016/j.paid.2016.06.062

Hart, W., & Adams, J. M. (2014). Are narcissists more accepting of others' narcissistic traits? *Personality and Individual Differences, 64*, 163-167. doi:10.1016/j.paid.2014.02.038

Hart, W., Adams, J. M., & Alex Burton, K. (2016). Narcissistic for the People: Narcissists and Non-narcissists Disagree about How to Make a Good Impression. *Personality and Individual Differences, 91*, 69-73. doi:10.1016/j.paid.2015.11.045

Hart, W., Kinrade, C., & Breeden, C. J. (2020). Revisiting narcissism and contingent self-esteem: A test of the psychodynamic mask model. *Personality and Individual Differences, 162*. doi:10.1016/j.paid.2020.110026

Hart, W., Richardson, K., Breeden, C. J., Tortoriello, G. K., & Kinrade, C. (2021). Exploring the interactive role of narcissism and self-esteem on self-presentation. *Self and Identity*. doi:10.1080/15298868.2021.1884593

Hart, W., Richardson, K., Tortoriello, G., & Tullett, A. (2017). Strategically out of control: A self-presentational conceptualization of narcissism and low self-control. *Personality and Individual Differences, 114*, 103-107. doi:10.1016/j.paid.2017.03.046

Hart, W., Richardson, K., & Tortoriello, G. K. (2018). Meet your public relations team: People with dark traits may help you manage your image. *Personality and Individual Differences, 134*, 164-173. doi:10.1016/j.paid.2018.06.019

Hart, W., Richardson, K., & Tortoriello, G. K. (2021). Revisiting the Interactive Effect of Narcissism and Self-Esteem on Responses to Ego Threat: Distinguishing Between Assertiveness and Intent to Harm. *Journal of Interpersonal Violence, 36*(7-8), 3662-3687. doi:10.1177/0886260518777551

Hart, W., Richardson, K., Tortoriello, G. K., & Breeden, C. J. (2019). Revisiting profiles and profile comparisons of grandiose and vulnerable narcissism on self-presentation tactic use. *Personality and Individual Differences, 151*. doi:10.1016/j.paid.2019.109523

Hart, W., Tortoriello, G. K., & Breeden, C. J. (2020). Entitled Due to Deprivation vs. Superiority: Evidence That Unidimensional Entitlement Scales Blend Distinct Entitlement Rationales across Psychological Dimensions. *Journal of Personality Assessment, 102*(6), 781-791. doi:10.1080/00223891.2019.1674319

Hart, W., Tortoriello, G. K., & Richardson, K. (2019). Feeling good about oneself heightens, not hinders, the goodness in narcissism. *Current Psychology, 38*(5), 1399-1408. doi:10.1007/s12144-018-9993-5

Hart, W., Tortoriello, G. K., & Richardson, K. (2020a). Deprived and Grandiose Explanations for Psychological Entitlement: Implications for Theory and Measurement. *Journal of Personality Assessment, 102*(4), 488-498. doi:10.1080/00223891.2019.1565573

Hart, W., Tortoriello, G. K., & Richardson, K. (2020b). Why are narcissistic people cold? A cognitive account emphasizing the perceived momentousness of successes and failures. *Personality and Individual Differences, 153*. doi:10.1016/j.paid.2019.109596

Hart, W., Tortoriello, G. K., & Richardson, K. (2021). Provoked Narcissistic Aggression: Examining the Role of De-Escalated and Escalated Provocations. *Journal of Interpersonal Violence, 36*(9-10), 4832-4853. doi:10.1177/0886260518789901

Hart, W., Tortoriello, G. K., Richardson, K., & Adams, J. (2018). "S/he's Taken": Effects of Grandiose and Vulnerable Narcissism on Responses to Relationship Threats from Rivals. *Journal of Individual Differences, 39*(4), 212-219. doi:10.1027/1614-0001/a000266

Hart, W., Tortoriello, G. K., Richardson, K., & Adams, J. M. (2021). Detecting the elusive narcissistic reactivity phenomenon: The case for a mechanistic focus. *Self and Identity, 20*(2), 311-322. doi:10.1080/15298868.2019.1634144

Hart, W., Tortoriello, G. K., Richardson, K., & Breeden, C. J. (2019). Profiles and profile comparisons between Dark Triad constructs on self-presentation tactic usage and tactic beliefs. *Journal of Personality, 87*(3), 501-517. doi:10.1111/jopy.12411

Hart, W., Tortoriello, G. K., Richardson, K., & Breeden, C. J. (2020). Substantive vs. superficial self-enhancement: Differentiating narcissism constructs from self-esteem following failure. *Personality and Individual Differences, 152*. doi:10.1016/j.paid.2019.109560

Hart, J., & Stekler, N. (2022). Does personality "Trump" ideology? narcissism predicts support for Trump via ideological tendencies. *Journal of Social Psychology*, *162*(3), 386-392. <https://doi.org/10.1080/00224545.2021.1944035>

Hart, W., Richardson, K., Breeden, C. J., Tortoriello, G. K., & Kinrade, C. (2022). Exploring the interactive role of narcissism and self-esteem on self-presentation [Article]. *Self and Identity*, *21*(2), 137-162. <https://doi.org/10.1080/15298868.2021.1884593>

Hart, W., Cease, C. K., Lambert, J. T., Witt, D. E., Hall, B. T., & Breeden, C. J. (2024). The Capone hypothesis: Do antagonistic individuals view themselves as more good than evil? *Personality and Individual Differences*, *216*, 6, Article 112426. <https://doi.org/10.1016/j.paid.2023.112426>

Hatemi, P. K., & Fazekas, Z. (2018). Narcissism and Political Orientations. *American Journal of Political Science, 62*(4), 873-888. doi:10.1111/ajps.12380

He, Y. (2023). Too Close to the Ego: Narcissists' Affective Reaction to Advertising Depends on Its Relevance to Self-Image. *Journal of Advertising*, *52*(2), 264-278. <https://doi.org/10.1080/00913367.2022.2027298>

Heinze, P. E., Fatfouta, R., & Schröder-Abé, M. (2020). Validation of an implicit measure of antagonistic narcissism. *Journal of Research in Personality, 88*. doi:10.1016/j.jrp.2020.103993

Hennessy, D. A. (2016). Are narcissists really angrier drivers? An examination of state driving anger among narcissistic subtypes. *Transportation Research Part F: Traffic Psychology and Behaviour, 42*, 267-275. doi:10.1016/j.trf.2016.06.025

Hepper, E. G., Gramzow, R. H., & Sedikides, C. (2010). Individual Differences in Self-Enhancement and Self-Protection Strategies: An Integrative Analysis. *Journal of Personality, 78*(2), 781-814. doi:10.1111/j.1467-6494.2010.00633.x

Hepper, E. G., Hart, C. M., Meek, R., Cisek, S., & Sedikides, C. (2014). Narcissism and empathy in young offenders and non-offenders. *European Journal of Personality, 28*(2), 201-210. doi:10.1002/per.1939

Hepper, E. G., Hart, C. M., & Sedikides, C. (2014). Moving Narcissus: Can Narcissists Be Empathic? *Personality and Social Psychology Bulletin, 40*(9), 1079-1091. doi:10.1177/0146167214535812

Hepper, E. G., Sedikides, C., & Cai, H. (2013). Self-Enhancement and Self-Protection Strategies in China: Cultural Expressions of a Fundamental Human Motive. *Journal of Cross-Cultural Psychology, 44*(1), 5-23. doi:10.1177/0022022111428515

Hermann, A., & Fuller, R. (2017). Trait Narcissism and Contemporary Religious Trends. *Archive for the Psychology of Religion, 39*(2), 99-117. doi:10.1163/15736121-12341339

Hermann, A. D., Teutemacher, A. M., & Lehtman, M. J. (2015). Revisiting the unmitigated approach model of narcissism: Replication and extension. *Journal of Research in Personality, 55*, 41-45. doi:10.1016/j.jrp.2014.12.002

Heym, N., Kibowski, F., Bloxsom, C. A. J., Blanchard, A., Harper, A., Wallace, L., . . . Sumich, A. (2021). The Dark Empath: Characterising dark traits in the presence of empathy. *Personality and Individual Differences, 169*. doi:10.1016/j.paid.2020.110172

Hibbard, S. (1993). Adult children of alcoholics: Narcissism, shame, and the differential effects of paternal and maternal alcoholism. *Psychiatry (New York), 56*(2), 153-162. doi:10.1521/00332747.1993.11024629

Hickman, S. E., Watson, P. J., & Morris, R. J. (1996). Optimism, pessimism, and the complexity of narcissism. *Personality and Individual Differences, 20*(4), 521-525. doi:10.1016/0191-8869(95)00223-5

Highhouse, S., Brooks, M. E., & Wang, Y. (2016). Status Seeking and Manipulative Self-presentation. *International Journal of Selection and Assessment, 24*(4), 352-361. doi:10.1111/ijsa.12153

Hill, E. M. (2016). The role of narcissism in health-risk and health-protective behaviors. *Journal of Health Psychology, 21*(9), 2021-2032. doi:10.1177/1359105315569858

Hill, P. L., & Roberts, B. W. (2012). Narcissism, well-being, and observer-rated personality across the lifespan. *Social Psychological and Personality Science, 3*(2), 216-223. doi:10.1177/1948550611415867

Hill, E. M., Martin, J. D., & Lego, J. E. (2021). College students' engagement in drunkorexia: Examining the role of sociocultural attitudes toward appearance, narcissism, and Greek affiliation. *Current Psychology*, *40*(9), 4468-4478. <https://doi.org/10.1007/s12144-019-00382-y>

Hill, R. W., & Yousey, G. P. (1998). Adaptive and maladaptive narcissism among university faculty, clergy, politicians, and librarians. *Current Psychology, 17*(2-3), 163-169. doi:10.1007/s12144-998-1003-x

Hirschi, A., & Jaensch, V. K. (2015). Narcissism and career success: Occupational self-efficacy and career engagement as mediators. *Personality and Individual Differences, 77*, 205-208. doi:10.1016/j.paid.2015.01.002

Hoskin, R. A., Blair, K. L., & Holmberg, D. (2023). Femmephobia Is a Uniquely Powerful Predictor of Anti-Gay Behavior. *Archives of Sexual Behavior*, 14. <https://doi.org/10.1007/s10508-023-02704-5>

Hodson, G., Hogg, S. M., & MacInnis, C. C. (2009). The role of "dark personalities" (narcissism, Machiavellianism, psychopathy), Big Five personality factors, and ideology in explaining prejudice. *Journal of Research in Personality, 43*(4), 686-690. doi:10.1016/j.jrp.2009.02.005

Hoffman, B. J., Strang, S. E., Kuhnert, K. W., Campbell, W. K., Kennedy, C. L., & Lopilato, A. C. (2013). Leader narcissism and ethical context: Effects on ethical leadership and leader effectiveness. *Journal of Leadership and Organizational Studies, 20*(1), 25-37. doi:10.1177/1548051812465891

Holtzman, N. S. (2011). Facing a psychopath: Detecting the dark triad from emotionally-neutral faces, using prototypes from the Personality Faceaurus. *Journal of Research in Personality, 45*(6), 648-654. doi:10.1016/j.jrp.2011.09.002

Holtzman, N. S., Vazire, S., & Mehl, M. R. (2010). Sounds like a narcissist: Behavioral manifestations of narcissism in everyday life. *Journal of Research in Personality, 44*(4), 478-484. doi:10.1016/j.jrp.2010.06.001

Horton, R. S. (2021). Parenthood, subjective well-being, and the moderating effects of parent narcissism. *Journal of Individual Differences, 42*(2), 57-63. doi:10.1027/1614-0001/a000329

Horton, R. S., Bleau, G., & Drwecki, B. (2006). Parenting narcissus: What are the links between parenting and narcissism? *Journal of Personality, 74*(2), 345-376. doi:10.1111/j.1467-6494.2005.00378.x

Horton, R. S., & Sedikides, C. (2009). Narcissistic responding to ego threat: When the status of the evaluator matters. *Journal of Personality, 77*(5), 1493-1526. doi:10.1111/j.1467-6494.2009.00590.x

Horton, R. S., & Tritch, T. (2014). Clarifying the links between grandiose narcissism and parenting. *Journal of Psychology: Interdisciplinary and Applied, 148*(2), 133-143. doi:10.1080/00223980.2012.752337

Horvath, S., & Morf, C. C. (2010). To be grandiose or not to be worthless: Different routes to self-enhancement for narcissism and self-esteem. *Journal of Research in Personality, 44*(5), 585-592. doi:10.1016/j.jrp.2010.07.002

Houston, J. M., Luchner, A., Davidson, A. J., Gonzalez, J., Steigerwald, N., & Leftwich, C. (2021). The Bright and Dark Aspects of Grit in the Pursuit of Success. *Psychological Reports, 124*(2), 839-861. doi:10.1177/0033294120907316

Howell, A. J., Dopko, R. L., Turowski, J. B., & Buro, K. (2011). The disposition to apologize. *Personality and Individual Differences, 51*(4), 509-514. doi:10.1016/j.paid.2011.05.009

Howes, S. S., Kausel, E. E., Jackson, A. T., & Reb, J. (2020). When and Why Narcissists Exhibit Greater Hindsight Bias and Less Perceived Learning. *Journal of Management, 46*(8), 1498-1528. doi:10.1177/0149206320929421

Huang, L., Krasikova, D. V., & Harms, P. D. (2020). Avoiding or embracing social relationships? A conservation of resources perspective of leader narcissism, leader–member exchange differentiation, and follower voice. *Journal of Organizational Behavior, 41*(1), 77-92. doi:10.1002/job.2423

Huang, X., Chen, L., Xu, E., Lu, F., & Tam, K. C. (2020). Shadow of the Prince: Parent-incumbents’ Coercive Control over Child-successors in Family Organizations. *Administrative Science Quarterly, 65*(3), 710-750. doi:10.1177/0001839219870449

Hyun, N. K., Park, Y., & Park, S. W. (2016). Narcissism and gift giving: Not every gift is for others. *Personality and Individual Differences, 96*, 47-51. doi:10.1016/j.paid.2016.02.057

Iliescu, D., Ispas, D., Sulea, C., & Ilie, A. (2015). Vocational fit and counterproductive work behaviors: A self-regulation perspective. *Journal of Applied Psychology, 100*(1), 21-39. doi:10.1037/a0036652

Imamoglu, A. H., & Batigun, A. D. (2020). The assessment of the relationship between narcissism, perceived parental rearing styles, and defense mechanisms. *Dusunen Adam-Journal of Psychiatry and Neurological Sciences, 33*(4), 388-401. doi:10.14744/DAJPNS.2020.00107

Irwin, H. J. (1995). Codependence, narcissism, and childhood trauma. *Journal of Clinical Psychology, 51*(5), 658-665. doi:10.1002/1097-4679(199509)51:5<658::AID-JCLP2270510511>3.0.CO;2-N

Jablonska, M. R., & Zajdel, R. (2020). The Dark Triad Traits and Problematic Internet Use: Their Structure and Relations. *Polish Sociological Review*(212), 477-495. doi:10.26412/psr212.06

Jain, N., Kowalski, C. M., Johnson, L. K., & Saklofske, D. H. (2023). Dark thoughts, dark deeds: An exploration of the relationship between the Dark Tetrad and aggression. *Current Psychology*, *42*(21), 18017-18032. <https://doi.org/10.1007/s12144-022-02993-4>

Jackson, D. L., McLellan, C. M., Frey, M. P., & Rauti, C. M. (2020). Are There Types of Academically Entitled Students? A Cluster Analysis. *Canadian Journal of Education, 43*(4), 1008-1034. Retrieved from https://www.scopus.com/inward/record.uri?eid=2-s2.0-85098772222&partnerID=40&md5=2f516b3f5110fc46a022c235d3e0641b

Jackson, L. A., Ervin, K. S., & Hodge, C. N. (1992). Narcissism and body image. *Journal of Research in Personality, 26*(4), 357-370. doi:10.1016/0092-6566(92)90065-C

Jahani, H. J. G., Ehsanikenari, A., & Sharif, A. S. (2018). Role of self-efficacy and negative perfectionism in the prediction of procrastination of narcissistic personality: A study on non-clinical subjects. *Emerging Science Journal, 2*(6), 388-399. doi:10.28991/esj-2018-01158

Jakobwitz, S., & Egan, V. (2006). The dark triad and normal personality traits. *Personality and Individual Differences, 40*(2), 331-339. doi:10.1016/j.paid.2005.07.006

Jauk, E., Breyer, D., Kanske, P., & Wakabayashi, A. (2021). Narcissism in independent and interdependent cultures. *Personality and Individual Differences, 177*. doi:10.1016/j.paid.2021.110716

Jauk, E., Neubauer, A. C., Mairunteregger, T., Pemp, S., Sieber, K. P., & Rauthmann, J. F. (2016). How Alluring Are Dark Personalities? The Dark Triad and Attractiveness in Speed Dating. *European Journal of Personality, 30*(2), 125-138. doi:10.1002/per.2040

Jauk, E., Weigle, E., Lehmann, K., Benedek, M., & Neubauer, A. C. (2017). The relationship between Grandiose and Vulnerable (Hypersensitive) Narcissism. *Frontiers in Psychology, 8*(SEP). doi:10.3389/fpsyg.2017.01600

Jin, S. V., & Ryu, E. (2018). “The Paradox of Narcissus and Echo in the Instagram Pond” in Light of the Selfie Culture from Freudian Evolutionary Psychology: Self-Loving and Confident but Lonely. *Journal of Broadcasting and Electronic Media, 62*(4), 554-577. doi:10.1080/08838151.2018.1474881

Jiyoung, L., & Gabsook, K. (2013). Effects of narcissistic personality traits and interpersonal relationship tendencies of art therapists on their countertransference management ability. *Arts in Psychotherapy, 40*(3), 298-305. doi:10.1016/j.aip.2013.05.008

Ji, Y. T., Liu, H. Y., Liu, S. M., Xu, M. Y., & Lin, Z. X. (2023). Are narcissists more creative? Only if we believe it: How narcissism can relate to creativity. *Frontiers in Psychology*, *13*, 12. <https://doi.org/10.3389/fpsyg.2022.1091770>

Jiang, L., Cui, A. P., & Shan, J. (2022). Quiet versus loud luxury: the influence of overt and covert narcissism on young Chinese and US luxury consumers' preferences? *International Marketing Review*, *39*(2), 309-334. <https://doi.org/10.1108/imr-02-2021-0093>

Jiwen Song, L., Ni, D., Zhu, J., Zheng, X., & Zhu, L. (2024). Servant Leadership and Employee Gratitude: The Moderating Role of Employee Narcissism. *Journal of Business and Psychology*. <https://doi.org/10.1007/s10869-023-09928-1>

Johnson, E. N., Lowe, D. J., & Reckers, P. M. J. (2021). The influence of auditor narcissism and moral disengagement on risk assessments of a narcissistic client CFO. *Journal of Accounting and Public Policy*. doi:10.1016/j.jaccpubpol.2021.106826

Jonason, P. K., & Davis, M. D. (2018). A gender role view of the Dark Triad traits. *Personality and Individual Differences, 125*, 102-105. doi:10.1016/j.paid.2018.01.004

Jonason, P. K., Jones, A., & Lyons, M. (2013). Creatures of the night: Chronotypes and the Dark Triad traits. *Personality and Individual Differences, 55*(5), 538-541. doi:10.1016/j.paid.2013.05.001

Jonason, P. K., & Kavanagh, P. (2010). The dark side of love: Love styles and the Dark Triad. *Personality and Individual Differences, 49*(6), 606-610. doi:10.1016/j.paid.2010.05.030

Jonason, P. K., Kavanagh, P. S., Webster, G. D., & Fitzgerald, D. (2011). Comparing the measured and latent dark triad: Are three measures better than one? *Journal of Methods and Measurement in the Social Sciences, 2*(1), 28-44.

Jonason, P. K., Koenig, B. L., & Tost, J. (2010). Living a Fast Life: The Dark Triad and Life History Theory. *Human Nature, 21*(4), 428-442. doi:10.1007/s12110-010-9102-4

Jonason, P. K., Li, N. P., & Buss, D. M. (2010). The costs and benefits of the Dark Triad: Implications for mate poaching and mate retention tactics. *Personality and Individual Differences, 48*(4), 373-378. doi:10.1016/j.paid.2009.11.003

Jonason, P. K., Li, N. P., Webster, G. D., & Schmitt, D. P. (2009). The Dark Triad: Facilitating a short-term mating strategy in men. *European Journal of Personality, 23*(1), 5-18. doi:10.1002/per.698

Jonason, P. K., Luevano, V. X., & Adams, H. M. (2012). How the Dark Triad traits predict relationship choices. *Personality and Individual Differences, 53*(3), 180-184. doi:10.1016/j.paid.2012.03.007

Jonason, P. K., Lyons, M., Bethell, E. J., & Ross, R. (2013). Different routes to limited empathy in the sexes: Examining the links between the Dark Triad and empathy. *Personality and Individual Differences, 54*(5), 572-576. doi:10.1016/j.paid.2012.11.009

Jonason, P. K., & Tost, J. (2010). I just cannot control myself: The Dark Triad and self-control. *Personality and Individual Differences, 49*(6), 611-615. doi:10.1016/j.paid.2010.05.031

Jonason, P. K., & Zeigler-Hill, V. (2018). The fundamental social motives that characterize dark personality traits. *Personality and Individual Differences, 132*, 98-107. doi:10.1016/j.paid.2018.05.031

Jonason, P. K., Zeigler-Hill, V., & Okan, C. (2017). Good v. evil: Predicting sinning with dark personality traits and moral foundations. *Personality and Individual Differences, 104*, 180-185. doi:10.1016/j.paid.2016.08.002

Jones, B. D., Woodman, T., Barlow, M., & Roberts, R. (2017). The darker side of personality: Narcissism predicts moral disengagement and antisocial behavior in sport. *Sport Psychologist, 31*(2), 109-116. doi:10.1123/tsp.2016-0007

Jones, D. N. (2013). What's mine is mine and what's yours is mine: The Dark Triad and gambling with your neighbor's money. *Journal of Research in Personality, 47*(5), 563-571. doi:10.1016/j.jrp.2013.04.005

Jones, D. N. (2014). Risk in the face of retribution: Psychopathic individuals persist in financial misbehavior among the Dark Triad. *Personality and Individual Differences, 67*, 109-113. doi:10.1016/j.paid.2014.01.030

Jones, D. N., & de Roos, M. S. (2017a). Differential Reproductive Behavior Patterns Among the Dark Triad. *Evolutionary Psychological Science, 3*(1), 10-19. doi:10.1007/s40806-016-0070-8

Jones, D. N., & De Roos, M. S. (2017b). Machiavellian flexibility in negative mate retention. *Personal Relationships, 24*(2), 265-279. doi:10.1111/pere.12181

Jones, D. N., & Figueredo, A. J. (2013). The Core of Darkness: Uncovering the Heart of the Dark Triad. *European Journal of Personality, 27*(6), 521-531. doi:10.1002/per.1893

Jones, D. N., & Olderbak, S. G. (2014). The Associations Among Dark Personalities and Sexual Tactics Across Different Scenarios. *Journal of Interpersonal Violence, 29*(6), 1050-1070. doi:10.1177/0886260513506053

Jones, D. N., & Paulhus, D. L. (2014). Introducing the Short Dark Triad (SD3): A Brief Measure of Dark Personality Traits. *Assessment, 21*(1), 28-41. doi:10.1177/1073191113514105

Jones, D. N., & Weiser, D. A. (2014). Differential infidelity patterns among the Dark Triad. *Personality and Individual Differences, 57*, 20-24. doi:10.1016/j.paid.2013.09.007

Jones, L. L., & Brunell, A. B. (2014). Clever and crude but not kind: Narcissism, self-esteem, and the self-reference effect. *Memory, 22*(4), 307-322. doi:10.1080/09658211.2013.778999

Jones, L. L., Norville, G. A., & Wright, A. M. (2017). Narcissism, self-esteem, and the phenomenology of autobiographical memories. *Memory, 25*(6), 800-815. doi:10.1080/09658211.2016.1223848

Jordan, D. G., Winer, E. S., Zeigler-Hill, V., & Marcus, D. K. (2022). A Network Approach to Understanding Narcissistic Grandiosity via the Narcissistic Admiration and Rivalry Questionnaire and the Narcissistic Personality Inventory. *Self and Identity*, *21*(6), 710-737. <https://doi.org/10.1080/15298868.2021.1944298>

Joubert, C. E. (1986). SOCIAL INTEREST, LONELINESS, AND NARCISSISM. *Psychological Reports, 58*(3), 870-870. doi:10.2466/pr0.1986.58.3.870

Joubert, C. E. (1989). THE FAMOUS SAYINGS TEST - SEX-DIFFERENCES AND SOME CORRELATIONS WITH OTHER VARIABLES. *Psychological Reports, 64*(3), 763-766. doi:10.2466/pr0.1989.64.3.763

Joubert, C. E. (1992). ANTECEDENTS OF NARCISSISM AND PSYCHOLOGICAL REACTANCE AS INDICATED BY COLLEGE-STUDENTS RETROSPECTIVE REPORTS OF THEIR PARENTS BEHAVIORS. *Psychological Reports, 70*(3), 1111-1115. doi:10.2466/PR0.70.4.1111-1115

Joubert, C. E. (1995). ASSOCIATIONS OF SOCIAL PERSONALITY-FACTORS WITH PERSONAL HABITS. *Psychological Reports, 76*(3), 1315-1321. doi:10.2466/pr0.1995.76.3c.1315

Joubert, C. E. (1998). Narcissism, need for power, and social interest. *Psychological Reports, 82*(2), 701-702. doi:10.2466/pr0.1998.82.2.701

Ju, C., Ji, M., Lan, J., & You, X. (2017). Narcissistic personality and risk perception among Chinese aviators: The mediating role of promotion focus. *International Journal of Psychology, 52*, 1-8. doi:10.1002/ijop.12243

Judd, J. D. (2012). *Latter-day Saint Young Adults, Narcissism, and Religiosity.* Brigham Young University, Retrieved from http://oatd.org/oatd/record?record=oai\:scholarsarchive.byu.edu\:etd-4316&q=%20%28NPI%29%20OR%20%28Narcissistic%20AND%20Personality%20AND%20Inventory%29%20

Judge, T. A., LePine, J. A., & Rich, B. L. (2006). Loving yourself abundantly: Relationship of the narcissistic personality to self- and other perceptions of workplace deviance, leadership, and task and contextual performance. *Journal of Applied Psychology, 91*(4), 762-776. doi:10.1037/0021-9010.91.4.762

Jung, S., & Jamieson, L. (2012). AN EXPLORATORY EXAMINATION OF OBSESSIVE, SCHIZOTYPAL, AND NARCISSISTIC TRAITS AMONG SEXUAL OFFENDERS. *Applied Psychology in Criminal Justice, 8*(1), 1-14.

Kalemi, G., Michopoulos, I., Efstathiou, V., Konstantopoulou, F., Tsaklakidou, D., Gournellis, R., & Douzenis, A. (2019). Narcissism but not criminality is associated with aggression in women: A study among female prisoners and women without a criminal record. *Frontiers in Psychiatry, 10*(FEB). doi:10.3389/fpsyt.2019.00021

Kalliopuska, M. (2008). Personality variables related to shyness. *Psychological Reports, 102*(1), 40-42. doi:10.2466/PR0.102.1.40-42

Kalowski, P., Szymaniak, K., & Maciantowicz, O. (2021). Exploring the Links Between Trait Anger, Self-Reported Sarcasm Use, and Narcissism. *Advances in Cognitive Psychology*, *17*(4), 261-273. <https://doi.org/10.5709/acp-0335-6>

Kanabar, J., & Fletcher, L. (2020). When does being in a talent pool reap benefits? The moderating role of narcissism. *Human Resource Development International*. doi:10.1080/13678868.2020.1840846

Kanabar, J., & Fletcher, L. (2022). When does being in a talent pool reap benefits? The moderating role of narcissism. *Human Resource Development International*, *25*(4), 415-432. <https://doi.org/10.1080/13678868.2020.1840846>

Kanbur, E. (2018). EFFECTS OF NARCISSISM ON ORGANIZATIONAL DISSENT. *Journal of Organizational Behavior Research, 3*(1), 169-181.

Kansi, J. (2003). The Narcissistic Personality Inventory: Applicability in a Swedish population sample. *Scandinavian Journal of Psychology, 44*(5), 441-448. doi:10.1046/j.1467-9450.2003.00365.x

Kapidzic, S. (2013). Narcissism as a predictor of motivations behind facebook profile picture selection. *Cyberpsychology, Behavior, and Social Networking, 16*(1), 14-19. doi:10.1089/cyber.2012.0143

Kapidzic, S., & Martins, N. (2015). Mirroring the Media: The Relationship Between Media Consumption, Media Internalization, and Profile Picture Characteristics on Facebook. *Journal of Broadcasting and Electronic Media, 59*(2), 278-297. doi:10.1080/08838151.2015.1029127

Kapoor, H. (2015). The Creative Side of the Dark Triad. *Creativity Research Journal, 27*(1), 58-67. doi:10.1080/10400419.2014.961775

Karandikar, S., Kapoor, H., Fernandes, S., & Jonason, P. K. (2019). Predicting moral decision-making with dark personalities and moral values. *Personality and Individual Differences, 140*, 70-75. doi:10.1016/j.paid.2018.03.048

Kardum, I., Hudek-Knezevic, J., Mehic, N., & Pilek, M. (2018). The effects of similarity in the dark triad traits on the relationship quality in dating couples. *Personality and Individual Differences, 131*, 38-44. doi:10.1016/j.paid.2018.04.020

Kardum, I., Hudek-Knezevic, J., & Mehic, N. (2022). Similarity indices of the Dark Triad traits based on self and partner-reports: Evidence from variable-centered and couple-centered approaches. *Personality and Individual Differences*, *193*. <https://doi.org/10.1016/j.paid.2022.111626>

Kausel, E. E., Culbertson, S. S., Leiva, P. I., Slaughter, J. E., & Jackson, A. T. (2015). Too arrogant for their own good? Why and when narcissists dismiss advice. *Organizational Behavior and Human Decision Processes, 131*, 33-50. doi:10.1016/j.obhdp.2015.07.006

Kavanagh, P. S., Signal, T. D., & Taylor, N. (2013). The Dark Triad and animal cruelty: Dark personalities, dark attitudes, and dark behaviors. *Personality and Individual Differences, 55*(6), 666-670. doi:10.1016/j.paid.2013.05.019

Keener, A. (2020). An examination of psychological characteristics and their relationship to academic entitlement among millennial and nonmillennial college students. *Psychology in the Schools, 57*(4), 572-582. doi:10.1002/pits.22338

Keiller, S. W. (2010). Male Narcissism and Attitudes Toward Heterosexual Women and Men, Lesbian Women, and Gay Men: Hostility toward Heterosexual Women Most of All. *Sex Roles, 63*(7-8), 530-541. doi:10.1007/s11199-010-9837-8

Kertzman, S., Kagan, A., Vainder, M., Lapidus, R., & Weizman, A. (2022). Relationship between smoking, narcissism, and impulsiveness among young women. *Bmc Psychology*, *10*(1), 7. <https://doi.org/10.1186/s40359-022-00809-5>

Kesenheimer, J. S., & Greitemeyer, T. (2021). Greenwash yourself: The relationship between communal and agentic narcissism and pro-environmental behavior. *Journal of Environmental Psychology, 75*. doi:10.1016/j.jenvp.2021.101621

Khodabakhsh, M. R., & Besharat, M. A. (2011). *Mediation effect of narcissism on the relationship between emotional intelligence and the quality of interpersonal relationships*.

Kiliç, N., & Eryilmaz, A. (2019). Investigating narcissistic personality traits in the context of positive Psychotherapy. *Noropsikiyatri Arsivi, 56*(1), 40-46. doi:10.29399/npa.22794

Kim, E., Lee, J. A., Sung, Y., & Choi, S. M. (2016). Predicting selfie-posting behavior on social networking sites: An extension of theory of planned behavior. *Computers in Human Behavior, 62*, 116-123. doi:10.1016/j.chb.2016.03.078

Kim, H., & Barry, C. T. (2021). The moderating effect of intolerance of uncertainty on the relation between narcissism and aggression. *Current Psychology*. doi:10.1007/s12144-021-01542-9

Kim, J. W., & Chock, T. M. (2017). Personality traits and psychological motivations predicting selfie posting behaviors on social networking sites. *Telematics and Informatics, 34*(5), 560-571. doi:10.1016/j.tele.2016.11.006

Kinrade, C., Hart, W., & Lambert, J. T. (2022). Mental toughness, rather than self-esteem, moderates effects of grandiose narcissism on aggression under conditions of ego-threat. *Personality and Individual Differences*, *189*, Article 111518. <https://doi.org/10.1016/j.paid.2022.111518>

Klein, V., Reininger, K. M., Briken, P., & Turner, D. (2020). Sexual narcissism and its association with sexual and well-being outcomes. *Personality and Individual Differences, 152*. doi:10.1016/j.paid.2019.109557

Kleitman, S., Hui, J. S. W., & Jiang, Y. (2019). Confidence to spare: individual differences in cognitive and metacognitive arrogance and competence. *Metacognition and Learning, 14*(3), 479-508. doi:10.1007/s11409-019-09210-x

Kokkinos, C. M., Baltzidis, E., & Xynogala, D. (2016). Prevalence and personality correlates of Facebook bullying among university undergraduates. *Computers in Human Behavior, 55*, 840-850. doi:10.1016/j.chb.2015.10.017

Koladich, S. J., & Atkinson, B. E. (2016). The dark triad and relationship preferences: A replication and extension. *Personality and Individual Differences, 94*, 253-255. doi:10.1016/j.paid.2016.01.023

Kong, D. T. (2015). Narcissists' negative perception of their counterpart's competence and benevolence and their own reduced trust in a negotiation context. *Personality and Individual Differences, 74*, 196-201. doi:10.1016/j.paid.2014.10.015

Konrath, S., Bushman, B. J., & Campbell, W. K. (2006). Attenuating the link between threatened egotism and aggression. *Psychological Science, 17*(11), 995-1001. doi:10.1111/j.1467-9280.2006.01818.x

Konrath, S., Bushman, B. J., & Grove, T. (2009). Seeing my world in a million little pieces: Narcissism, self-construal, and cognitive-perceptual style. *Journal of Personality, 77*(4), 1197-1228. doi:10.1111/j.1467-6494.2009.00579.x

Konrath, S., Ho, M. H., & Zarins, S. (2016). The Strategic Helper: Narcissism and Prosocial Motives and Behaviors. *Current Psychology, 35*(2), 182-194. doi:10.1007/s12144-016-9417-3

Körner, R., Heydasch, T., & Schütz, A. (2021). It's All about Power: Validation of Trait and State Versions of the German Personal Sense of Power Scale. *European Journal of Psychological Assessment*. doi:10.1027/1015-5759/a000642

Körner, R., Heydasch, T., & Schütz, A. (2022). Dominance and Prestige as Self-Concept Facets. *Journal of Personality Assessment*, *105*(5), 590-609. <https://doi.org/10.1080/00223891.2022.2137028>

Kowal, M., & Sorokowski, P. (2022). Sex Differences in Physical Attractiveness Investments: Overlooked Side of Masculinity. *International Journal of Environmental Research and Public Health*, *19*(7), 9. <https://doi.org/10.3390/ijerph19073842>

Kowalski, C. M., Rogoza, R., Vernon, P. A., & Schermer, J. A. (2018). The Dark Triad and the self-presentation variables of socially desirable responding and self-monitoring. *Personality and Individual Differences, 120*, 234-237. doi:10.1016/j.paid.2017.09.007

Kowalski, C. M., Vernon, P. A., & Schermer, J. A. (2017). Vocational interests and dark personality: Are there dark career choices? *Personality and Individual Differences, 104*, 43-47. doi:10.1016/j.paid.2016.07.029

Kowalski, C. M., Vernon, P. A., & Schermer, J. A. (2019). The Dark Triad and facets of personality. *Current Psychology*. doi:10.1007/s12144-019-00518-0

Krafcik, D. (2011). *Words from the wise| A qualitative and quantitative study of nominated exemplars of wisdom.* Institute of Transpersonal Psychology, Retrieved from http://oatd.org/oatd/record?record=oai\:pqdtoai.proquest.com\:3457971&q=%20%28NPI%29%20OR%20%28Narcissistic%20AND%20Personality%20AND%20Inventory%29%20

Kraft, L., Zimmermann, J., Schmukle, S. C., Czarna, A. Z., Sekerdej, M., & Dufner, M. (2023). Who Gets the Credit for Success and the Blame for Failure? On the Links Between Narcissism and Self- and Group-Serving Biases. *European Journal of Personality*, 18. <https://doi.org/10.1177/08902070231199366>

Kray, L. J., Kennedy, J. A., & Rosenblum, M. (2022). Who do they think they are?: A social-cognitive account of gender differences in social sexual identity and behavior at work. *Organizational Behavior and Human Decision Processes*, *172*, 24. <https://doi.org/10.1016/j.obhdp.2022.104186>

Ksinan, A. J., Mališ, J., & Vazsonyi, A. T. (2019). Swiping away the moments that make up a dull day: Narcissism, boredom, and compulsive smartphone use. *Current Psychology*. doi:10.1007/s12144-019-00228-7

Ksinan, A. J., & Vazsonyi, A. T. (2016). Narcissism, Internet, and social relations: A study of two tales. *Personality and Individual Differences, 94*, 118-123. doi:10.1016/j.paid.2016.01.016

Kühne, R., & Opree, S. J. (2019). Validating the Short Material Values Scale for Children for Use Across the Lifespan. *Child and Youth Care Forum, 48*(3), 339-359. doi:10.1007/s10566-018-9482-x

Kwan, V. S. Y., Kuang, L. L., & Hui, N. H. H. (2009). Identifying the sources of self-esteem: The mixed medley of benevolence, merit, and bias. *Self and Identity, 8*(2-3), 176-195. doi:10.1080/15298860802504874

La Sala, L., Skues, J., & Grant, S. (2014). Personality traits and Facebook use: The combined/interactive effect of extraversion, neuroticism and conscientiousness. *Social Networking, 3*(05), 211.

Labuschagne, N. (1996). *Narcissism, family of origin, and career self-efficacy: a comparative study of university students.*

Lachowicz-Tabaczek, K., Lewandowska, B., Kochan-Wójcik, M., Andrzejewska, B. E., & Juszkiewicz, A. (2019). Grandiose and Vulnerable Narcissism as Predictors of the Tendency to Objectify Other People. *Current Psychology*. doi:10.1007/s12144-019-00569-3

Lachowicz-Tabaczek, K., & Kozlowska, M. A. (2021). Being others-oriented during the pandemic: Individual differences in the sense of responsibility for collective health as a robust predictor of compliance with the COVID-19 containing measures. *Personality and Individual Differences*, *183*, 9. <https://doi.org/10.1016/j.paid.2021.111138>

Ladd, E. R., Welsh, M. C., Vitulli, W. F., Labbé, E. E., & Law, J. G. (1997). Narcissism and causal attribution. *Psychological Reports, 80*(1), 171-178. doi:10.2466/pr0.1997.80.1.171

Lakey, C. E., Rose, P., Campbell, W. K., & Goodie, A. S. (2008). Probing the link between narcissism and gambling: The mediating role of judgment and decision-making biases. *Journal of Behavioral Decision Making, 21*(2), 113-137. doi:10.1002/bdm.582

Lambert, A., & Desmond, J. (2013). Loyal now, but not forever! A study of narcissism and male consumer-brand relationships. *Psychology and Marketing, 30*(8), 690-706. doi:10.1002/mar.20638

Lamborn, P. B. (2015). *The Relationship Between Narcissism, Sex Roles and Ratings of Video Recorded Job Interviews.* Citeseer,

Lambourn-Kavcic, B., & Day, H. D. (1995). Characteristics of male partners of adult female incest survivors. *Journal of Contemporary Psychotherapy, 25*(4), 387-398. doi:10.1007/BF02306570

Lamkin, J., Clifton, A., Campbell, W. K., & Miller, J. D. (2014). An examination of the perceptions of social network characteristics associated with grandiose and vulnerable narcissism. *Personality Disorders: Theory, Research, and Treatment, 5*(2), 137-145. doi:10.1037/per0000024

Lamkin, J., Maples-Keller, J. L., & Miller, J. D. (2018). How Likable Are Personality Disorder and General Personality Traits to Those Who Possess Them? *Journal of Personality, 86*(2), 173-185. doi:10.1111/jopy.12302

Lämmle, L., Nussbeck, F. W., & Ziegler, M. (2021). Hello from the Other Side: Can We Perceive Others’ Darkness? Observers’ Accuracy of the Dark Triad. *Journal of Personality Assessment, 103*(1), 106-119. doi:10.1080/00223891.2019.1683020

Lämmle, L., & Ziegler, M. (2021). Our vulnerable dark side—two laboratory approaches. *International Journal of Environmental Research and Public Health*, *18*(8), Article 3941. <https://doi.org/10.3390/ijerph18083941>

Lange, J., Redford, L., & Crusius, J. (2019). A Status-Seeking Account of Psychological Entitlement. *Personality and Social Psychology Bulletin, 45*(7), 1113-1128. doi:10.1177/0146167218808501

Lang, Y., Zhang, H. Y., Liu, J. L., & Zhang, X. Y. (2022). Narcissistic Enough to Challenge: The Effect of Narcissism on Change-Oriented Organizational Citizenship Behavior. *Frontiers in Psychology*, *12*, 17. <https://doi.org/10.3389/fpsyg.2021.792818>

Lannin, D. G., Guyll, M., Krizan, Z., Madon, S., & Cornish, M. (2014). When are grandiose and vulnerable narcissists least helpful? *Personality and Individual Differences, 56*(1), 127-132. doi:10.1016/j.paid.2013.08.035

Laracy, N. (2016). *The Downside of Wealth| Toward a Psychopathology of Money Accumulation.* The Chicago School of Professional Psychology, Retrieved from <http://oatd.org/oatd/record?record=oai\:pqdtoai.proquest.com\:10142664&q=%20%28NPI%29%20OR%20%28Narcissistic%20AND%20Personality%20AND%20Inventory%29%20>

Lapierre-Bédard, A., Rancourt-Tremblay, Z., Painchaud, J. A. M., Allard, M., & Descôteaux, J. (2023). Narcissism and concern: The mediating role of explicit motives. *Current Psychology*, *42*(25), 21693-21703. <https://doi.org/10.1007/s12144-022-03261-1>

Lasson, C., & Raynal, P. (2021). Personality profiles in young adults with orthorexic eating behaviors. *Eating and Weight Disorders*. doi:10.1007/s40519-021-01124-5

Lauder, C., & March, E. (2023). Catching the catfish: Exploring gender and the Dark Tetrad of personality as predictors of catfishing perpetration. *Computers in Human Behavior*, *140*, 9. <https://doi.org/10.1016/j.chb.2022.107599>

Laverdière, O., Gamache, D., Morin, A. J. S., & Diguer, L. (2020). French adaptation of the Mini-IPIP: A short measure of the Big Five. *Revue Europeenne de Psychologie Appliquee, 70*(3). doi:10.1016/j.erap.2019.100512

Lavner, J. A., Lamkin, J., Miller, J. D., Campbell, W. K., & Karney, B. R. (2016). Narcissism and Newlywed Marriage: Partner Characteristics and Marital Trajectories. *Personality Disorders: Theory, Research, and Treatment, 7*(2), 169-179. doi:10.1037/per0000137

Law, H., & Falkenbach, D. M. (2018). Hostile Attribution Bias as a Mediator of the Relationships of Psychopathy and Narcissism With Aggression. *International Journal of Offender Therapy and Comparative Criminology, 62*(11), 3355-3371. doi:10.1177/0306624X17742614

Lawrence, C. (2006). Measuring individual responses to aggression-triggering events: Development of the Situational Triggers of Aggressive Responses (STAR) scale. *Aggressive Behavior, 32*(3), 241-252. doi:10.1002/ab.20122

Le, B. M., & Impett, E. A. (2019). Parenting goal pursuit is linked to emotional well-being, relationship quality, and responsiveness. *Journal of Social and Personal Relationships, 36*(3), 879-904. doi:10.1177/0265407517747417

Le, T. N. (2005). A measure of immature love. *Individual Differences Research, 3*(2), 72-87. Retrieved from <https://www.scopus.com/inward/record.uri?eid=2-s2.0-44649178283&partnerID=40&md5=b68b50d138707d66b27d201403412e86>

Lee, T., Liu, C. H., Gan, B., & Yang, C. K. (2023). The double-edged sword of narcissism: influences of university students' grandiose and vulnerable narcissism on entrepreneurial intention. *Asia Pacific Journal of Management*, 36. <https://doi.org/10.1007/s10490-023-09934-0>

Leary, M. R., Tate, E. B., Adams, C. E., Allen, A. B., & Hancock, J. (2007). Self-compassion and reactions to unpleasant self-relevant events: The implications of treating oneself kindly. *Journal of Personality and Social Psychology, 92*(5), 887-904. doi:10.1037/0022-3514.92.5.887

Leask, Z. (1997). *Narcissism, physical self-efficacy and exercise addiction : a comparative study of runners and aerobics exercisers.* University of KwaZulu-Natal, Retrieved from http://oatd.org/oatd/record?record=handle\:10413\%2F5955&q=%20%28NPI%29%20OR%20%28Narcissistic%20AND%20Personality%20AND%20Inventory%29%20

Leckelt, M., Küfner, A. C. P., Nestler, S., & Back, M. D. (2015). Behavioral processes underlying the decline of narcissists' popularity over time. *Journal of Personality and Social Psychology, 109*(5), 856-871. doi:10.1037/pspp0000057

Lee, E., Choi, T. R., Lee, T., & Sung, Y. (2019). Using Instagram while "in a Relationship": The Roles of Narcissism and Self-Esteem. *Journal of Individual Differences, 40*(2), 111-117. doi:10.1027/1614-0001/a000282

Lee, J. A., & Sung, Y. (2016). Hide-and-seek: Narcissism and "Selfie"-related behavior. *Cyberpsychology, Behavior, and Social Networking, 19*(5), 347-351. doi:10.1089/cyber.2015.0486

Lee, K., & Ashton, M. C. (2005). Psychopathy, Machiavellianism, and Narcissism in the Five-Factor Model and the HEXACO model of personality structure. *Personality and Individual Differences, 38*(7), 1571-1582. doi:10.1016/j.paid.2004.09.016

Lee, S. Y., Gregg, A. P., & Park, S. H. (2013). The person in the purchase: Narcissistic consumers prefer products that positively distinguish them. *Journal of Personality and Social Psychology, 105*(2), 335-352. doi:10.1037/a0032703

Lehmann, V., Huis in't Veld, E. M. J., & Vingerhoets, A. J. J. M. (2013). The human and animal baby schema effect: Correlates of individual differences. *Behavioural Processes, 94*, 99-108. doi:10.1016/j.beproc.2013.01.001

Leonelli, S., & Masciarelli, F. (2019). *The antecedents of serial entrepreneurship: The role of entrepreneurs' narcissism and human capital*.

Lessard, J., Greenberger, E., Chen, C., & Farruggia, S. (2011). Are youths' feelings of entitlement always "bad"?: Evidence for a distinction between exploitive and non-exploitive dimensions of entitlement. *Journal of Adolescence, 34*(3), 521-529. doi:10.1016/j.adolescence.2010.05.014

Leung, L. (2013). Generational differences in content generation in social media: The roles of the gratifications sought and of narcissism. *Computers in Human Behavior, 29*(3), 997-1006. doi:10.1016/j.chb.2012.12.028

Leung, Y. K., Franken, I. H. A., & Thurik, A. R. (2020). Psychiatric symptoms and entrepreneurial intention: The role of the behavioral activation system. *Journal of Business Venturing Insights, 13*. doi:10.1016/j.jbvi.2019.e00153

Leunissen, J. M., Sedikides, C., & Wildschut, T. (2017). Why Narcissists are Unwilling to Apologize: The Role of Empathy and Guilt. *European Journal of Personality, 31*(4), 385-403. doi:10.1002/per.2110

Lindner, D., & Tantleff-Dunn, S. (2017). The Development and Psychometric Evaluation of the Self-Objectification Beliefs and Behaviors Scale. *Psychology of Women Quarterly, 41*(2), 254-272. doi:10.1177/0361684317692109

Linton, D. K., & Power, J. L. (2013). The personality traits of workplace bullies are often shared by their victims: Is there a dark side to victims? *Personality and Individual Differences, 54*(6), 738-743. doi:10.1016/j.paid.2012.11.026

Littrell, C. K., Grieve, F. G., Derryberry, W. P., Brausch, A., Parker, O. J., & Scott, J. N. (2021). Relationships Among Facets of Narcissism, Symptoms of Eating Disorders, and Symptoms of Muscle Dysmorphia. *Journal of Men's Studies, 29*(1), 118-128. doi:10.1177/1060826520918323

Littrell, S., Fugelsang, J., & Risko, E. F. (2020). Overconfidently underthinking: narcissism negatively predicts cognitive reflection. *Thinking and Reasoning, 26*(3), 352-380. doi:10.1080/13546783.2019.1633404

Liu, H., Ting-Ju Chiang, J., Fehr, R., Xu, M., & Wang, S. (2017). How do leaders react when treated unfairly? Leader narcissism and self-interested behavior in response to unfair treatment. *Journal of Applied Psychology, 102*(11), 1590-1599. doi:10.1037/apl0000237

Liu, X., Zheng, X., Li, N., Yu, Y., Harms, P. D., & Yang, J. (2021). Both a curse and a blessing? A social cognitive approach to the paradoxical effects of leader narcissism. *Human Relations*. doi:10.1177/00187267211015925

Liu, S. M., Liu, X. L., Wang, H., & Wang, Y. (2022a). Humble Leader Behavior and Its Effects on Performance at the Team and Individual Level: A Multi-Perspective Study. *Group & Organization Management*, *47*(5), 1008-1041. <https://doi.org/10.1177/10596011211024429>

Liu, X., Mao, J. Y., Zheng, X. M., Ni, D., & Harms, P. D. (2022b). When and why narcissism leads to taking charge? The roles of coworker narcissism and employee comparative identity. *Journal of Occupational and Organizational Psychology*, *95*(4), 758-787. <https://doi.org/10.1111/joop.12401>

Liu, X., Zhang, L., Gupta, A., Zheng, X. M., & Wu, C. Q. (2022c). Upper echelons and intra-organizational learning: How executive narcissism affects knowledge transfer among business units. *Strategic Management Journal*, *43*(11), 2351-2381. <https://doi.org/10.1002/smj.3406>

Liu, Z. H., Zhang, X. Y., Xu, H. Z., Deng, H., Li, J. J., & Lan, Y. Y. (2022d). The effect of i-deals on employees' unethical behavior during the COVID-19 pandemic: The roles of hubristic pride and grandiose narcissism. *Frontiers in Psychology*, *13*, 13. <https://doi.org/10.3389/fpsyg.2022.938864>

Liu, Z. Q., Zhou, R., Wei, L. H., Ouyang, X., & Zhou, K. (2023). How and when does leader narcissism hinder team radical creativity? The role of team information elaboration and inter-team competition. *Chinese Management Studies*, *17*(1), 46-63. <https://doi.org/10.1108/cms-08-2021-0347>

Lobbestael, J., Baumeister, R. F., Fiebig, T., & Eckel, L. A. (2014). The role of grandiose and vulnerable narcissism in self-reported and laboratory aggression and testosterone reactivity. *Personality and Individual Differences, 69*, 22-27. doi:10.1016/j.paid.2014.05.007

Lobbestael, J., & Freund, V. L. (2021). Humor in Dark Personalities: An Empirical Study on the Link Between Four Humor Styles and the Distinct Subfactors of Psychopathy and Narcissism. *Frontiers in Psychology, 12*. doi:10.3389/fpsyg.2021.548450

Lobene, E. V., Meade, A. W., & Pond, S. B. (2015). Perceived Overqualification: A Multi-Source Investigation of Psychological Predisposition and Contextual Triggers. *Journal of Psychology: Interdisciplinary and Applied, 149*(7), 684-710. doi:10.1080/00223980.2014.967654

Locke, K. D. (2009). Aggression, narcissism, self-esteem, and the attribution of desirable and humanizing traits to self versus others. *Journal of Research in Personality, 43*(1), 99-102. doi:10.1016/j.jrp.2008.10.003

Loeffler, L. A. K., Huebben, A. K., Radke, S., Habel, U., & Derntl, B. (2020). The Association Between Vulnerable/Grandiose Narcissism and Emotion Regulation. *Frontiers in Psychology, 11*. doi:10.3389/fpsyg.2020.519330

Long, A. D., & Herr, N. R. (2022). Narcissism, Empathy, and Rape Myth Acceptance Among Heterosexual College Males. *Archives of Sexual Behavior*, *51*(5), 2373-2383. <https://doi.org/10.1007/s10508-021-02256-6>

Lowe-Calverley, E., & Grieve, R. (2017). Web of deceit: Relationships between the Dark Triad, perceived ability to deceive and cyberloafing. *Cyberpsychology, 11*(2). doi:10.5817/CP2017-2-5

Luchner, A. F., Houston, J. M., Walker, C., & Alex Houston, M. (2011). Exploring the relationship between two forms of narcissism and competitiveness. *Personality and Individual Differences, 51*(6), 779-782. doi:10.1016/j.paid.2011.06.033

Luhtanen, R. K., & Crocker, J. (2005). Alcohol use in college students: Effects of level of self-esteem, narcissism, and contingencies of self-worth. *Psychology of Addictive Behaviors, 19*(1), 99-103. doi:10.1037/0893-164X.19.1.99

Lui, J. H. L., Chrysosferidis, J., Mousavi, S. Z., Barry, C. T., & Benson, C. S. (2019). Perceptions of Agentic and Communal Narcissism on Facebook. *Cyberpsychology, Behavior, and Social Networking, 22*(8), 529-534. doi:10.1089/cyber.2019.0135

Lull, R. B., & Dickinson, T. M. (2018). Does television cultivate narcissism? Relationships between television exposure, preferences for specific genres, and subclinical narcissism. *Psychology of Popular Media Culture, 7*(1), 47-60. doi:10.1037/ppm0000107

Lunt, C. A. (2016). " Getting Ahead" Versus" Getting Along": Examining the Role of Agency and Communion in Prejudice.

Luo, Y. L. L., Cai, H., Sedikides, C., & Song, H. (2014). Distinguishing communal narcissism from agentic narcissism: A behavior genetics analysis on the agency-communion model of narcissism. *Journal of Research in Personality, 49*(1), 52-58. doi:10.1016/j.jrp.2014.01.001

Lustman, M., Wiesenthal, D. L., & Flett, G. L. (2010). Narcissism and aggressive driving: Is an inflated view of the self a road Hazard? *Journal of Applied Social Psychology, 40*(6), 1423-1449. doi:10.1111/j.1559-1816.2010.00624.x

Lynch, B. P., Meisel, M. K., Campbell, W. K., & vanDellen, M. R. (2019). Promotion by others through social networks. *Self and Identity, 18*(6), 631-649. doi:10.1080/15298868.2018.1505652

Lyons, B. D., Bowling, N. A., & Burns, G. N. (2020). Accentuating dark triad behavior through low organizational commitment: a study on peer reporting. *Ethics and Behavior*. doi:10.1080/10508422.2020.1850286

Lyons, M., Blinkhorn, V., Collier, E. S., & Bertamini, M. (2019). Mine is bigger than yours! narcissism predicts biases in perceived head size. *Studia Psychologica, 61*(4), 245-257. doi:10.21909/sp.2019.04.786

Lyons, M., Houghton, E., Brewer, G., & O’Brien, F. (2020). The Dark Triad and Sexual Assertiveness Predict Sexual Coercion Differently in Men and Women. *Journal of Interpersonal Violence*. doi:10.1177/0886260520922346

Lyons, M., & Hughes, S. (2015). Feeling me, feeling you? Links between the Dark Triad and internal body awareness. *Personality and Individual Differences, 86*, 308-311. doi:10.1016/j.paid.2015.06.039

Lyons, M., & Rice, H. (2014). Thieves of time? Procrastination and the Dark Triad of personality. *Personality and Individual Differences, 61-62*, 34-37. doi:10.1016/j.paid.2014.01.002

Lyons, M. T., & Hughes, S. (2015). Malicious mouths? The Dark Triad and motivations for gossip. *Personality and Individual Differences, 78*, 1-4. doi:10.1016/j.paid.2015.01.009

Lyons, P. A., Kenworthy, J. B., & Popan, J. R. (2010). Ingroup identification and group-level narcissism as predictors of U.S. citizens' attitudes and behavior toward Arab immigrants. *Personality and Social Psychology Bulletin, 36*(9), 1267-1280. doi:10.1177/0146167210380604

Lyons, B. D., Bowling, N. A., & Burns, G. N. (2022a). Accentuating dark triad behavior through low organizational commitment: a study on peer reporting. *Ethics and Behavior*, *32*(1), 32-43. <https://doi.org/10.1080/10508422.2020.1850286>

Lyons, M., Houghton, E., Brewer, G., & O’Brien, F. (2022b). The Dark Triad and Sexual Assertiveness Predict Sexual Coercion Differently in Men and Women. *Journal of Interpersonal Violence*, *37*(7-8), NP4889-NP4904. <https://doi.org/10.1177/0886260520922346>

Lyvers, M., Narayanan, S. S., & Thorberg, F. A. (2019). Disordered social media use and risky drinking in young adults: Differential associations with addiction-linked traits. *Australian Journal of Psychology, 71*(3), 223-231. doi:10.1111/ajpy.12236

Lyvers, M., Salviani, A., Costan, S., & Thorberg, F. A. (2022). Alexithymia, narcissism and social anxiety in relation to social media and internet addiction symptoms. *International Journal of Psychology*, *57*(5), 606-612. <https://doi.org/10.1002/ijop.12840>

Maaß, U., Lämmle, L., Bensch, D., & Ziegler, M. (2016). Narcissists of a Feather Flock Together: Narcissism and the Similarity of Friends. *Personality and Social Psychology Bulletin, 42*(3), 366-384. doi:10.1177/0146167216629114

Macenczak, L. A., Campbell, S., Henley, A. B., & Campbell, W. K. (2016). Direct and interactive effects of narcissism and power on overconfidence. *Personality and Individual Differences, 91*, 113-122. doi:10.1016/j.paid.2015.11.053

Macenczak, L., Campbell, S., & Henley, A. (2021). Moderating impact of narcissism on the link between contextual variables and perceptions of fairness. *Personality and Individual Differences*, *181*, 8, Article 111015. <https://doi.org/10.1016/j.paid.2021.111015>

Maciantowicz, O., & Zajenkowski, M. (2020). Is narcissistic anger fueled by neuroticism? the relationship between grandiose and vulnerable narcissism, neuroticism, and trait anger. *Current Psychology, 39*(5), 1674-1681. doi:10.1007/s12144-018-9866-y

MacLaren, V. V., & Best, L. A. (2013). Disagreeable narcissism mediates an effect of BAS on addictive behaviors. *Personality and Individual Differences, 55*(2), 101-105. doi:10.1016/j.paid.2013.02.004

Maffly-Kipp, J., Truong, T. N., Edens, J. F., & Vess, M. (2023). Dark triad traits are associated with a weaker morally-good true self bias in self-perceptions. *Self and Identity*, *22*(5), 832-848. <https://doi.org/10.1080/15298868.2023.2169749>

Maftei, A., & Patrausanu, A. M. (2023). Digital Reflections: Narcissism, Stress, Social Media Addiction, and Nomophobia. *Journal of Psychology*, 14. <https://doi.org/10.1080/00223980.2023.2256453>

Mahadevan, N., Gregg, A. P., & Sedikides, C. (2020). The ups and downs of social life: Within-person variations in daily status and inclusion differentially predict self-regard and interpersonal behavior. *Journal of Personality, 88*(6), 1111-1128. doi:10.1111/jopy.12559

Mahadevan, N., Gregg, A. P., Sedikides, C., & De Waal-Andrews, W. G. (2016). Winners, losers, insiders, and outsiders: Comparing hierometer and sociometer theories of self-regard. *Frontiers in Psychology, 7*(MAR). doi:10.3389/fpsyg.2016.00334

Mahadevan, N., & Jordan, C. (2022). Desperately Seeking Status: How Desires for, and Perceived Attainment of, Status and Inclusion Relate to Grandiose and Vulnerable Narcissism. *Personality and Social Psychology Bulletin*, *48*(5), 704-717. <https://doi.org/10.1177/01461672211021189>

Mahmoud, A. B., Hack-polay, D., Fuxman, L., Massetti, B., & Al Samarh, A. Z. (2020). Developing and validating a new multi-dimensional scale for anti-social behavior in a higher education setting. *Scandinavian Journal of Psychology, 61*(4), 502-512. doi:10.1111/sjop.12635

Mailhos, A., Buunk, A. P., & Cabana, Á. (2016). Signature size signals sociable dominance and narcissism. *Journal of Research in Personality, 65*, 43-51. doi:10.1016/j.jrp.2016.09.004

Malesza, M., & Kaczmarek, M. C. (2018). Grandiose narcissism versus vulnerable narcissism and impulsivity. *Personality and Individual Differences, 126*, 61-65. doi:10.1016/j.paid.2018.01.021

Malesza, M., & Kalinowski, K. (2019). Dark triad and impulsivity – an ecological momentary assessment approach. *Current Psychology*. doi:10.1007/s12144-019-00320-y

Malkin, M. L., Zeigler-Hill, V., Barry, C. T., & Southard, A. C. (2013). The View From the Looking Glass: How Are Narcissistic Individuals Perceived by Others? *Journal of Personality, 81*(1), 1-15. doi:10.1111/j.1467-6494.2013.00780.x

Maltby, J. (2010). An interest in fame: Confirming the measurement and empirical conceptualization of fame interest. *British Journal of Psychology, 101*(3), 411-432. doi:10.1348/000712609X466568

Mandarino, K., Pruett, M. K., & Fieldstone, L. (2016). Co-parenting in a Highly Conflicted Separation/Divorce: Learning about Parents and their Experiences of Parenting Coordination, Legal, and Mental Health Interventions. *Family Court Review, 54*(4), 564-577. doi:10.1111/fcre.12243

Manley, H., Roberts, R., Beattie, S., & Woodman, T. (2018). I'll get there because I'm great, or am I? Narcissistic vulnerability moderates the narcissistic grandiosity – goal persistence relationship. *Personality and Individual Differences, 120*, 65-74. doi:10.1016/j.paid.2017.08.024

Mann, H. (2006). *Perfectionism and Defensively High Self-esteem: Understanding the Role of Perfectionism, Implicit Self-esteem, and Explicit Self-esteem in Predicting Defensiveness*: ProQuest.

Manson, J. H. (2020). Is Narcissism a Slow Life History Strategy Indicator?: The Answer Depends on the LHS Instrument. *Evolutionary Psychology, 18*(3). doi:10.1177/1474704920946236

Maples-Keller, J. L., & Miller, J. D. (2018). Insight and the dark triad: Comparing self- and meta-perceptions in relation to psychopathy, narcissism, and machiavellianism. *Personality Disorders: Theory, Research, and Treatment, 9*(1), 30-39. doi:10.1037/per0000207

March, E., Litten, V., Sullivan, D. H., & Ward, L. (2020). Somebody that I (used to) know: Gender and dimensions of dark personality traits as predictors of intimate partner cyberstalking. *Personality and Individual Differences, 163*. doi:10.1016/j.paid.2020.110084

March, E., & McBean, T. (2018). New evidence shows self-esteem moderates the relationship between narcissism and selfies. *Personality and Individual Differences, 130*, 107-111. doi:10.1016/j.paid.2018.03.053

Marchlewska, M., Castellanos, K. A., Lewczuk, K., Kofta, M., & Cichocka, A. (2019). My way or the highway: High narcissism and low self-esteem predict decreased support for democracy. *British Journal of Social Psychology, 58*(3), 591-608. doi:10.1111/bjso.12290

Marchlewska, M., & Cichocka, A. (2017). An autobiographical gateway: Narcissists avoid first-person visual perspective while retrieving self-threatening memories. *Journal of Experimental Social Psychology, 68*, 157-161. doi:10.1016/j.jesp.2016.06.003

Marcus, B., MacHilek, F., & Schütz, A. (2006). Personality in cyberspace: Personal Web sites as media for personality expressions and impressions. *Journal of Personality and Social Psychology, 90*(6), 1014-1031. doi:10.1037/0022-3514.90.6.1014

Marcus, B., & Schütz, A. (2005). Who are the people reluctant to participate in research? Personality correlates of four different types of noniesponse as inferred from self- And observer ratings. *Journal of Personality, 73*(4), 959-984. doi:10.1111/j.1467-6494.2005.00335.x

Marcus, D. K., Preszler, J., & Zeigler-Hill, V. (2018). A network of dark personality traits: What lies at the heart of darkness? *Journal of Research in Personality, 73*, 56-62. doi:10.1016/j.jrp.2017.11.003

Marion, B. E., & Sellbom, M. (2011). An examination of gender-moderated test bias on the Levenson Self-Report Psychopathy Scale. *Journal of Personality Assessment, 93*(3), 235-243. doi:10.1080/00223891.2011.558873

Mark Young, S., & Pinsky, D. (2006). Narcissism and celebrity. *Journal of Research in Personality, 40*(5), 463-471. doi:10.1016/j.jrp.2006.05.005

Marks, A. D., Blore, R. L., Hine, D. W., & Dear, G. E. (2012). Development and validation of a revised measure of codependency. *Australian Journal of Psychology, 64*(3), 119-127. doi:10.1111/j.1742-9536.2011.00034.x

Marshall, T. C., Lefringhausen, K., & Ferenczi, N. (2015). The Big Five, self-esteem, and narcissism as predictors of the topics people write about in Facebook status updates. *Personality and Individual Differences, 85*, 35-40. doi:10.1016/j.paid.2015.04.039

Martin, B. A. S., Jin, H. S., O'Connor, P. J., & Hughes, C. (2019). The relationship between narcissism and consumption behaviors: A comparison of measures. *Personality and Individual Differences, 141*, 196-199. doi:10.1016/j.paid.2019.01.014

Martin, R. M. (2020). Gender differences in competitiveness and narcissism as exhibited through pictures on Facebook. *Personality and Individual Differences, 162*. doi:10.1016/j.paid.2020.110011

Martinez, M. A., Zeichner, A., Reidy, D. E., & Miller, J. D. (2008). Narcissism and displaced aggression: Effects of positive, negative, and delayed feedback. *Personality and Individual Differences, 44*(1), 140-149. doi:10.1016/j.paid.2007.07.012

Martinsen, Ø. L., Arnulf, J. K., Furnham, A., & Lang-Ree, O. C. (2019). Narcissism and creativity. *Personality and Individual Differences, 142*, 166-171. doi:10.1016/j.paid.2018.09.032

Martingano, A. J., Konrath, S., Zarins, S., & Okaomee, A. A. (2022). Empathy, Narcissism, Alexithymia, and Social Media Use. *Psychology of Popular Media*, *11*(4), 413-422. <https://doi.org/10.1037/ppm0000419>

Martinovic, D., Tokic, D., Martinovic, L., Rakusic, M., Kumric, M., Rusic, D., Vilovic, M., Vrdoljak, J., Kurir, T. T., & Bozic, J. (2022). Orthorexia nervosa and its association with narcissism in fitness center users. *Eating and Weight Disorders-Studies on Anorexia Bulimia and Obesity*, *27*(6), 2155-2163. <https://doi.org/10.1007/s40519-022-01368-9>

Maske, M. K., & Sohn, M. (2023). Do Birds of a Feather Flock Together? The Joint Effects of Manager and Subordinate Narcissism on Performance Evaluation. *European Accounting Review*, 25. <https://doi.org/10.1080/09638180.2023.2235379>

Mathieu, C., & St-Jean, T. (2013). Entrepreneurial personality: The role of narcissism. *Personality and Individual Differences, 55*(5), 527-531. doi:10.1016/j.paid.2013.04.026

Matosic, D., Ntoumanis, N., Boardley, I. D., & Sedikides, C. (2020). Narcissism, beliefs about controlling interpersonal style, and moral disengagement in sport coaches. *International Journal of Sport and Exercise Psychology, 18*(5), 592-606. doi:10.1080/1612197X.2018.1549580

Matosic, D., Ntoumanis, N., Boardley, I. D., Sedikides, C., Stewart, B. D., & Chatzisarantis, N. (2017). Narcissism and coach interpersonal style: A self-determination theory perspective. *Scandinavian Journal of Medicine and Science in Sports, 27*(2), 254-261. doi:10.1111/sms.12635

Matosic, D., Ntoumanis, N., Boardley, I. D., Stenling, A., & Sedikides, C. (2016). Linking narcissism, motivation, and doping attitudes in sport: A multilevel investigation involving coaches and athletes. *Journal of Sport and Exercise Psychology, 38*(6), 556-566. doi:10.1123/jsep.2016-0141

Matsuo, A., & DeSouza, E. R. (2016). The Effects of Anticipated Negative Feedback on Psychological States Among Narcissists. *SAGE Open, 6*(2). doi:10.1177/2158244016650921

Milic, A., Kardum, I., & Svegar, D. (2023). Contours of the envious personality: Reassessing the capacity of the Big Five and the Dark Triad personality traits in predicting dispositional envy. *Current Psychology*, *42*(16), 14051-14064. <https://doi.org/10.1007/s12144-021-02633-3>

Miller, B. (2022). Exploring the Posting of Nude Photographs on Reddit in Relation to Self-Esteem, Perceived Attractiveness, Narcissism, and Sensation Seeking. *Archives of Sexual Behavior*, *51*(6), 3083-3092. <https://doi.org/10.1007/s10508-022-02301-y>

Mithen, P., Briant, O., & Shaw, L. (2023). Do you really want to hurt me?: Exploring the role of Narcissism, driver comparison and ego threat in driver aggression using a conceptual definition of aggression. *Transportation Research Part F-Traffic Psychology and Behaviour*, *95*, 450-463. <https://doi.org/10.1016/j.trf.2023.05.003>

Maxwell, K., Donnellan, M. B., Hopwood, C. J., & Ackerman, R. A. (2011). The two faces of Narcissus? An empirical comparison of the Narcissistic Personality Inventory and the Pathological Narcissism Inventory. *Personality and Individual Differences, 50*(5), 577-582. doi:10.1016/j.paid.2010.11.031

Mayer, J. D., Panter, A. T., & Caruso, D. R. (2012). Does personal intelligence exist? Evidence from a new ability-based measure. *Journal of Personality Assessment, 94*(2), 124-140. doi:10.1080/00223891.2011.646108

Maynard, D. C., Brondolo, E. M., Connelly, C. E., & Sauer, C. E. (2015). I’m too good for this job: Narcissism’s role in the experience of overqualification. *Applied Psychology, 64*(1), 208-232. doi:10.1111/apps.12031

McCann, J. T., & Biaggio, M. K. (1989). NARCISSISTIC PERSONALITY FEATURES AND SELF-REPORTED ANGER. *Psychological Reports, 64*(1), 55-58. doi:10.2466/pr0.1989.64.1.55

McCullough, M. E., Emmons, R. A., Kilpatrick, S. D., & Mooney, C. N. (2003). Narcissists as "victims": The role of narcissism in the perception of transgressions. *Personality and Social Psychology Bulletin, 29*(7), 885-893. doi:10.1177/0146167203029007007

McDaniel, A. K., McKinney, B. C., & Kimsey, W. D. (2017). The effect of narcissism on conflict management message style preference: A look at millennials. *American Communication Journal, 19*(1), 1-10. Retrieved from https://www.scopus.com/inward/record.uri?eid=2-s2.0-85019560542&partnerID=40&md5=03a5ce861a55cd613876292448af167e

McGinty, M. M. (2015). *Healthy retirement in former NFL players| The role of narcissism.* Alliant International University, Retrieved from http://oatd.org/oatd/record?record=oai\:pqdtoai.proquest.com\:3702684&q=%20%28NPI%29%20OR%20%28Narcissistic%20AND%20Personality%20AND%20Inventory%29%20

McGregor, I., Nail, P. R., Kocalar, D., & Haji, R. (2013). I'm OK, I'm OK: Praise makes narcissists with low implicit self-esteem indifferent to the suffering of others. *Personality and Individual Differences, 55*(6), 655-659. doi:10.1016/j.paid.2013.05.007

McHoskey, J. (1995). NARCISSISM AND MACHIAVELLIANISM. *Psychological Reports, 77*(3), 755-759. doi:10.2466/pr0.1995.77.3.755

McHoskey, J. W., Worzel, W., & Szyarto, C. (1998). Machiavellianism and Psychopathy. *Journal of Personality and Social Psychology, 74*(1), 192-210. doi:10.1037/0022-3514.74.1.192

McIntyre, K. L. (2017). *Narcissism in Athletics| Is the Athlete/Jock Distinction Useful?* The Chicago School of Professional Psychology, Retrieved from http://oatd.org/oatd/record?record=oai\:pqdtoai.proquest.com\:10260542&q=%20%28NPI%29%20OR%20%28Narcissistic%20AND%20Personality%20AND%20Inventory%29%20

McIntyre, M. H., Barrett, E. S., McDermott, R., Johnson, D. D. P., Cowden, J., & Rosen, S. P. (2007). Finger length ratio (2D:4D) and sex differences in aggression during a simulated war game. *Personality and Individual Differences, 42*(4), 755-764. doi:10.1016/j.paid.2006.08.009

McKinney, B. C., Kelly, L., & Duran, R. L. (2012). Narcissism or Openness?: College Students' Use of Facebook and Twitter. *Communication Research Reports, 29*(2), 108-118. doi:10.1080/08824096.2012.666919

McLarty, B. D., & Holt, D. T. (2019). A Bright Side to Family Firms: How Socioemotional Wealth Importance Affects Dark Traits–Job Performance Relationships. *Family Business Review, 32*(4), 378-395. doi:10.1177/0894486519888397

McLarty, B. D., Skorodziyevskiy, V., & Muldoon, J. The Dark Triad's incremental influence on entrepreneurial intentions. *Journal of Small Business Management*. doi:10.1080/00472778.2021.1883042

McLarty, B. D., Skorodziyevskiy, V., & Muldoon, J. (2021). The Dark Triad’s incremental influence on entrepreneurial intentions. *Journal of Small Business Management*, 1-29. doi:10.1080/00472778.2021.1883042

McNulty, J. K., & Russell, V. M. (2016). Forgive and Forget, or Forgive and Regret? Whether Forgiveness Leads to Less or More Offending Depends on Offender Agreeableness. *Personality and Social Psychology Bulletin, 42*(5), 616-631. doi:10.1177/0146167216637841

McNulty, J. K., & Widman, L. (2013). The implications of sexual narcissism for sexual and marital satisfaction. *Archives of Sexual Behavior, 42*(6), 1021-1032. doi:10.1007/s10508-012-0041-5

Mefdedović, J., & Petrović, B. (2016). Can there be an immoral morality? Dark personality traits as predictors of Moral foundations. *Psihologija, 49*(2), 185-197. doi:10.2298/PSI1602185M

Mehdizadeh, S. (2010). Self-presentation 2.0: Narcissism and self-esteem on facebook. *Cyberpsychology, Behavior, and Social Networking, 13*(4), 357-364. doi:10.1089/cyber.2009.0257

Meier, L. L., & Semmer, N. K. (2012). Lack of reciprocity and strain: Narcissism as a moderator of the association between feeling under-benefited and irritation. *Work and Stress, 26*(1), 56-67. doi:10.1080/02678373.2012.657038

Meier, L. L., & Semmer, N. K. (2013). Lack of reciprocity, narcissism, anger, and instigated workplace incivility: A moderated mediation model. *European Journal of Work and Organizational Psychology, 22*(4), 461-475. doi:10.1080/1359432X.2012.654605

Meisel, M. K., Ning, H., Campbell, W. K., & Goodie, A. S. (2016). Narcissism, Overconfidence, and Risk Taking in U.S. and Chinese Student Samples. *Journal of Cross-Cultural Psychology, 47*(3), 385-400. doi:10.1177/0022022115621968

Menton, W. H., Crighton, A. H., Tarescavage, A. M., Marek, R. J., Hicks, A. D., & Ben-Porath, Y. S. (2019). Equivalence of Laptop and Tablet Administrations of the Minnesota Multiphasic Personality Inventory–2 Restructured Form. *Assessment, 26*(4), 661-669. doi:10.1177/1073191117714558

Meurs, J. A., Fox, S., Kessler, S. R., & Spector, P. E. (2013). It's all about me: The role of narcissism in exacerbating the relationship between stressors and counterproductive work behaviour. *Work and Stress, 27*(4), 368-382. doi:10.1080/02678373.2013.849776

Michel, J. S., & Bowling, N. A. (2013). Does Dispositional Aggression Feed the Narcissistic Response? The Role of Narcissism and Aggression in the Prediction of Job Attitudes and Counterproductive Work Behaviors. *Journal of Business and Psychology, 28*(1), 93-105. doi:10.1007/s10869-012-9265-6

Miles, G. J., Smyrnios, K. X., Jackson, M., & Francis, A. J. P. (2019). Reward-punishment sensitivity bias predicts narcissism subtypes: Implications for the etiology of narcissistic personalities. *Personality and Individual Differences, 141*, 143-151. doi:10.1016/j.paid.2019.01.004

Miller, J. D., & Campbell, W. K. (2008). Comparing clinical and social-personality conceptualizations of narcissism. *Journal of Personality, 76*(3), 449-476. doi:10.1111/j.1467-6494.2008.00492.x

Miller, J. D., Campbell, W. K., Young, D. L., Lakey, C. E., Reidy, D. E., Zeichner, A., & Goodie, A. S. (2009). Examining the relations among narcissism, impulsivity, and self-defeating behaviors. *Journal of Personality, 77*(3), 761-794. doi:10.1111/j.1467-6494.2009.00564.x

Miller, J. D., Dir, A., Gentile, B., Wilson, L., Pryor, L. R., & Campbell, W. K. (2010). Searching for a Vulnerable Dark Triad: Comparing Factor 2 Psychopathy, Vulnerable Narcissism, and Borderline Personality Disorder. *Journal of Personality, 78*(5), 1529-1564. doi:10.1111/j.1467-6494.2010.00660.x

Miller, J. D., Gaughan, E. T., Maples, J., & Price, J. (2011). A comparison of agreeableness scores from the big five inventory and the neo PI-R: Consequences for the study of narcissism and psychopathy. *Assessment, 18*(3), 335-339. doi:10.1177/1073191111411671

Miller, J. D., Gaughan, E. T., Pryor, L. R., Kamen, C., & Campbell, W. K. (2009). Is research using the narcissistic personality inventory relevant for understanding narcissistic personality disorder? *Journal of Research in Personality, 43*(3), 482-488. doi:10.1016/j.jrp.2009.02.001

Miller, J. D., Gentile, B., & Campbell, W. K. (2013). A test of the construct validity of the five-factor narcissism inventory. *Journal of Personality Assessment, 95*(4), 377-387. doi:10.1080/00223891.2012.742903

Miller, J. D., Gentile, B., Carter, N. T., Crowe, M., Hoffman, B. J., & Campbell, W. K. (2018). A Comparison of the Nomological Networks Associated With Forced-Choice and Likert Formats of the Narcissistic Personality Inventory. *Journal of Personality Assessment, 100*(3), 259-267. doi:10.1080/00223891.2017.1310731

Miller, J. D., Gentile, B., Wilson, L., & Campbell, W. K. (2013). Grandiose and vulnerable narcissism and the DSM-5 pathological personality trait model. *Journal of Personality Assessment, 95*(3), 284-290. doi:10.1080/00223891.2012.685907

Miller, J. D., Lynam, D. R., McCain, J. L., Few, L. R., Crego, C., Widiger, T. A., & Campbell, W. K. (2016). Thinking structurally about narcissism: An examination of the five-factor narcissism inventory and its components. *Journal of Personality Disorders, 30*(1), 1-18. doi:10.1521/pedi_2015_29_177

Miller, J. D., Maples, J. L., Buffardi, L., Cai, H., Gentile, B., Kisbu-Sakarya, Y., . . . Campbell, W. K. (2015). Narcissism and United States' culture: The view from home and around the world. *Journal of Personality and Social Psychology, 109*(6), 1068-1089. doi:10.1037/a0039543

Miller, J. D., Price, J., & Campbell Ii, W. K. (2012). Is the Narcissistic Personality Inventory Still Relevant? A Test of Independent Grandiosity and Entitlement Scales in the Assessment of Narcissism. *Assessment, 19*(1), 8-13. doi:10.1177/1073191111429390

Miller, K. J., & Mesagno, C. (2014). Personality traits and exercise dependence: Exploring the role of narcissism and perfectionism. *International Journal of Sport and Exercise Psychology, 12*(4), 368-381. doi:10.1080/1612197X.2014.932821

Miller, L. A. (2014). *Measuring the capacity for cognitive and affective empathy in psychopathy and narcissism*: Western Carolina University.

Miller, M. J., Smith, T. S., Wilkinson, L., & Tobacyk, J. (1987). NARCISSISM AND SOCIAL INTEREST AMONG COUNSELORS-IN-TRAINING. *Psychological Reports, 60*(3), 765-766. doi:10.2466/pr0.1987.60.3.765

Moeller, S. J., Crocker, J., & Bushman, B. J. (2009). Creating hostility and conflict: Effects of entitlement and self-image goals. *Journal of Experimental Social Psychology, 45*(2), 448-452. doi:10.1016/j.jesp.2008.11.005

Mohd. Shamsudin, F., Hamouche, S., Abdulmajid Cheikh Ali, D., Bani-Melhem, S., & Jamal Bani-Melhem, A. (2022). Why do employees withhold knowledge? The role of competitive climate, envy and narcissism. *Journal of Knowledge Management*, *27*(7), 1925-1947. <https://doi.org/10.1108/JKM-02-2022-0133>

Molenda, Z., Marchlewska, M., & Rogoza, M. (2023). Nothing hurts like (in-group) love? National narcissism, conspiracy intentions, and non-prosocial managing emotions of others. *Personality and Individual Differences*, *201*, 5. <https://doi.org/10.1016/j.paid.2022.111947>

Moon, H. C., & Kim, W. (2022). The effect of leadership type on follower performance in Asian technology industries: the moderating effects of narcissism. *Asian Journal of Technology Innovation*, 31. <https://doi.org/10.1080/19761597.2022.2127814>

Morf, C. C., & Rhodewalt, F. (1993). NARCISSISM AND SELF-EVALUATION MAINTENANCE - EXPLORATIONS IN OBJECT RELATIONS. *Personality and Social Psychology Bulletin, 19*(6), 668-676. doi:10.1177/0146167293196001

Morf, C. C., Schürch, E., Küfner, A., Siegrist, P., Vater, A., Back, M., . . . Schröder-Abé, M. (2017). Expanding the Nomological Net of the Pathological Narcissism Inventory: German Validation and Extension in a Clinical Inpatient Sample. *Assessment, 24*(4), 419-443. doi:10.1177/1073191115627010

Morgades-Bamba, C. I., Raynal, P., & Chabrol, H. (2020). Exploring the Radicalization Process in Young Women. *Terrorism and Political Violence, 32*(7), 1439-1457. doi:10.1080/09546553.2018.1481051

Moskowitz, D. A., Rieger, G., & Seal, D. W. (2009). Narcissism, self-evaluations, and partner preferences among men who have sex with men. *Personality and Individual Differences, 46*(7), 725-728. doi:10.1016/j.paid.2009.01.033

Mota, S., Leckelt, M., Geukes, K., Nestler, S., Humberg, S., Schröder-Abé, M., . . . Back, M. D. (2019). A comprehensive examination of narcissists’ self-perceived and actual socioemotional cognition ability. *Collabra: Psychology, 5*(1). doi:10.1525/collabra.174

Mouilso, E. R., & Calhoun, K. S. (2012). Narcissism, psychopathy and Five-Factor Model in sexual assault perpetration. *Personality and Mental Health, 6*(3), 228-241. doi:10.1002/pmh.1188

Mouilso, E. R., & Calhoun, K. S. (2016). Personality and Perpetration: Narcissism Among College Sexual Assault Perpetrators. *Violence Against Women, 22*(10), 1228-1242. doi:10.1177/1077801215622575

Myers, E. M., & Zeigler-Hill, V. (2008). No shades of gray: Splitting and self-esteem instability. *Personality and Individual Differences, 45*(2), 139-145. doi:10.1016/j.paid.2008.03.012

Myers, E. M., & Zeigler-Hill, V. (2012). How much do narcissists really like themselves? Using the bogus pipeline procedure to better understand the self-esteem of narcissists. *Journal of Research in Personality, 46*(1), 102-105. doi:10.1016/j.jrp.2011.09.006

Naderi, I., & Strutton, D. (2014). Can normal narcissism be managed to promote green product purchases? Investigating a counterintuitive proposition. *Journal of Applied Social Psychology, 44*(5), 375-391. doi:10.1111/jasp.12230

Narayan, C. (1990). BIRTH-ORDER AND NARCISSISM. *Psychological Reports, 67*(3), 1184-1186.

Nathan DeWall, C., Buffardi, L. E., Bonser, I., & Keith Campbell, W. (2011). Narcissism and implicit attention seeking: Evidence from linguistic analyses of social networking and online presentation. *Personality and Individual Differences, 51*(1), 57-62. doi:10.1016/j.paid.2011.03.011

Neave, L., Tzemou, E., & Fastoso, F. (2020). Seeking attention versus seeking approval: How conspicuous consumption differs between grandiose and vulnerable narcissists. *Psychology and Marketing, 37*(3), 418-427. doi:10.1002/mar.21308

Neckar, J. (2013). Self-enhancement and coping:The costs and benefits of positive illusions. *Studia Psychologica, 55*(4), 299-310. doi:10.21909/sp.2013.04.644

Nelson, J. M., Hardy, S. A., & Watkins, P. (2023). Transcendent Indebtedness to God: A New Construct in the Psychology of Religion and Spirituality. *Psychology of Religion and Spirituality*, *15*(1), 105-117. <https://doi.org/10.1037/rel0000458>

Nenadic, I., Lorenz, C., & Gaser, C. (2021). Narcissistic personality traits and prefrontal brain structure. *Scientific Reports*, *11*(1), 9. <https://doi.org/10.1038/s41598-021-94920-z>

Neria, A. L., Vizcaino, M., & Jones, D. N. (2016). Approach/avoidance tendencies in dark personalities. *Personality and Individual Differences, 101*, 264-269. doi:10.1016/j.paid.2016.05.054

Neufeld, D. C. (2013). *Who is the most envious of them all? Examining how 3 narcissistic subtypes relate to dispositional and episodic envy*: University of Manitoba (Canada).

Neumann, E., Obliers, R., & Albus, C. (2012). Medical Students' Attitudes towards Mental Illness - A Matter of Studies or Personality? *Psychotherapie Psychosomatik Medizinische Psychologie, 62*(2), 66-72. doi:10.1055/s-0031-1301360

Nevicka, B., Baas, M., & Ten Velden, F. S. (2016). The Bright Side of Threatened Narcissism: Improved Performance Following Ego Threat. *Journal of Personality, 84*(6), 809-823. doi:10.1111/jopy.12223

Nevicka, B., De Hoogh, A. H. B., Den Hartog, D. N., & Belschak, F. D. (2018). Narcissistic leaders and their victims: Followers low on self-esteem and low on core self-evaluations suffer most. *Frontiers in Psychology, 9*(MAR). doi:10.3389/fpsyg.2018.00422

Nevicka, B., De Hoogh, A. H. B., Van Vianen, A. E. M., Beersma, B., & McIlwain, D. (2011). All I need is a stage to shine: Narcissists' leader emergence and performance. *Leadership Quarterly, 22*(5), 910-925. doi:10.1016/j.leaqua.2011.07.011

Nevicka, B., & Sedikides, C. (2021). Employee narcissism and promotability prospects. *Journal of Personality*. doi:10.1111/jopy.12619

Nevicka, B., Van Vianen, A. E. M., De Hoogh, A. H. B., & Voorn, B. C. M. (2018). Narcissistic leaders: An asset or a liability? Leader visibility, follower responses, and group-level absenteeism. *Journal of Applied Psychology, 103*(7), 703-723. doi:10.1037/apl0000298

Neville, L., & Fisk, G. M. (2019). Getting to Excess: Psychological Entitlement and Negotiation Attitudes. *Journal of Business and Psychology, 34*(4), 555-574. doi:10.1007/s10869-018-9557-6

Ng, H. K. S., Cheung, R. Y. H., & Tam, K. P. (2014). Unraveling the link between narcissism and psychological health: New evidence from coping flexibility. *Personality and Individual Differences, 70*, 7-10. doi:10.1016/j.paid.2014.06.006

Ng, H. K. S., Tam, K. P., & Shu, T. M. (2011). The money attitude of covert and overt narcissists. *Personality and Individual Differences, 51*(2), 160-165. doi:10.1016/j.paid.2011.03.036

Nguyen, K. T., & Shaw, L. (2020). The aetiology of non-clinical narcissism: Clarifying the role of adverse childhood experiences and parental overvaluation. *Personality and Individual Differences, 154*. doi:10.1016/j.paid.2019.109615

Nicholls, E., & Stukas, A. A. (2011). Narcissism and the self-evaluation maintenance model: Effects of social comparison threats on relationship closeness. *The Journal of social psychology, 151*(2), 201-212.

Niemyjska, A., Bazińska, R., & Drat-Ruszczak, K. (2020). Hunting lovers: Narcissists keep trophies from their past relationships. *Personality and Individual Differences, 163*. doi:10.1016/j.paid.2020.110060

Noser, A. E., Zeigler-Hill, V., & Besser, A. (2014). Stress and affective experiences: The importance of dark personality features. *Journal of Research in Personality, 53*, 158-164. doi:10.1016/j.jrp.2014.10.007

O'Neill, T. A., & Allen, N. J. (2014). Team task conflict resolution: An examination of its linkages to team personality composition and team effectiveness outcomes. *Group Dynamics, 18*(2), 159-173. doi:10.1037/gdn0000004

O'Reilly, C. A., & Hall, N. (2021). Grandiose narcissists and decision making: Impulsive, overconfident, and skeptical of experts–but seldom in doubt. *Personality and Individual Differences, 168*. doi:10.1016/j.paid.2020.110280

O'Reilly, C. A., III, & Doerr, B. (2020). Conceit and deceit: Lying, cheating, and stealing among grandiose narcissists. *Personality and Individual Differences, 154*. doi:10.1016/j.paid.2019.109627

O'Reilly, C. A., III, Doerr, B., & Chatman, J. A. (2018). “See You in Court”: How CEO narcissism increases firms’ vulnerability to lawsuits. *Leadership Quarterly, 29*(3), 365-378. doi:10.1016/j.leaqua.2017.08.001

O'Reilly, C. A., & Pfeffer, J. (2021). Why are grandiose narcissists more effective at organizational politics? Means, motive, and opportunity. *Personality and Individual Differences, 172*. doi:10.1016/j.paid.2020.110557

O'Reilly, C. A., Chatman, J. A., & Doerr, B. (2021). When „me“ trumps „we“: Narcissistic leaders and cultures they create. *Academy of Management Discoveries*, *7*(3), 419-450. <https://doi.org/10.5465/amd.2019.0163>

O'Reilly, C. A., & Pfeffer, J. (2021). Organizational power and politics: The narcissist's advantage? [Article]. *Personality and Individual Differences*, *182*, 6, Article 111061. <https://doi.org/10.1016/j.paid.2021.111061>

Oashi, O. (2004). Relation of type a behavior and multidimensionally measured narcissistic personality of Japanese university students. *Psychological Reports, 94*(1), 51-54. doi:10.2466/pr0.94.1.51-54

Odaci, H., & Çelik, Ç. B. (2013). Who are problematic internet users? An investigation of the correlations between problematic internet use and shyness, loneliness, narcissism, aggression and self-perception. *Computers in Human Behavior, 29*(6), 2382-2387. doi:10.1016/j.chb.2013.05.026

Odermatt, I., König, C. J., Kleinmann, M., Bachmann, M., Röder, H., & Schmitz, P. (2018). Incivility in Meetings: Predictors and Outcomes. *Journal of Business and Psychology, 33*(2), 263-282. doi:10.1007/s10869-017-9490-0

Ohmann, K., & Burgmer, P. (2016). Nothing compares to me: How narcissism shapes comparative thinking. *Personality and Individual Differences, 98*, 162-170. doi:10.1016/j.paid.2016.03.069

Olejarnik, S. Z., & Romano, D. (2023). Is playing violent video games a risk factor for aggressive behaviour? Adding narcissism, self-esteem and PEGI ratings to the debate. *Frontiers in Psychology*, *14*, 13. <https://doi.org/10.3389/fpsyg.2023.1155807>

Oleson, K. C., Poehlmann, K. M., Yost, J. H., Lynch, M. E., & Arkin, R. M. (2000). Subjective overachievement: Individual differences in self-doubt and concern with performance. *Journal of Personality, 68*(3), 491-524. doi:10.1111/1467-6494.00104

Ong, C. W., Roberts, R., Woodman, T., & Arthur, C. A. (2022). The Leader Ship Was Destined to Sink: An Examination of Dominance and Prestige on the Rise and Fall of the Narcissistic Leader. *Group Dynamics-Theory Research and Practice*, 10. <https://doi.org/10.1037/gdn0000177>

Olssøn, I., Svindseth, M. F., & Dahl, A. A. (2016). Is there an association between the level of grandiose narcissism severity of psychopathology? *Nordic Journal of Psychiatry, 70*(2), 121-127. doi:10.3109/08039488.2015.1058418

Omori, K., & Allen, M. R. (2021). Narcissism as a predictor of number of selfies: a cross-cultural examination of Japanese and American postings. *Communication Research Reports*. doi:10.1080/08824096.2021.1914017

Ong, C. W., Roberts, R., Arthur, C. A., Woodman, T., & Akehurst, S. (2016). The Leader Ship Is Sinking: A Temporal Investigation of Narcissistic Leadership. *Journal of Personality, 84*(2), 237-247. doi:10.1111/jopy.12155

Öngen, D. E. (2010). *Relationships between narcissism and aggression among non-referred Turkish university students.* Paper presented at the Procedia - Social and Behavioral Sciences.

Onley, M., Veselka, L., Schermer, J. A., & Vernon, P. A. (2013). Survival of the scheming: A genetically informed link between the dark triad and mental toughness. *Twin Research and Human Genetics, 16*(6), 1087-1095. doi:10.1017/thg.2013.66

Opree, S. J., & Kühne, R. (2016). Generation Me in the Spotlight: Linking Reality TV to Materialism, Entitlement, and Narcissism. *Mass Communication and Society, 19*(6), 800-819. doi:10.1080/15205436.2016.1199706

Orth, U., & Luciano, E. C. (2015). Self-esteem, narcissism, and stressful life events: Testing for selection and socialization. *Journal of Personality and Social Psychology, 109*(4), 707-721. doi:10.1037/pspp0000049

Oswald, F., Lopes, A., Skoda, K., Hesse, C. L., & Pedersen, C. L. (2020). I’ll Show You Mine so You’ll Show Me Yours: Motivations and Personality Variables in Photographic Exhibitionism. *Journal of Sex Research, 57*(5), 597-609. doi:10.1080/00224499.2019.1639036

Otterbring, T., Festila, A., & Folwarczny, M. (2021). Selfless or Selfish? The impact of message framing and egoistic motivation on narcissists’ compliance with preventive health behaviors during COVID-19. *Current Research in Ecological and Social Psychology*, *2*. <https://doi.org/10.1016/j.cresp.2021.100023>

Özcan, N. A., Kocak, Ö. E., & Arslan, R. (2018). The role of aggression in the relationship between grandiose narcissistic traits and interpersonal style: University students in Turkey. *Klinik Psikiyatri Dergisi, 21*(4), 341-350. doi:10.5505/kpd.2018.85547

Ozimek, P., Bierhoff, H. W., & Rohmann, E. (2021). How Downward and Upward Comparisons on Facebook Influence Grandiose and Vulnerable Narcissists' Self-Esteem-A Priming Study. Behavioral *Sciences*, *11*(3). <https://doi.org/10.3390/bs11030039>

Ozkum, S. B., Lannin, D. G., & Wu, S. (2023). Divergent reactions to COVID-19 exhibited in grandiose and vulnerable narcissism. *Social and Personality Psychology Compass*, *17*(11), Article e12849. <https://doi.org/10.1111/spc3.12849>

Özsoy, E., Rauthmann, J. F., Jonason, P. K., & Ardıç, K. (2017). Reliability and validity of the Turkish versions of Dark Triad Dirty Dozen (DTDD-T), Short Dark Triad (SD3-T), and Single Item Narcissism Scale (SINS-T). *Personality and Individual Differences, 117*, 11-14. doi:10.1016/j.paid.2017.05.019

Packer West, M., Miller, J. D., & Lynam, D. R. (2024). Comparing Brief Measures of Narcissism—Internal Consistency, Validity, and Coverage. *Journal of Personality Assessment*, *106*(1), 83-99. <https://doi.org/10.1080/00223891.2023.2183863>

Pajevic, M., Vukosavljevic-Gvozden, T., Stevanovic, N., & Neumann, C. S. (2018). The relationship between the Dark Tetrad and a two-dimensional view of empathy. *Personality and Individual Differences, 123*, 125-130. doi:10.1016/j.paid.2017.11.009

Paleczek, D., Bergner, S., & Rybnicek, R. (2018). Predicting career success: is the dark side of personality worth considering? *Journal of Managerial Psychology, 33*(6), 437-456. doi:10.1108/JMP-11-2017-0402

Palmer, C. A., Ramsey, M. A., Morey, J. N., & Gentzler, A. L. (2016). How do people share their positive events?: Individual differences in capitalizing, bragging, and mass-sharing. *Journal of Individual Differences, 37*(4), 250-259. doi:10.1027/1614-0001/a000212

Panek, E. T., Nardis, Y., & Konrath, S. (2013). Defining social networking sites and measuring their use: How narcissists differ in their use of Facebook and Twitter. *Computers in Human Behavior, 29*(5), 2004-2012. doi:10.1016/j.chb.2013.04.012

Pantic, I., Milanovic, A., Loboda, B., Błachnio, A., Przepiorka, A., Nesic, D., . . . Ristic, S. (2017). Association between physiological oscillations in self-esteem, narcissism and internet addiction: A cross-sectional study. *Psychiatry Research, 258*, 239-243. doi:10.1016/j.psychres.2017.08.044

Park, A., Ickes, W., & Robinson, R. L. (2014). More f#!%ing rudeness: Reliable personality predictors of verbal rudeness and other ugly confrontational behaviors. *Journal of Aggression, Conflict and Peace Research, 6*(1), 26-43. doi:10.1108/JACPR-04-2013-0009

Park, S. W., & Colvin, C. R. (2014). Narcissism and Discrepancy Between Self and Friends' Perceptions of Personality. *Journal of Personality, 82*(4), 278-286. doi:10.1111/jopy.12053

Park, S. W., & Colvin, C. R. (2015). Narcissism and Other-Derogation in the Absence of Ego Threat. *Journal of Personality, 83*(3), 334-345. doi:10.1111/jopy.12107

Park, S. W., Ferrero, J., Colvin, C. R., & Carney, D. R. (2013). Narcissism and Negotiation: Economic Gain and Interpersonal Loss. *Basic and Applied Social Psychology, 35*(6), 569-574. doi:10.1080/01973533.2013.840633

Patel, S., Batterham, P. J., Calear, A. L., & Cryer, R. (2016). Predictors of Comfort and Confidence among Medical Students in Providing Care to Patients at Risk of Suicide. *Academic Psychiatry, 40*(6), 919-922. doi:10.1007/s40596-016-0583-2

Paulsen, J. (2018). *Correlates of Measures of Adaptive and Pathological Narcissism.* University of Minnesota, Retrieved from http://oatd.org/oatd/record?record=handle\:11299\%2F200278&q=%20%28NPI%29%20OR%20%28Narcissistic%20AND%20Personality%20AND%20Inventory%29%20

Pearson, C., & Hussain, Z. (2016). Smartphone use, addiction, narcissism, and personality: A mixed methods investigation. In *Gaming and Technology Addiction: Breakthroughs in Research and Practice* (Vol. 1, pp. 212-229).

Peebles, S. A. (2006). *An empirical and existential examination of narcissistic Functioning*: Auburn University.

Peralta, C. F., & Saldanha, M. F. (2017). Can dealing with emotional exhaustion lead to enhanced happiness? The roles of planning and social support. *Work and Stress, 31*(2), 121-144. doi:10.1080/02678373.2017.1308445

Persson, B. N. (2019). Searching for Machiavelli but finding psychopathy and narcissism. *Personality Disorders: Theory, Research, and Treatment, 10*(3), 235-245. doi:10.1037/per0000323

Peterson, J. L., & DeHart, T. (2014). In Defense of Self-Love: An Observational Study on Narcissists' Negative Behavior During Romantic Relationship Conflict. *Self and Identity, 13*(4), 477-490. doi:10.1080/15298868.2013.868368

Peterson, S. J., Galvin, B. M., & Lange, D. (2012). Ceo servant leadership: Exploring executive characteristics and firm performance. *Personnel Psychology, 65*(3), 565-596. doi:10.1111/j.1744-6570.2012.01253.x

Pethman, T. M. I., & Erlandsson, S. I. (2002). Aberrant self-promotion or subclinical psychopathy in a Swedish general population. *Psychological Record, 52*(1), 33-50. doi:10.1007/BF03395413

Piff, P. K. (2014). Wealth and the Inflated Self: Class, Entitlement, and Narcissism. *Personality and Social Psychology Bulletin, 40*(1), 34-43. doi:10.1177/0146167213501699

Pilch, I., & Górnik-Durose, M. E. (2016). Do we need “dark” traits to explain materialism? The incremental validity of the Dark Triad over the HEXACO domains in predicting materialistic orientation. *Personality and Individual Differences, 102*, 102-106. doi:10.1016/j.paid.2016.06.047

Pilch, I., & Górnik-Durose, M. E. (2017). Grandiose and Vulnerable Narcissism, Materialism, Money Attitudes, and Consumption Preferences. *Journal of Psychology: Interdisciplinary and Applied, 151*(2), 185-206. doi:10.1080/00223980.2016.1252707

Pincus, A. L., Ansell, E. B., Pimentel, C. A., Cain, N. M., Wright, A. G. C., & Levy, K. N. (2009). Initial Construction and Validation of the Pathological Narcissism Inventory. *Psychological Assessment, 21*(3), 365-379. doi:10.1037/a0016530

Plante, T. G., & McCreadie, A. (2019). The Santa Clara Ethics Scale. *Pastoral Psychology, 68*(3), 321-329. doi:10.1007/s11089-019-00861-w

Plouffe, R. A., Wilson, C. A., & Saklofske, D. H. (2020a). Examining the Relationships Between Childhood Exposure to Intimate Partner Violence, the Dark Tetrad of Personality, and Violence Perpetration in Adulthood. *Journal of Interpersonal Violence*. doi:10.1177/0886260520948517

Plouffe, R. A., Wilson, C. A., & Saklofske, D. H. (2020b). The role of dark personality traits in intimate partner violence: a multi-study investigation. *Current Psychology*. doi:10.1007/s12144-020-00871-5

Plouffe, R. A., Wilson, C. A., & Saklofske, D. H. (2022). Examining the Relationships Between Childhood Exposure to Intimate Partner Violence, the Dark Tetrad of Personality, and Violence Perpetration in Adulthood. *Journal of Interpersonal Violence*, *37*(5-6). <https://doi.org/10.1177/0886260520948517>

Podnar, D. J. (2013). Friendly antagonism in humorous interactions: Explorations of prosocial teasing.

Poless, P. G., Torstveit, L., Lugo, R. G., Andreassen, M., & Sütterlin, S. (2018). Guilt and proneness to shame: Unethical behaviour in vulnerable and grandiose narcissism. *Europe's Journal of Psychology, 14*(1), 28-43. doi:10.5964/ejop.v14i1.1355

Ponti, L., Ghinassi, S., & Tani, F. (2020). The Role of Vulnerable and Grandiose Narcissism in Psychological Perpetrated Abuse Within Couple Relationships: The Mediating Role of Romantic Jealousy. *Journal of Psychology: Interdisciplinary and Applied, 154*(2), 144-158. doi:10.1080/00223980.2019.1679069

Porcerelli, J. H., & Sandler, B. A. (1995). Narcissism and empathy in steroid users. *American Journal of Psychiatry, 152*(11), 1672-1674. doi:10.1176/ajp.152.11.1672

Porter, T., & Schumann, K. (2018). Intellectual humility and openness to the opposing view. *Self and Identity, 17*(2), 139-162. doi:10.1080/15298868.2017.1361861

Potard, C., Lignier, B., & Henry, A. (2018). Psychometric properties of a French version of the narcissistic personality inventory in young adults. *Swiss Journal of Psychology, 77*(1), 23-32. doi:10.1024/1421-0185/a000204

Pourramzani, A., & Monajemi, E. (2021). Prevalence of Adaptive Narcissism and Its Demographic Correlations Among the Interns of Guilan University of Medical Sciences, Medical Faculty: A Cross-sectional Study. *Iranian Journal of Psychiatry and Behavioral Sciences, 15*(1). doi:10.5812/ijpbs.101094

Pruysers, S. (2021). A psychological predisposition towards populism? Evidence from Canada. *Contemporary Politics, 27*(1), 105-124. doi:10.1080/13569775.2020.1851930

Przepiorka, A. M., Blachnio, A., & Wiesenthal, D. L. (2014). The determinants of driving aggression among Polish drivers. *Transportation Research Part F: Traffic Psychology and Behaviour, 27*(PA), 69-80. doi:10.1016/j.trf.2014.09.007

Rajesh, T., & Rangaiah, D. B. (2020). Facebook addiction and personality. *Heliyon, 6*(1). doi:10.1016/j.heliyon.2020.e03184

Raskin, R., & Novacek, J. (1989). An MMPI Description of the Narcissistic Personality. *Journal of Personality Assessment, 53*(1), 66-80. doi:10.1207/s15327752jpa5301_8

Raskin, R., & Terry, H. (1988). A PRINCIPAL-COMPONENTS ANALYSIS OF THE NARCISSISTIC PERSONALITY-INVENTORY AND FURTHER EVIDENCE OF ITS CONSTRUCT-VALIDITY. *Journal of Personality and Social Psychology, 54*(5), 890-902. doi:10.1037/0022-3514.54.5.890

Rathner, E. M., Djamali, J., Terhorst, Y., Schuller, B., Cummins, N., Salamon, G., . . . Baumeister, H. (2018). *How did you like 2017? Detection of language markers of depression and narcissism in personal narratives*.

Rathvon, N., & Holmstrom, R. W. (1996). An MMPI-2 portrait of narcissism. *Journal of Personality Assessment, 66*(1), 1-19. doi:10.1207/s15327752jpa6601_1

Rauthmann, J. F. (2011). Acquisitive or protective self-presentation of dark personalities? Associations among the Dark Triad and self-monitoring. *Personality and Individual Differences, 51*(4), 502-508. doi:10.1016/j.paid.2011.05.008

Rauthmann, J. F. (2012). The Dark Triad and Interpersonal Perception: Similarities and Differences in the Social Consequences of Narcissism, Machiavellianism, and Psychopathy. *Social Psychological and Personality Science, 3*(4), 487-496. doi:10.1177/1948550611427608

Rauthmann, J. F., & Sherman, R. A. (2023). Patterned person-situation fit in daily life: Examining magnitudes, stabilities, and correlates of trait-situation and state-situation fit. *European Journal of Personality*, *37*(5), 501-523. <https://doi.org/10.1177/08902070221104636>

Rawn, K. P., Keller, P. S., & Widiger, T. A. (2023). Parent Grandiose Narcissism and Child Socio-Emotional Well Being: The Role of Parenting. *Psychological Reports*, 19. <https://doi.org/10.1177/00332941231208900>

Re, D. E., Wang, S. A., He, J. C., & Rule, N. O. (2016). Selfie Indulgence: Self-Favoring Biases in Perceptions of Selfies. *Social Psychological and Personality Science, 7*(6), 588-596. doi:10.1177/1948550616644299

Reed, P., Bircek, N. I., Osborne, L. A., Viganò, C., & Truzoli, R. (2018). Visual social media use moderates the relationship between initial problematic internet use and later narcissism. *Open Psychology Journal, 11*(1), 163-170. doi:10.2174/1874350101811010163

Reid, A. J., & Thomas, C. N. (2017). A case study in smartphone usage and gratification in the age of narcissism. *International Journal of Technology and Human Interaction, 13*(2), 40-56. doi:10.4018/IJTHI.2017040103

Reidy, D. E., Foster, J. D., & Zeichner, A. (2010). Narcissism and Unprovoked Aggression. *Aggressive Behavior, 36*(6), 414-422. doi:10.1002/ab.20356

Reina, C. S., Zhang, Z., & Peterson, S. J. (2014). CEO grandiose narcissism and firm performance: The role of organizational identification. *Leadership Quarterly, 25*(5), 958-971. doi:10.1016/j.leaqua.2014.06.004

Reinhard, D. A., Konrath, S. H., Lopez, W. D., & Cameron, H. G. (2012). Expensive egos: Narcissistic males have higher cortisol. *PLoS ONE, 7*(1). doi:10.1371/journal.pone.0030858

Reis, N. A., Kowalski, K. C., Ferguson, L. J., Sabiston, C. M., Sedgwick, W. A., & Crocker, P. R. E. (2015). Self-compassion and women athletes' responses to emotionally difficult sport situations: An evaluation of a brief induction. *Psychology of Sport and Exercise, 16*(P3), 18-25. doi:10.1016/j.psychsport.2014.08.011

Rentzsch, K., & Gebauer, J. E. (2019). On the Popularity of Agentic and Communal Narcissists: The Tit-for-Tat Hypothesis. *Personality and Social Psychology Bulletin, 45*(9), 1365-1377. doi:10.1177/0146167218824359

Richardson, K., Hart, W., & Kinrade, C. (2020). Investigating how self-esteem moderates grandiose narcissism's interpersonal orientation. *Journal of Personality*. doi:10.1111/jopy.12612

Richardson, K., Hart, W., Tortoriello, G. K., & Breeden, C. J. (2021). An interaction model for the role of self-evaluations and antagonistic pursuits in subjective well-being. *British Journal of Psychology, 112*(2), 493-518. doi:10.1111/bjop.12473

Riketta, M. (2008). "Who identifies with which group?" the motive-feature match principle and its limitations. *European Journal of Social Psychology, 38*(4), 715-735. doi:10.1002/ejsp.534

Roberts, R., Callow, N., Hardy, L., Woodman, T., & Thomas, L. (2010). Interactive effects of different visual imagery perspectives and narcissism on motor performance. *Journal of Sport and Exercise Psychology, 32*(4), 499-517. doi:10.1123/jsep.32.4.499

Roberts, R., Cooke, A., Woodman, T., Hupfeld, H., Barwood, C., & Manley, H. (2019). When the going gets tough, who gets going? An examination of the relationship between narcissism, effort, and performance. *Sport, Exercise, and Performance Psychology, 8*(1), 93-105. doi:10.1037/spy0000124

Roberts, R., Woodman, T., Lofthouse, S., & Williams, L. (2015). Not all players are equally motivated: The role of narcissism. *European Journal of Sport Science, 15*(6), 536-542. doi:10.1080/17461391.2014.987324

Roche, M. J., Pincus, A. L., Lukowitsky, M. R., Ménard, K. S., & Conroy, D. E. (2013). An integrative approach to the assessment of narcissism. *Journal of Personality Assessment, 95*(3), 237-248. doi:10.1080/00223891.2013.770400

Rogoza, R., Cieciuch, J., Strus, W., & Baran, T. (2019). Seeking a Common Framework for Research on Narcissism: An Attempt to Integrate the Different Faces of Narcissism Within the Circumplex of Personality Metatraits. *European Journal of Personality, 33*(4), 437-455. doi:10.1002/per.2206

Rohmann, E., Bierhoff, H. W., & Schmohr, M. (2011). Narcissism and perceived inequity in attractiveness in romantic relationships. *European Psychologist, 16*(4), 295-302. doi:10.1027/1016-9040/a000025

Rohmann, E., Brailovskaia, J., & Bierhoff, H. W. (2019). The framework of self-esteem: Narcissistic subtypes, positive/negative agency, and self-evaluation. *Current Psychology*. doi:10.1007/s12144-019-00431-6

Rohmann, E., Hanke, S., & Bierhoff, H. W. (2019). Grandiose and Vulnerable Narcissism in Relation to Life Satisfaction, Self-Esteem, and Self-Construal. *Journal of Individual Differences, 40*(4), 194-203. doi:10.1027/1614-0001/a000292

Rohmann, E., Neumann, E., Herner, M. J., & Bierhoff, H. W. (2012). Grandiose and vulnerable narcissism: Self-construal, attachment, and love in romantic relationships. *European Psychologist, 17*(4), 279-290. doi:10.1027/1016-9040/a000100

Rohmann, E., Brailovskaia, J., & Bierhoff, H. W. (2021). The framework of self-esteem: Narcissistic subtypes, positive/negative agency, and self-evaluation. *Current Psychology*, *40*(10), 4843-4850. <https://doi.org/10.1007/s12144-019-00431-6>

Rose, A. L., & Kocovski, N. L. (2020). The Social Self-Compassion Scale (SSCS): Development, Validity, and Associations with Indices of Well-Being, Distress, and Social Anxiety. *International Journal of Mental Health and Addiction*. doi:10.1007/s11469-020-00302-3

Rose, C., & Wilson, M. S. (2014). Perceptions of everyday deceptions: Individual differences in narcissism and psychopathy associated with black and white untruths. In *Handbook of the Psychology of Narcissism: Diverse Perspectives* (pp. 229-248).

Rose, K. C., & Anastasio, P. A. (2014). Entitlement is about 'others', narcissism is not: Relations to sociotropic and autonomous interpersonal styles. *Personality and Individual Differences, 59*, 50-53. doi:10.1016/j.paid.2013.11.004

Rose, P. (2007). Mediators of the Association Between Narcissism and Compulsive Buying: The Roles of Materialism and Impulse Control. *Psychology of Addictive Behaviors, 21*(4), 576-581. doi:10.1037/0893-164X.21.4.576

Roseborough, J. E. (2010). *Narcissism and the Belief in an Unjust World: The Influence of Personality on Perceptions of Injustice, Driving Anger, and Aggressive Driving*: York University.

Rosenthal, S. A., & Hooley, J. M. (2010). Narcissism assessment in social-personality research: Does the association between narcissism and psychological health result from a confound with self-esteem? *Journal of Research in Personality, 44*(4), 453-465. doi:10.1016/j.jrp.2010.05.008

Rosenthal, S. A., Hooley, J. M., Montoya, R. M., van der Linden, S. L., & Steshenko, Y. (2020). The Narcissistic Grandiosity Scale: A Measure to Distinguish Narcissistic Grandiosity From High Self-Esteem. *Assessment, 27*(3), 487-507. doi:10.1177/1073191119858410

Rovelli, P., & Curnis, C. (2020). The perks of narcissism: Behaving like a star speeds up career advancement to the CEO position. *Leadership Quarterly*. doi:10.1016/j.leaqua.2020.101489

Rovelli, P., Massis, A. D., & Gomez-Mejia, L. R. (2023). Are narcissistic CEOs good or bad for family firm innovation? *Human Relations*, *76*(5), 776-806. <https://doi.org/10.1177/00187267221076834>

Rowatt, W. C., Kang, L. L., Haggard, M. C., & LaBouff, J. P. (2014). A Social-Personality Perspective on Humility, Religiousness, and Spirituality. *Journal of Psychology and Theology, 42*(1), 31-40.

Rubinstein, G. (2014). Narcissism in the third millennium: Personality disorder or cultural phenomenon? In *Handbook of the Psychology of Narcissism: Diverse Perspectives* (pp. 105-123).

Rubinstein, G. (2016). Modesty doesn't become me: Narcissism and the big five among male and female candidates for the big brother TV show. *Journal of Individual Differences, 37*(4), 223-230. doi:10.1027/1614-0001/a000209

Rubenstein, A. L., Xia, Y. H., Lan, Y. Y., Morrison, H. M., & Newton, D. W. (2023). Strengthening Supervisor Bonds but Impairing Coworker Relations? The Divergent Effects of Voice Endorsement. *Journal of Management*, 26. <https://doi.org/10.1177/01492063231213657>

Ruggiero, G. M., Veronese, G., Castiglioni, M., Procaccia, R., & Sassaroli, S. (2017). Cognitive avoidance, humiliation and narcissism in non-clinical individuals: An experimental study. In *Advances in Psychology Research* (Vol. 128, pp. 1-16).

Ruiz, J. M., Smith, T. W., & Rhodewalt, F. (2001). Distinguishing narcissism and hostility: Similarities and differences in interpersonal circumplex and five-factor correlates. *Journal of Personality Assessment, 76*(3), 537-555. doi:10.1207/S15327752JPA7603_12

Sabouri, S., Gerber, M., Bahmani, D. S., Lemola, S., Clough, P. J., Kalak, N., . . . Brand, S. (2016). Examining Dark Triad traits in relation to mental toughness and physical activity in young adults. *Neuropsychiatric Disease and Treatment, 12*, 229-235. doi:10.2147/NDT.S97267

Safarzadeh, M. H., & Mohammadian, M. A. (2023). Auditors' narcissism and their professional skepticism: evidence from Iran. *Asian Review of Accounting*, 29. <https://doi.org/10.1108/ara-12-2022-0284>

Sakalaki, M., & Sotiriou, P. (2012). Pro-self orientation and preference for deceitful strategies: Social value orientation, dispositional and behavioral correlates of economic opportunism. *Studia Psychologica, 54*(2), 157-165. Retrieved from https://www.scopus.com/inward/record.uri?eid=2-s2.0-84865702391&partnerID=40&md5=13a136f36a6e95528d8ec0cab71cb2c5

Sakkar Sudha, K., & Shahnawaz, M. G. (2020). Narcissism personality trait and performance: task-oriented leadership and authoritarian styles as mediators. *Leadership and Organization Development Journal, 41*(2), 280-293. doi:10.1108/LODJ-09-2019-0399

Saleem, M., Anderson, C. A., & Barlett, C. P. (2015). Assessing Helping and Hurting Behaviors Through the Tangram Help/Hurt Task. *Personality and Social Psychology Bulletin, 41*(10), 1345-1362. doi:10.1177/0146167215594348

Saleem, M., Barlett, C. P., Anderson, C. A., & Hawkins, I. (2017). Helping and hurting others: Person and situation effects on aggressive and prosocial behavior as assessed by the Tangram task. *Aggressive Behavior, 43*(2), 133-146. doi:10.1002/ab.21669

Sanecka, E. (2021). Grandiose and vulnerable narcissism and regulatory focus at work in relation to strengths use and deficit correction in the workplace. *Plos One*, *16*(10 October). <https://doi.org/10.1371/journal.pone.0258609>

Samuel, D. B., & Widiger, T. A. (2008). Convergence of narcissism measures from the perspective of general personality functioning. *Assessment, 15*(3), 364-374. doi:10.1177/1073191108314278

Schmid, E. A., Knipfer, K., & Peus, C. V. (2021). Narcissistic Leaders–Promise or Peril? The Patterns of Narcissistic Leaders’ Behaviors and Their Relation to Team Performance. *Frontiers in Psychology*, *12*. <https://doi.org/10.3389/fpsyg.2021.660452>

Schmitt, D. P., Alcalay, L., Allik, J., Alves, I. C. B., Anderson, C. A., Angelini, A. L., . . . Zupančič, A. (2017). Narcissism and the strategic pursuit of short-term mating: Universal links across 11 world regions of the international sexuality description project-2. *Psihologijske Teme, 26*(1), 89-137. doi:10.31820/pt.26.1.5

Schnieders, T. C., & Gore, J. S. (2011). We don't want your kind here: When people high in narcissism show prejudice against immigrants. *Journal of Social, Evolutionary, and Cultural Psychology, 5*(3), 175-193. doi:10.1037/h0099265

Schoenleber, M., Johnson, L. R., & Berenbaum, H. (2023). Self-conscious emotion traits & reactivity in narcissism. *Current Psychology*, 13. <https://doi.org/10.1007/s12144-023-05256-y>

Schnure, K. A. (2013). *Narcissism and its measurement: A conditional reasoning measure for narcissism.* Georgia Tech, Retrieved from http://oatd.org/oatd/record?record=handle\:1853\%2F52156&q=%20%28NPI%29%20OR%20%28Narcissistic%20AND%20Personality%20AND%20Inventory%29%20

Schreer, G. E. (2002). Narcissism and aggression: Is inflated self-esteem related to aggressive driving. *North American Journal of Psychology, 4*(3), 333-342.

Schriber, R. A., Chung, J. M., Sorensen, K. S., & Robins, R. W. (2017). Dispositional contempt: A first look at the contemptuous person. *Journal of Personality and Social Psychology, 113*(2), 280-309. doi:10.1037/pspp0000101

Schröder-Abé, M., & Fatfouta, R. (2019). Shades of narcissistic dishonesty: Grandiose versus vulnerable narcissism and the role of self-conscious emotions. *Journal of Economic Psychology, 71*, 148-158. doi:10.1016/j.joep.2018.06.003

Schütz, A., Marcus, B., & Sellin, I. (2004). Measuring narcissism as a personality construct: Psychometric properties of a long and a short version of the German Narcissistic Personality Inventory. *Diagnostica, 50*(4), 202-218. doi:10.1026/0012-1924.50.4.202

Schyns, B., Braun, S. H., & Xia, Y. (2023). What motivates narcissistic individuals to lead? The role of identity across cultures. *Personality and Individual Differences*, *206*, 9. <https://doi.org/10.1016/j.paid.2023.112107>

Scott, G. G., Boyle, E. A., Czerniawska, K., & Courtney, A. (2018). Posting photos on Facebook: The impact of Narcissism, Social Anxiety, Loneliness, and Shyness. *Personality and Individual Differences, 133*, 67-72. doi:10.1016/j.paid.2016.12.039

Sedikides, C., Rudich, E. A., Gregg, A. P., Kumashiro, M., & Rusbult, C. (2004). Are normal narcissists psychologically healthy?: Self-esteem matters. *Journal of Personality and Social Psychology, 87*(3), 400-416. doi:10.1037/0022-3514.87.3.400

Selcuk, F. U., & Gungor, N. D. (2022). Narcissism and Political Left-Right Orientation in View of Basic Human Values: A Sample of Faculty of Management Students From Turkey. *Changing Societies & Personalities*, *6*(4), 764-784. <https://doi.org/10.15826/csp.2022.6.4.202>

Selle, K., Brown, A. A., El-Alayli, A., & Ewert, S. (2019). Grandiose Narcissists’ Public Versus Private Attributions for a Collaborative Success. *Current Psychology, 38*(2), 497-503. doi:10.1007/s12144-017-9628-2

Semenyna, S. W., & Honey, P. L. (2015). Dominance styles mediate sex differences in Dark Triad traits. *Personality and Individual Differences, 83*, 37-43. doi:10.1016/j.paid.2015.03.046

Set, Z. (2021). Mediating role of narcissism, vulnerable narcissism, and self-compassion in the relationship between attachment dimensions and psychopathology. *Anadolu Psikiyatri Dergisi*, *22*(3), 147-152. <https://doi.org/10.5455/apd.99551>

Shah, M., Sarfraz, M., Khawaja, K. F., & Tariq, J. (2020). Does narcissism encourage unethical pro-organizational behavior in the service sector? A case study in Pakistan. *Global Business and Organizational Excellence, 40*(1), 44-57. doi:10.1002/joe.22062

Sharpe, E. E., Schofield, M. B., Roberts, B. H., Kamal, A., & Maratos, F. A. (2023). Exploring the role of compassion, self-criticism and the dark triad on obesity and emotion regulation. *Current Psychology*, 11. <https://doi.org/10.1007/s12144-023-05319-0>

She, Z., Li, Q., London, M., Yang, B., & Yang, B. (2019). Effects of CEO narcissism on decision-making comprehensiveness and speed. *Journal of Managerial Psychology, 35*(1), 42-55. doi:10.1108/JMP-01-2019-0042

Sheldon, K. M., Sedikides, C., Ntoumanis, N., Corcoran, M., & Titova, L. (2020). Narcissism and social motives: Successful pursuit of egosystem goals boosts narcissism. *Self and Identity, 19*(7), 841-862. doi:10.1080/15298868.2019.1690036

Sherman, R. A., Nave, C. S., & Funder, D. C. (2013). Situational construal is related to personality and gender. *Journal of Research in Personality, 47*(1), 1-14. doi:10.1016/j.jrp.2012.10.008

Sherry, S. B., Gralnick, T. M., Hewitt, P. L., Sherry, D. L., & Flett, G. L. (2014). Perfectionism and narcissism: Testing unique relationships and gender differences. *Personality and Individual Differences, 61-62*, 52-56. doi:10.1016/j.paid.2014.01.007

Shi, Y., Luo, Y. L. L., Yang, Z., Liu, Y., & Bao, H. (2018). Do narcissists enjoy visiting social networking sites? It depends on how adaptive they are. *Frontiers in Psychology, 9*(SEP). doi:10.3389/fpsyg.2018.01739

Shi, L. P., Sun, S. J., & Geng, Y. G. (2022). Differential Eating Behavior Patterns among the Dark Triad. *International Journal of Environmental Research and Public Health*, *19*(12), 10. <https://doi.org/10.3390/ijerph19127062>

Shimberg, J., Josephs, L., & Grace, L. (2016). Empathy as a Mediator of Attitudes Toward Infidelity Among College Students. *Journal of Sex and Marital Therapy, 42*(4), 353-368. doi:10.1080/0092623X.2015.1053019

Shoikhedbrod, A., Ward Struthers, C., Guilfoyle, J. R., van Monsjou, E., Halilova, J., & Saleemi, S. (2019). How, when, and why transgressors’ narcissism affects motivation to apologize (or not). *Journal of Research in Personality, 78*, 36-51. doi:10.1016/j.jrp.2018.11.003

Shulman, D. G., & Ferguson, G. R. (1988). An experimental investigation of Kernberg's and Kohut's theories of narcissism. *Journal of Clinical Psychology, 44*(3), 445-451. doi:10.1002/1097-4679(198805)44:3<445::AID-JCLP2270440322>3.0.CO;2-Z

Siedor, L., Maples-Keller, J. L., Miller, J. D., & Keith Campbell, W. (2016). Narcissism and Hypomania Revisited: a Test of the Similarities and Differences in Their Empirical Networks. *Current Psychology, 35*(2), 244-254. doi:10.1007/s12144-016-9408-4

Siem, B., Kretzmeyer, B., & Stürmer, S. (2021). The role of self-evaluation in predicting attitudes toward supporters of COVID-19-related conspiracy theories: A direct and a conceptual replication of Cichoka et al. (2016). *Journal of Pacific Rim Psychology*, *15*, 14. <https://doi.org/10.1177/18344909211052587>

Simpson, A. J., Hermann, A. D., Lehtman, M. J., & Fuller, R. C. (2016). Interpersonal Transgressions and Interest in Spiritual Activities: The Role of Narcissism. *Current Psychology, 35*(2), 195-206. doi:10.1007/s12144-015-9393-z

Singh, S., Farley, S. D., & Donahue, J. J. (2018). Grandiosity on display: Social media behaviors and dimensions of narcissism. *Personality and Individual Differences, 134*, 308-313. doi:10.1016/j.paid.2018.06.039

Skues, J. L., Williams, B., & Wise, L. (2012). The effects of personality traits, self-esteem, loneliness, and narcissism on Facebook use among university students. *Computers in Human Behavior, 28*(6), 2414-2419. doi:10.1016/j.chb.2012.07.012

Smith, B. M. (1990). THE MEASUREMENT OF NARCISSISM IN ASIAN, CAUCASIAN, AND HISPANIC AMERICAN WOMEN. *Psychological Reports, 67*(3), 779-785. doi:10.2466/PR0.67.7.779-785

Song, G. H. Y. (2021). How Does Job Insecurity Affect Workplace Harassment? The Interaction Effect of Hypercompetitive Attitude, Coworker Impression Management, and Leader Narcissism. *Frontiers in Psychology*, *12*, 11. <https://doi.org/10.3389/fpsyg.2021.753061>

Sommer, K. L., Kirkland, K. L., Newman, S. R., Estrella, P., & Andreassi, J. L. (2009). Narcissism and cardiovascular reactivity to rejection imagery. *Journal of Applied Social Psychology, 39*(5), 1083-1115. doi:10.1111/j.1559-1816.2009.00473.x

Spano, L. (2001). The relationship between exercise and anxiety, obsessive-compulsiveness, and narcissism. *Personality and Individual Differences, 30*(1), 87-93. doi:10.1016/S0191-8869(00)00012-X

Spielmann, S. S., & Gahman, K. P. (2020). Detectability and desirability of fear of being single in online dating profiles. *Journal of Personality*. doi:10.1111/jopy.12597

Starlinger, A., Voracek, M., & Tran, U. S. (2022). Vulnerable narcissism and the dark factor of personality: Insights from a cross-validated item-level and scale-level factor-analytic approach. *Personality and Individual Differences*, *185*, 8. <https://doi.org/10.1016/j.paid.2021.111283>

Stanescu, D. F., & Mohorea, L. (2015). THE DARK TRIAD OF PERSONALITY IN ORGANIZATIONAL LIFE A CORRELATIONAL STUDY WITH COUNTERPRODUCTIVE WORK BEHAVIOUR AND WORK LOCUS OF CONTROL. In C. Bratianu, A. Zbuchea, F. Pinzaru, E. M. Vatamanescu, & R. D. Leon (Eds.), *STRATEGICA: LOCAL VERSUS GLOBAL* (pp. 555-561).

Stangor, C., & Thompson, E. P. (2002). Needs for cognitive economy and self-enhancement as unique predictors of intergroup attitudes. *European Journal of Social Psychology, 32*(4), 563-575. doi:10.1002/ejsp.114

Steffens, N. K., & Haslam, S. A. (2020). The narcissistic appeal of leadership theories. *American Psychologist*. doi:10.1037/amp0000738

Steffens, N. K., & Haslam, S. A. (2022). The Narcissistic Appeal of Leadership Theories. *American Psychologist*, *77*(2), 234-248. <https://doi.org/10.1037/amp0000738>

Steffgen, G. (2007). Do narcissism and clarity of self-concept affect aggressive driving behavior in ego-threatening situations? *Zeitschrift fur Sozialpsychologie, 38*(1), 43-52. doi:10.1024/0044-3514.38.1.43

Steininger, B., & Pietschnig, J. (2022). Evidence for the superordinate predictive ability of trait psychopathy: The Dark Triad and quality of sexual life. *Personality and Individual Differences*, *193*, 5. <https://doi.org/10.1016/j.paid.2022.111620>

Strelan, P. (2007). Who forgives others, themselves, and situations? The roles of narcissism, guilt, self-esteem, and agreeableness. *Personality and Individual Differences, 42*(2), 259-269. doi:10.1016/j.paid.2006.06.017

Stucke, T. S. (2001). The Relationship between Self-Concept and Self-Reported Aggressive Driving Behavior. *Zeitschrift fur Sozialpsychologie, 32*(4), 261-273. doi:10.1024//0044-3514.32.4.261

Stucke, T. S. (2002). Narcissism and self-concept clarity as personality correlates of mobbing perpetrators. *Zeitschrift fur Arbeits- und Organisationspsychologie, 46*(4), 216-221. doi:10.1026//0932-4089.46.4.216

Stucke, T. S., & Sporer, S. L. (2002). When a grandiose self-image is threatened: Narcissism and self-concept clarity as predictors of negative emotions and aggression following ego-threat. *Journal of Personality, 70*(4), 509-532. doi:10.1111/1467-6494.05015

Sturman, T. S. (2000). The motivational foundations and behavioral expressions of three narcissistic styles. *Social Behavior and Personality, 28*(4), 393-407. doi:10.2224/sbp.2000.28.4.393

Sullivan, B. F., & Geaslin, D. L. (2001). The role of narcissism, self-esteem, and irrational beliefs in predicting aggression. *Journal of Social Behavior and Personality, 16*(1), 53-68. Retrieved from <https://www.scopus.com/inward/record.uri?eid=2-s2.0-24044501341&partnerID=40&md5=e7b2e7ebbce869a9c47b3eebac3a389e>

Subra, B. (2023). Why narcissists are more likely to be aggressive? The role of hostile attribution bias. *International Journal of Psychology*, *58*(6), 518-525. <https://doi.org/10.1002/ijop.12924>

Sumanth, J. J., & Cable, D. M. (2011). Status And Organizational Entry: How Organizational And Individual Status Affect Justice Perceptions Of Hiring Systems. *Personnel Psychology, 64*(4), 963-1000. doi:10.1111/j.1744-6570.2011.01233.x

Sung, Y., Kim, E., & Choi, S. M. (2018). #Me and brands: understanding brand-selfie posters on social media. *International Journal of Advertising, 37*(1), 14-28. doi:10.1080/02650487.2017.1368859

Sung, Y., Lee, J. A., Kim, E., & Choi, S. M. (2016). Why we post selfies: Understanding motivations for posting pictures of oneself. *Personality and Individual Differences, 97*, 260-265. doi:10.1016/j.paid.2016.03.032

Sutin, A. R., & Robins, R. W. (2005). Continuity and correlates of emotions and motives in self-defining memories. *Journal of Personality, 73*(3), 793-824. doi:10.1111/J.1467-6494.2005.00329.x

Sutin, A. R., & Robins, R. W. (2008). Going forward by drawing from the past: Personal strivings, personally meaningful memories, and personality traits. *Journal of Personality, 76*(3), 631-663. doi:10.1111/j.1467-6494.2008.00499.x

Svindseth, M. F., SØrebØ, Ø., NØttestad, J. A., Roaldset, J. O., Wallin, J., & Dahl, A. A. (2009). Psychometric examination and normative data for the Narcissistic Personality Inventory 29 item version: Personality and Social Sciences. *Scandinavian Journal of Psychology, 50*(2), 151-159. doi:10.1111/j.1467-9450.2008.00686.x

Swami, V., Cass, L., Waseem, M., & Furham, A. (2015). What is the relationship between facets of narcissism and women's body image? *Personality and Individual Differences, 87*, 185-189. doi:10.1016/j.paid.2015.08.006

Sweeten, G. J. D., Jeske, D., & Lin, R. (2017). *Narcissism: A factor behind the selective sharing of news online*.

Szabó, E., & Bereczkei, T. (2017). Different paths to different strategies? Unique associations among facets of the Dark Triad, empathy, and trait emotional intelligence. *Advances in Cognitive Psychology, 13*(4), 306-313. doi:10.5709/acp-0230-7

Tanrikulu, I., & Erdur-Baker, O. (2021). Motives Behind Cyberbullying Perpetration: A Test of Uses and Gratifications Theory. *Journal of Interpersonal Violence*, *36*(13-14). <https://doi.org/10.1177/0886260518819882>

Trahair, C., MacDonald, K. B., Furnham, A., & Schermer, J. A. (2022). Altruism and the Dark Triad. *Current Issues in Personality Psychology*, *10*(3), 234-239. <https://doi.org/10.5114/cipp.2022.113436>

Taylor, C. M. (1995). *The Profile of Narcissistic Dispositions (POND): development and validation.* University of British Columbia,

Thomas, J., Hashmi, A. A., Chung, M. C., Morgan, K., & Lyons, M. (2013). The narcissistic mask: An exploration of 'the defensive grandiosity hypothesis'. *Personality and Mental Health, 7*(2), 160-167. doi:10.1002/pmh.1219

Thomas, L. B., Fadeeva, A., & Oliver, E. J. (2020). The double negative: Personality differentially predicts sensitivity to need support and thwarting, and subsequent behavioural response planning. *Personality and Individual Differences, 156*. doi:10.1016/j.paid.2019.109767

Tisaker, N. (2016). *Depression, self-esteem and narcissism and its association with Facebook use.* University of Cape Town,

Tobacyk, J. J., & Mitchell, T. E. (1987). OUT-OF-BODY EXPERIENCE STATUS AS A MODERATOR OF EFFECTS OF NARCISSISM ON PARANORMAL BELIEFS. *Psychological Reports, 60*(2), 440-442. doi:10.2466/pr0.1987.60.2.440

Toirova, M., & Baek, Y. Why narcissistic individuals engage in unethical pro-organizational behavior. Testing a moderated mediation model. *Asia-Pacific Journal of Business Administration*. doi:10.1108/APJBA-11-2020-0396

Tortoriello, G. K., & Hart, W. (2018). A tale of two audiences: Narcissism, failure reactivity, and perceived criticism from the self and others as internalized audiences. *Self and Identity, 17*(2), 236-254. doi:10.1080/15298868.2017.1382385

Tortoriello, G. K., & Hart, W. (2019). Blurring the Dichotomy of Good and Evil: The Idiosyncratic Helping Strategies Associated with Unmitigated-Agentic and Unmitigated-Communal Personalities. *European Journal of Personality, 33*(6), 674-701. doi:10.1002/per.2223

Tortoriello, G. K., Hart, W., Richardson, K., & Tullett, A. M. (2017). Do narcissists try to make romantic partners jealous on purpose? An examination of motives for deliberate jealousy-induction among subtypes of narcissism. *Personality and Individual Differences, 114*, 10-15. doi:10.1016/j.paid.2017.03.052

Traiser, S., & Eighmy, M. A. (2011). Moral Development and Narcissism of Private and Public University Business Students. *Journal of Business Ethics, 99*(3), 325-334. doi:10.1007/s10551-011-0809-x

Tran, U. S., Bertl, B., Kossmeier, M., Pietschnig, J., Stieger, S., & Voracek, M. (2018). “I'll teach you differences”: Taxometric analysis of the Dark Triad, trait sadism, and the Dark Core of personality. *Personality and Individual Differences, 126*, 19-24. doi:10.1016/j.paid.2018.01.015

Trechera, J. L., Millán Vásquez de la Torre, G., & Fernández Morales, E. (2009). An empirical study of narcissistic personality disorder (NPD). *Acta Colombiana de Psicologia, 11*(2), 25-36. Retrieved from https://www.scopus.com/inward/record.uri?eid=2-s2.0-75249107060&partnerID=40&md5=c3b6b92b89810476e490735ff5206e60

Trent, N. L., Beauregard, M., & Schwartz, G. E. (2020). Preliminary development and validation of a scale to measure universal love. *Spirituality in Clinical Practice, 7*(1), 51-64. doi:10.1037/scp0000198

Trombly, D. R. C., & Zeigler-Hill, V. (2017). The Dark Triad and Disordered Gambling. *Current Psychology, 36*(4), 740-746. doi:10.1007/s12144-016-9461-z

Truhan, T. E., Wilson, P., Mõttus, R., & Papageorgiou, K. A. (2021). The many faces of dark personalities: An examination of the Dark Triad structure using psychometric network analysis. *Personality and Individual Differences, 171*. doi:10.1016/j.paid.2020.110502

Tschanz, B. T., Morf, C. C., & Turner, C. W. (1998). Gender differences in the structure of narcissism: A multi-sample analysis of the narcissistic personality inventory. *Sex Roles, 38*(9-10), 863-870. doi:10.1023/a:1018833400411

Tudose, L. M. (2022). The relationship between dark triad and emotional intelligence. *Anthropological Researches and Studies*, *2022*(12), 256-267. <https://doi.org/10.26758/12.1.19>

Turel, O., & Gil-Or, O. (2019). To share or not to share? The roles of false Facebook self, sex, and narcissism in re-posting self-image enhancing products. *Personality and Individual Differences, 151*. doi:10.1016/j.paid.2019.109506

Turner, I. N., Foster, J. D., & Webster, G. D. (2019). The Dark Triad's inverse relations with cognitive and emotional empathy: High-powered tests with multiple measures. *Personality and Individual Differences, 139*, 1-6. doi:10.1016/j.paid.2018.10.030

Türkmen, A., & Aytac, M. B. (2023). The role of overt and covert narcissism in virtual goods purchase motivations and intention. *Electronic Commerce Research*, 31. <https://doi.org/10.1007/s10660-023-09724-2>

Twenge, J. M., & Campbell, W. K. (2003). "Isn't it fun to get the respect that we're going to deserve?" Narcissism, social rejection, and aggression. *Personality and Social Psychology Bulletin, 29*(2), 261-272. doi:10.1177/0146167202239051

Twenge, J. M., Campbell, W. K., & Freeman, E. C. (2012). Generational differences in young adults' life goals, concern for others, and civic orientation, 1966-2009. *Journal of Personality and Social Psychology, 102*(5), 1045-1062. doi:10.1037/a0027408

Tylka, T. L., & Iannantuono, A. C. (2016). Perceiving beauty in all women: Psychometric evaluation of the Broad Conceptualization of Beauty Scale. *Body Image, 17*, 67-81. doi:10.1016/j.bodyim.2016.02.005

Ubaradka, A., Fathima, A., & Batra, S. (2023). Psychological Correlates of Perfectionistic Self-Presentation Among Social Media Users. *International Journal of Cyber Behavior, Psychology and Learning*, *13*(1). <https://doi.org/10.4018/IJCBPL.324089>

Uji, M., Yukihiro, T., Adachi, K., & Kitamura, T. (2014). Narcissistic grandiose self: Its defensive function against depressive mood and the damage it causes in the context of negative interpersonal life events. In *Handbook of the Psychology of Narcissism: Diverse Perspectives* (pp. 215-226).

Umegaki, Y., & Higuchi, A. (2022). Personality traits and mental health of social networking service users: A cross-sectional exploratory study among Japanese undergraduates. *Computers in Human Behavior Reports*, *6*. <https://doi.org/10.1016/j.chbr.2022.100177>

Unger-Aviram, E., Zeigler-Hill, V., Barina, M., & Besser, A. (2018). Narcissism, collective efficacy, and satisfaction in self-managed teams: The moderating role of team goal orientation. *Group Dynamics, 22*(3), 172-186. doi:10.1037/gdn0000089

Uppal, N. (2020). CEO narcissism, CEO duality, TMT agreeableness and firm performance: An empirical investigation in auto industry in India. *European Business Review, 32*(4), 573-590. doi:10.1108/EBR-06-2019-0121

Utz, S., Muscanell, N., & Göritz, A. S. (2014). Give, match, or take: A new personality construct predicts resource and information sharing. *Personality and Individual Differences, 70*, 11-16. doi:10.1016/j.paid.2014.06.011

Utz, S., Tanis, M., & Vermeulen, I. (2012). It is all about being popular: The effects of need for popularity on social network site use. *Cyberpsychology, Behavior, and Social Networking, 15*(1), 37-42. doi:10.1089/cyber.2010.0651

Uziel, L., & Cohen, B. (2020). Self-deception and discrepancies in self-evaluation. *Journal of Research in Personality, 88*. doi:10.1016/j.jrp.2020.104008

Vaal, S., Schofield, M. B., Baker, I. S., & Roberts, B. L. H. (2022). Narcissism, national narcissism, COVID-19 conspiracy belief, and social media use as predictors of compliance with COVID-19 public health guidelines. *Current Psychology*, 8. <https://doi.org/10.1007/s12144-022-03715-6>

Vaillancourt, T. (2013). Students Aggress Against Professors in Reaction to Receiving Poor Grades: An Effect Moderated by Student Narcissism and Self-Esteem. *Aggressive Behavior, 39*(1), 71-84. doi:10.1002/ab.21450

van Gerven, E. J. G., De Hoogh, A. H. B., Den Hartog, D. N., & Belschak, F. D. (2022). Gender Differences in the Perceived Behavior of Narcissistic Leaders. *Frontiers in Psychology*, *13*, 13, Article 809193. <https://doi.org/10.3389/fpsyg.2022.809193>

van der Linden, S., & Rosenthal, S. A. (2016). Measuring narcissism with a single question? A replication and extension of the Single-Item Narcissism Scale (SINS). *Personality and Individual Differences, 90*, 238-241. doi:10.1016/j.paid.2015.10.050

van Mulukom, V., Patterson, R. E., & van Elk, M. (2020). Broadening Your Mind to Include Others: The relationship between serotonergic psychedelic experiences and maladaptive narcissism. *Psychopharmacology, 237*(9), 2725-2737. doi:10.1007/s00213-020-05568-y

van Teffelen, M. W., Vancleef, L. M. G., & Lobbestael, J. (2021). Provoked aggression, psychopathy and narcissism: Comparing the impact of social exclusion and insult. *Psychology of Violence*, *11*(1), 82-91. <https://doi.org/10.1037/vio0000340>

Vater, A., Moritz, S., & Roepke, S. (2018). Does a narcissism epidemic exist in modern western societies? Comparing narcissism and self-esteem in East and West Germany. *PLoS ONE, 13*(1). doi:10.1371/journal.pone.0188287

Vaughan-Johnston, T. I., & Jacobson, J. A. (2020). “Need” personality constructs and preferences for different types of self-relevant feedback. *Personality and Individual Differences, 154*. doi:10.1016/j.paid.2019.109671

Vazire, S., Naumann, L. P., Rentfrow, P. J., & Gosling, S. D. (2008). Portrait of a narcissist: Manifestations of narcissism in physical appearance. *Journal of Research in Personality, 42*(6), 1439-1447. doi:10.1016/j.jrp.2008.06.007

Venema, T. A. G., & Pfattheicher, S. (2021). Perceived susceptibility to COVID-19 infection and narcissistic traits. *Personality and Individual Differences, 175*. doi:10.1016/j.paid.2021.110696

Veronica Smith, C., Hadden, B. W., Webster, G. D., Jonason, P. K., Gesselman, A. N., & Crysel, L. C. (2014). Mutually attracted or repulsed? Actor-partner interdependence models of Dark Triad traits and relationship outcomes. *Personality and Individual Differences, 67*, 35-41. doi:10.1016/j.paid.2014.01.044

Virk, P., & Kumari, S. (2023). Perspective Taking and Mindfulness as Mediators of the Relationship Between Maladaptive Narcissism and Empathy. *Social Behavior and Personality*, *51*(7), 13. <https://doi.org/10.2224/sbp.12413>

Vize, C. E., Collison, K. L., Miller, J. D., & Lynam, D. R. (2020). The "Core" of the Dark Triad: A Test of Competing Hypotheses. *Personality Disorders-Theory Research and Treatment, 11*(2), 91-99. doi:10.1037/per0000386

Volmer, J., Koch, I. K., & Wolff, C. (2019). Illuminating the ‘dark core’: Mapping global versus specific sources of variance across multiple measures of the dark triad. *Personality and Individual Differences, 145*, 97-102. doi:10.1016/j.paid.2019.03.024

Vonk, J., Zeigler-Hill, V., Mayhew, P., & Mercer, S. (2013). Mirror, mirror on the wall, which form of narcissist knows self and others best of all? *Personality and Individual Differences, 54*(3), 396-401. doi:10.1016/j.paid.2012.10.010

Wagstaff, D. L. (2018). Comparing mating motivations, social processes, and personality as predictors of women's cosmetics use. *Evolutionary Behavioral Sciences, 12*(4), 367-380. doi:10.1037/ebs0000119

Wai, M., & Tiliopoulos, N. (2012). The affective and cognitive empathic nature of the dark triad of personality. *Personality and Individual Differences, 52*(7), 794-799. doi:10.1016/j.paid.2012.01.008

Waldman, D. A., Wang, D., Hannah, S. T., & Balthazard, P. A. (2017). A neurological and ideological perspective of ethical leadership. *Academy of Management Journal, 60*(4), 1285-1306. doi:10.5465/amj.2014.0644

Waldman, D. A., Wang, D., Hannah, S. T., Owens, B. P., & Balthazard, P. A. (2018). Psychological and neurological predictors of abusive supervision. *Personnel Psychology, 71*(3), 399-421. doi:10.1111/peps.12262

Wales, W. J., Patel, P. C., & Lumpkin, G. T. (2013). In pursuit of greatness: CEO narcissism, entrepreneurial orientation, and firm performance variance. *Journal of Management Studies, 50*(6), 1041-1069. doi:10.1111/joms.12034

Wallace, H. M., & Baumeister, R. F. (2002). The performance of narcissists rises and falls with perceived opportunity for glory. *Journal of Personality and Social Psychology, 82*(5), 819-834. doi:10.1037//0022-3514.82.5.819

Wallace, H. M., Grotzinger, A., Howard, T. J., & Parkhill, N. (2015). When People Evaluate Others, the Level of Others’ Narcissism Matters Less to Evaluators Who Are Narcissistic. *Social Psychological and Personality Science, 6*(7), 805-813. doi:10.1177/1948550615587985

Wallace, H. M., Ready, C. B., & Weitenhagen, E. (2009). Narcissism and task persistence. *Self and Identity, 8*(1), 78-93. doi:10.1080/15298860802194346

Wallace, H. M., Scheiner, B. R. M., & Grotzinger, A. (2016). Grandiose Narcissism Predicts Willingness to Behave Badly, Without Proportional Tolerance for Others’ Bad Behavior. *Current Psychology, 35*(2), 234-243. doi:10.1007/s12144-016-9410-x

Wallace, H. M., Carrillo, A., & Kelley, J. (2022). Perceptions of narcissism in college professors. *Journal of Social Psychology*, 18. <https://doi.org/10.1080/00224545.2022.2050167>

Walsh, J. A., Krienert, J. L., & McAdams, S. (2021). A retrospective examination of bullying victimisation during high school: Exploring narcissism deficits and empathy. In *Empathy versus Offending, Aggression and Bullying: Advancing Knowledge using the Basic Empathy Scale* (pp. 211-223).

Wang, C. H., & Lopez-Fernandez, O. (2019). Shades of foods: Prevalence and correlates of food addiction. *Aloma, 37*(1), 21-34. Retrieved from https://www.scopus.com/inward/record.uri?eid=2-s2.0-85066846303&partnerID=40&md5=fb10cb489613dfc95c16547cf151e164

Wang, D. (2017). A study of the relationship between narcissism, extraversion, drive for entertainment, and narcissistic behavior on social networking sites. *Computers in Human Behavior, 66*, 138-148. doi:10.1016/j.chb.2016.09.036

Wang, D. (2019). A study of the relationship between narcissism, extraversion, body-esteem, social comparison orientation and selfie-editing behavior on social networking sites. *Personality and Individual Differences, 146*, 127-129. doi:10.1016/j.paid.2019.04.012

Wang, L., Owens, B. P., Jason Li, J., & Shi, L. (2018). Exploring the affective impact, boundary conditions, and antecedents of leader humility. *Journal of Applied Psychology, 103*(9), 1019-1038. doi:10.1037/apl0000314

Wang, Q., Zhang, Z., Song, P., Liu, Z., Zhang, Q., Vivino, A. A., . . . Hu, P. (2021). Factor structure and construct validity of the short form of managing the emotions of others (MEOS-SF) scale in the Chinese sample. *PLoS ONE, 16*(4 April). doi:10.1371/journal.pone.0249774

Wang, R., & Jiang, J. (2014). How narcissistic employees respond to abusive supervision: Two roles of narcissism in decreasing perception and increasing deviance. *Psychological Reports, 115*(2), 372-380. doi:10.2466/01.21.PR0.115c22z2

Wang, S. S., & Stefanone, M. A. (2013). Showing Off? Human Mobility and the Interplay of Traits, Self-Disclosure, and Facebook Check-Ins. *Social Science Computer Review, 31*(4), 437-457. doi:10.1177/0894439313481424

Wang, Y., Xie, X., Wang, X., Wang, P., Nie, J., & Lei, L. (2020). Narcissism and selfie-posting behavior: the mediating role of body satisfaction and the moderating role of attitude toward selfie-posting behavior. *Current Psychology, 39*(2), 665-672. doi:10.1007/s12144-018-9795-9

Wang, L., & Guo, Q. (2022). How Narcissistic Leaders Impact on Subordinate's Followership During the COVID-19? The Moderating Role of Organizational Identification. *Frontiers in Psychology*, *13*, 10, Article 858779. <https://doi.org/10.3389/fpsyg.2022.858779>

Wang, Z., Yang, S., Li, X., Liu, P., & Liu, N. (2024). To share or not to share? A double-edge effect of narcissism on knowledge sharing in hospitality. *International Journal of Hospitality Management*, *117*. <https://doi.org/10.1016/j.ijhm.2023.103637>

Wardecker, B. M., Chopik, W. J., LaBelle, O. P., & Edelstein, R. S. (2018). Is narcissism associated with baseline cortisol in men and women? *Journal of Research in Personality, 72*, 44-49. doi:10.1016/j.jrp.2016.07.006

Watson, P. J., Hood, R. W., & Morris, R. J. (1984). RELIGIOUS ORIENTATION, HUMANISTIC VALUES, AND NARCISSISM. *Review of Religious Research, 25*(3), 257-264. doi:10.2307/3511123

Watson, P. J., Taylor, D., & Morris, R. J. (1987). Narcissism, sex roles, and self-functioning. *Sex Roles, 16*(7-8), 335-350. doi:10.1007/BF00289546

Webster, G. D., Kirkpatrick, L. A., Nezlek, J. B., Smith, C. V., & Paddock, E. L. (2007). Different slopes for different folks: Self-esteem instability and gender as moderators of the relationship between self-esteem and attitudinal aggression. *Self and Identity, 6*(1), 74-94. doi:10.1080/15298860600920488

Wehner, C., Ziegler, M., Gödeke, W., & Lämmle, L. (2021). Further inflaming the discussion or cooling down feelings? A network analysis of the Dark Triad and the Five Factor Model of personality. *Personality and Individual Differences, 175*. doi:10.1016/j.paid.2021.110717

Weidman, A. C., Cheng, J. T., & Tracy, J. L. (2018). The psychological structure of humility. *Journal of Personality and Social Psychology, 114*(1), 153-178. doi:10.1037/pspp0000112

Weidmann, R., Chopik, W. J., Ackerman, R. A., Allroggen, M., Bianchi, E. C., Brecheen, C., Campbell, W. K., Gerlach, T. M., Geukes, K., Grijalva, E., Grossmann, I., Hopwood, C. J., Hutteman, R., Konrath, S., Kuefner, A. C. P., Leckelt, M., Miller, J. D., Penke, L., Pincus, A. L., . . . Back, M. D. (2023). Age and gender gifferences in narcissism: A comprehensive study across eight measures and over 250,000 participants. *Journal of Personality and Social Psychology*, *124*(6), 1277-1298. <https://doi.org/10.1037/pspp0000463>

Weis-Rappaport, H., & Kluger, A. N. (2022). The effects of listening with "time-sharing" on psychological safety and social anxiety: the moderating role of narcissism and depression. *Journal of Social Psychology*, 12. <https://doi.org/10.1080/00224545.2022.2161337>

Weikel, K. A., Mowery Avara, R., Hanson, C. A., & Kater, H. (2010). College adjustment difficulties and the overt and covert forms of narcissism. *Journal of College Counseling, 13*(2), 100-110. doi:10.1002/j.2161-1882.2010.tb00052.x

Weinstock, J., Massura, C. E., & Petry, N. M. (2013). Professional and Pathological Gamblers: Similarities and Differences. *Journal of Gambling Studies, 29*(2), 205-216. doi:10.1007/s10899-012-9308-y

Weiser, E. B. (2015). #Me: Narcissism and its facets as predictors of selfie-posting frequency. *Personality and Individual Differences, 86*, 477-481. doi:10.1016/j.paid.2015.07.007

Werner, K. M., Smyth, A., & Milyavskaya, M. (2019). Do Narcissists Benefit from Materialistic Pursuits? Examining the Relation Between Narcissistic Tendencies, Extrinsic Goals, and Well-Being. *Collabra-Psychology, 5*(1). doi:10.1525/collabra.253

West, M. P., Miller, J. D., & Lynam, D. R. (2023). Comparing Brief Measures of Narcissism-Internal Consistency, Validity, and Coverage. *Journal of Personality Assessment*, 17. <https://doi.org/10.1080/00223891.2023.2183863>

Westerman, J. W., Bergman, J. Z., Bergman, S. M., & Daly, J. P. (2012). Are universities creating millennial narcissistic employees? an empirical examination of narcissism in business students and its implications. *Journal of Management Education, 36*(1), 5-32. doi:10.1177/1052562911408097

Westerman, J. W., Whitaker, B. G., Bergman, J. Z., Bergman, S. M., & Daly, J. P. (2016). Faculty narcissism and student outcomes in business higher education: A student-faculty fit analysis. *International Journal of Management Education, 14*(2), 63-73. doi:10.1016/j.ijme.2016.02.001

Wickens, C. M., Wiesenthal, D. L., & Roseborough, J. E. W. (2015). Personality predictors of driver vengeance. *Violence and Victims, 30*(1), 148-162. doi:10.1891/0886-6708.VV-D-13-00111

Widman, L., & McNulty, J. K. (2010). Sexual narcissism and the perpetration of sexual aggression. *Archives of Sexual Behavior, 39*(4), 926-939. doi:10.1007/s10508-008-9461-7

Wiegand, J. P. (2023). When overqualification turns dark: A moderated-mediation model of perceived overqualification, narcissism, frustration, and counterproductive work behavior. *Personality and Individual Differences*, *214*, Article 112351. <https://doi.org/10.1016/j.paid.2023.112351>

Wiehe, V. R. (2003). Empathy and narcissism in a sample of child abuse perpetrators and a comparison sample of foster parents. *Child Abuse and Neglect, 27*(5), 541-555. doi:10.1016/S0145-2134(03)00034-6

Will, G. J., Rutledge, R. B., Moutoussis, M., & Dolan, R. J. (2017). Neural and computational processes underlying dynamic changes in self-esteem. *eLife, 6*. doi:10.7554/eLife.28098

Willis, M., Birthrong, A., King, J. S., Nelson-Gray, R. O., & Latzman, R. D. (2017). Are infidelity tolerance and rape myth acceptance related constructs? An association moderated by psychopathy and narcissism. *Personality and Individual Differences, 117*, 230-235. doi:10.1016/j.paid.2017.06.015

Wilson, M. S., & Sibley, C. G. (2011). Narcissism creep?: Evidence for age-related differences in narcissism in the New Zealand general population. *New Zealand Journal of Psychology, 40*(3), 89-95. Retrieved from https://www.scopus.com/inward/record.uri?eid=2-s2.0-82355184590&partnerID=40&md5=47a256e7f29a7583e9acdab04e8fc192

Wink, P., & Gough, H. G. (1990). New Narcissism Scales for the California Psychological Inventory and MMPI. *Journal of Personality Assessment, 54*(3-4), 446-462. doi:10.1080/00223891.1990.9674010

Winter, S., Neubaum, G., Eimler, S. C., Gordon, V., Theil, J., Herrmann, J., . . . Krämer, N. C. (2014). Another brick in the Facebook wall - How personality traits relate to the content of status updates. *Computers in Human Behavior, 34*, 194-202. doi:10.1016/j.chb.2014.01.048

Wirtz, N., & Rigotti, T. (2020). When grandiose meets vulnerable: narcissism and well-being in the organizational context. *European Journal of Work and Organizational Psychology, 29*(4), 556-569. doi:10.1080/1359432X.2020.1731474

Witt, E. A., & Donnellan, M. B. (2008). Furthering the case for the MPQ-based measures of psychopathy. *Personality and Individual Differences, 45*(3), 219-225. doi:10.1016/j.paid.2008.04.002

Witt, E. A., Donnellan, M. B., Blonigen, D. M., Krueger, R. F., & Conger, R. D. (2009). Assessment of fearless dominance and impulsive antisociality via normal personality measures: Convergent validity, criterion validity, and developmental change. *Journal of Personality Assessment, 91*(3), 265-276. doi:10.1080/00223890902794317

Womick, J., Foltz, R. M., & King, L. A. (2019). “Releasing the beast within”? Authenticity, well-being, and the Dark Tetrad. *Personality and Individual Differences, 137*, 115-125. doi:10.1016/j.paid.2018.08.022

Wood, A. D., Borja, K., & Hoke, L. (2020). Narcissism for Fun and Profit: An Empirical Examination of Narcissism and Its Determinants in a Sample of Generation Z Business College Students. *Journal of Management Education*. doi:10.1177/1052562920965626

Woodman, T., Roberts, R., Hardy, L., Callow, N., & Rogers, C. H. (2011). There is an “i” in team: Narcissism and social loafing. *Research Quarterly for Exercise and Sport, 82*(2), 285-290. doi:10.1080/02701367.2011.10599756

Wright, K., & Furnham, A. (2015). How to spot a narcissist: Mental health literacy with respect to Narcissistic Personality Disorder. *Personality and Mental Health, 9*(2), 150-161. doi:10.1002/pmh.1277

Wu, K., Chen, C., & Greenberger, E. (2019). Nice guys and gals can finish first: Personality and speed-dating success among Asian Americans. *Journal of Social and Personal Relationships, 36*(8), 2507-2527. doi:10.1177/0265407518790103

Wu, M. S., Song, C., & Ma, Y. (2019). Selfie taking may be nonharmful: Evidence from adaptive and maladaptive narcissism among Chinese young adults. *Human Behavior and Emerging Technologies, 1*(3), 240-244. doi:10.1002/hbe2.166

Wu, Y. C. J., Chang, W. H., & Yuan, C. H. (2015). Do Facebook profile pictures reflect user's personality? *Computers in Human Behavior, 51*, 880-889. doi:10.1016/j.chb.2014.11.014

Xiao, X., & Su, Y. (2023). Stumble on information or misinformation? Examining the interplay of incidental news exposure, narcissism, and new media literacy in misinformation engagement. *Internet Research*, *33*(3), 1228-1248. <https://doi.org/10.1108/INTR-10-2021-0791>

Yang, M., Zhu, X., Sai, X., Zhao, F., Wu, H., & Geng, Y. (2019). The Dark Triad and sleep quality: Mediating role of anger rumination. *Personality and Individual Differences, 151*. doi:10.1016/j.paid.2019.06.027

Yadav, R., & Batra, S. (2023). Does Narcissism Influence Entrepreneurial Intentions? A Theory of Planned Behaviour Perspective. *Journal of Entrepreneurship*, *32*(2), 449-478. <https://doi.org/10.1177/09713557231184456>

Yang, W., Cun, L., Du, X., Yang, J., Wang, Y., Wei, D., . . . Qiu, J. (2015). Gender differences in brain structure and resting-state functional connectivity related to narcissistic personality. *Scientific Reports, 5*. doi:10.1038/srep10924

Yang, D. D., Wu, T. Y., Atkin, D. J., Rios, D. I., & Liu, Y. M. (2021). Social media portrait-editing intentions: Comparisons between Chinese and American female college students. *Telematics and Informatics*, *65*, 11. <https://doi.org/10.1016/j.tele.2021.101714>

Yang, N., Chen, H., Li, X., Yu, M. Y., & Wang, X. H. F. (2022). Leader status and team performance-the role of leader popularity and leader narcissism. *Current Psychology*, *42*(25), 21384-21396. <https://doi.org/10.1007/s12144-022-03240-6>

Ye, S., Lam, Z. K. W., Ma, Z., & Ng, T. K. (2016). Differential relations of narcissism and self-esteem to romantic relationships: The mediating role of perception discrepancy. *Asian Journal of Social Psychology, 19*(4), 374-384. doi:10.1111/ajsp.12160

Yilmaz, R., Yilmaz, F. G. K., & Avci, U. (2023). Examining the role of cyberloafing, narcissism, locus of control, and social appearance anxiety on the Internet gaming disorder in university students. *Psychology in the Schools*, *60*(8), 3040-3055. <https://doi.org/10.1002/pits.22894>

You, S. Y., Li, Z. Y., Jia, L. D., & Cai, Y. H. (2023). CEO narcissism and innovation ambidexterity: The moderating roles of CEO power and firm reputation. *Journal of Product Innovation Management*, *40*(2), 175-194. <https://doi.org/10.1111/jpim.12653>

Younus, A., Qureshi, M. A., Griffith, J., O'Riordan, C., & Pasi, G. (2016). *A study into the correlation between narcissism and facebook communication patterns*.

Yuk, H., Garrett, T. C., & Hwang, E. (2021). Effects of Grandiose and Vulnerable Narcissism on Donation Intentions: The Moderating Role of Donation Information Openness. *Sustainability*, *13*(13), 11. <https://doi.org/10.3390/su13137280>

Zacher, H., Pearce, L. K., Rooney, D., & McKenna, B. (2014). Leaders' Personal Wisdom and Leader-Member Exchange Quality: The Role of Individualized Consideration. *Journal of Business Ethics, 121*(2), 171-187. doi:10.1007/s10551-013-1692-4

Zachry, C. E., Phan, L. V., Blackie, L. E. R., & Jayawickreme, E. (2018). Situation-Based Contingencies Underlying Wisdom-Content Manifestations: Examining Intellectual Humility in Daily Life. *Journals of Gerontology - Series B Psychological Sciences and Social Sciences, 73*(8), 1404-1415. doi:10.1093/geronb/gby016

Zágon, I. K., & Jackson, H. J. (1994). Construct validity of a psychopathy measure. *Personality and Individual Differences, 17*(1), 125-135. doi:10.1016/0191-8869(94)90269-0

Zajenkowski, M., Czarna, A. Z., Szymaniak, K., & Dufner, M. (2020). What do highly narcissistic people think and feel about (their) intelligence? *Journal of Personality, 88*(4), 703-718. doi:10.1111/jopy.12520

Zajenkowski, M., & Fronczyk, K. (2020). How do narcissists perceive personality items? Measurement invariance of a Big Five scale across low and high narcissism groups. *Personality and Individual Differences, 152*. doi:10.1016/j.paid.2019.109595

Zajenkowski, M., & Gignac, G. E. (2018). Why do angry people overestimate their intelligence? Neuroticism as a suppressor of the association between Trait-Anger and subjectively assessed intelligence. *Intelligence, 70*, 12-21. doi:10.1016/j.intell.2018.07.003

Zajenkowski, M., & Gignac, G. E. (2021a). Narcissism and intelligence among couples: Why are narcissistic women perceived as intelligent by their romantic partners? *Personality and Individual Differences, 172*. doi:10.1016/j.paid.2020.110579

Zajenkowski, M., & Gignac, G. E. (2021b). Telling people they are intelligent correlates with the feeling of narcissistic uniqueness: The influence of IQ feedback on temporary state narcissism. *Intelligence*, *89*, 8. <https://doi.org/10.1016/j.intell.2021.101595>

Zajenkowski, M., Jankowski, K. S., & Stolarski, M. (2019). Why do evening people consider themselves more intelligent than morning individuals? The role of big five, narcissism, and objective cognitive ability. *Chronobiology International, 36*(12), 1741-1751. doi:10.1080/07420528.2019.1680559

Zajenkowski, M., Maciantowicz, O., Szymaniak, K., & Urban, P. (2018). Vulnerable and grandiose narcissism are differentially associated with ability and trait emotional intelligence. *Frontiers in Psychology, 9*(AUG). doi:10.3389/fpsyg.2018.01606

Zajenkowski, M., & Szymaniak, K. (2019). Narcissism between facets and domains. The relationships between two types of narcissism and aspects of the Big Five. *Current Psychology*. doi:10.1007/s12144-019-0147-1

Zajenkowski, M., Witowska, J., Maciantowicz, O., & Malesza, M. (2016). Vulnerable past, grandiose present: The relationship between vulnerable and grandiose narcissism, time perspective and personality. *Personality and Individual Differences, 98*, 102-106. doi:10.1016/j.paid.2016.03.092

Zamora, J., Ungson, N. D., & Seidman, G. (2022). The end justifies the me: Self-interest moderates the relationship between Dark Triad traits and utilitarian moral decisions. *Personality and Individual Differences*, *184*, 7. <https://doi.org/10.1016/j.paid.2021.111134>

Zara, A., & Özdemir, B. (2018). God complex: The effects of narcissistic personality on relational and sexual behavior. *Anadolu Psikiyatri Dergisi, 19*(1), 29-36. doi:10.5455/apd.263103

Zdunek, R. R., Czarna, A. Z., & Sedikides, C. (2022). Grandiose (communal and agentic) narcissism and predicted (dis) obedience in the Milgram paradigm. *Personality and Individual Differences*, *189*, 6. <https://doi.org/10.1016/j.paid.2022.111514>

Zeigler-Hill, V. (2006). Discrepancies between implicit and explicit self-esteem: Implications for narcissism and self-esteem instability. *Journal of Personality, 74*(1), 119-144. doi:10.1111/j.1467-6494.2005.00371.x

Zeigler-Hill, V., Besser, A., Morag, J., & Keith Campbell, W. (2016). The Dark Triad and sexual harassment proclivity. *Personality and Individual Differences, 89*, 47-54. doi:10.1016/j.paid.2015.09.048

Zeigler-Hill, V., Chadha, S., & Osterman, L. (2008). Psychological defense and self-esteem instability: Is defense style associated with unstable self-esteem? *Journal of Research in Personality, 42*(2), 348-364. doi:10.1016/j.jrp.2007.06.002

Zeigler-Hill, V., Clark, C. B., & Pickard, J. D. (2008). Narcissistic subtypes and contingent self-esteem: Do all narcissists base their self-esteem on the same domains? *Journal of Personality, 76*(4), 753-774. doi:10.1111/j.1467-6494.2008.00503.x

Zeigler-Hill, V., Myers, E. M., & Clark, C. B. (2010). Narcissism and self-esteem reactivity: The role of negative achievement events. *Journal of Research in Personality, 44*(2), 285-292. doi:10.1016/j.jrp.2010.02.005

Żemojtel-Piotrowska, M., Piotrowski, J., Rogoza, R., Baran, T., Hitokoto, H., & Maltby, J. (2019). Cross-cultural invariance of NPI-13: Entitlement as culturally specific, leadership and grandiosity as culturally universal. *International Journal of Psychology, 54*(4), 439-447. doi:10.1002/ijop.12487

Żemojtel-Piotrowska, M., Piotrowski, J., Sedikides, C., Sawicki, A., Czarna, A. Z., Fatfouta, R., & Baran, T. (2021). Communal collective narcissism. *Journal of Personality*. doi:10.1111/jopy.12636

Żemojtel-Piotrowska, M. A., Piotrowski, J., Pers, P., Tomiałowicz, E., & Clinton, A. (2018). Narcissism and its relationship with counterproductive work behavior: Mediational effects of psychological entitlement and subjective well-being. *Polish Psychological Bulletin, 49*(4), 442-448. doi:10.24425/119513

Zhang, H., Ou, A. Y., Tsui, A. S., & Wang, H. (2017). CEO humility, narcissism and firm innovation: A paradox perspective on CEO traits. *Leadership Quarterly, 28*(5), 585-604. doi:10.1016/j.leaqua.2017.01.003

Zhang, H., Wang, Z., You, X., Lü, W., & Luo, Y. (2015). Associations between narcissism and emotion regulation difficulties: Respiratory sinus arrhythmia reactivity as a moderator. *Biological Psychology, 110*, 1-11. doi:10.1016/j.biopsycho.2015.06.014

Zhang, L., & Baumeister, R. F. (2006). Your money or your self-esteem: Threatened egotism promotes costly entrapment in losing endeavors. *Personality and Social Psychology Bulletin, 32*(7), 881-893. doi:10.1177/0146167206287120

Zhang, S., Roberts, R., Woodman, T., & Cooke, A. (2020). I am great, but only when I also want to dominate: Maladaptive narcissism moderates the relationship between adaptive narcissism and performance under pressure. *Journal of Sport and Exercise Psychology, 42*(4), 323-335. doi:10.1123/JSEP.2019-0204

Zhang, G., Wang, H., & Li, M. (2023). Leader narcissism, perceived leader narcissism, and employee outcomes: The moderating effect of goal congruence. *Journal of Business Research*, *166*. <https://doi.org/10.1016/j.jbusres.2023.114115>

Zhang, K., & Cui, Z. (2022a). Are narcissists always bad apples? The relationship between employee narcissism and creative deviance. *Frontiers in Psychology*, *13*. <https://doi.org/10.3389/fpsyg.2022.1026649>

Zhang, S., Kim, Y. K., Fingerman, K. L., Birditt, K. S., & Charles, S. T. (2022b). Narcissism, Social Experiences, and Mood in Late Life [Article]. *Journals of Gerontology - Series B Psychological Sciences and Social Sciences*, *77*(8), 1442-1453. <https://doi.org/10.1093/geronb/gbac019>

Zhang, S., & Boardley, I. (2023). The ‘selves’ in banned performance enhancement: Investigating narcissism and compassion in the context of doping. *Performance Enhancement and Health*, *11*(1). <https://doi.org/10.1016/j.peh.2022.100243>

Zhang, S., Roberts, R., Akehurst, S., & Woodman, T. (2024). Narcissism and antisocial behaviour in sport: The moderating role of self-compassion. *Psychology of Sport and Exercise*, *70*. <https://doi.org/10.1016/j.psychsport.2023.102528>

Zhang, S. G., Roberts, R., Woodman, T., Pitkethly, A., English, C., & Nightingale, D. (2021). Foresee the Glory and Train Better: Narcissism, Goal-Setting, and Athlete Training. *Sport Exercise and Performance Psychology*, *10*(3), 381-393. <https://doi.org/10.1037/spy0000264>

Zheng, P., & Leung, L. (2016). Linking psychological attributes, gratifications and social networking site use to social capital of the net generation in China. *International Journal of Cyber Behavior, Psychology and Learning, 6*(3), 17-33. doi:10.4018/IJCBPL.2016070102

Zhao, Y. X., Zhou, K., & Liu, W. X. (2023). Why and when narcissistic employees are more creative in the workplace? A social cognitive perspective. *Management and Organization Review*, *19*(3), 567-593, Article Pii s1740877622000420. <https://doi.org/10.1017/mor.2022.42>

Zhu, X., & Geng, Y. (2021). HERO or DT, what comes first? The causal relationship between psychological capital and dark tetrad differed. *Current Psychology*. doi:10.1007/s12144-021-01544-7

Zitek, E. M., & Jordan, A. H. (2016). Narcissism Predicts Support for Hierarchy (At Least When Narcissists Think They Can Rise to the Top). *Social Psychological and Personality Science, 7*(7), 707-716. doi:10.1177/1948550616649241

Zuckerman, M., & O'Loughlin, R. E. (2006). Self-enhancement by social comparison: A prospective analysis. *Personality and Social Psychology Bulletin, 32*(6), 751-760. doi:10.1177/0146167205286111

Zvi, L., & Elaad, E. (2018). Correlates of narcissism, self-reported lies, and self-assessed abilities to tell and detect lies, tell truths, and believe others. *Journal of Investigative Psychology and Offender Profiling, 15*(3), 271-286. doi:10.1002/jip.1511

**The following 22 unpublished articles which were included in the studies by Twenge et al. (2021) and Hamamura et al. (2020) were included without being reviewed by the author of this study:**

Adler (2000)

Bartels (2005)

Campbell, Finkel, Buffardi, Kumashiro & Rusbult (2007) Study 1

DeWall (2004

Gaertner, Iuzzini, & O’Mara (2006)

Konrath & Bushman (2006)

Krusemark (2005)

Krusemark (2006)

Liu (2005)

Mead (2006)

DeYoung (2009)

Faulkner, K. (2012)

Konrath 2009a unpublished data

Konrath 2009b unpublished data

Price (2010)

White (2009)

Zarins, & Konrath (2014) Study 2

Hamamura, T. (2019). Self-Regards among Australian Students

Lagudi, A. L. (2017). *The relationship between Baumrind’s (1971) Parenting Styles and Narcissism: A community study.* Jansen Newman Institute.

Friesdorf, R. (2017). NPI-40 data.

Lange, J. (2016). NPI data.

Craig Nathanson. (2008). Exploring the dynamics of revenge, (November), 146. http://doi.org/10.1017/CBO9781107415324.004
