# Supplementary material for: A Farewell to the Narcissism Epidemic? A Cross‐Temporal Meta‐Analysis of Global NPI Scores (1982–2023)
Source: J Pers. 2024 Oct 14;93(4):884–94. doi: 10.1111/jopy.12982 (PMC12224556; doi:10.1111/jopy.12982)

**Global - all samples - NPI40**


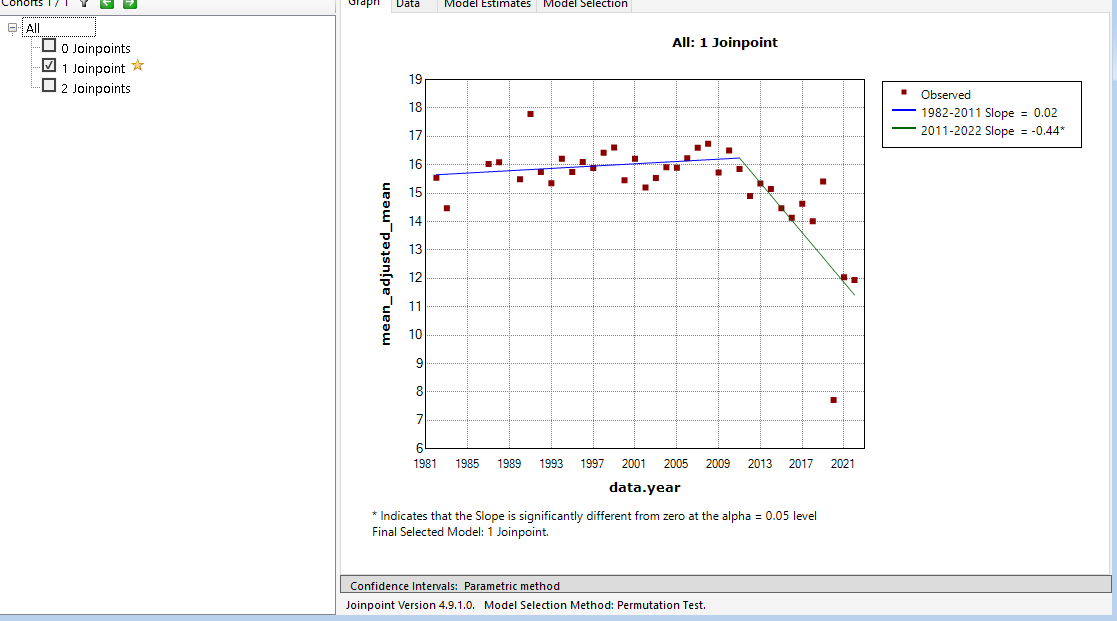


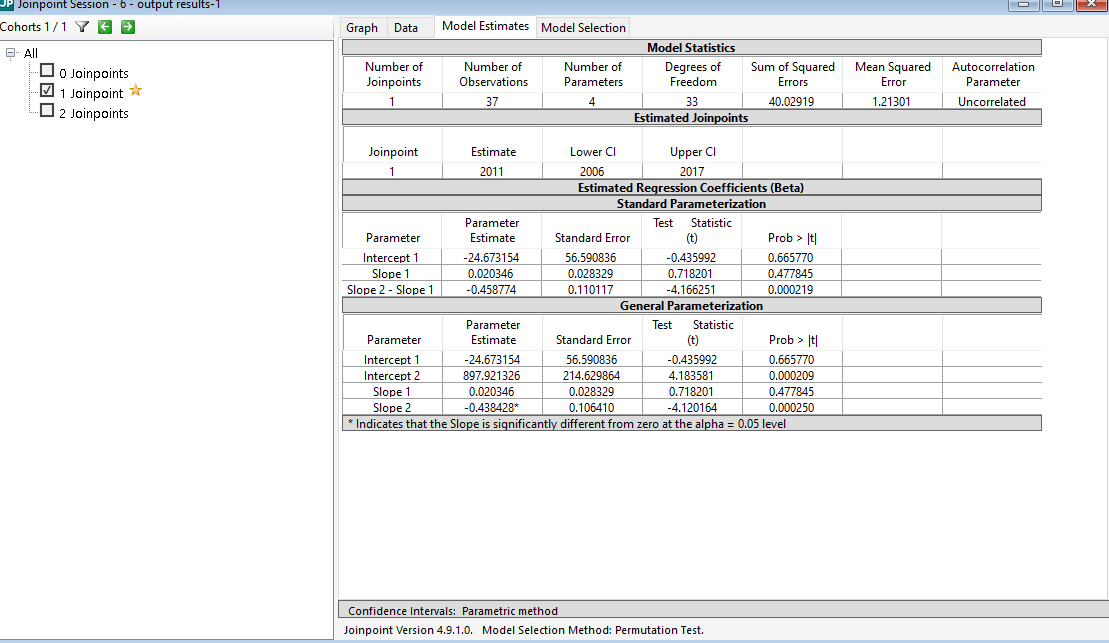


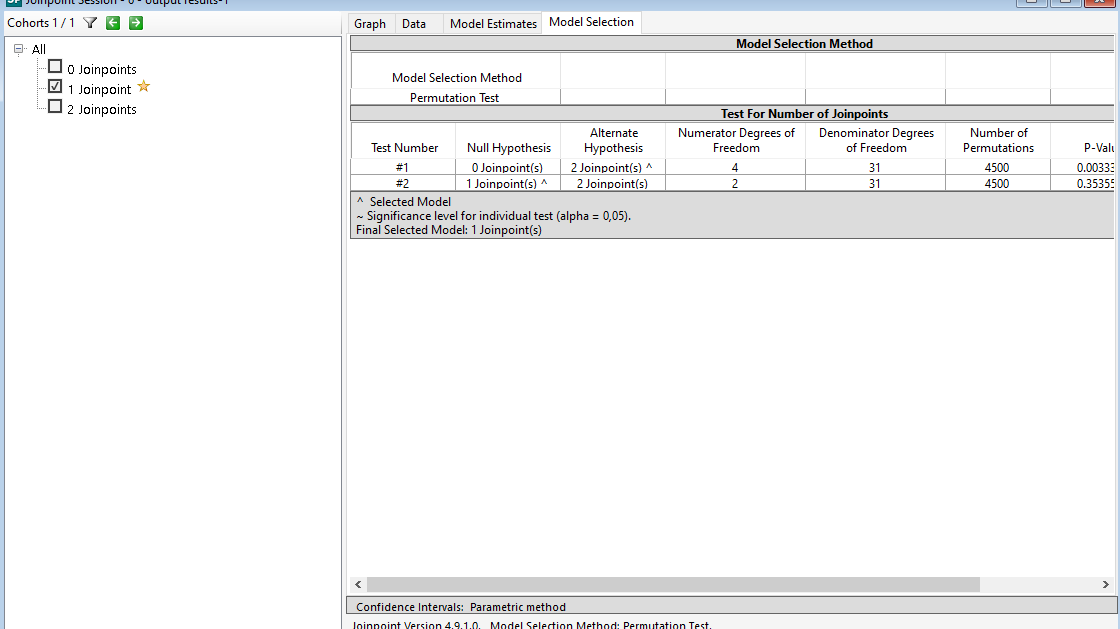


**Global – all samples – any NPI**


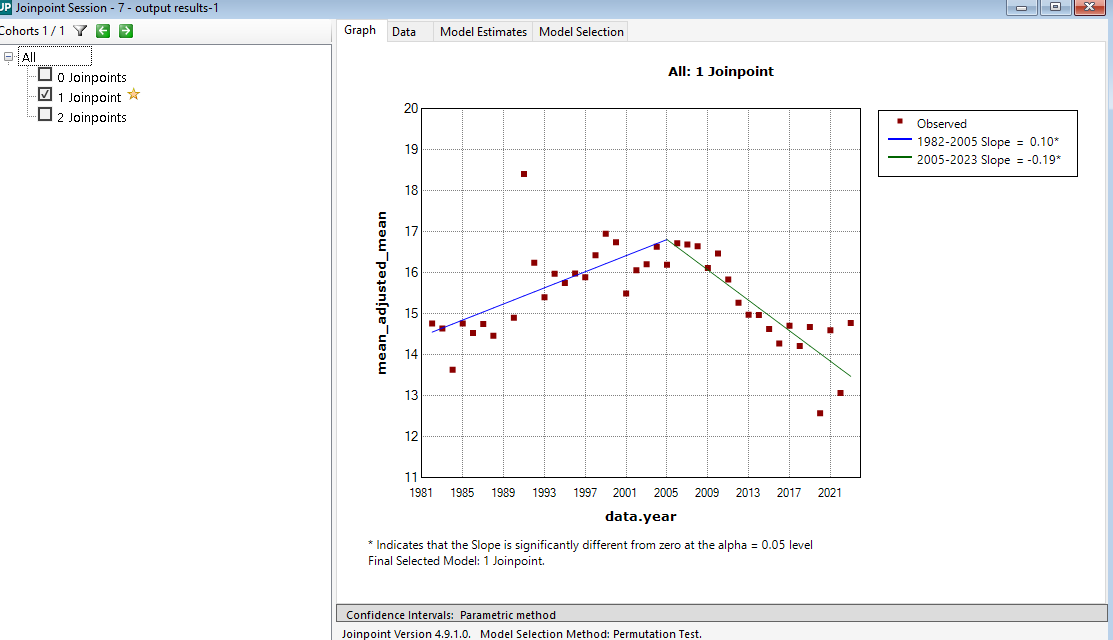


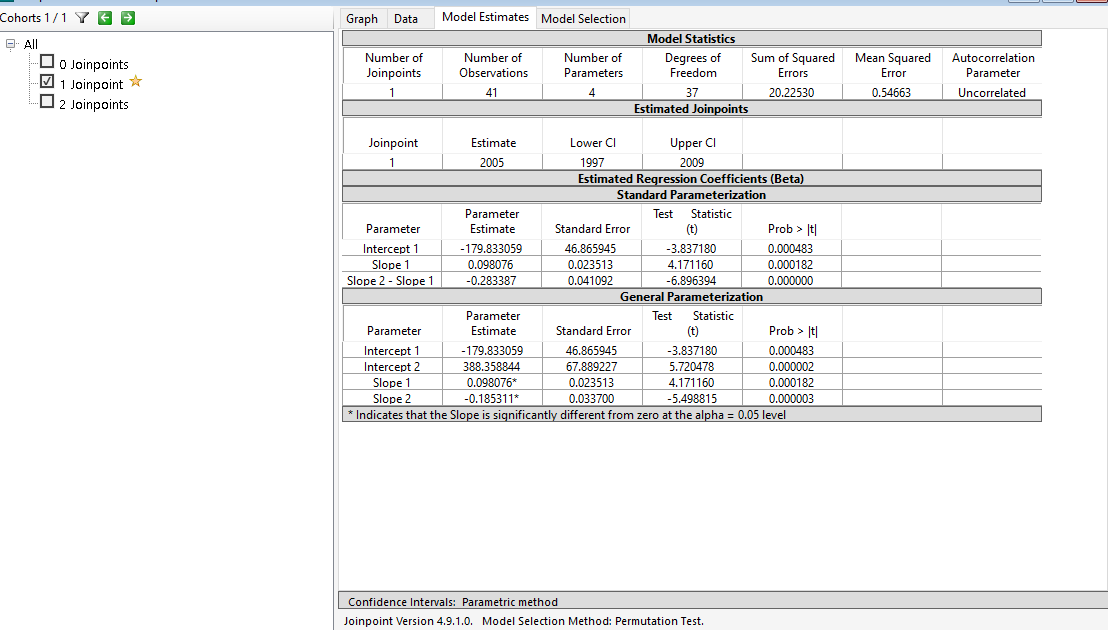


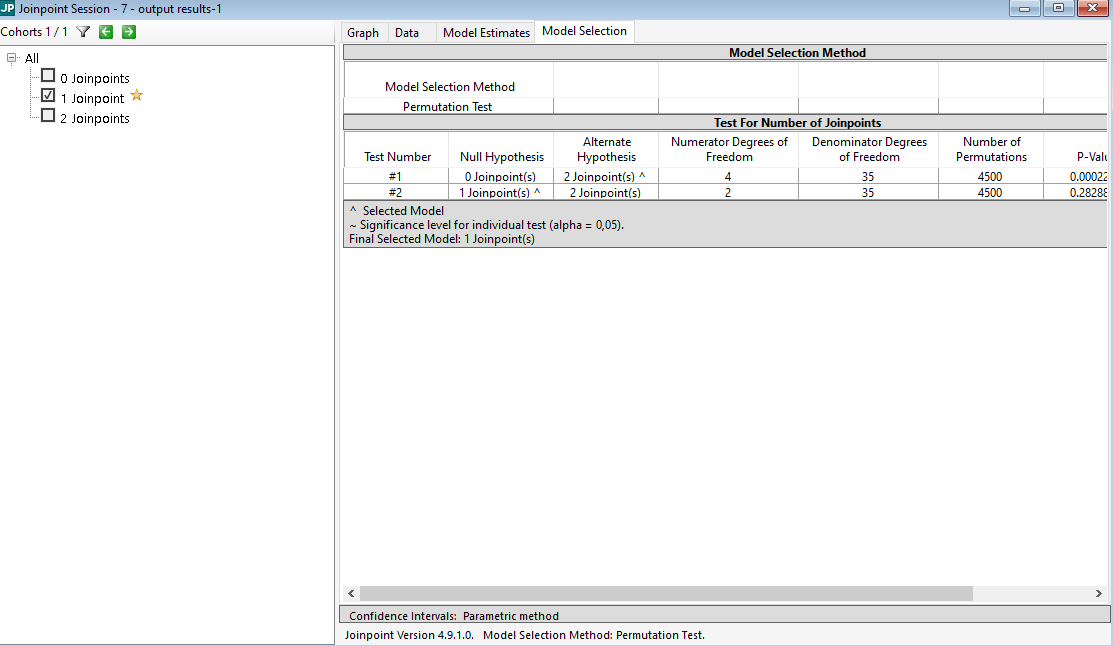


**Global - students - NPI40**


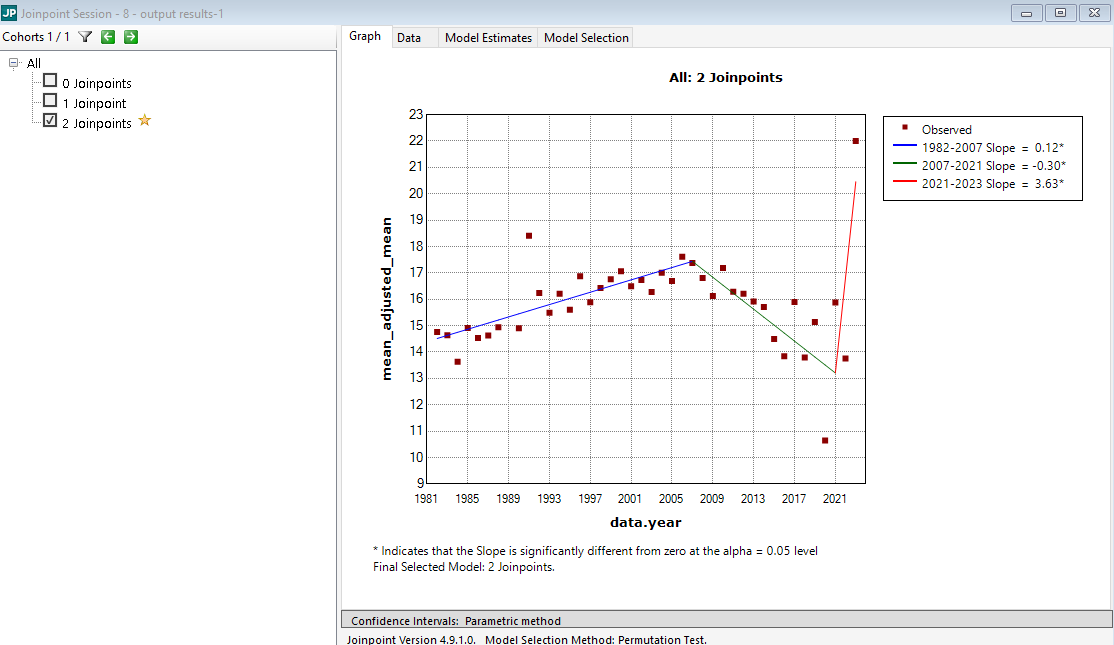

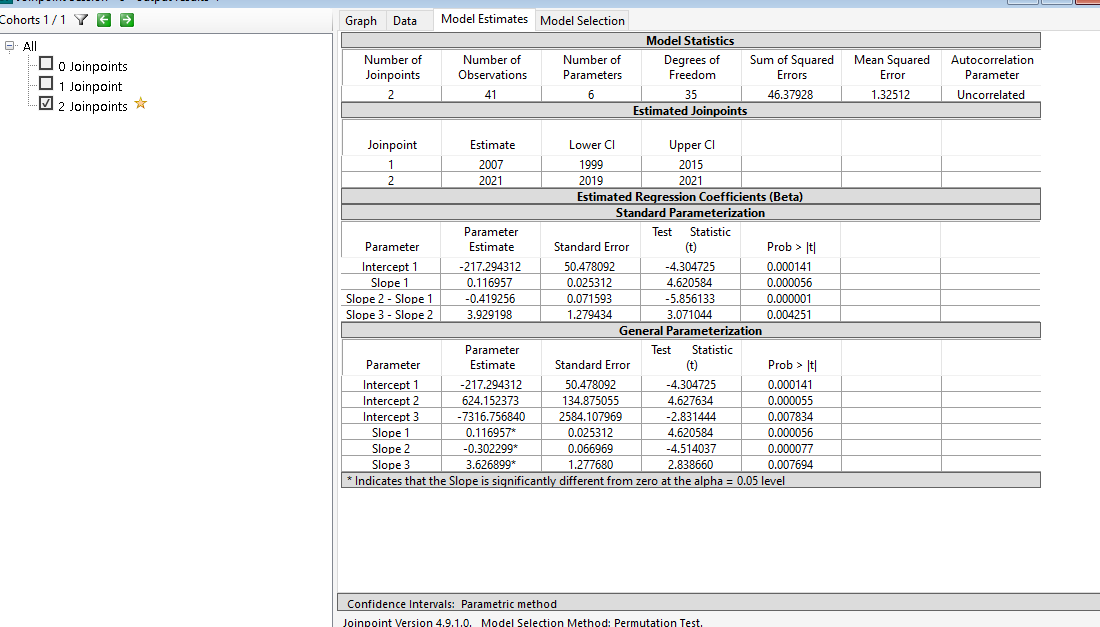


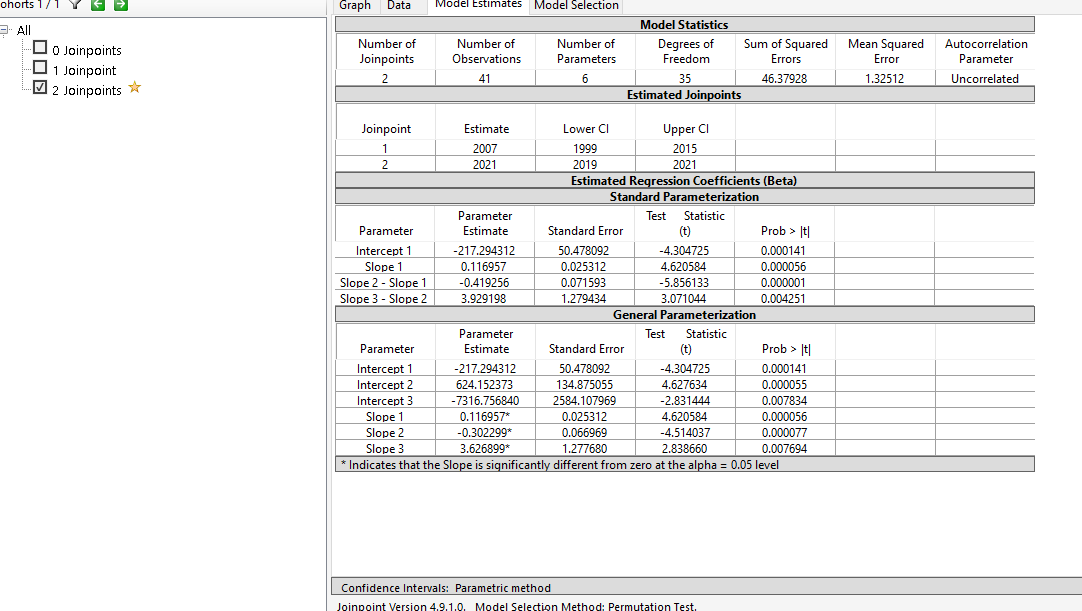


**Global - students – any NPI**


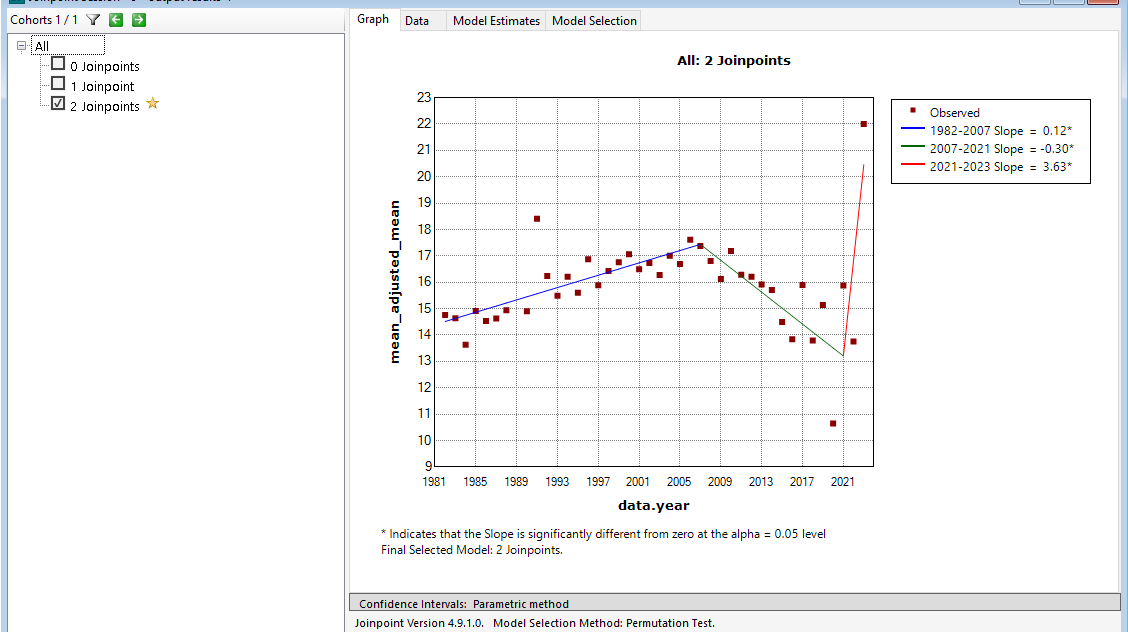


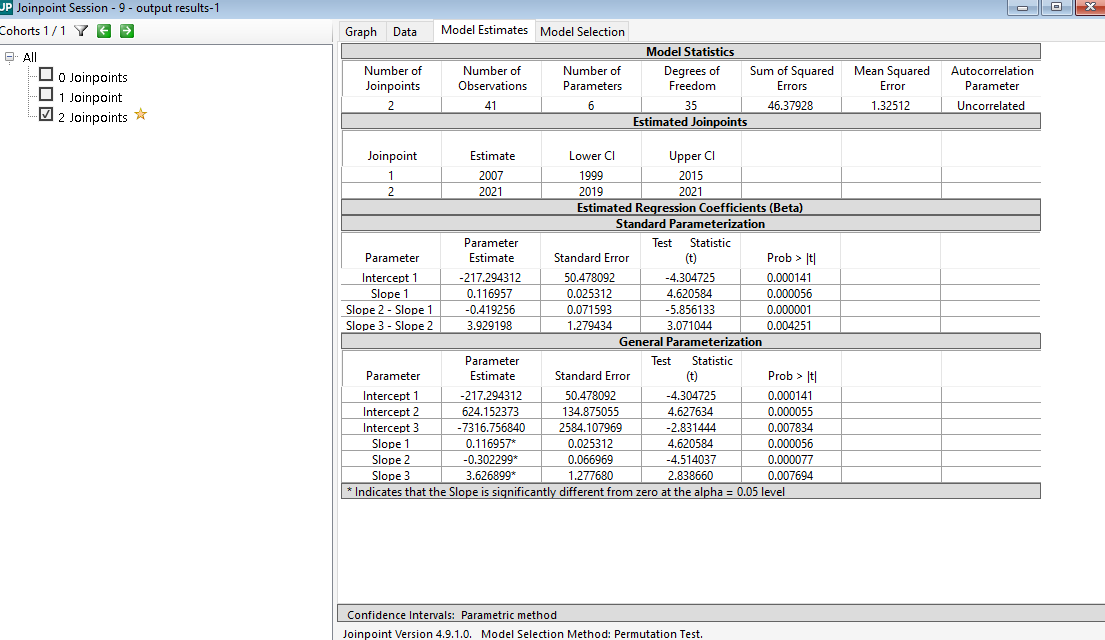


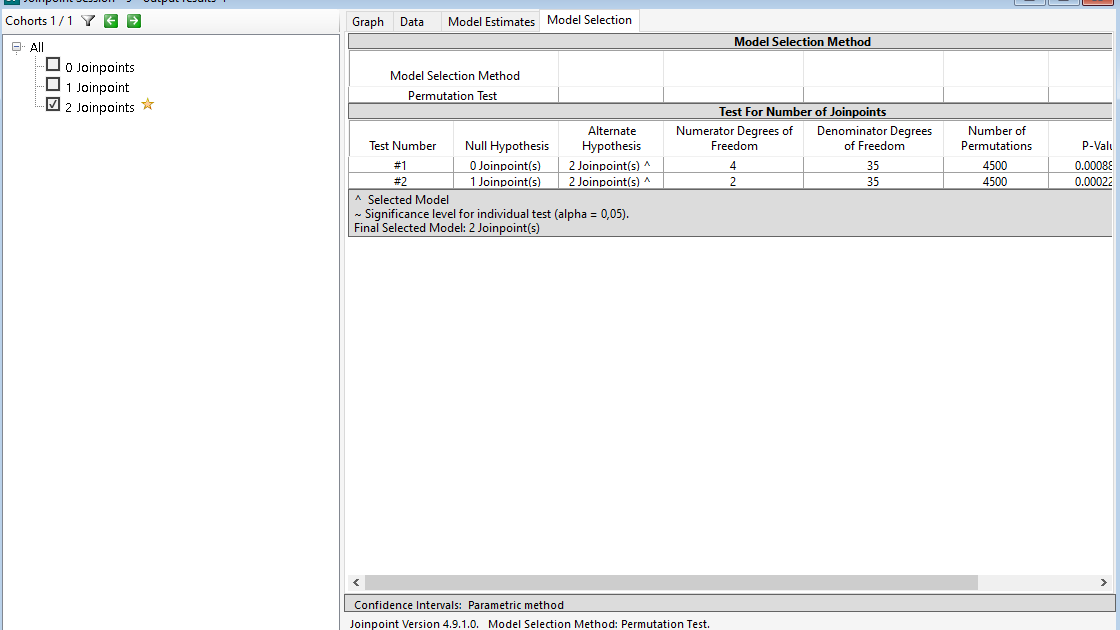


**USA - all samples - NPI40**


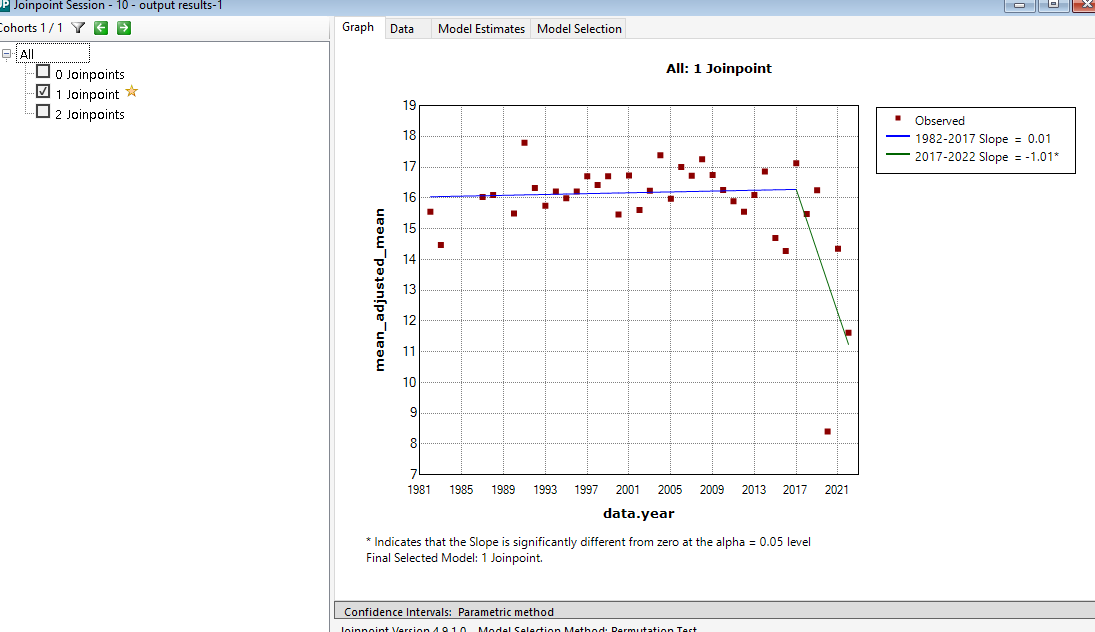


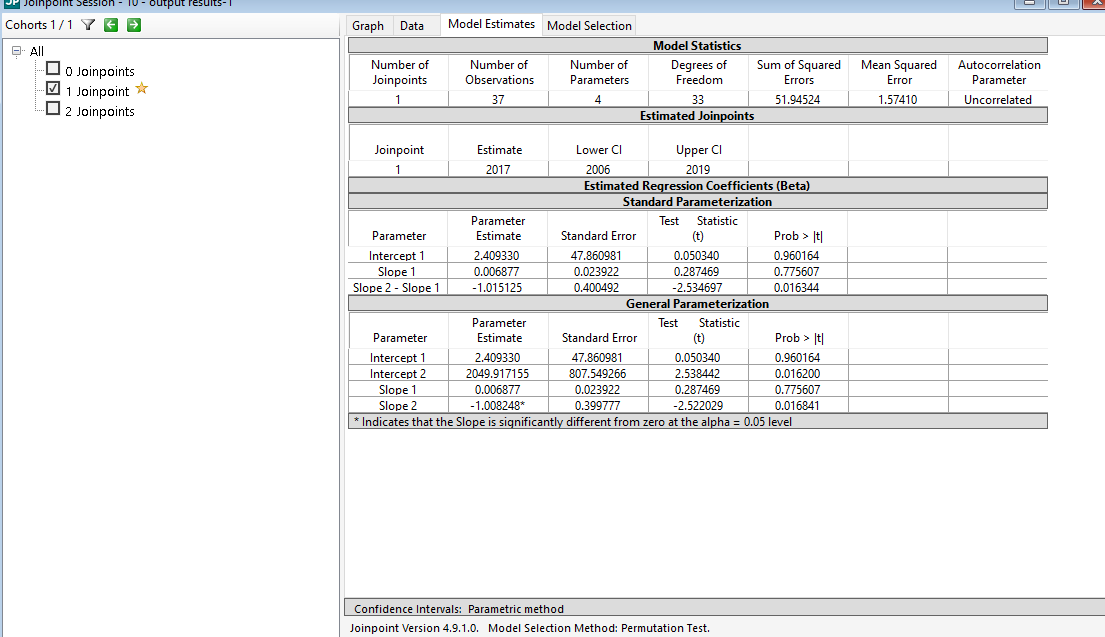


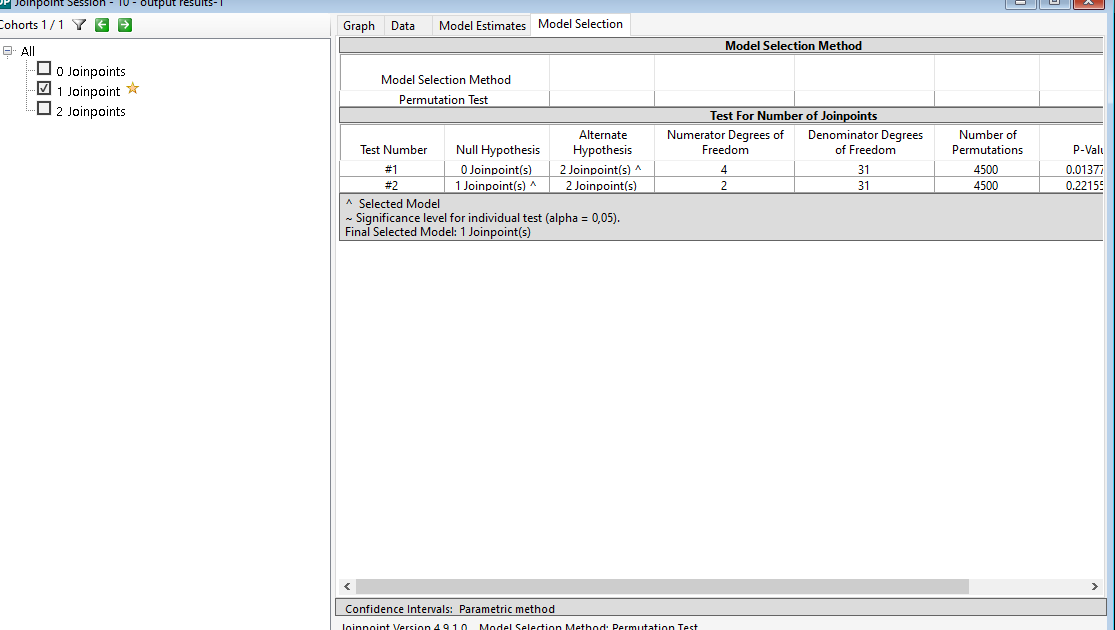


**USA - all samples – any NPI**


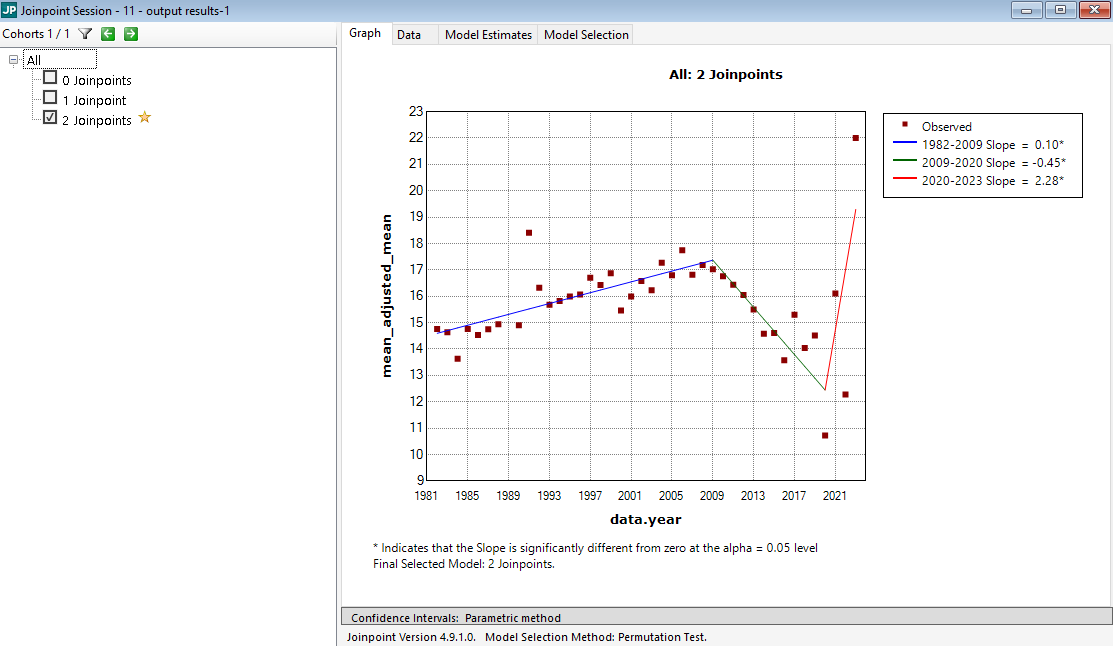


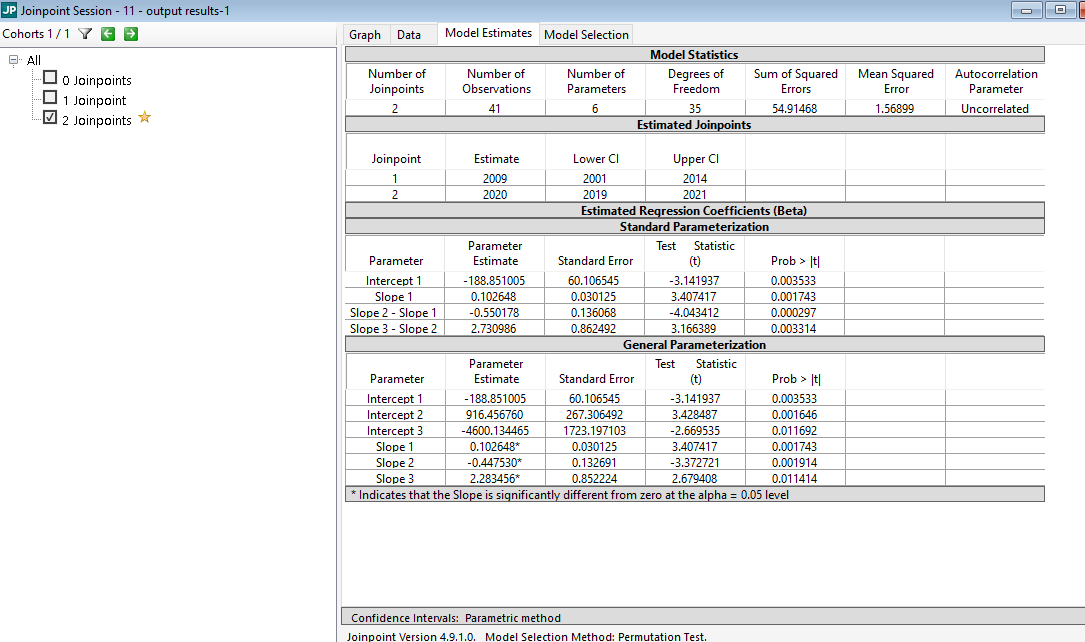


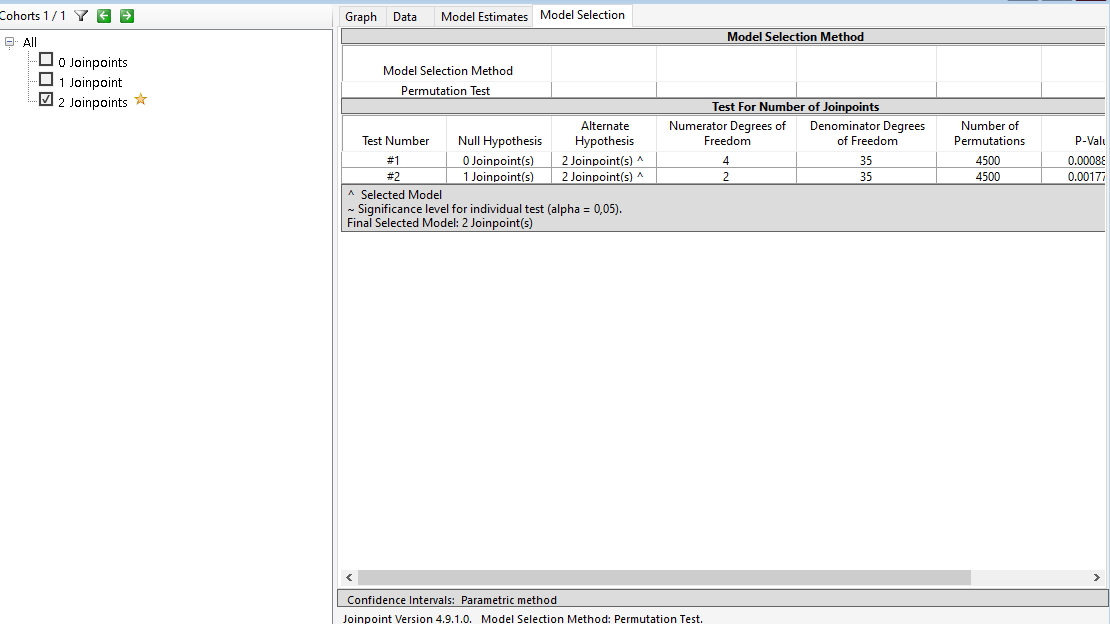


**USA - students - NPI40**


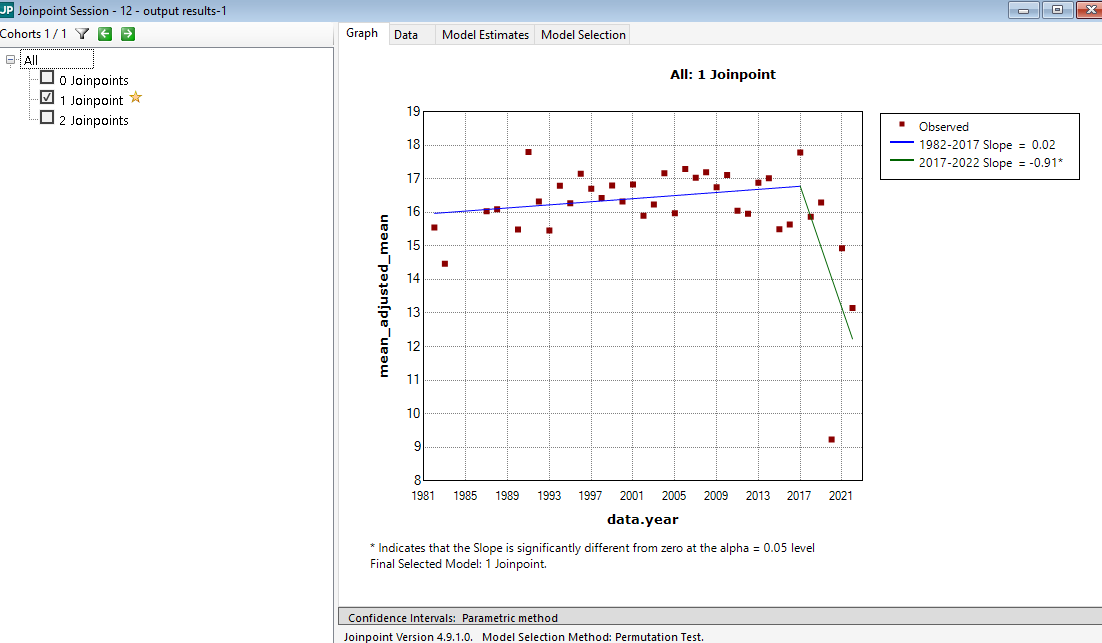


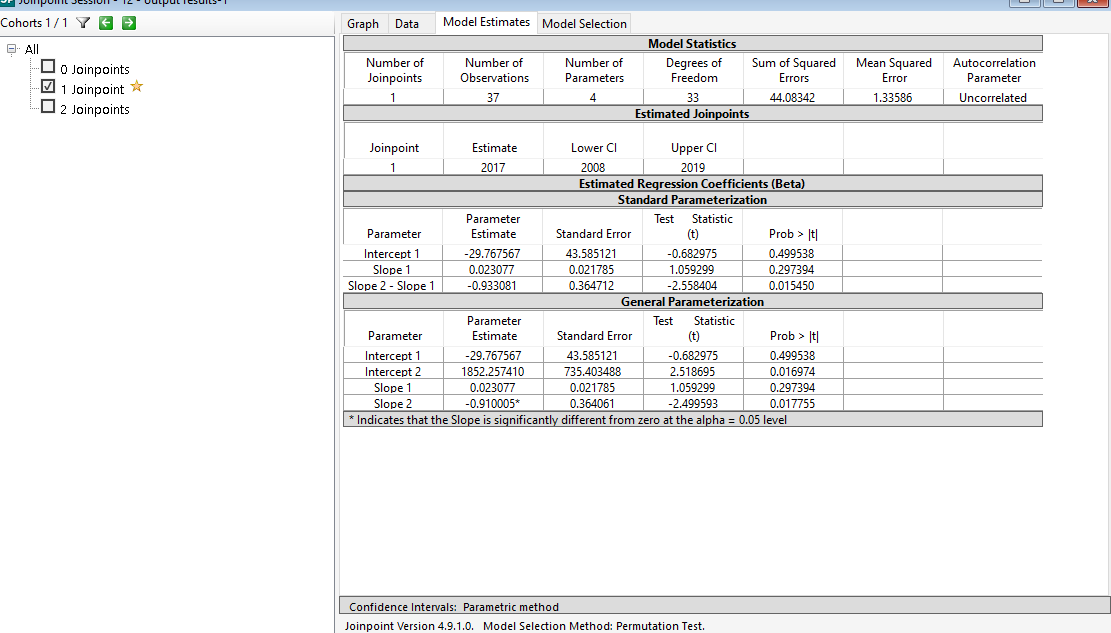


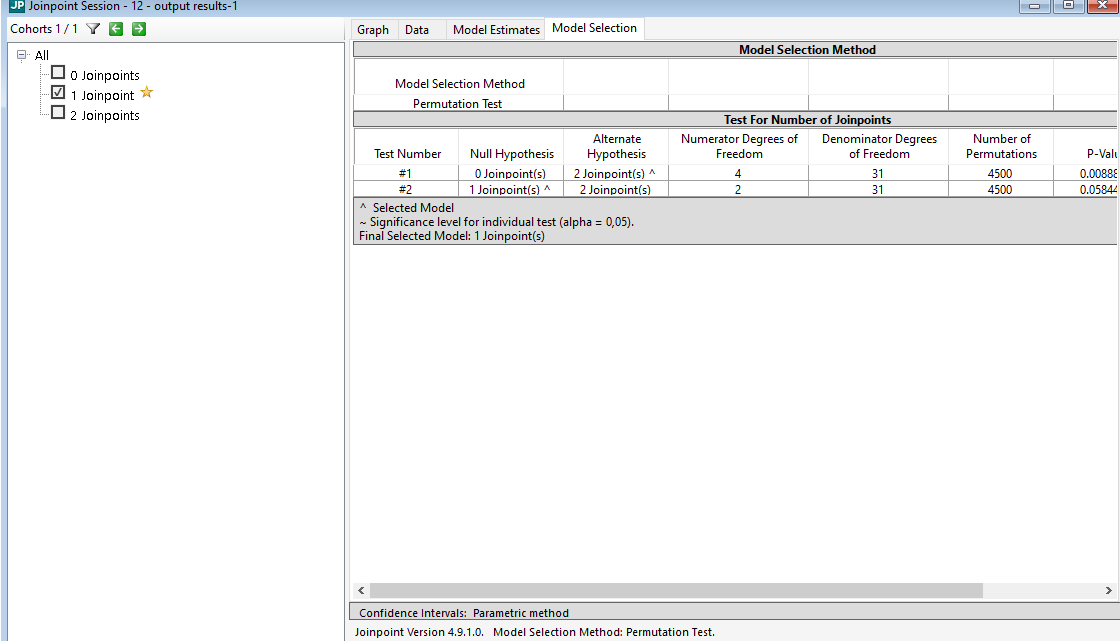


**USA - students - any NPI**


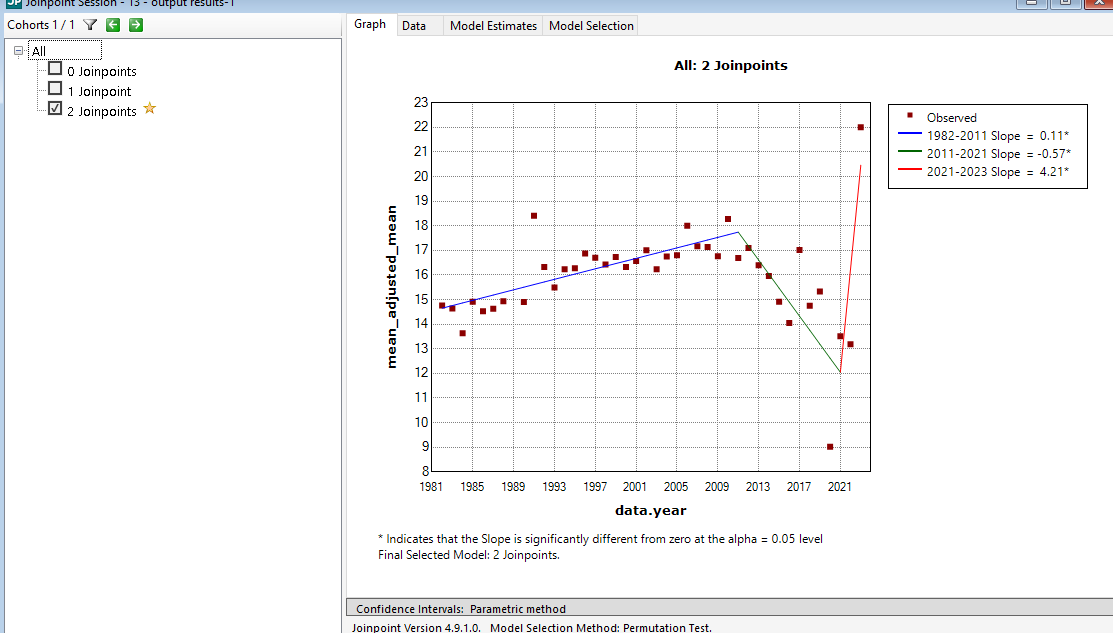


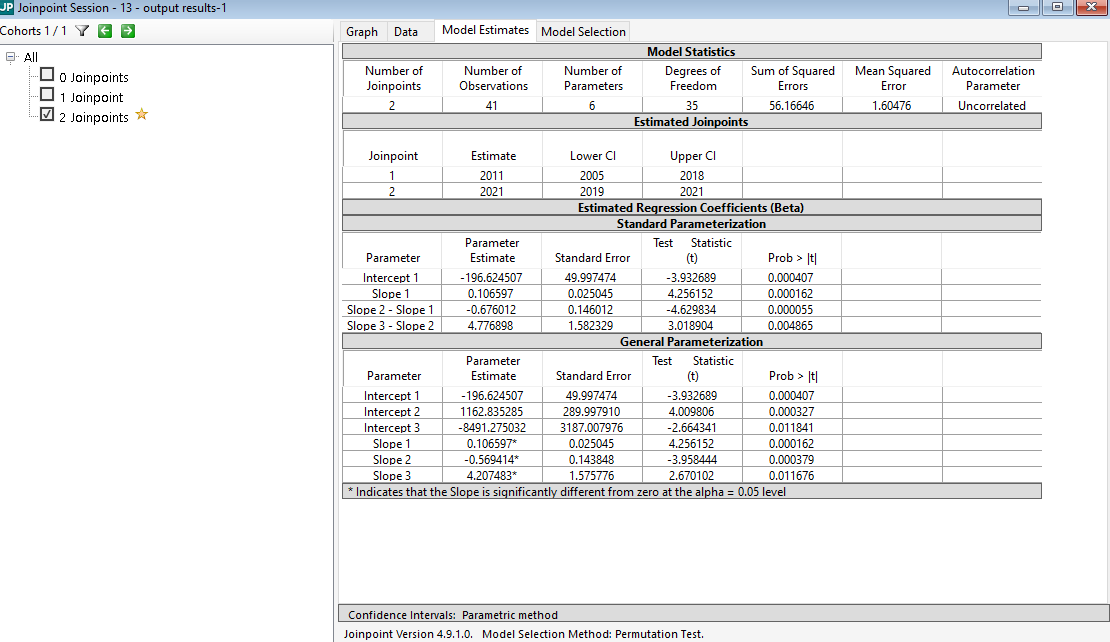


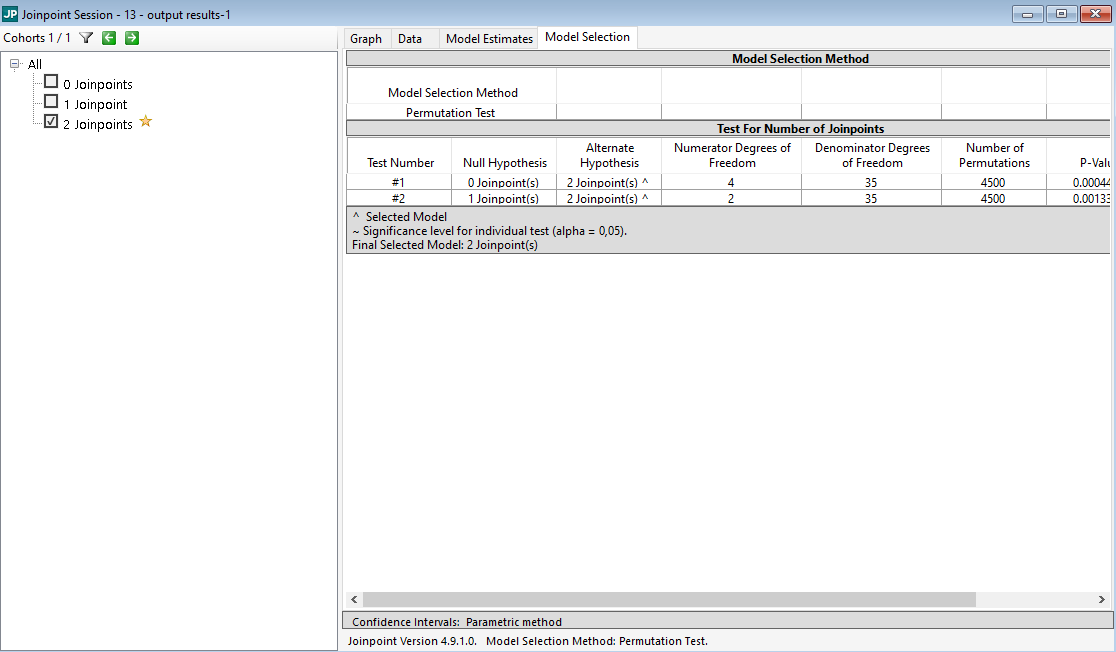

Supplement: Supplementary file 1 — Data S1. [file JOPY-93-884-s001.zip › Joinpoints analyses/Joinpoints output.docx]
