# Supplementary material for: A Farewell to the Narcissism Epidemic? A Cross‐Temporal Meta‐Analysis of Global NPI Scores (1982–2023)
Source: J Pers. 2024 Oct 14;93(4):884–94. doi: 10.1111/jopy.12982 (PMC12224556; doi:10.1111/jopy.12982)
Supplement: Supplementary file 1 — Data S1. [file JOPY-93-884-s001.zip › Tables_S1_to_S43 (2) (1).docx]

| **Table S1**  *Single and multiple linear weighted meta-regression as well as residualized regressions for U.S.-based samples on any NPI scores from 1982 to 2023.* | | | | | | | | |
| --- | --- | --- | --- | --- | --- | --- | --- | --- |
| Predictors | Model fit | *k* | *b* | *SE* | β | $\eta_{p}^{2}$ | *p* | |
| Single regression | | | | | | | | |
|  | *R²* = .055***; *F* (1, 746) = 44.20*** | 747 |  |  |  |  |  | |
| Year of data collection (1982-2023) |  |  | -0.108 | 0.016 | -.253 | .056 | <.001 | |
| Multiple regression | | | | | | | | |
|  | *R²* = .096***; *F* (3, 547) = 20.69*** | 550 |  |  |  |  |  | |
| Year of data collection (1982-2023) |  |  | -0.062 | 0.020 | -.135 | .031 | .002 | |
| Sample mean age |  |  | 0.114 | 0.183 | -.028 | .005 | <.001 | |
| Percentage of women in sample |  |  | -0.026 | 0.007 | -.305 | .029 | <.001 | |
| Regression on residualized values | | | | | | | | |
|  | *R²* = .014**; *F* (1, 549) = 9.93** | 550 |  |  |  |  |  | |
| Year of data collection^a^ (1982-2023) |  |  | -0.061 | 0.020 | -.141 | .016 | .002 | |
| *Note.* Variables were weighted based on sample size; all *R²* are adjusted values; *k* = number of samples; *b* = unstandardized regression coefficient; *SE* = standard error of unstandardized coefficient; β = standardized regression coefficient; $\eta_{p}^{2}$ = partial eta squared; Variance Inflation Factors (VIFs) in multiple Regression were all <1.1.  ^a^ Adjusted for confounding variables (Percentage of women in sample & sample mean age).  ***p*<.01; ****p* <.001. | | | | | | | |  |

| **Table S2**  *Single and multiple linear weighted meta-regression as well as residualized regressions for U.S.-based samples on forced-choice-based NPI-40 scores from 1982 to 2023.* | | | | | | | |
| --- | --- | --- | --- | --- | --- | --- | --- |
| Predictors | Model fit | *k* | *b* | *SE* | β | $\eta_{p}^{2}$ | *p* |
| Single regression | | | | | | | |
|  | *R²* = .007***; *F* (1, 467) = 34.20*** | 468 |  |  |  |  |  |
| Year of data collection (1982-2023) |  |  | -0.100 | 0.017 | -.268 | .068 | <.001 |
| Multiple regression | | | | | | | |
|  | *R²* = .195***; *F* (3, 330) = 27.91*** | 333 |  |  |  |  |  |
| Year of data collection (1982-2023) |  |  | -0.082 | 0.021 | -.192 | .069 | <.001 |
| Sample mean age |  |  | 0.141 | 0.021 | -.331 | .106 | <.001 |
| Percentage of women in sample |  |  | -0.031 | 0.006 | -.293 | .057 | <.001 |
| Regression on residualized values | | | | | | | |
|  | *R²* = .034***; *F* (1, 332) = 14.47*** | 333 |  |  |  |  |  |
| Year of data collection^a^ (1982-2023) |  |  | -0.081 | 0.021 | -.205 | .042 | <.001 |
| *Note.* Variables were weighted based on sample size; all *R²* are adjusted values; *k* = number of samples; *b* = unstandardized regression coefficient; *SE* = standard error of unstandardized coefficient; β = standardized regression coefficient; $\eta_{p}^{2}$ = partial eta squared; Variance Inflation Factors (VIFs) in multiple Regression were all <1.1.  ^a^ Adjusted for confounding variables (Percentage of women in sample & sample mean age).  ****p* <.001. | | | | | | | |

| **Table S3**  *Single and multiple linear weighted meta-regression as well as residualized regressions for U.S.-based student samples on any NPI scores from 1982 to 2023.* | | | | | | | |
| --- | --- | --- | --- | --- | --- | --- | --- |
| Predictors | Model fit | *k* | *b* | *SE* | β | $\eta_{p}^{2}$ | *p* |
| Single regression | | | | | | | |
|  | *R²* = .031***; *F* (1, 539) = 18.53*** | 540 |  |  |  |  |  |
| Year of data collection (1982-2023) |  |  | -0.071 | 0.017 | -.191 | .033 | <.001 |
| Multiple regression | | | | | | | |
|  | *R²* = .060***; *F* (3, 371) = 9.03*** | 374 |  |  |  |  |  |
| Year of data collection (1982-2023) |  |  | -0.057 | 0.02 | -.140 | .027 | .010 |
| Sample mean age |  |  | 0.270 | 0.111 | -.170 | .018 | .016 |
| Percentage of women in sample |  |  | -0.024 | 0.008 | -.210 | .026 | .002 |
| Regression on residualized values | | | | | | | |
|  | *R²* = .025*; *F* (1, 373) = 6.58* | 374 |  |  |  |  |  |
| Year of data collection^a^ (1982-2023) |  |  | -0.056 | 0.022 | -.139 | .017 | .011 |
| *Note.* Variables were weighted based on sample size; all *R²* are adjusted values; *k* = number of samples; *b* = unstandardized regression coefficient; *SE* = standard error of unstandardized coefficient; β = standardized regression coefficient; $\eta_{p}^{2}$ = partial eta squared; Variance Inflation Factors (VIFs) in multiple Regression were all <1.1.  ^a^ Adjusted for confounding variables (Percentage of women in sample & sample mean age).  **p*<.05; ***p*<.01; ****p* <.001. | | | | | | | |

| **Table S4**  *Single and multiple linear weighted meta-regression as well as residualized regressions for global samples on any NPI scores from 1982 to 2023.* | | | | | | | |
| --- | --- | --- | --- | --- | --- | --- | --- |
| Predictors | Model fit | *k* | *b* | *SE* | β | $\eta_{p}^{2}$ | *p* |
| Single regression | | | | | | | |
|  | *R*² = .050***; *F*(1, 1619) = 86.73*** | 1620 |  |  |  |  |  |
| Year of data collection (1982-2023) |  |  | -0.145 | 0.016 | -.267 | .050 | <.001 |
| Multiple regression | | | | | | | |
|  | *R²* = .080***; *F* (3, 1326) = 39.80*** | 1329 |  |  |  |  |  |
| Year of data collection (1982-2023) |  |  | -0.153 | 0.019 | -.252 | .055 | <.001 |
| Sample mean age |  |  | -0.06 | 0.013 | -.115 | .008 | <.001 |
| Percentage of women in sample |  |  | -0.023 | 0.004 | -.166 | .022 | <.001 |
| Regression on residualized values | | | | | | | |
|  | *R²* = .042***; *F* (1, 1328) = 60.08*** | 1329 |  |  |  |  |  |
| Year of data collection^a^ (1982-2023) |  |  | -0.148 | 0.019 | -.253 | .004 | <.001 |
| *Note.* Variables were weighted based on sample size; all *R²* are adjusted values; *k* = number of samples; *b* = unstandardized regression coefficient; *SE* = standard error of unstandardized coefficient; β = standardized regression coefficient; $\eta_{p}^{2}$ = partial eta squared; Variance Inflation Factors (VIFs) in multiple Regression were all <1.1.  ^a^ Adjusted for confounding variables (Percentage of women in sample & sample mean age, Scale, Items & Sample).  ****p* <.001. | | | | | | | |

| **Table S5**  *Single and multiple linear weighted meta-regression as well as residualized regressions for global samples on forced choice-based NPI-40 scores from 1982 to 2023.* | | | | | | | |
| --- | --- | --- | --- | --- | --- | --- | --- |
| Predictors | Model fit | *k* | *b* | *SE* | β | $\eta_{p}^{2}$ | *p* |
| Single regression | | | | | | | |
|  | *R²* = .116***; *F* (1, 812) = 107.8*** | 813 |  |  |  |  |  |
| Year of data collection (1982-2023) |  |  | -0.159 | 0.015 | -.379 | .117 | <.001 |
| Multiple regression | | | | | | | |
|  | *R²* = .177***; *F* (3, 640) = 47.05*** | 643 |  |  |  |  |  |
| Year of data collection (1982-2023) |  |  | -0.133 | 0.018 | -.282 | .099 | <.001 |
| Sample mean age |  |  | -0.113 | 0.015 | -.275 | .061 | <.001 |
| Percentage of women in sample |  |  | -0.026 | 0.005 | -.260 | .044 | <.001 |
| Regression on residualized values | | | | | | | |
|  | *R²* = .007***; *F* (1, 642) = 50.56*** | 643 |  |  |  |  |  |
| Year of data collection^a^ (1982-2023) |  |  | -0.131 | 0.185 | -.298 | .007 | <.001 |
| *Note.* Variables were weighted based on sample size; all *R²* are adjusted values; *k* = number of samples; *b* = unstandardized regression coefficient; *SE* = standard error of unstandardized coefficient; β = standardized regression coefficient; $\eta_{p}^{2}$ = partial eta squared; Variance Inflation Factors (VIFs) in multiple Regression were all <1.1.  ^a^ Adjusted for confounding variables (Percentage of women in sample & sample mean age).  ****p* <.001. | | | | | | | |

| **Table S6**  *Single and multiple linear weighted meta-regression as well as residualized regressions for global student samples on any NPI scores from 1982 to 2023.* | | | | | | | |
| --- | --- | --- | --- | --- | --- | --- | --- |
| Predictors | Model fit | *k* | *b* | *SE* | β | $\eta_{p}^{2}$ | *p* |
| Single regression | | | | | | | |
|  | *R²* = .040***; *F* (1, 866) = 37.47*** | 867 |  |  |  |  |  |
| Year of data collection (1982-2023) |  |  | -0.096 | 0.016 | -.218 | .041 | <.001 |
| Multiple regression | | | | | | | |
|  | *R²* = .060***; *F* (3, 674) = 15.47*** | 677 |  |  |  |  |  |
| Year of data collection (1982-2023) |  |  | -0.100 | 0.020 | -.186 | <.001 | <.001 |
| Sample mean age |  |  | -0.002 | 0.055 | -.001 | <.001 | .969 |
| Percentage of women in sample |  |  | -0.026 | 0.006 | -.211 | <.001 | <.001 |
| Regression on residualized values | | | | | | | |
|  | *R²* = .028***; *F* (1, 676) = 20.15*** | 677 |  |  |  |  |  |
| Year of data collection^a^ (1982-2023) |  |  | -0.090 | 0.020 | -.185 | .029 | <.001 |
| *Note.* Variables were weighted based on sample size; all *R²* are adjusted values; *k* = number of samples; *b* = unstandardized regression coefficient; *SE* = standard error of unstandardized coefficient; β = standardized regression coefficient; $\eta_{p}^{2}$ = partial eta squared; Variance Inflation Factors (VIFs) in multiple Regression were all <1.1.  ^a^ Adjusted for confounding variables (Percentage of women in sample & sample mean age).  ****p* <.001. | | | | | | | |

| **Table S7**  *Single and multiple linear weighted meta-regression as well as residualized regressions for global student samples on forced choice-based NPI-40 scores from 1982 to 2023.* | | | | | | | |
| --- | --- | --- | --- | --- | --- | --- | --- |
| Predictors | Model fit | *k* | *b* | *SE* | β | $\eta_{p}^{2}$ | *p* |
| Single regression | | | | | | | |
|  | *R²* = .038***; *F* (1, 520) = 21.35*** | 521 |  |  |  |  |  |
| Year of data collection (1982-2023) |  |  | -0.072 | 0.016 | -.195 | .039 | <.001 |
| Multiple regression | | | | | | | |
|  | *R²* = .104***; *F* (3, 387) = 16.14*** | 390 |  |  |  |  |  |
| Year of data collection (1982-2023) |  |  | -0.008 | 0.021 | -.180 | .050 | <.001 |
| Sample mean age |  |  | -0.122 | 0.071 | -.082 | .007 | .005 |
| Percentage of women in sample |  |  | -0.030 | 0.006 | -.310 | .061 | <.001 |
| Regression on residualized values | | | | | | | |
|  | *R²* = .032***; *F* (1, 389) = 14.06*** | 390 |  |  |  |  |  |
| Year of data collection^a^ (1982-2023) |  |  | -0.078 | 0.021 | -.183 | .035 | <.001 |
| *Note.* Variables were weighted based on sample size; all *R²* are adjusted values; *k* = number of samples; *b* = unstandardized regression coefficient; *SE* = standard error of unstandardized coefficient; β = standardized regression coefficient; $\eta_{p}^{2}$ = partial eta squared; Variance Inflation Factors (VIFs) in multiple Regression were all <1.1.  ^a^ Adjusted for confounding variables (Percentage of women in sample & sample mean age).  ****p* <.001. | | | | | | | |

| **Table S8**  *Single and multiple linear weighted meta-regression as well as residualized regressions for Asian samples on any NPI scores from 1982 to 2023.* | | | | | | | |
| --- | --- | --- | --- | --- | --- | --- | --- |
| Predictors | Model fit | *k* | *b* | *SE* | β | $\eta_{p}^{2}$ | *p* |
| Single regression | | | | | | | |
|  | *R²* = .086***; *F* (1, 174) = 17.46*** | 175 |  |  |  |  |  |
| Year of data collection (1982-2023) |  |  | -0.291 | 0.070 | -.342 | .091 | <.001 |
| Multiple regression | | | | | | | |
|  | *R²* = .120***; *F* (3, 136) = 7.263*** | 140 |  |  |  |  |  |
| Year of data collection (1982-2023) |  |  | -0.315 | 0.079 | -.392 | .114 | <.001 |
| Sample mean age |  |  | -0.011 | 0.065 | -.018 | <.001 | .871 |
| Percentage of women in sample |  |  | -0.019 | 0.009 | -.131 | .031 | .038 |
| Regression on residualized values | | | | | | | |
|  | *R²* = .093***; *F* (1, 138) = 15.19*** | 139 |  |  |  |  |  |
| Year of data collection^a^ (1982-2023) |  |  | -0.294 | 0.075 | -.359 | .099 | <.001 |
| *Note.* Variables were weighted based on sample size; all *R²* are adjusted values; *k* = number of samples; *b* = unstandardized regression coefficient; *SE* = standard error of unstandardized coefficient; β = standardized regression coefficient; $\eta_{p}^{2}$ = partial eta squared; Variance Inflation Factors (VIFs) in multiple Regression were all <1.1.  ^a^ Adjusted for confounding variables (Percentage of women in sample & sample mean age).  ****p* <.001. | | | | | | | |

| **Table S9**  *Single and multiple linear weighted meta-regression as well as residualized regressions for Asian samples on forced choice-based NPI-40 scores from 1982 to 2023.* | | | | | | | |
| --- | --- | --- | --- | --- | --- | --- | --- |
| Predictors | Model fit | *k* | *b* | *SE* | β | $\eta_{p}^{2}$ | *p* |
| Single regression | | | | | | | |
|  | *R²* = .017; *F* (1, 50) = 1.901 | 51 |  |  |  |  |  |
| Year of data collection (1982-2023) |  |  | -0.107 | 0.078 | -.189 | .036 | .174 |
| Multiple regression | | | | | | | |
|  | *R²* = .121*; *F* (3, 40) = 2.978* | 43 |  |  |  |  |  |
| Year of data collection (1982-2023) |  |  | -0.124 | 0.086 | -.217 | .012 | .162 |
| Sample mean age |  |  | -0.219 | 0.078 | -.413 | .155 | .008 |
| Percentage of women in sample |  |  | -0.013 | 0.012 | -.130 | .027 | .299 |
| Regression on residualized values | | | | | | | |
|  | *R²* = .022; *F* (1, 42) = 43.4 | 43 |  |  |  |  |  |
| Year of data collection^a^ (1982-2023) |  |  | -0.116 | 0.082 | -.186 | .045 | .167 |
| *Note.* Variables were weighted based on sample size; all *R²* are adjusted values; *k* = number of samples; *b* = unstandardized regression coefficient; *SE* = standard error of unstandardized coefficient; β = standardized regression coefficient; $\eta_{p}^{2}$ = partial eta squared; Variance Inflation Factors (VIFs) in multiple Regression were all <1.1.  ^a^ Adjusted for confounding variables (Percentage of women in sample & sample mean age).  **p* <.05. | | | | | | | |

| **Table S10**  *Single and multiple linear weighted meta-regression as well as residualized regressions for Asian student samples on any NPI scores from 1982 to 2023.* | | | | | | | |
| --- | --- | --- | --- | --- | --- | --- | --- |
| Predictors | Model fit | *k* | *b* | *SE* | β | $\eta_{p}^{2}$ | *p* |
| Single regression | | | | | | | |
|  | *R²* = <.001; *F* (1, 67) = 0.045 | 68 |  |  |  |  |  |
| Year of data collection (1982-2023) |  |  | 0.019 | .087 | .032 | <.001 | .083 |
| Multiple regression | | | | | | | |
|  | *R²* = .130**; *F* (3, 57) = 3.978** | 60 |  |  |  |  |  |
| Year of data collection (1982-2023) |  |  | -0.070 | 0.076 | -.130 | .004 | .364 |
| Sample mean age |  |  | 0.535 | 0.164 | -.374 | .163 | .020 |
| Percentage of women in sample |  |  | -0.012 | 0.016 | -.112 | .012 | .429 |
| Regression on residualized values | | | | | | | |
|  | *R²* = .002; *F* (1, 59) = 0.851 | 60 |  |  |  |  |  |
| Year of data collection^a^ (1982-2023) |  |  | -0.069 | 0.074 | -.144 | .014 | .360 |
| *Note.* Variables were weighted based on sample size; all *R²* are adjusted values; *k* = number of samples; *b* = unstandardized regression coefficient; *SE* = standard error of unstandardized coefficient; β = standardized regression coefficient; $\eta_{p}^{2}$ = partial eta squared; Variance Inflation Factors (VIFs) in multiple Regression were all <1.1.  ^a^ Adjusted for confounding variables (Percentage of women in sample & sample mean age).  ***p* <.01. | | | | | | | |

| **Table S11**  *Single and multiple linear weighted meta-regression as well as residualized regressions for Asian student samples on forced-choice-based NPI-40 scores from 1982 to 2023.* | | | | | | | |
| --- | --- | --- | --- | --- | --- | --- | --- |
| Predictors | Model fit | *k* | *b* | *SE* | β | $\eta_{p}^{2}$ | *p* |
| Single regression | | | | | | | |
|  | *R²* = <.001; *F* (1, 23) = 0.986 | 24 |  |  |  |  |  |
| Year of data collection (1982-2023) |  |  | -0.058 | .058 | -.202 | .004 | .331 |
| Multiple regression | | | | | | | |
|  | *R²* = .120; *F* (3, 19) = 2.830 | 22 |  |  |  |  |  |
| Year of data collection (1982-2023) |  |  | -0.099 | 0.061 | -.338 | .030 | .124 |
| Sample mean age |  |  | 0.757 | 0.299 | .649 | .276 | .020 |
| Percentage of women in sample |  |  | -0.011 | 0.014 | -.176 | .034 | .424 |
| Regression on residualized values | | | | | | | |
|  | *R²* = .065; *F* (1, 21) = 2.53 | 22 |  |  |  |  |  |
| Year of data collection^a^ (1982-2023) |  |  | -0.088 | 0.055 | -.337 | .107 | .128 |
| *Note.* Variables were weighted based on sample size; all *R²* are adjusted values; *k* = number of samples; *b* = unstandardized regression coefficient; *SE* = standard error of unstandardized coefficient; β = standardized regression coefficient; $\eta_{p}^{2}$ = partial eta squared; Variance Inflation Factors (VIFs) in multiple Regression were all <1.1.  ^a^ Adjusted for confounding variables (Percentage of women in sample & sample mean age). | | | | | | | |

| **Table S12**  *Single and multiple linear weighted meta-regression as well as residualized regressions for European samples on any NPI scores from 1982 to 2023.* | | | | | | | |
| --- | --- | --- | --- | --- | --- | --- | --- |
| Predictors | Model fit | *k* | *b* | *SE* | β | $\eta_{p}^{2}$ | *p* |
| Single regression | | | | | | | |
|  | *R²* = .037***; *F* (1, 428) = 17.08*** | 429 |  |  |  |  |  |
| Year of data collection (1982-2023) |  |  | -0.213 | 0.052 | -.242 | .038 | <.001 |
| Multiple regression | | | | | | | |
|  | *R²* = .081***; *F* (3, 387) = 12.49*** | 390 |  |  |  |  |  |
| Year of data collection (1982-2023) |  |  | -0.296 | 0.570 | -.306 | .068 | <.001 |
| Sample mean age |  |  | -0.023 | 0.024 | -.044 | .006 | .351 |
| Percentage of women in sample |  |  | -0.022 | 0.009 | -.152 | .017 | .009 |
| Regression on residualized values | | | | | | | |
|  | *R²* = .006***; *F* (1, 389) = 26.78*** | 390 |  |  |  |  |  |
| Year of data collection^a^ (1982-2023) |  |  | -0.291 | 0.056 | -.302 | .0644 | <.001 |
| *Note.* Variables were weighted based on sample size; all *R²* are adjusted values; *k* = number of samples; *b* = unstandardized regression coefficient; *SE* = standard error of unstandardized coefficient; β = standardized regression coefficient; $\eta_{p}^{2}$ = partial eta squared; Variance Inflation Factors (VIFs) in multiple Regression were all <1.1.  ^a^ Adjusted for confounding variables (Percentage of women in sample & sample mean age).  ****p* <.001. | | | | | | | |

| **Table S13**  *Single and multiple linear weighted meta-regression as well as residualized regressions for European samples on forced choice-based NPI-40 scores from 1982 to 2023.* | | | | | | | |
| --- | --- | --- | --- | --- | --- | --- | --- |
| Predictors | Model fit | *k* | *b* | *SE* | β | $\eta_{p}^{2}$ | *p* |
| Single regression | | | | | | | |
|  | *R²* = .183***; *F* (1, 150) = 34.78*** | 151 |  |  |  |  |  |
| Year of data collection (1982-2023) |  |  | -0.379 | 0.064 | -.559 | .188 | <.001 |
| Multiple regression | | | | | | | |
|  | *R²* = .271***; *F* (3, 131) = 17.62*** | 134 |  |  |  |  |  |
| Year of data collection (1982-2023) |  |  | -0.410 | 0.066 | -.569 | .236 | <.001 |
| Sample mean age |  |  | -0.057 | 0.044 | -.137 | .027 | .201 |
| Percentage of women in sample |  |  | -0.034 | 0.115 | -.341 | .062 | <.001 |
| Regression on residualized values | | | | | | | |
|  | *R²* = .022***; *F* (1, 133) = 38.68*** | 134 |  |  |  |  |  |
| Year of data collection^a^ (1982-2023) |  |  | -0.409 | 0.066 | -.576 | .022 | <.001 |
| *Note.* Variables were weighted based on sample size; all *R²* are adjusted values; *k* = number of samples; *b* = unstandardized regression coefficient; *SE* = standard error of unstandardized coefficient; β = standardized regression coefficient; $\eta_{p}^{2}$ = partial eta squared; Variance Inflation Factors (VIFs) in multiple Regression were all <1.1.  ^a^ Adjusted for confounding variables (Percentage of women in sample & sample mean age).  ****p* <.001. | | | | | | | |

| **Table S14**  *Single and multiple linear weighted meta-regression as well as residualized regressions for European student samples on any NPI scores from 1982 to 2023.* | | | | | | | |
| --- | --- | --- | --- | --- | --- | --- | --- |
| Predictors | Model fit | *k* | *b* | *SE* | β | $\eta_{p}^{2}$ | *p* |
| Single regression | | | | | | | |
|  | *R²* = .037*; *F* (1, 140) = 6.43* | 141 |  |  |  |  |  |
| Year of data collection (1982-2023) |  |  | -.213 | .083 | -.241 | .043 | .001 |
| Multiple regression | | | | | | | |
|  | *R²* = .069**; *F* (3, 128) = 4.24* | 131 |  |  |  |  |  |
| Year of data collection (1982-2023) |  |  | -0.220 | 0.088 | -.247 | .050 | .013 |
| Sample mean age |  |  | -0.085 | 0.094 | -.066 | .005 | .368 |
| Percentage of women in sample |  |  | -0.030 | 0.013 | -.204 | .039 | .025 |
| Regression on residualized values | | | | | | | |
|  | *R²* = .039**; *F* (1, 130) = 6.378** | 131 |  |  |  |  |  |
| Year of data collection^a^ (1982-2023) |  |  | -0.220 | 0.087 | -.243 | .047 | .013 |
| *Note.* Variables were weighted based on sample size; all *R²* are adjusted values; *k* = number of samples; *b* = unstandardized regression coefficient; *SE* = standard error of unstandardized coefficient; β = standardized regression coefficient; $\eta_{p}^{2}$ = partial eta squared; Variance Inflation Factors (VIFs) in multiple Regression were all <1.1.  ^a^ Adjusted for confounding variables (Percentage of women in sample & sample mean age).  **p*<.05; ***p* <.01. | | | | | | | |

| **Table S15**  *Single and multiple linear weighted meta-regression as well as residualized regressions for European student samples on forced-choice-based NPI-40 scores from 1982 to 2023.* | | | | | | | |
| --- | --- | --- | --- | --- | --- | --- | --- |
| Predictors | Model fit | *k* | *b* | *SE* | β | $\eta_{p}^{2}$ | *p* |
| Single regression | | | | | | | |
|  | *R²* = .011; *F* (1, 53) = 1.60 | 54 |  |  |  |  |  |
| Year of data collection (1982-2023) |  |  | -.095 | .075 | -.152 | .030 | .211 |
| Multiple regression | | | | | | | |
|  | *R²* = .007; *F* (3, 47) = 1.12 | 50 |  |  |  |  |  |
| Year of data collection (1982-2023) |  |  | -0.106 | 0.079 | -.192 | .030 | .185 |
| Sample mean age |  |  | 0.011 | 0.090 | -.142 | .023 | .903 |
| Percentage of women in sample |  |  | -0.017 | 0.013 | -.407 | .102 | .193 |
| Regression on residualized values | | | | | | | |
|  | *R²* = .017; *F* (1, 49) = 1.87 | 50 |  |  |  |  |  |
| Year of data collection^a^ (1982-2023) |  |  | -0.106 | 0.077 | -.169 | .037 | .177 |
| *Note.* Variables were weighted based on sample size; all *R²* are adjusted values; *k* = number of samples; *b* = unstandardized regression coefficient; *SE* = standard error of unstandardized coefficient; β = standardized regression coefficient; $\eta_{p}^{2}$ = partial eta squared; Variance Inflation Factors (VIFs) in multiple Regression were all <1.1.  ^a^ Adjusted for confounding variables (Percentage of women in sample & sample mean age). | | | | | | | |

| **Table S16**  *Single and multiple linear weighted meta-regression as well as residualized regressions for North American samples on any NPI scores from 1982 to 2023.* | | | | | | | |
| --- | --- | --- | --- | --- | --- | --- | --- |
| Predictors | Model fit | *k* | *b* | *SE* | β | $\eta_{p}^{2}$ | *p* |
| Single regression | | | | | | | |
|  | *R²* = .060***; *F* (1, 862) = 55.77*** | 863 |  |  |  |  |  |
| Year of data collection (1982-2023) |  |  | -0.115 | 0.015 | -.263 | .060 | <.001 |
| Multiple regression | | | | | | | |
|  | *R²* = .099***; *F* (3, 655) = 25.12*** | 658 |  |  |  |  |  |
| Year of data collection (1982-2023) |  |  | -0.075 | 0.019 | -.157 | .038 | <.001 |
| Sample mean age |  |  | -0.106 | 0.016 | -.241 | .050 | <.001 |
| Percentage of women in sample |  |  | -0.022 | 0.006 | -.176 | .022 | <.001 |
| Regression on residualized values | | | | | | | |
|  | *R²* = .020***; *F* (1, 657) = 14.68*** | 658 |  |  |  |  |  |
| Year of data collection^a^ (1982-2023) |  |  | -0.072 | 0.018 | -.161 | .029 | <.001 |
| *Note.* Variables were weighted based on sample size; all *R²* are adjusted values; *k* = number of samples; *b* = unstandardized regression coefficient; *SE* = standard error of unstandardized coefficient; β = standardized regression coefficient; $\eta_{p}^{2}$ = partial eta squared; Variance Inflation Factors (VIFs) in multiple Regression were all <1.1.  ^a^ Adjusted for confounding variables (Percentage of women in sample & sample mean age).  ****p* <.001. | | | | | | | |

| **Table S17**  *Single and multiple linear weighted meta-regression as well as residualized regressions for North American samples on forced choice-based NPI-40 scores from 1982 to 2023.* | | | | | | | |
| --- | --- | --- | --- | --- | --- | --- | --- |
| Predictors | Model fit | *k* | *b* | *SE* | β | $\eta_{p}^{2}$ | *p* |
| Single regression | | | | | | | |
|  | *R²* = .070***; *F* (1, 541) = 39.72*** | 542 |  |  |  |  |  |
| Year of data collection (1982-2023) |  |  | -0.100 | 0.016 | -.268 | .069 | <.001 |
| Multiple regression | | | | | | | |
|  | *R²* = .188***; *F* (3, 398) = 30.09*** | 401 |  |  |  |  |  |
| Year of data collection (1982-2023) |  |  | -0.087 | 0.019 | -.206 | .070 | <.001 |
| Sample mean age |  |  | -0.119 | 0.018 | -.289 | .090 | <.001 |
| Percentage of women in sample |  |  | -0.028 | 0.006 | -.276 | .052 | <.001 |
| Regression on residualized values | | | | | | | |
|  | *R²* = .046***; *F* (1, 400) = 20.33*** | 401 |  |  |  |  |  |
| Year of data collection^a^ (1982-2023) |  |  | -0.086 | 0.019 | -.220 | .049 | <.001 |
| *Note.* Variables were weighted based on sample size; all *R²* are adjusted values; *k* = number of samples; *b* = unstandardized regression coefficient; *SE* = standard error of unstandardized coefficient; β = standardized regression coefficient; $\eta_{p}^{2}$ = partial eta squared; Variance Inflation Factors (VIFs) in multiple Regression were all <1.1.  ^a^ Adjusted for confounding variables (Percentage of women in sample & sample mean age).  ****p* <.001. | | | | | | | |

| **Table S18**  *Single and multiple linear weighted meta-regression as well as residualized regressions for North American student samples on any NPI scores from 1982 to 2023.* | | | | | | | |
| --- | --- | --- | --- | --- | --- | --- | --- |
| Predictors | Model fit | *k* | *b* | *SE* | β | $\eta_{p}^{2}$ | *p* |
| Single regression | | | | | | | |
|  | *R²* = .038***; *F* (1, 627) = 25.7*** | 628 |  |  |  |  |  |
| Year of data collection (1982-2023) |  |  | -.080 | .016 | -.205 | .040 | .001 |
| Multiple regression | | | | | | | |
|  | *R²* = .070***; *F* (3, 455) = 12.52*** | 458 |  |  |  |  |  |
| Year of data collection (1982-2023) |  |  | -0.065 | 0.020 | -.154 | .034 | <.001 |
| Sample mean age |  |  | -0.256 | 0.098 | -.162 | .017 | .009 |
| Percentage of women in sample |  |  | -0.025 | 0.007 | -.219 | .029 | <.001 |
| Regression on residualized values | | | | | | | |
|  | *R²* = .020**; *F* (1, 457) = 10.30** | 458 |  |  |  |  |  |
| Year of data collection^a^ (1982-2023) |  |  | -0.064 | 0.020 | -.153 | .022 | .001 |
| *Note.* Variables were weighted based on sample size; all *R²* are adjusted values; *k* = number of samples; *b* = unstandardized regression coefficient; *SE* = standard error of unstandardized coefficient; β = standardized regression coefficient; $\eta_{p}^{2}$ = partial eta squared; Variance Inflation Factors (VIFs) in multiple Regression were all <1.1.  ^a^ Adjusted for confounding variables (Percentage of women in sample & sample mean age).  ***p* <.01; ****p* <.001. | | | | | | | |

| **Table S19**  *Single and multiple linear weighted meta-regression as well as residualized regressions for North American student samples on forced-choice-based NPI-40 scores from 1982 to 2023.* | | | | | | | |
| --- | --- | --- | --- | --- | --- | --- | --- |
| Predictors | Model fit | *k* | *b* | *SE* | β | $\eta_{p}^{2}$ | *p* |
| Single regression | | | | | | | |
|  | *R²* = .0325***; *F* (1, 428) = 11.86*** | 429 |  |  |  |  |  |
| Year of data collection (1982-2023) |  |  | -.057 | .017 | -.163 | .028 | <.001 |
| Multiple regression | | | | | | | |
|  | *R²* = .105***; *F* (3, 302) = 13.00*** | 305 |  |  |  |  |  |
| Year of data collection (1982-2023) |  |  | -0.068 | 0.023 | -.163 | .004 | .003 |
| Sample mean age |  |  | 0.018 | 0.135 | -.009 | <.001 | .892 |
| Percentage of women in sample |  |  | -0.035 | 0.007 | -.358 | .075 | <.001 |
| Regression on residualized values | | | | | | | |
|  | *R²* = .026**; *F* (1, 304) = 9.03** | 305 |  |  |  |  |  |
| Year of data collection^a^ (1982-2023) |  |  | -0.067 | 0.022 | -.165 | .029 | .002 |
| *Note.* Variables were weighted based on sample size; all *R²* are adjusted values; *k* = number of samples; *b* = unstandardized regression coefficient; *SE* = standard error of unstandardized coefficient; β = standardized regression coefficient; $\eta_{p}^{2}$ = partial eta squared; Variance Inflation Factors (VIFs) in multiple Regression were all <1.1.  ^a^ Adjusted for confounding variables (Percentage of women in sample & sample mean age).  ***p* <.01; ****p* <.001. | | | | | | | |

| **Table S20**  *Single and multiple linear weighted meta-regression as well as residualized regressions for Oceanian samples on any NPI scores from 1982 to 2023.* | | | | | | | |
| --- | --- | --- | --- | --- | --- | --- | --- |
| Predictors | Model fit | *k* | *b* | *SE* | β | $\eta_{p}^{2}$ | *p* |
| Single regression | | | | | | | |
|  | *R²* = .052; *F* (1, 52) = 3.872 | 53 |  |  |  |  |  |
| Year of data collection (1982-2023) |  |  | -0.220 | 0.112 | -.294 | .069 | .054 |
| Multiple regression | | | | | | | |
|  | *R²* = .126*; *F* (3, 47) = 3.41* | 50 |  |  |  |  |  |
| Year of data collection (1982-2023) |  |  | -0.190 | 0.109 | -.262 | .076 | .087 |
| Sample mean age |  |  | -0.227 | 0.092 | -.468 | .088 | .018 |
| Percentage of women in sample |  |  | -0.038 | 0.028 | -.295 | .037 | .184 |
| Regression on residualized values | | | | | | | |
|  | *R²* = .042; *F* (1, 49) = 3.16 | 50 |  |  |  |  |  |
| Year of data collection^a^ (1982-2023) |  |  | -0.060 | 0.106 | -.286 | .061 | .082 |
| *Note.* Variables were weighted based on sample size; all *R²* are adjusted values; *k* = number of samples; *b* = unstandardized regression coefficient; *SE* = standard error of unstandardized coefficient; β = standardized regression coefficient; $\eta_{p}^{2}$ = partial eta squared; Variance Inflation Factors (VIFs) in multiple Regression were all <1.1.  ^a^ Adjusted for confounding variables (Percentage of women in sample & sample mean age).  **p* <.05. | | | | | | | |

| **Table S21**  *Single and multiple linear weighted meta-regression as well as residualized regressions for Oceanian samples on forced choice-based NPI-40 scores from 1982 to 2023.* | | | | | | | |
| --- | --- | --- | --- | --- | --- | --- | --- |
| Predictors | Model fit | *k* | *b* | *SE* | β | $\eta_{p}^{2}$ | *p* |
| Single regression | | | | | | | |
|  | *R²* = -.051; *F* (1, 19) = 0.024 | 20 |  |  |  |  |  |
| Year of data collection (1982-2023) |  |  | -0.022 | 0.144 | -.041 | .001 | .878 |
| Multiple regression | | | | | | | |
|  | *R²* = .375*; *F* (3, 16) = 4.80* | 19 |  |  |  |  |  |
| Year of data collection (1982-2023) |  |  | <0.001 | <0.001 | .156 | .001 | .481 |
| Sample mean age |  |  | <0.001 | <0.001 | -.928 | .047 | .003 |
| Percentage of women in sample |  |  | <0.001 | <0.001 | -.030 | .007 | .915 |
| Regression on residualized values | | | | | | | |
|  | *R²* = -.024; *F* (1, 18) = 0.55 | 19 |  |  |  |  |  |
| Year of data collection^a^ (1982-2023) |  |  | -0.079 | 0.106 | -.165 | .030 | .467 |
| *Note.* Variables were weighted based on sample size; all *R²* are adjusted values; *k* = number of samples; *b* = unstandardized regression coefficient; *SE* = standard error of unstandardized coefficient; β = standardized regression coefficient; $\eta_{p}^{2}$ = partial eta squared; Variance Inflation Factors (VIFs) in multiple Regression were all <1.1.  ^a^ Adjusted for confounding variables (Percentage of women in sample & sample mean age).  **p* <.05. | | | | | | | |

| **Table S22**  *Single and multiple linear weighted meta-regression as well as residualized regressions for U.S.-based student samples on forced choice-based NPI-40 scores before the global financial crisis (until 2008).* | | | | | | | |
| --- | --- | --- | --- | --- | --- | --- | --- |
| Predictors | Model fit | *k* | *b* | *SE* | β | $\eta_{p}^{2}$ | *p* |
| Single regression | | | | | | | |
|  | *R²* = <.001; *F* (1, 183) = 0.002 | 184 |  |  |  |  |  |
| Year of data collection (1982-2008) |  |  | -0.001 | 0.024 | -.003 | <.001 | .965 |
| Multiple regression | | | | | | | |
|  | *R²* = .130**; *F* (3, 97) = 5.99** | 100 |  |  |  |  |  |
| Year of data collection (1982-2008) |  |  | 0.047 | 0.030 | .151 | .022 | .113 |
| Sample mean age |  |  | 0.038 | 0.138 | .034 | .001 | .782 |
| Percentage of women in sample |  |  | -0.024 | 0.006 | -.449 | .139 | <.001 |
| Regression on residualized values | | | | | | | |
|  | *R²* = .016; *F* (1, 99) = 2.61 | 100 |  |  |  |  |  |
| Year of data collection^a^ (1982-2008) |  |  | 0.047 | 0.029 | .158 | .026 | .109 |
| *Note.* Variables were weighted based on sample size; all *R²* are adjusted values; *k* = number of samples; *b* = unstandardized regression coefficient; *SE* = standard error of unstandardized coefficient; β = standardized regression coefficient; $\eta_{p}^{2}$ = partial eta squared; Variance Inflation Factors (VIFs) in multiple Regression were all <1.1.  ^a^ Adjusted for confounding variables (Percentage of women in sample & sample mean age).  ***p*<.01. | | | | | | | |

| **Table S23**  *Single and multiple linear weighted meta-regression as well as residualized regressions for U.S.-based samples any NPI scores before the global financial crisis (until 2008).* | | | | | | | |
| --- | --- | --- | --- | --- | --- | --- | --- |
| Predictors | Model fit | *k* | *b* | *SE* | β | $\eta_{p}^{2}$ | *p* |
| Single regression | | | | | | | |
|  | *R²* = .010*; *F* (1, 284) = 3.98* | 285 |  |  |  |  |  |
| Year of data collection (1982-2008) |  |  | 0.042 | 0.021 | .116 | .014 | .047 |
| Multiple regression | | | | | | | |
|  | *R²* = .199***; *F* (3, 162) = 14.68*** | 165 |  |  |  |  |  |
| Year of data collection (1982-2008) |  |  | <0.001 | <0.001 | .303 | .073 | <.001 |
| Sample mean age |  |  | -<0.001 | <0.001 | -.389 | .123 | <.001 |
| Percentage of women in sample |  |  | -<0.001 | <0.001 | -.223 | .048 | <.001 |
| Regression on residualized values | | | | | | | |
|  | *R²* = .086***; *F* (1, 164) = 16.45*** | 165 |  |  |  |  |  |
| Year of data collection^a^ (1982-2008) |  |  | 0.102 | 0.025 | .318 | .091 | <.001 |

| *Note.* Variables were weighted based on sample size; all *R²* are adjusted values; *k* = number of samples; *b* = unstandardized regression coefficient; *SE* = standard error of unstandardized coefficient; β = standardized regression coefficient; $\eta_{p}^{2}$ = partial eta squared; Variance Inflation Factors (VIFs) in multiple Regression were all <1.1.  ^a^ Adjusted for confounding variables (Percentage of women in sample & sample mean age).  **p*<.05; ****p* <.001. |
| --- |

| **Table S24**  *Single and multiple linear weighted meta-regression as well as residualized regressions for U.S.-based samples force-choice-based NPI-40 before the global financial crisis (until 2008).* | | | | | | | |
| --- | --- | --- | --- | --- | --- | --- | --- |
| Predictors | Model fit | *k* | *b* | *SE* | β | $\eta_{p}^{2}$ | *p* |
| Single regression | | | | | | | |
|  | *R²* = -.003; *F* (1, 210) = 0.28 | 211 |  |  |  |  |  |
| Year of data collection (1982-2008) |  |  | -0.012 | 0.023 | -.036 | .001 | .599 |
| Multiple regression | | | | | | | |
|  | *R²* = .020***; *F* (3, 117) = 11.15*** | 120 |  |  |  |  |  |
| Year of data collection (1982-2008) |  |  | 0.050 | 0.028 | .146 | .021 | .074 |
| Sample mean age |  |  | 0.097 | 0.031 | -.316 | .111 | .002 |
| Percentage of women in sample |  |  | -0.231 | 0.006 | -.387 | .012 | <.001 |
| Regression on residualized values | | | | | | | |
|  | *R²* = .019; *F* (1, 119) = 3.29 | 120 |  |  |  |  |  |
| Year of data collection^a^ (1982-2008) |  |  | 0.050 | 0.028 | .161 | .027 | .072 |
| *Note.* Variables were weighted based on sample size; all *R²* are adjusted values; *k* = number of samples; *b* = unstandardized regression coefficient; *SE* = standard error of unstandardized coefficient; β = standardized regression coefficient; $\eta_{p}^{2}$ = partial eta squared; Variance Inflation Factors (VIFs) in multiple Regression were all <1.1.  ^a^ Adjusted for confounding variables (Percentage of women in sample & sample mean age).  ****p* <.001. | | | | | | | |

| **Table S25**  *Single and multiple linear weighted meta-regression as well as residualized regressions for U.S.-based student samples on any NPI scores before the global financial crisis (until 2008).* | | | | | | | |
| --- | --- | --- | --- | --- | --- | --- | --- |
| Predictors | Model fit | *k* | *b* | *SE* | β | $\eta_{p}^{2}$ | *p* |
| Single regression | | | | | | | |
|  | *R²* = .009; *F* (1, 251) = 3.83 | 252 |  |  |  |  |  |
| Year of data collection (1982-2008) |  |  | 0.038 | 0.020 | .113 | .013 | .067 |
| Multiple regression | | | | | | | |
|  | *R²* = .152***; *F* (3, 137) = 9.35*** | 140 |  |  |  |  |  |
| Year of data collection (1982-2008) |  |  | <0.001 | <0.001 | .338 | .104 | <.001 |
| Sample mean age |  |  | -<0.001 | <0.001 | -.141 | .011 | .196 |
| Percentage of women in sample |  |  | -<0.001 | <0.001 | -.296 | .071 | .001 |
| Regression on residualized values | | | | | | | |
|  | *R²* = .109***; *F* (1, 139) = 18.05*** | 140 |  |  |  |  |  |
| Year of data collection^a^ (1982-2008) |  |  | 0.106 | 0.025 | .353 | .115 | <.001 |
| *Note.* Variables were weighted based on sample size; all *R²* are adjusted values; *k* = number of samples; *b* = unstandardized regression coefficient; *SE* = standard error of unstandardized coefficient; β = standardized regression coefficient; $\eta_{p}^{2}$ = partial eta squared; Variance Inflation Factors (VIFs) in multiple Regression were all <1.1.  ^a^ Adjusted for confounding variables (Percentage of women in sample & sample mean age).  ****p* <.001. | | | | | | | |

| **Table S26**  *Single and multiple linear weighted meta-regression as well as residualized regressions for global samples on any NPI scores before the global financial crisis (until 2008).* | | | | | | | |
| --- | --- | --- | --- | --- | --- | --- | --- |
| Predictors | Model fit | *k* | *b* | *SE* | β | $\eta_{p}^{2}$ | *p* |
| Single regression | | | | | | | |
|  | *R*² = <.001; *F*(1, 388) = 0.84 | 389 |  |  |  |  |  |
| Year of data collection (1982-2008) |  |  | 0.021 | 0.023 | .048 | .002 | .360 |
| Multiple regression | | | | | | | |
|  | *R²* = .173***; *F* (3, 252) = 18.85*** | 255 |  |  |  |  |  |
| Year of data collection (1982-2008) |  |  | <0.001 | <0.001 | .196 | .027 | .001 |
| Sample mean age |  |  | -<0.001 | <0.001 | -.393 | .145 | <.001 |
| Percentage of women in sample |  |  | -<0.001 | <0.001 | -.171 | .026 | .009 |
| Regression on residualized values | | | | | | | |
|  | *R²* = .037**; *F* (1, 254) = 10.93** | 255 |  |  |  |  |  |
| Year of data collection^a^ (1982-2008) |  |  | 0.083 | 0.025 | .199 | .041 | .001 |
| *Note.* Variables were weighted based on sample size; all *R²* are adjusted values; *k* = number of samples; *b* = unstandardized regression coefficient; *SE* = standard error of unstandardized coefficient; β = standardized regression coefficient; $\eta_{p}^{2}$ = partial eta squared; Variance Inflation Factors (VIFs) in multiple Regression were all <1.1.  ^a^ Adjusted for confounding variables (Percentage of women in sample & sample mean age, Scale, Items & Sample).  ***p*<.01; ****p* <.001. | | | | | | | |

| **Table S27**  *Single and multiple linear weighted meta-regression as well as residualized regressions for global samples on forced choice-based NPI-40 scores before the global financial crisis (until 2008).* | | | | | | | |
| --- | --- | --- | --- | --- | --- | --- | --- |
| Predictors | Model fit | *k* | *b* | *SE* | β | $\eta_{p}^{2}$ | *p* |
| Single regression | | | | | | | |
|  | *R²* = -.002; *F* (1, 276) = 0.38 | 277 |  |  |  |  |  |
| Year of data collection (1982-2008) |  |  | 0.015 | 0.024 | .037 | .001 | .540 |
| Multiple regression | | | | | | | |
|  | *R²* = .192***; *F* (3, 174) = 15.10*** | 177 |  |  |  |  |  |
| Year of data collection (1982-2008) |  |  | 0.055 | 0.028 | .131 | .019 | .053 |
| Sample mean age |  |  | -0.132 | 0.025 | -.295 | .015 | <.001 |
| Percentage of women in sample |  |  | -0.019 | 0.006 | -.272 | .061 | <.001 |
| Regression on residualized values | | | | | | | |
|  | *R²* = .016; *F* (1, 176) = 3.82 | 177 |  |  |  |  |  |
| Year of data collection^a^ (1982-2008) |  |  | 0.055 | 0.028 | .137 | .021 | .052 |
| *Note.* Variables were weighted based on sample size; all *R²* are adjusted values; *k* = number of samples; *b* = unstandardized regression coefficient; *SE* = standard error of unstandardized coefficient; β = standardized regression coefficient; $\eta_{p}^{2}$ = partial eta squared; Variance Inflation Factors (VIFs) in multiple Regression were all <1.1.  ^a^ Adjusted for confounding variables (Percentage of women in sample & sample mean age).  ****p* <.001. | | | | | | | |

| **Table S28**  *Single and multiple linear weighted meta-regression as well as residualized regressions for global student samples on any NPI scores before the global financial crisis (until 2008).* | | | | | | | |
| --- | --- | --- | --- | --- | --- | --- | --- |
| Predictors | Model fit | *k* | *b* | *SE* | β | $\eta_{p}^{2}$ | *p* |
| Single regression | | | | | | | |
|  | *R²* = .012*; *F* (1, 305) = 4.61* | 306 |  |  |  |  |  |
| Year of data collection (1982-2008) |  |  | .043 | 0.020 | .119 | .015 | .033 |
| Multiple regression | | | | | | | |
|  | *R²* = .118***; *F* (3, 191) = 9.62*** | 194 |  |  |  |  |  |
| Year of data collection (1982-2008) |  |  | <0.001 | <0.001 | .282 | .075 | <.001 |
| Sample mean age |  |  | -<0.001 | <0.001 | -.105 | .008 | .217 |
| Percentage of women in sample |  |  | -<0.001 | <0.001 | -.058 | .058 | <.001 |
| Regression on residualized values | | | | | | | |
|  | *R²* = .078***; *F* (1, 193) = 17.50*** | 194 |  |  |  |  |  |
| Year of data collection^a^ (1982-2008) |  |  | 0.100 | 0.023 | .288 | .083 | <.001 |
| *Note.* Variables were weighted based on sample size; all *R²* are adjusted values; *k* = number of samples; *b* = unstandardized regression coefficient; *SE* = standard error of unstandardized coefficient; β = standardized regression coefficient; $\eta_{p}^{2}$ = partial eta squared; Variance Inflation Factors (VIFs) in multiple Regression were all <1.1.  ^a^ Adjusted for confounding variables (Percentage of women in sample & sample mean age).  **p*<.05; ****p* <.001. | | | | | | | |

| **Table S29**  *Single and multiple linear weighted meta-regression as well as residualized regressions for global student samples on forced choice-based NPI-40 scores before the global financial crisis (until 2008).* | | | | | | | |
| --- | --- | --- | --- | --- | --- | --- | --- |
| Predictors | Model fit | *k* | *b* | *SE* | β | $\eta_{p}^{2}$ | *p* |
| Single regression | | | | | | | |
|  | *R²* = -.003; *F* (1, 219) = 0.36 | 220 |  |  |  |  |  |
| Year of data collection (1982-2008) |  |  | 0.013 | 0.022 | .042 | .002 | .552 |
| Multiple regression | | | | | | | |
|  | *R²* = .147; *F* (3, 133) = 8.80*** | 136 |  |  |  |  |  |
| Year of data collection (1982-2008) |  |  | 0.057 | 0.026 | .170 | .033 | .032 |
| Sample mean age |  |  | -0.151 | 0.103 | -.140 | .020 | .143 |
| Percentage of women in sample |  |  | -0.024 | 0.006 | -.426 | .125 | <.001 |
| Regression on residualized values | | | | | | | |
|  | *R²* = .027*; *F* (1, 135) = 4.76* | 136 |  |  |  |  |  |
| Year of data collection^a^ (1982-2008) |  |  | 0.057 | 0.026 | .181 | .034 | .030 |
| *Note.* Variables were weighted based on sample size; all *R²* are adjusted values; *k* = number of samples; *b* = unstandardized regression coefficient; *SE* = standard error of unstandardized coefficient; β = standardized regression coefficient; $\eta_{p}^{2}$ = partial eta squared; Variance Inflation Factors (VIFs) in multiple Regression were all <1.1.  ^a^ Adjusted for confounding variables (Percentage of women in sample & sample mean age).  **p*<.05; ****p* <.001. | | | | | | | |

| **Table S30**  *Single and multiple linear weighted meta-regression as well as residualized regressions for U.S.-based student samples on forced choice-based NPI-40 scores after the global financial crisis (since 2008).* | | | | | | | |
| --- | --- | --- | --- | --- | --- | --- | --- |
| Predictors | Model fit | *k* | *b* | *SE* | β | $\eta_{p}^{2}$ | *p* |
| Single regression | | | | | | | |
|  | *R²* = .048***; *F* (1, 206) = 11.33*** | 207 |  |  |  |  |  |
| Year of data collection (2008-2023) |  |  | -0.180 | 0.053 | -.193 | .052 | <.001 |
| Multiple regression | | | | | | | |
|  | *R²* = .116***; *F* (3, 164) = 8.29*** | 167 |  |  |  |  |  |
| Year of data collection (2008-2023) |  |  | -0.158 | 0.061 | -.164 | .061 | .010 |
| Sample mean age |  |  | 0.179 | 0.224 | .060 | .008 | .423 |
| Percentage of women in sample |  |  | -0.045 | 0.013 | -.357 | .073 | <.001 |
| Regression on residualized values | | | | | | | |
|  | *R²* = .030*; *F* (1, 166) = 6.13* | 167 |  |  |  |  |  |
| Year of data collection^a^ (2008-2023) |  |  | -0.143 | 0.058 | -.153 | .036 | .014 |
| *Note.* Variables were weighted based on sample size; all *R²* are adjusted values; *k* = number of samples; *b* = unstandardized regression coefficient; *SE* = standard error of unstandardized coefficient; β = standardized regression coefficient; $\eta_{p}^{2}$ = partial eta squared; Variance Inflation Factors (VIFs) in multiple Regression were all <1.1.  ^a^ Adjusted for confounding variables (Percentage of women in sample & sample mean age).  **p*<.05; ****p*<.001. | | | | | | | |

| **Table S31**  *Single and multiple linear weighted meta-regression as well as residualized regressions for U.S.-based samples on any NPI scores after the global financial crisis (since 2008).* | | | | | | | | |
| --- | --- | --- | --- | --- | --- | --- | --- | --- |
| Predictors | Model fit | *k* | *b* | *SE* | β | $\eta_{p}^{2}$ | *p* | |
| Single regression | | | | | | | | |
|  | *R²* = .050***; *F* (1, 493) = 27.04*** | 494 |  |  |  |  |  | |
| Year of data collection (2008-2023) |  |  | -0.237 | 0.045 | -.214 | .052 | <.001 | |
| Multiple regression | | | | | | | | |
|  | *R²* = .088***; *F* (3, 408) = 14.28*** | 411 |  |  |  |  |  | |
| Year of data collection (2008-2023) |  |  | -0.104 | 0.046 | -.097 | .026 | .024 | |
| Sample mean age |  |  | -0.113 | 0.022 | -.244 | .041 | <.001 | |
| Percentage of women in sample |  |  | -0.034 | 0.009 | -.223 | .035 | <.001 | |
| Regression on residualized values | | | | | | | | |
|  | *R²* = .009*; *F* (1, 410) = 5.00* | 411 |  |  |  |  |  | |
| Year of data collection^a^ (2008-2023) |  |  | -0.101 | 0.045 | -.101 | .012 | .026 | |
| *Note.* Variables were weighted based on sample size; all *R²* are adjusted values; *k* = number of samples; *b* = unstandardized regression coefficient; *SE* = standard error of unstandardized coefficient; β = standardized regression coefficient; $\eta_{p}^{2}$ = partial eta squared; Variance Inflation Factors (VIFs) in multiple Regression were all <1.1.  ^a^ Adjusted for confounding variables (Percentage of women in sample & sample mean age).  **p*<.05; ****p* <.001.  . | | | | | | | |  |

| **Table S32**  *Single and multiple linear weighted meta-regression as well as residualized regressions for U.S.-based samples on force-choice-based NPI-40 after the global financial crisis (since 2008).* | | | | | | | |
| --- | --- | --- | --- | --- | --- | --- | --- |
| Predictors | Model fit | *k* | *b* | *SE* | β | $\eta_{p}^{2}$ | *p* |
| Single regression | | | | | | | |
|  | *R²* = .079***; *F* (1, 279) = 24.94*** | 280 |  |  |  |  |  |
| Year of data collection (2008-2023) |  |  | -0.251 | 0.050 | -.266 | .082 | <.001 |
| Multiple regression | | | | | | | |
|  | *R²* = .019***; *F* (3, 230) = 19.54*** | 233 |  |  |  |  |  |
| Year of data collection (2008-2023) |  |  | -0.136 | 0.050 | .144 | .064 | .007 |
| Sample mean age |  |  | -0.152 | 0.026 | -.318 | .101 | <.001 |
| Percentage of women in sample |  |  | -0.042 | 0.010 | -.323 | .069 | <.001 |
| Regression on residualized values | | | | | | | |
|  | *R²* = .026**; *F* (1, 232) = 7.12** | 233 |  |  |  |  |  |
| Year of data collection^a^ (2008-2023) |  |  | -0.130 | 0.048 | -.151 | .030 | .008 |
| *Note.* Variables were weighted based on sample size; all *R²* are adjusted values; *k* = number of samples; *b* = unstandardized regression coefficient; *SE* = standard error of unstandardized coefficient; β = standardized regression coefficient; $\eta_{p}^{2}$ = partial eta squared; Variance Inflation Factors (VIFs) in multiple Regression were all <1.1.  ^a^ Adjusted for confounding variables (Percentage of women in sample & sample mean age).  ***p*<.01; ****p* <.001. | | | | | | | |

| **Table S33**  *Single and multiple linear weighted meta-regression as well as residualized regressions for U.S.-based student samples on any NPI scores after the global financial crisis (since 2008).* | | | | | | | |
| --- | --- | --- | --- | --- | --- | --- | --- |
| Predictors | Model fit | *k* | *b* | *SE* | β | $\eta_{p}^{2}$ | *p* |
| Single regression | | | | | | | |
|  | *R²* = .055***; *F* (1, 317) = 19.59*** | 318 |  |  |  |  |  |
| Year of data collection (2008-2023) |  |  | -0.222 | 0.050 | -.213 | .059 | <.001 |
| Multiple regression | | | | | | | |
|  | *R²* = .095***; *F* (3, 255) = 10.05*** | 258 |  |  |  |  |  |
| Year of data collection (2008-2023) |  |  | -0.162 | 0.052 | -.158 | .038 | .002 |
| Sample mean age |  |  | 0.546 | 0.156 | .248 | .052 | <.001 |
| Percentage of women in sample |  |  | -0.028 | 0.011 | -.192 | .023 | .014 |
| Regression on residualized values | | | | | | | |
|  | *R²* = .030**; *F* (1, 257) = 9.08** | 258 |  |  |  |  |  |
| Year of data collection^a^ (2008-2023) |  |  | -0.154 | 0.051 | -.156 | .034 | .003 |
| *Note.* Variables were weighted based on sample size; all *R²* are adjusted values; *k* = number of samples; *b* = unstandardized regression coefficient; *SE* = standard error of unstandardized coefficient; β = standardized regression coefficient; $\eta_{p}^{2}$ = partial eta squared; Variance Inflation Factors (VIFs) in multiple Regression were all <1.1.  ^a^ Adjusted for confounding variables (Percentage of women in sample & sample mean age).  ** *p* <.01; ****p* <.001. | | | | | | | |

| **Table S34**  *Single and multiple linear weighted meta-regression as well as residualized regressions for global samples on any NPI scores after the global financial crisis (since 2008).* | | | | | | | |
| --- | --- | --- | --- | --- | --- | --- | --- |
| Predictors | Model fit | *k* | *b* | *SE* | β | $\eta_{p}^{2}$ | *p* |
| Single regression | | | | | | | |
|  | *R*² = .089***; *F*(1, 1295) = 127.90*** | 1296 |  |  |  |  |  |
| Year of data collection (2008-2023) |  |  | -0.347 | 0.031 | -.301 | .090 | <.001 |
| Multiple regression | | | | | | | |
|  | *R²* = .114***; *F* (3, 1130) = 49.75*** | 1133 |  |  |  |  |  |
| Year of data collection (2008-2023) |  |  | -0.322 | 0.032 | -.281 | .094 | <.001 |
| Sample mean age |  |  | -0.044 | 0.014 | -.088 | .004 | .001 |
| Percentage of women in sample |  |  | -0.024 | 0.005 | -.162 | .024 | <.001 |
| Regression on residualized values | | | | | | | |
|  | *R²* = .081***; *F* (1, 1132) = 101.20*** | 1133 |  |  |  |  |  |
| Year of data collection^a^ (2008-2023) |  |  | -0.316 | 0.031 | -.284 | .082 | <.001 |
| *Note.* Variables were weighted based on sample size; all *R²* are adjusted values; *k* = number of samples; *b* = unstandardized regression coefficient; *SE* = standard error of unstandardized coefficient; β = standardized regression coefficient; $\eta_{p}^{2}$ = partial eta squared; Variance Inflation Factors (VIFs) in multiple Regression were all <1.1.  ^a^ Adjusted for confounding variables (Percentage of women in sample & sample mean age, Scale, Items & Sample).  ****p* <.001. | | | | | | | |

| **Table S35**  *Single and multiple linear weighted meta-regression as well as residualized regressions for global samples on forced choice-based NPI-40 scores after the global financial crisis (since 2008).* | | | | | | | |
| --- | --- | --- | --- | --- | --- | --- | --- |
| Predictors | Model fit | *k* | *b* | *SE* | β | $\eta_{p}^{2}$ | *p* |
| Single regression | | | | | | | |
|  | *R²* = .013***; *F* (1, 582) = 87.33*** | 583 |  |  |  |  |  |
| Year of data collection (2008-2023) |  |  | -0.343 | 0.037 | -.368 | .130 | <.001 |
| Multiple regression | | | | | | | |
|  | *R²* = .173***; *F* (3, 507) = 36.56*** | 510 |  |  |  |  |  |
| Year of data collection (2008-2023) |  |  | -0.243 | 0.037 | -.259 | .102 | <.001 |
| Sample mean age |  |  | -0.109 | 0.018 | -.249 | .051 | <.001 |
| Percentage of women in sample |  |  | -0.029 | 0.006 | -.267 | .046 | <.001 |
| Regression on residualized values | | | | | | | |
|  | *R²* = .075***; *F* (1, 509) = 42.66*** | 510 |  |  |  |  |  |
| Year of data collection^a^ (2008-2023) |  |  | -0.238 | 0.036 | -.274 | .077 | <.001 |
| *Note.* Variables were weighted based on sample size; all *R²* are adjusted values; *k* = number of samples; *b* = unstandardized regression coefficient; *SE* = standard error of unstandardized coefficient; β = standardized regression coefficient; $\eta_{p}^{2}$ = partial eta squared; Variance Inflation Factors (VIFs) in multiple Regression were all <1.1.  ^a^ Adjusted for confounding variables (Percentage of women in sample & sample mean age).  ****p* <.001. | | | | | | | |

| **Table S36**  *Single and multiple linear weighted meta-regression as well as residualized regressions for global student samples on any NPI scores after the global financial crisis (since 2008).* | | | | | | | |
| --- | --- | --- | --- | --- | --- | --- | --- |
| Predictors | Model fit | *k* | *b* | *SE* | β | $\eta_{p}^{2}$ | *p* |
| Single regression | | | | | | | |
|  | *R²* = .037***; *F* (1, 610) = 24.29*** | 611 |  |  |  |  |  |
| Year of data collection (2008-2023) |  |  | -.202 | 0.041 | -.188 | .038 | <.001 |
| Multiple regression | | | | | | | |
|  | *R²* = .056***; *F* (3, 524) = 11.48*** | 527 |  |  |  |  |  |
| Year of data collection (2008-2023) |  |  | -0.166 | 0.044 | -.153 | .033 | <.001 |
| Sample mean age |  |  | 0.035 | 0.063 | .023 | <.001 | .577 |
| Percentage of women in sample |  |  | -0.029 | 0.007 | -.208 | .030 | <.001 |
| Regression on residualized values | | | | | | | |
|  | *R²* = .024***; *F* (1, 526) = 13.96*** | 527 |  |  |  |  |  |
| Year of data collection^a^ (2008-2023) |  |  | -0.161 | 0.043 | -.150 | .026 | <.001 |
| *Note.* Variables were weighted based on sample size; all *R²* are adjusted values; *k* = number of samples; *b* = unstandardized regression coefficient; *SE* = standard error of unstandardized coefficient; β = standardized regression coefficient; $\eta_{p}^{2}$ = partial eta squared; Variance Inflation Factors (VIFs) in multiple Regression were all <1.1.  ^a^ Adjusted for confounding variables (Percentage of women in sample & sample mean age).  ****p* <.001. | | | | | | | |

| **Table S37**  *Single and multiple linear weighted meta-regression as well as residualized regressions for global student samples on forced choice-based NPI-40 scores after the global financial crisis (since 2008).* | | | | | | | |
| --- | --- | --- | --- | --- | --- | --- | --- |
| Predictors | Model fit | *k* | *b* | *SE* | β | $\eta_{p}^{2}$ | *p* |
| Single regression | | | | | | | |
|  | *R²* = .052***; *F* (1, 336) = 19.48*** | 337 |  |  |  |  |  |
| Year of data collection (2008-2023) |  |  | -0.188 | 0.043 | -.204 | .055 | <.001 |
| Multiple regression | | | | | | | |
|  | *R²* = .110***; *F* (3, 284) = 12.77*** | 287 |  |  |  |  |  |
| Year of data collection (2008-2023) |  |  | -0.164 | 0.046 | -.173 | .060 | <.001 |
| Sample mean age |  |  | -0.072 | 0.085 | -.045 | .002 | .396 |
| Percentage of women in sample |  |  | -0.035 | 0.008 | -.315 | .061 | <.001 |
| Regression on residualized values | | | | | | | |
|  | *R²* = .370***; *F* (1, 286) = 12.04*** | 287 |  |  |  |  |  |
| Year of data collection^a^ (2008-2023) |  |  | -0.158 | 0.046 | -.173 | .040 | <.001 |
| *Note.* Variables were weighted based on sample size; all *R²* are adjusted values; *k* = number of samples; *b* = unstandardized regression coefficient; *SE* = standard error of unstandardized coefficient; β = standardized regression coefficient; $\eta_{p}^{2}$ = partial eta squared; Variance Inflation Factors (VIFs) in multiple Regression were all <1.1.  ^a^ Adjusted for confounding variables (Percentage of women in sample & sample mean age).  ****p* <.001. | | | | | | | |

**Table S38**

*Multiple linear weighted meta-regression on the effects of study inclusion in Twenge et al. (2008) and data collection year for U.S.-based student samples on forced choice-based NPI-40 scores from 1982 to 2023.*

| Predictors | Model fit | *k* | *b* | *SE* | β | $\eta_{p}^{2}$ | *p* |
| --- | --- | --- | --- | --- | --- | --- | --- |
|  | *R²* = .031***; *F* (3, 367) = 4.94*** | 370 |  |  |  |  |  |
| Year of data collection (1982-2023) |  |  | -0.083 | 0.024 | -.240 | .023 | < .001 |
| Included in Twenge et al. (2008) |  |  | -296.612 | 123.206 | .302 | < .001 | .218 |
| Year of data collection (1982-2023) *  Included in Twenge et al. (2008) |  |  | 0.148 | 0.061 | .042 | .016 | .017 |

*Note.* Variables were weighted based on sample size; all *R²* are adjusted values; *k* = number of samples; *b* = unstandardized regression coefficient; *SE* = standard error of unstandardized coefficient; β = standardized regression coefficient; $\eta_{p}^{2}$ = partial eta squared; Variance Inflation Factors (VIFs) in multiple Regression were all <1.1.

****p* <.001.

**Table S39**

*Multiple linear weighted meta-regression on the effects of study inclusion in Twenge et al. (2008) and data collection year for global samples on any NPI scores from 1982 to 2023.*

| Predictors | Model fit | *k* | *b* | *SE* | β | $\eta_{p}^{2}$ | *p* |
| --- | --- | --- | --- | --- | --- | --- | --- |
|  | *R²* = .054***; *F* (3, 11617) = 31.66*** | 11620 |  |  |  |  |  |
| Year of data collection (1982-2023) |  |  | -0.166 | 0.018 | -.304 | .051 | < .001 |
| Included in Twenge et al. (2008) |  |  | -416.909 | 181.663 | .401 | .002 | .199 |
| Year of data collection (1982-2023) *  Included in Twenge et al. (2008) |  |  | 0.208 | 0.091 | .381 | .003 | .022 |

*Note.* Variables were weighted based on sample size; all *R²* are adjusted values; *k* = number of samples; *b* = unstandardized regression coefficient; *SE* = standard error of unstandardized coefficient; β = standardized regression coefficient; $\eta_{p}^{2}$ = partial eta squared; Variance Inflation Factors (VIFs) in multiple Regression were all <1.1.

****p* <.001.

**Table S40**

*Multiple linear weighted meta-regression on the effects of age and data collection year for U.S.-based student samples on forced choice-based NPI-40 scores from 1982 to 2023.*

| Predictors | Model fit | *k* | *b* | *SE* | β | $\eta_{p}^{2}$ | *p* |
| --- | --- | --- | --- | --- | --- | --- | --- |
|  | *R²* = .044**; *F* (3, 249) = 3.81** | 252 |  |  |  |  |  |
| Year of data collection (1982-2023) |  |  | -0.232 | 0.471 | -.248 | .042 | .001 |
| Sample mean age |  |  | -14.533 | 46.391 | .032 | .001 | .731 |
| Year of data collection (1982-2023) *   Sample mean age |  |  | 0.007 | 0.023 | .029 | .001 | .753 |

*Note.* Variables were weighted based on sample size; all *R²* are adjusted values; *k* = number of samples; *b* = unstandardized regression coefficient; *SE* = standard error of unstandardized coefficient; β = standardized regression coefficient; $\eta_{p}^{2}$ = partial eta squared; Variance Inflation Factors (VIFs) in multiple Regression were all <1.1.

***p* <.01.

**Table S41**

*Multiple linear weighted meta-regression on the effects of age and data collection year for global samples on any NPI scores from 1982 to 2023.*

| Predictors | Model fit | *k* | *b* | *SE* | β | $\eta_{p}^{2}$ | *p* |
| --- | --- | --- | --- | --- | --- | --- | --- |
|  | *R²* = .059***; *F* (3, 1340) = 27.9*** | 1343 |  |  |  |  |  |
| Year of data collection (1982-2023) |  |  | -0.216 | 0.066 | -.273 | .052 | <.001 |
| Sample mean age |  |  | -5.048 | 5.112 | -.094 | .006 | <.001 |
| Year of data collection (1982-2023) *   Sample mean age |  |  | 0.002 | 0.003 | .039 | .001 | .327 |

*Note.* Variables were weighted based on sample size; all *R²* are adjusted values; *k* = number of samples; *b* = unstandardized regression coefficient; *SE* = standard error of unstandardized coefficient; β = standardized regression coefficient; $\eta_{p}^{2}$ = partial eta squared; Variance Inflation Factors (VIFs) in multiple Regression were all <1.1.

****p* <.001.

**Table S42**

*Multiple linear weighted meta-regression on the effects of NPI-type (forced-choice NPI-40 vs. else) and data collection year on global NPI scores from 1982 to 2023.*

| Predictors | Model fit | *k* | *b* | *SE* | β | $\eta_{p}^{2}$ | *p* |
| --- | --- | --- | --- | --- | --- | --- | --- |
|  | *R²* = 0.066***; *F* (3, 1617) = 38.94*** | 1620 |  |  |  |  |  |
| Year of data collection (1982-2023) |  |  | -0.173 | 0.024 | -.317 | .052 | <.001 |
| NPI type |  |  | -28.739 | 64.833 | -.265 | .017 | <.001 |
| Year of data collection (1982-2023) *  NPI type |  |  | 0.014 | 0.032 | .025 | .001 | .670 |

*Note.* Variables were weighted based on sample size; all *R²* are adjusted values; *k* = number of samples; *b* = unstandardized regression coefficient; *SE* = standard error of unstandardized coefficient; β = standardized regression coefficient; $\eta_{p}^{2}$ = partial eta squared; Variance Inflation Factors (VIFs) in multiple Regression were all <1.1.

****p* <.001.

**Table S43**

*Multiple linear weighted meta-regression on the effects of sample type (student samples vs. else) and data collection year for global samples on any NPI scores from 1982 to 2023.*

| Predictors | Model fit | *k* | *b* | *SE* | β | $\eta_{p}^{2}$ | *p* |
| --- | --- | --- | --- | --- | --- | --- | --- |
|  | *R²* = 0.058***; *F* (3, 1617) = 34.13*** | 1620 |  |  |  |  |  |
| Year of data collection (1982-2023) |  |  | -0.223 | 0.027 | -.409 | .052 | <.001 |
| Sample type |  |  | -256.267 | 68.278 | .001 | .017 | .994 |
| Year of data collection (1982-2023) * Sample type |  |  | 0.127 | 0.035 | .234 | .001 | <.001 |

*Note.* Variables were weighted based on sample size; all *R²* are adjusted values; *k* = number of samples; *b* = unstandardized regression coefficient; *SE* = standard error of unstandardized coefficient; β = standardized regression coefficient; $\eta_{p}^{2}$ = partial eta squared; Variance Inflation Factors (VIFs) in multiple Regression were all <1.1.

****p* <.001.
